# Redox activation of JNK2α2 mediates thyroid hormone-stimulated proliferation of neonatal murine cardiomyocytes

**DOI:** 10.1038/s41598-019-53705-1

**Published:** 2019-11-27

**Authors:** Lin Tan, Nikolay Bogush, Hussain Naib, Jennifer Perry, John W. Calvert, David I. K. Martin, Robert M. Graham, Nawazish Naqvi, Ahsan Husain

**Affiliations:** 1 Department of Medicine (Cardiology), Emory University School of Medicine, Atlanta, Georgia USA; 2 Department of Animal Resources, Emory University School of Medicine, Atlanta, Georgia USA; 3 Department of Surgery, Emory University School of Medicine, Atlanta, Georgia USA; 4 Children’s Hospital Oakland Research Institute, Oakland, California USA; 5 Victor Chang Cardiac Research Institute, Darlinghurst, New South Wales Australia

**Keywords:** Cell-cycle exit, Cardiac regeneration

## Abstract

Mitochondria-generated reactive oxygen species (mROS) are frequently associated with DNA damage and cell cycle arrest, but physiological increases in mROS serve to regulate specific cell functions. T3 is a major regulator of mROS, including hydrogen peroxide (H_2_O_2_). Here we show that exogenous thyroid hormone (T3) administration increases cardiomyocyte numbers in neonatal murine hearts. The mechanism involves signaling by mitochondria-generated H_2_O_2_ (mH_2_O_2_) acting via the redox sensor, peroxiredoxin-1, a thiol peroxidase with high reactivity towards H_2_O_2_ that activates c-Jun N-terminal kinase-2α2 (JNK2α2). JNK2α2, a relatively rare member of the JNK family of mitogen-activated protein kinases (MAPK), phosphorylates c-Jun, a component of the activator protein 1 (AP-1) early response transcription factor, resulting in enhanced insulin-like growth factor 1 (IGF-1) expression and activation of proliferative ERK1/2 signaling. This non-canonical mechanism of MAPK activation couples T3 actions on mitochondria to cell cycle activation. Although T3 is regarded as a maturation factor for cardiomyocytes, these studies identify a novel redox pathway that is permissive for T3-mediated cardiomyocyte proliferation—this because of the expression of a pro-proliferative JNK isoform that results in growth factor elaboration and ERK1/2 cell cycle activation.

## Introduction

At birth, cardiomyocytes are relatively quiescent compared to those of the fetal heart^1^. Cell cycle activity markedly increases in these cells between postnatal day-4 (P4) and P6^1^. This reactivation of the cell cycle, in rodents, during the neonatal period, culminates in cell replication^2–6^ and binucleation^1,3,4^ and then, after P6, cardiomyocytes become uniformly quiescent^7^.

Two recent reports propose an intriguing mechanism that places enhanced mitochondrial biogenesis in neonatal cardiomyocytes at the nexus between cardiomyocyte maturation and cell cycle block. Hirose *et al*.^8^ proposed that a rise in circulating T3, starting at P4, causes cardiomyocyte maturation, which upregulates α-myosin heavy chain (αMHC) gene (*Myh6*) expression and shifts metabolism from glycolysis to oxidative phosphorylation. In contrast, studies by Puente *et al*.^9^ implicate the increase in oxygen tension that occurs after birth in the generation of reactive oxygen species (ROS). One of these, mitochondria-generated hydrogen peroxide (mH_2_O_2_), they proposed, activates the DNA damage response (DDR) pathway^9^, its mediators including p-ATM, p-CHK2, p-p53 and p21, which then perpetually maintains cell cycle block in mature cardiomyocytes.

Here we evaluate the effect of exogenously administered T3 in regulating neonatal murine cardiomyocyte proliferative capacity. We show that T3 increases the expression of *Igf1*, which encodes insulin-like growth factor-1 (IGF-1), a mitogen for murine neonatal cardiomyocytes^10^. Moreover, *in vivo*, T3 increases cardiomyocyte numbers by expanding the population of mononuclear cardiomyocytes, without affecting nuclear ploidy. This T3 proliferative response involves mH_2_O_2_-mediated redox activation of c-Jun N-terminal kinase-2 (JNK2α2) by peroxiredoxin-1 (Prx1); a mechanism coupling T3-stimulated mitochondrial biogenesis with activation of the cell cycle.

## Results

### T3 regulates genes critical for maturation and mitosis in neonatal cardiomyocytes

**Figure 1:**
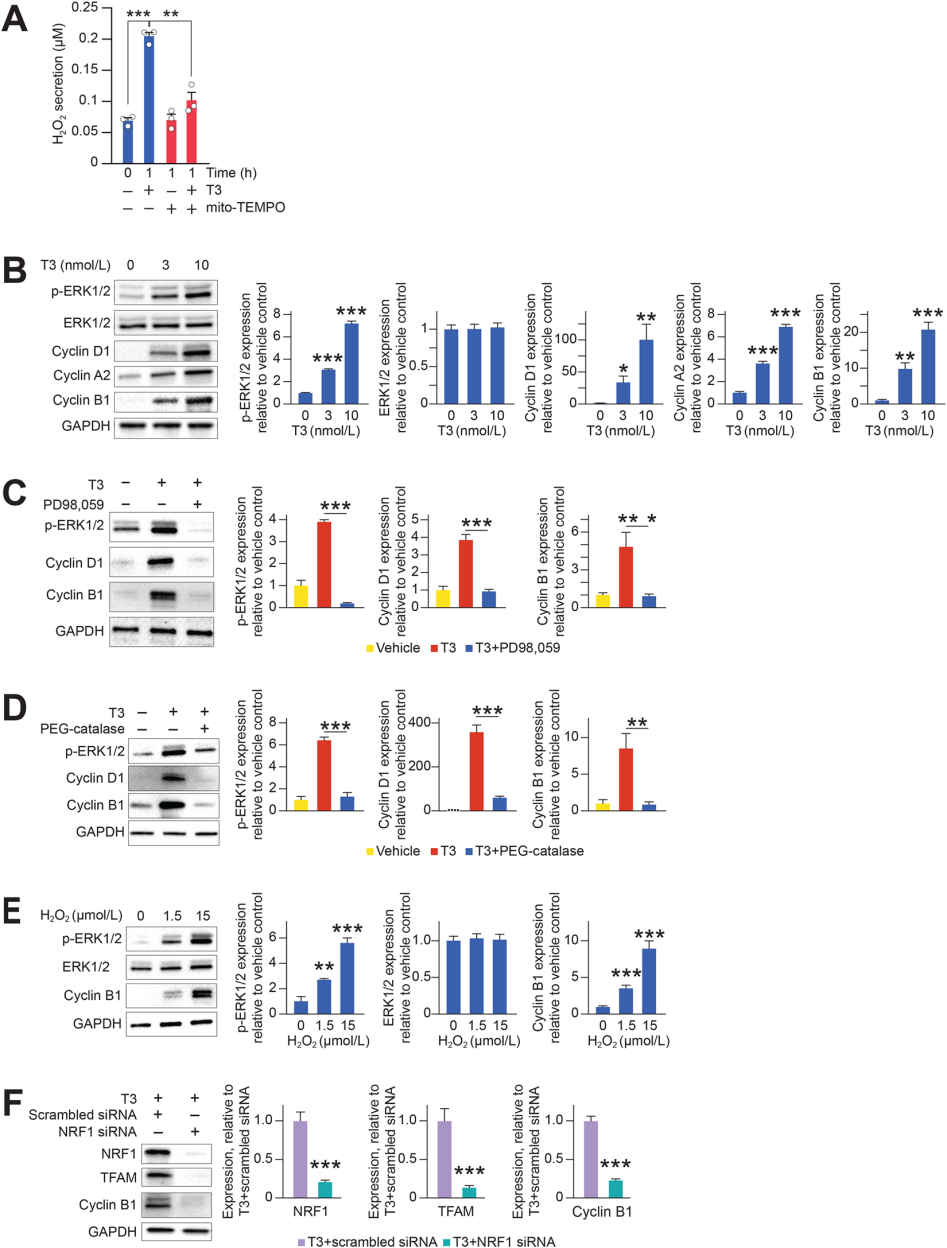
T3-dependent mitogenic signaling requires H_2_O_2_ and mitochondrial biogenesis. (**A**) Mito-TEMPO impedes the increases in media H_2_O_2_ levels that results due to T3 (10 nmol/L) treatment of neonatal cardiomyocytes in culture. (**B**) Representative immunoblots and quantitative analyses showing that T3 treatment to neonatal cardiomyocytes in culture increases phosphorylated-ERK (p-ERK1/2 T202/Y204) protein levels as well as cyclins D1, A2 and B1 in a concentration-dependent manner. (**C**) Inhibition of T3 (10 nmol/L)-dependent ERK1/2 activation by the ERK1/2 inhibitor (PD98059) repressed cyclin D1/B1 expression. (**D**) Scavenging H_2_O_2_ with PEG-catalase (200 U/ml) inhibited T3 (10 nmol/L)-mediated p-ERK1/2 and cyclin D1/B1 accumulation. (**E**) Treatment of neonatal cardiomyocytes with low concentrations of H_2_O_2_ (1.5–15 µmol/L) increases phosphorylated-ERK (p-ERK1/2 T202/Y204) and cyclin B1 protein levels. (**F**) NRF1 siRNA knockdown prevented TFAM expression and simultaneously reduced cyclin B1 accumulation as compared to treatment with scrambled siRNA. *P < 0.05; **P < 0.01; ***P < 0.001 compared to groups indicated on the graph. *n* = 4/group. Error bars indicate SEM.

To determine the effect of T3 on cardiomyocyte maturation and proliferation, we used primary cultures of mouse neonatal (P2-P3) cardiomyocytes maintained in serum-free media. Cardiomyocytes were exposed to T3 or vehicle for 5 h, and gene expression was analyzed by RT-qPCR. T3 upregulated multiple genes that promote cardiomyocyte maturation. These include genes that promote mitochondrial biogenesis, oxidative phosphorylation and lipid biosynthesis (Supplementary Table S1) and genes that are associated with cardiomyocyte differentiation (Supplementary Table S2). In keeping with its effects on genes that promote oxidative phosphorylation (Supplementary Table S1), T3 increased the secretion of H_2_O_2_ into the extracellular space by 3-fold to ~0.25 µmol/L (Fig. 1A), which was inhibited by mito-TEMPO, a mitochondria-targeted antioxidant (Fig. 1A). T3 did not increase the expression of genes that promote DDR, but it increased *Ppm1d* expression. *Ppm1d* (encodes Wip1 phosphatase) relieves checkpoint arrest by de-phosphorylating DDR-pathway components^11^. T3 increased the expression of *Igf1* and *Igf1r* as well as the expression of genes that promote G_1_/S, S, G_2_/M and M phases of the cell cycle (Supplementary Table S3) and it stimulated the expression of genes that are critical for cytokinesis or are positive regulators of cytokinesis (e.g., *Anln*, *AurkA*, *AurkB*, *Ect2*, *Igf1r*, *Incenp* and *Plk1*) (Supplementary Table S3). Several of these targets were also validated at the protein level by quantitative immunoblot analysis (Supplementary Table S4).

### mH_2_O_2_ is required for T3-stimulated proliferative signaling in cardiomyocytes

H_2_O_2_ can stimulate or inhibit cell proliferation depending on concentration and cellular context. We asked if proliferative signaling in neonatal cardiomyocytes by T3 requires H_2_O_2_ generation. *In vitro*, T3 increased the phosphorylation of ERK1/2 (Fig. 1B)—which is necessary for G_1_/S phase transition^12^—and it increased the abundance of cyclins D1/A2/B1 (Fig. 1B). ERK1/2 inhibition with PD98,059 abrogated this T3 effect (Fig. 1C). H_2_O_2_ scavenging with polyethylene glycol (PEG)-catalase (a cell permeant form of catalase) inhibited T3-stimulated ERK1/2 phosphorylation and accumulation of cyclins (Fig. 1D). In contrast, exposing cardiomyocytes to H_2_O_2_ (1.5–15 µmol/L) caused ERK1/2 phosphorylation and increased expression of cyclin B1 (Fig. 1E). We take these effects of T3 on ERK1/2 activation (by phosphorylation) and cell cycle-promoting cyclin expression to indicate that H_2_O_2_ is necessary and sufficient for T3 proliferative signaling in neonatal cardiomyocytes.

The PPARγ coactivator 1α (PGC-1α) and nuclear respiratory factor-1 (NRF1) together increase transcription of mitochondrial factor A (*Tfam*), which promotes mitochondrial biogenesis^13^. We found that T3-induced proliferative signaling was associated with increased expression of PGC-1α, NRF1, and TFAM and of genes that regulate oxidative phosphorylation and lipid biosynthesis (Supplementary Table S1). In T3-treated cardiomyocytes, NRF1 depletion with siRNA inhibited the accumulation of TFAM and cyclin B1 (Fig. 1F). Thus, in neonatal cardiomyocytes, nucleus-to-mitochondria signaling enhances mH_2_O_2_ production, which mediates T3-stimulated proliferative signaling in cardiomyocytes.

### T3 is a mitogen for neonatal cardiomyocytes

Cardiomyocyte numbers increase in mice during the neonatal period^2–6^. This increase, which expands the cardiomyocyte population by ~30%, occurs soon after P4 (Supplementary Fig. S1), concurrent with the initial postnatal rise in circulating T3.

Here we questioned if exogenous T3 administration at P2 and P3 increases cardiomyocyte proliferation (between P2 and P7), as our *in vitro* data predicts, or alternatively activates cell cycle checkpoints causing an increase in ploidy, binucleation and a diminution in cardiomyocyte numbers, as would be expected based on the work of Hirose *et al*.^8^, which proposes that a developmental rise in T3 at ~P4/P5 causes cell cycle arrest.

**Figure 2:**
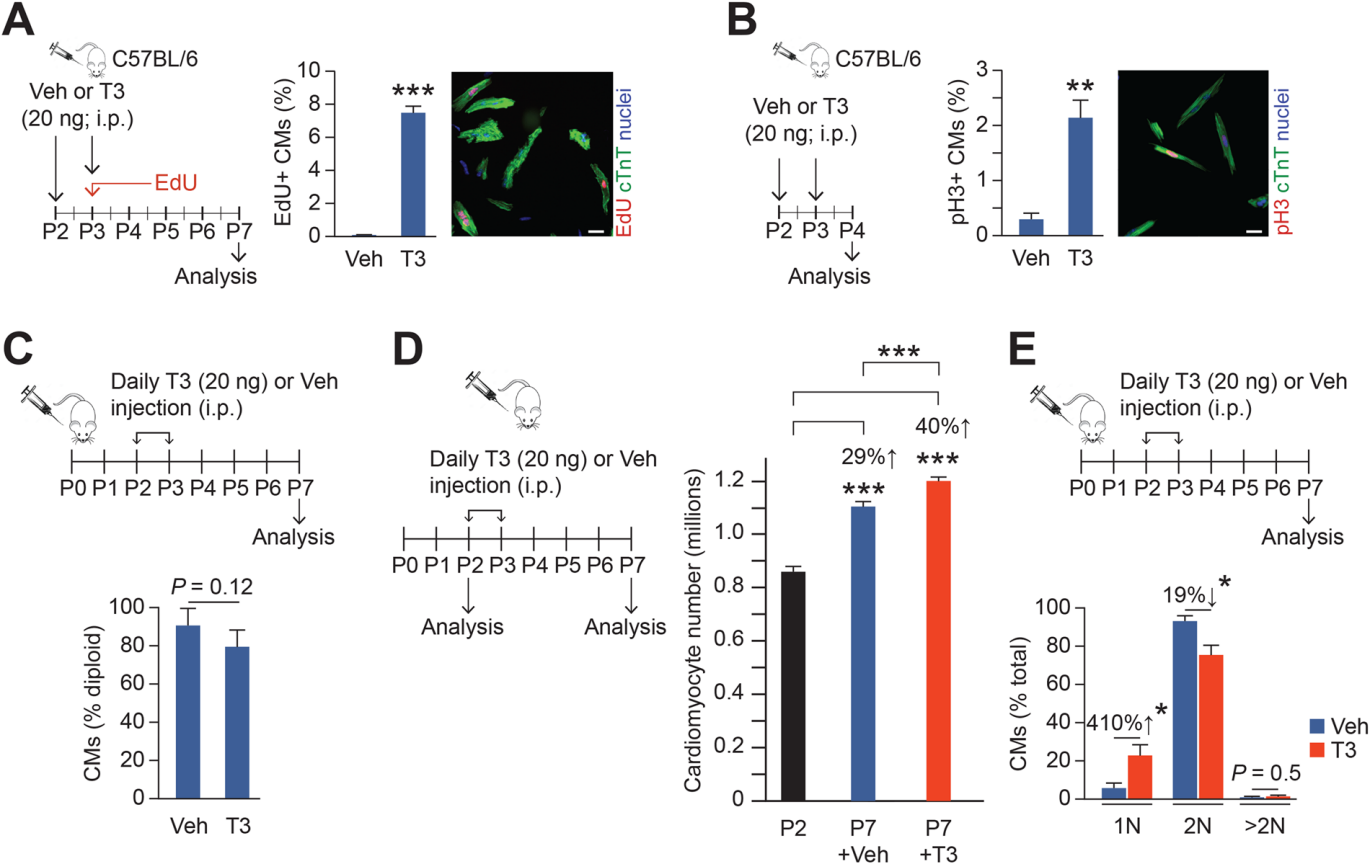
T3 acts as a mitogen for neonatal cardiomyocytes *in vivo*. (**A**,**B**) Protocol for the acute treatment of neonatal mice with T3 and injecting EdU for detection of S-phase is shown in the schematic. Cardiomyocytes (CMs) were identified by cTnT labeling. T3 increases DNA synthesis (EdU + CMs) (**A**) and mitosis (pH3 + CMs) (**B**) of neonatal cardiomyocytes *in vivo*. *P*-values are shown on each graph. White bars represent 20 µm. (**C**) Exogenous *in vivo* T3-treatment to the neonatal mice, as per protocol shown by the schematic, does not impact nuclear ploidy. *n* = 5/group. (**D**) T3 treatment increases ventricular cardiomyocyte (CM) numbers between P2 and P7. *n* = 11–12/group. (**E**) T3 treatment increases the percentage of mononuclear cardiomyocytes and decreases binuclear cardiomyocytes. *n* = 5/group. Error bars indicate SEM. *P < 0.05, **P < 0.01, and ***P < 0.001 compared to control or groups indicated on the graph.

T3 treatment, *in vivo*, resulted in an increased S phase (estimated by EdU-labeling) in cardiomyocytes, from 0.01% to >7% (Fig. 2A), as well as an ~7-fold increase in mitosis (estimated by PH3-labeling) (Fig. 2B). Moreover, estimation of nuclear ploidy did not show evidence of G_2_/M block (Fig. 2C).

Next, we assessed the effect of T3 on cardiomyocyte numbers in neonatal hearts. We found that T3 administration increased cardiomyocyte numbers by ~10% (~100,000 cells; *P* < 0.001) (Fig. 2D) over and above the 30% developmental increase in the cardiac endowment of the heart that occurs between birth and P7. Cardiomyocyte number was determined by enzymatic digestion of hearts using a Langendorf preparation followed by mechanical disaggregation to create a cardiomyocyte suspension. Cardiomyocytes, readily distinguished from non-cardiomyocytes by their size and rod shape, were counted using a hemocytometer. Efficacy of enzymatic digestion was determined to ascertain if differences in cardiomyocyte numbers in vehicle versus T3-treated P7 mice might merely be due to variations in digestion. At P7, digestion efficiencies were ~97% for both mouse groups (97.0 ± 1.1% and 97.1 ± 1.4% in vehicle- and T3-treated hearts, respectively; *P* = 0.94), with microscopic examination revealing that residual undigested tissue was almost entirely from cardiac valves and blood vessels. Moreover, over-digestion did not generate higher cardiomyocyte yields (not shown).

The experiments described above indicate that exogenous T3 promotes cardiomyocyte cytokinesis in neonatal hearts. If cytokinesis is associated with a partial block in the transition from karyokinesis to cytokinesis it would promote multinucleation, which did not occur (Fig. 2E).

**Figure 3:**
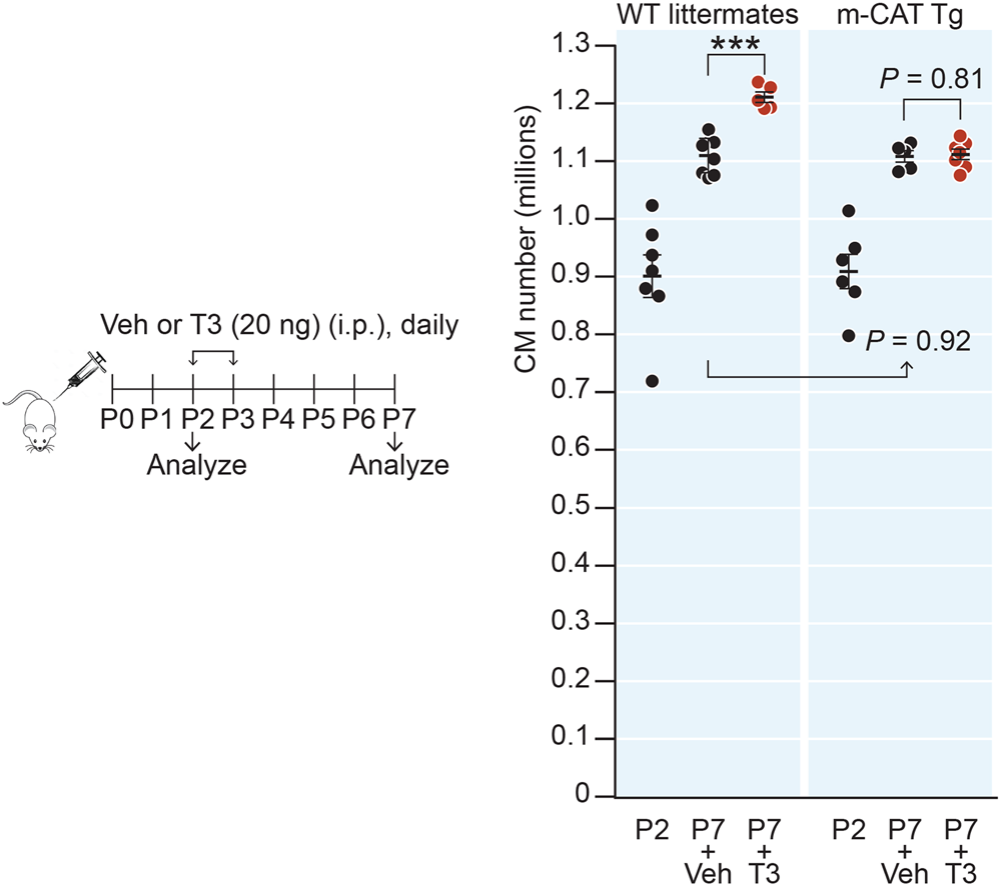
Scavenging H_2_O_2_ in mitochondria suppresses T3-stimulated but not developmental cardiomyocyte expansion in neonates. Cardiomyocyte numbers in vehicle or T3-treated mice showing the effect of genetically targeted H_2_O_2_-ROS scavenger, catalase, to the mitochondria (m-CAT-Tg). Error bars indicate SEM. ***P < 0.001.

Our cell culture studies predict that the T3 mitogenic response in neonatal cardiomyocytes is mediated by mH_2_O_2_. To explore the *in vivo* importance of this mechanism, we used a genetic model in which catalase is targeted to the mitochondria (m-CAT) to scavenge mH_2_O_2_^14^. We found that cardiomyocyte numbers were not significantly different between m-CAT-transgenic mice (m-CAT-Tg) and their wild type (WT) littermates either immediately after birth, at P2, or at P7. However, although T3 administration at P2 and P3 further increased cardiomyocyte numbers in WT mice by P7, it failed to do so in m-CAT-Tg mice (Fig. 3). These results show that the developmental increase in cardiomyocyte numbers during the neonatal period is unaffected by mH_2_O_2_ scavenging, but the T3 mitogenic effect in these cells requires mH_2_O_2_.

### mH_2_O_2_/IGF-1 mediates T3-stimulated proliferative signaling in neonatal cardiomyocytes

**Figure 4:**
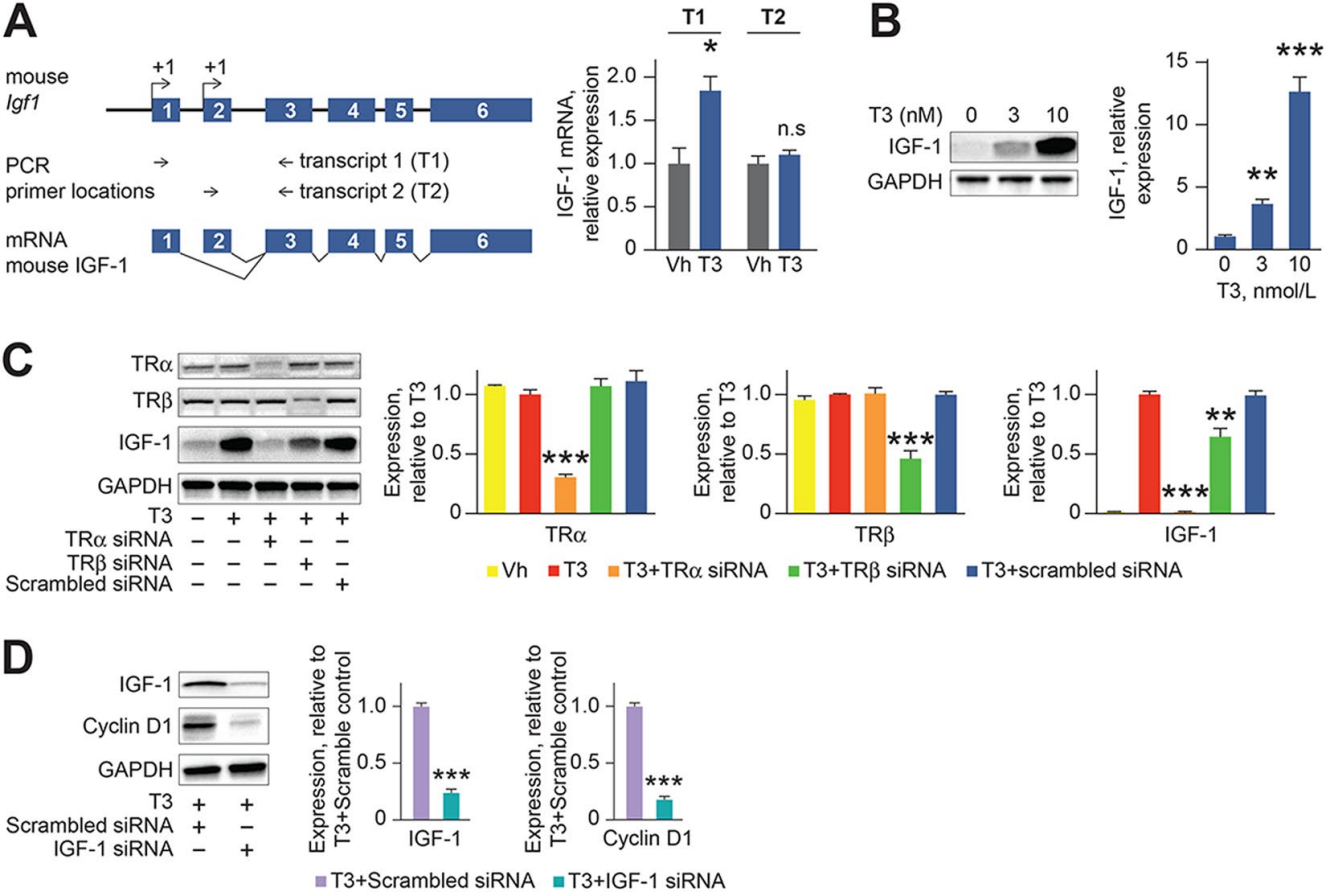
T3-stimulated proliferative signaling in neonatal cardiomyocytes requires IGF-1 and T3 receptor-α. (**A**) Schematic showing the location of two potential *Igf1* transcription start sites and the two discriminating primer pairs for quantification of distinct transcripts. mRNA quantification by RT-qPCR of *Igf1* transcripts showing that T3 enhances the transcription of *Igf1* from the proximal promoter. (**B**) Representative immunoblot and quantitative analyses of neonatal cardiomyocytes lysate showing that T3 increases IGF-1 formation in a dose dependent manner. (**C**) Knockdown of TRα, and to a lesser extent TRβ, prevents T3-dependent IGF-1 formation. (**D**) Representative immunoblot and quantitative analyses of neonatal cardiomyocyte lysate showing that knockdown of IGF-1 with siRNA prevents T3-dependent induction of cyclin D1. Error bars indicate SEM. *n* = 4 biological replicates/group. n.s., nonsignificant; **P < 0.01, ***P < 0.001, compared to controls.

T3 increased *Igf1* expression in neonatal cardiomyocytes (Supplementary Table S3). IGF signaling is required for zebrafish cardiomyocyte proliferation during heart development and regeneration^15^. We therefore investigated the role of IGF-1 in the T3 mitogenic response in neonatal murine cardiomyocytes. *Igf1* has two mutually exclusive leader exons that each have multiple promoter sites, which are variably used^16^. In osteoblasts, T3 binds thyroid receptor-α (TRα) on the thyroid response element (TRE) on intron 1 of *Igf1* to stimulate transcription from the distal promoter^17^. We found that in neonatal cardiomyocytes, T3 increased IGF-1 mRNA transcription from the proximal, but not the distal promoter (Fig. 4A). Moreover, T3 (3–10 nmol/L) stimulated IGF-1 formation (Fig. 4B); a response mediated by TRα but not TRβ (Fig. 4C). IGF-1 depletion with siRNA inhibited T3-stimulated accumulation of cyclin D1 (Fig. 4D), indicating that T3 proliferative signaling in cardiomyocytes requires IGF-1 formation.

Analysis of the proximal *Igf1* promoter sequence using Alibaba2 predicted multiple activator protein 1 (AP-1)/c-Jun binding sites (Supplementary Fig. S2A). c-Jun is a component of the AP1 complex. AP1 inhibition with SR11302 prevented T3-stimulated IGF-1 expression in cardiomyocytes *in vitro* (Supplementary Fig. S2B) suggesting that AP1 activation mediates T3-stimulated IGF-1 formation. In keeping with this conclusion, T3 increased c-Jun (S73) phosphorylation (Supplementary Fig. S2C). To understand the order of T3 signaling events in cardiomyocytes, we inhibited signaling intermediates. We found that c-Jun activation was prevented by H_2_O_2_ scavenging, but not by IGF-1 inhibition (Supplementary Fig. S2C). In contrast, T3-induced IGF-1 expression was inhibited by siRNA-mediated c-Jun depletion (Supplemental Fig. S2D). These experiments established that T3 first stimulates H_2_O_2_ generation and then H_2_O_2_ stimulates c-Jun activation, and that these signaling events precede IGF-1 formation.

**Figure 5:**
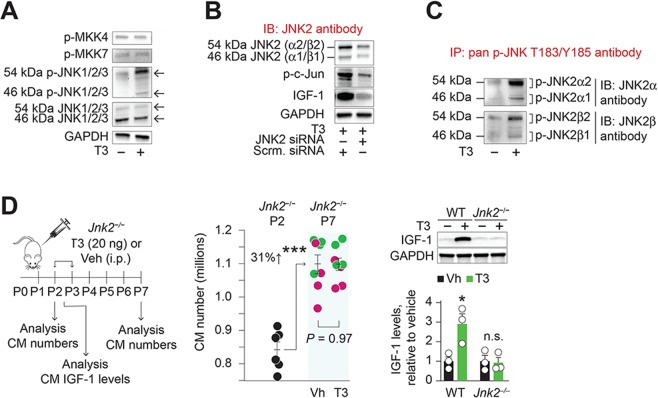
JNK2 activation mediates T3-stimulated cardiomyocyte proliferation in neonatal cardiomyocytes. (**A**) T3-stimulates ~54 kDa JNK phosphorylation, but not that of MKK4 and 7, indicating non-canonical activation of JNK by T3. (**B**) T3-stimulated c-Jun phosphorylation, the downstream effector of JNK activation and IGF-1 expression require JNK2. (**C**) Immunoprecipitation studies in lysates from neonatal cardiomyocytes show that T3 mainly phosphorylates the ~54 kDa JNK2α2 isoform (JNK2α2). In A–C, immunoblots are representative of 2 independent experiments. (**D**) Genetic *Jnk2* deletion inhibits T3-stimulated cardiomyocyte proliferation but not developmental increases in cardiomyocyte numbers in neonates. Individual colors represent data from pups from unique litters. *Jnk2* deletion also inhibits *in vivo* T3-stimulated IGF-1 expression in cardiomyocytes. Representative immunoblots of cardiomyocyte lysates are shown above the graph. In the latter experiment, T3 or vehicle treatment was given on P2 and the hearts harvested 12 h later for immunoblotting. *P < 0.05, ***P < 0.001, compared to WT littermates; n.s., nonsignificant. Error bars indicate SEM.

Because H_2_O_2_ is required for T3-mediated IGF-1 formation, we asked if c-Jun or any of the kinases in the cascade that leads to c-Jun phosphorylation might be the target of redox signaling by H_2_O_2_. c-Jun is phosphorylated by JNK1–3 isoforms, which in turn are activated by mitogen-activated protein kinase kinases 4 and 7 (MKK4/7)^18^. We found that T3 increased the abundance of phosphorylated JNK (T183/T185) in cardiomyocytes, but not that of MKK4/7 (Fig. 5A). At least 10 different splicing isoforms of JNKs regulate apoptosis, cellular proliferation, and differentiation^18^: JNK1 preferentially mediates apoptosis, while JNK2 is associated with cellular proliferation^19^. Although ~46 kDa isoforms predominate in cardiomyocytes, T3 phosphorylated mainly ~54 kDa JNK isoform(s). JNK2 depletion with siRNA inhibited phosphorylation of c-Jun, as well as inhibiting IGF-1 accumulation in T3-treated cardiomyocytes (Fig. 5B). The ~54 kDa type 2 JNKs consist of α2 and β2 isoforms, while the α1 and β1 isoforms are ~46 kDa. T3 signaling mainly phosphorylated the 54 kDa JNK2α-isoform (JNK2α2) (Fig. 5C). T3-stimulated JNK phosphorylation was attenuated by H_2_O_2_ scavenging, but not by inhibition of IGF-1 (Supplementary Fig. S3A), placing T3-stimulated JNK phosphorylation downstream of H_2_O_2_ generation and upstream of IGF-1 formation. Supporting this conclusion, low concentrations of H_2_O_2_ (1.5 µmol/L) mimicked the T3 effect on ~54 kDa JNK phosphorylation (Supplementary Fig. S3B).

Taken together, these studies indicate that T3 activation of c-Jun is dependent on H_2_O_2_ and JNK2, and that H_2_O_2_ is both necessary and sufficient for JNK2 phosphorylation. This redox-activation mechanism differs significantly from canonical pathways of JNK activation that promiscuously activate multiple JNK isoforms^18,19^.

To further probe the involvement of JNK2 in T3-mediated cardiomyocyte replication, we used *Jnk2*^−/−^ mice^20^. The basal cardiomyocyte population at P2, and the developmental expansion of cardiomyocyte numbers in the neonatal period between P2 and P7, were unaffected by *Jnk2* deletion (Fig. 5D). However, in *Jnk2*^−/−^ mice, exogenous T3 administration failed to increase cardiomyocyte replication (Fig. 5D) and IGF-1 levels (Fig. 5D).

**Figure 6:**
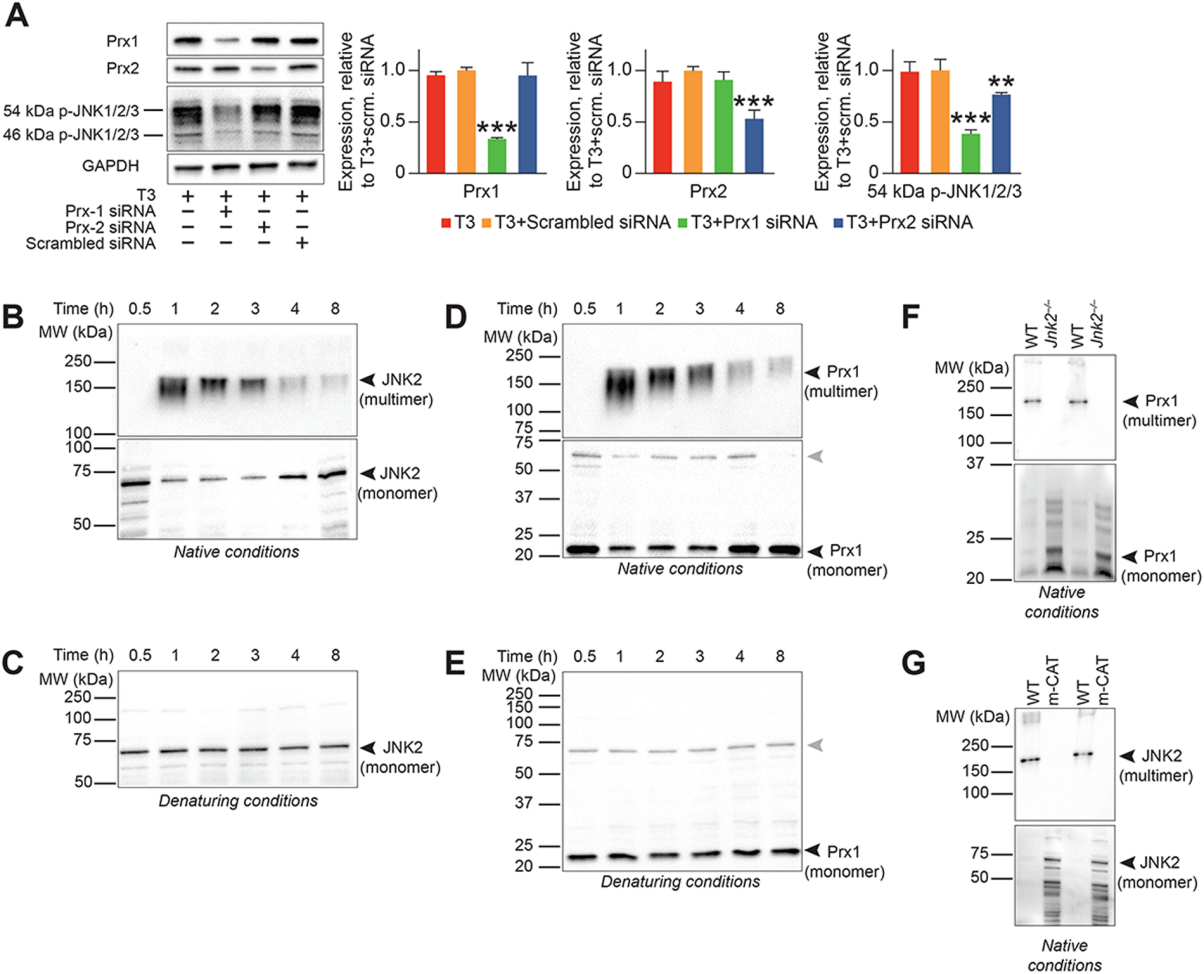
The H_2_O_2_ sensor Prx1 plays a major role in T3-dependent JNK2 phosphorylation. (**A**) Representative immunoblot of lysates from cultured neonatal cardiomyocytes showing that knockdown of Prx1 and not Prx2 mainly prevents T3-dependent high molecular weight (54 kDa isoform) JNK phosphorylation. Representative of 4 biological replicates. Error bars indicate SEM. **P < 0.01; ***P < 0.001. Quantitative analyses of immunoblots is also shown. (**B**) Immunoblot of lysates under non-reducing conditions, to prevent dissociation of disulfide bridges, shows that T3-treatment results in a JNK2 multimer of ~160 kDa within 1 h with a concomitant decrease in the abundance of JNK2 monomer. The multimer dissociates over the next 3–4 h with the abundance of the ~60 kDa monomer increasing concomitantly. (**C**) Under reducing SDS-PAGE condition JNK2 fractionates as a single ~60 kDa species at all times. (**D**) Immunoblotting with Prx1-specific antibody under non-reducing conditions shows that T3-treatment results in a Prx1 multimer of ~160 kDa within 1 h, with a concomitant decrease in Prx1 monomer. The multimer dissociates over the next 2–3 h, which is associated with the concomitant detection of an ~22 kDa monomeric Prx1 species. (**E**) Immunoblotting T3-treated cardiomyocyte lysates under reducing SDS-PAGE condition shows that Prx1 fractionates as a single ~22 kDa species at all times. (**F**,**G**) *In vivo*, 1-h T3 treatment induces Prx1/JNK2 multimer formation (~160 kDa) in WT mice but not in *Jnk*2^−/−^ (**F**) or m-CAT transgenic mice (**G**). Immunoblots in (**B**–**G**) are representative of 2–3 biological replicates.

These findings raise the question of how H_2_O_2_ induces JNK2 phosphorylation, which then drives cardiomyocyte proliferation. Unlike other JNK isoforms, JNK2α2 undergoes dimerization-dependent trans-autophosphorylation/autoactivation^19^. JNK2α2 dimers (held together by non-covalent interactions) are unstable, hence, trans-autophosphorylation is seen only at high JNK2α2 concentrations^19^. We considered the possibility that H_2_O_2_ might stabilize JNK2α2 dimers by inducing inter-molecular disulfide bond formation. But it seemed unlikely that H_2_O_2_ levels of only ~0.25 µmol/L, as induced by T3 (Fig. 1A), would be sufficient to directly oxidize JNK2α2. At this low concentration, it is more likely that H_2_O_2_ reacts with highly sensitive thiol peroxidases, such as peroxiredoxins (Prxs)^21,22^, rather than directly with JNK2. Consistent with this notion, siRNA-mediated depletion of Prx1 inhibited T3-mediated JNK phosphorylation (Fig. 6A).

Peroxiredoxins can convert the incoming oxidizing equivalent (the peroxide) into one that is transmissible from protein to protein: the disulfide bond^21,22^. These reactions generate Prx–target protein multimers, stabilized by disulfide bonds, as reaction intermediates^21–23^. To look for such intermediates, we probed lysates from T3-treated cardiomyocytes under non-reducing conditions. An ~160 kDa JNK2 multimer first appeared between 0.5 and 1 h after T3 treatment, but then decomposed over the next 3–4 h (Fig. 6B). Temporal changes in the abundance of the JNK2 multimer were reciprocally related to the abundance of its ~60 kDa monomer (Fig. 6B). Under reducing conditions, only JNK2 monomers were observed (Fig. 6C), indicating stabilization of the JNK2 multimer by disulfide bonds. In the same T3-treated cell lysates, we noted that the formation and decomposition of a Prx1 multimer (~160 kDa) was concordant with the disappearance and reappearance of its monomer (~22 kDa) (Fig. 6D,E). The kinetics of Prx1 multimer formation was similar to that of JNK2 (Fig. 6B,C). Consideration of monomer/multimer sizes is consistent with the notion that the multimer is composed of 2 molecules each of JNK2 and Prx1. The Prx1 multimer is stabilized by disulfide bonds (Fig. 6E) and requires JNK2 (Fig. 6F). The finding that T3-stimulated JNK2 multimerization does not occur in cardiomyocytes of m-CAT-Tg mice supports a role for mH_2_O_2_ in JNK2 multimerization (Fig. 6G).

### T3-stimulated cell cycle checkpoint inhibition is mediated by Wip1

**Figure 7:**
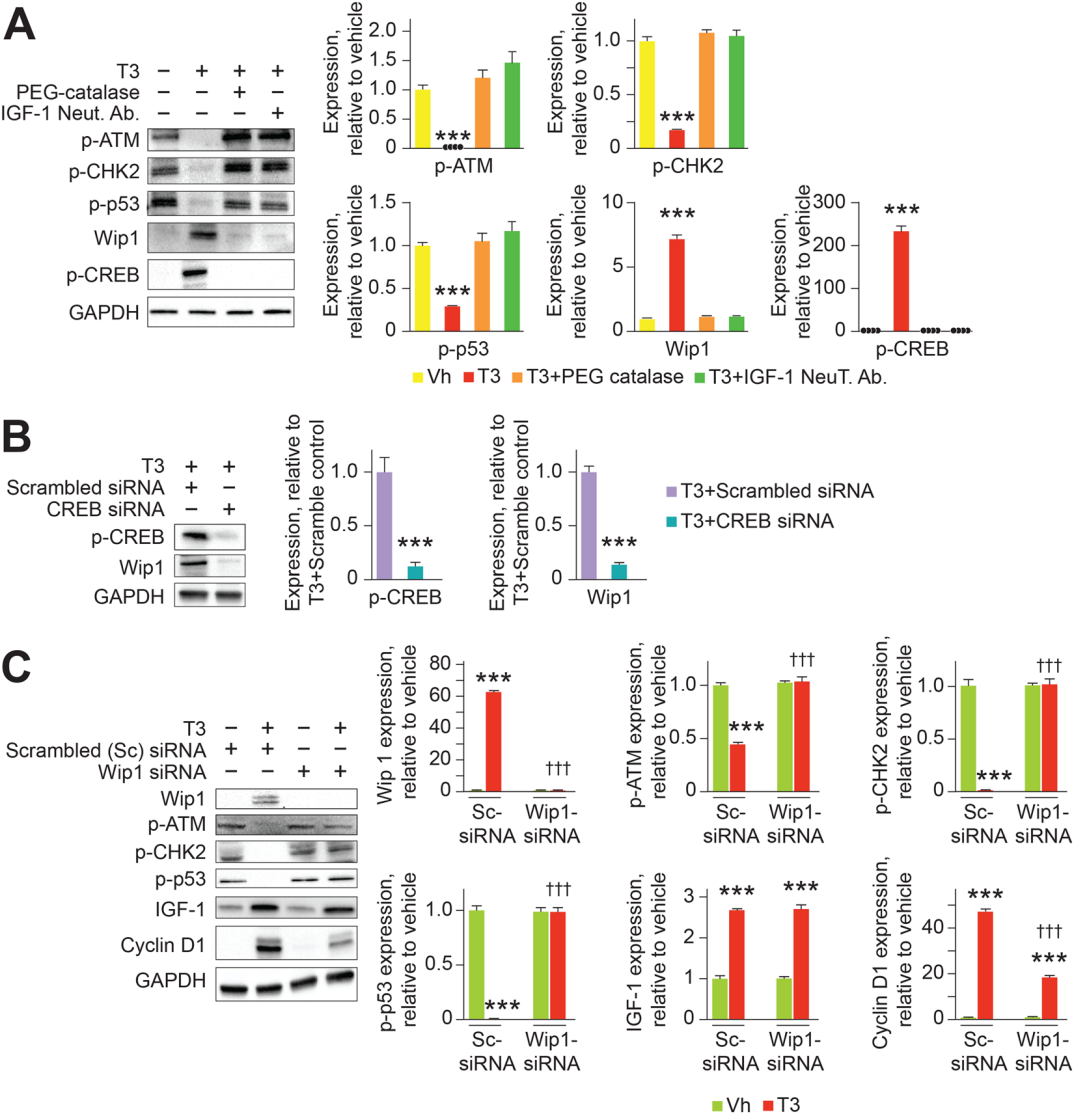
T3/mH_2_O_2_ signaling inhibits the activation of cell cycle checkpoint proteins by increasing CREB-mediated Wip1 expression. (**A**) Representative immunoblots of lysates from cultured neonatal cardiomyocytes, and their quantitative analyses, showing that T3 inhibits ATM, CHK2 and p53 phosphorylation, while increasing CREB phosphorylation and Wip1 phosphatase expression. H_2_O_2_ scavenging, by PEG-catalase, or IGF-1 inhibition, using a IGF-1 neutralizing antibody (IGF-1 Neut Ab), inhibited these effects of T3. ***P < 0.001, compared to vehicle controls. (**B**) Representative immunoblot, and their quantitative analyses, showing that knockdown of CREB using siRNA prevents T3-dependent Wip1 expression in cardiomyocytes. ***P < 0.001, compared to T3 + Scrambled siRNA controls. (**C**) Representative immunoblot, and their quantitative analyses, showing that knockdown of Wip1 using siRNA prevents T3 from inhibiting ATM, CHK2, and p53 phosphorylation, but not stimulation of IGF-1 expression. ***P < 0.001, comparing T3-treated groups to vehicle-treated groups for cardiomyocytes pretreated with scrambled siRNA or Wip1 siRNA. ^†††^*P* < 0.001, comparing the effects of Scrambled (Sc) siRNA or Wip1 siRNA pretreatment on cardiomyocytes exposed to either vehicle or T3 treatment. In (**A**–**C**), immunoblots are representative of four biological replicates. Error bars indicate SEM. n = 4/group.

It has been proposed that mH_2_O_2_ activates the DDR in neonatal murine cardiomyocytes^9^. In this situation, checkpoint arrest results from activation, by phosphorylation, of the ATM-driven DDR pathway components: ATM (S1981), CHK2 (T68) and p53 (S15). We found that, despite increasing mH_2_O_2_ generation (Fig. 1A), T3 inhibited ATM, CHK2 and p53 phosphorylation (Fig. 7A). Wip1 phosphatase relieves checkpoint arrest by de-phosphorylating these DDR pathway components^11^. Given that Wip1 expression is regulated by CREB^24^, which is in turn regulated by IGF-1^25^, we asked if T3 induces Wip1 expression by increasing IGF-1 formation. We found that T3-dependent formation of IGF-1 and CREB phosphorylation increased Wip1 expression in cardiomyocytes (Fig. 7A,B), and that Wip1 is necessary for checkpoint inactivation by T3 (Fig. 7C). Importantly also, without influencing IGF-1 formation, Wip1 depletion by siRNA decreased T3-stimulated cyclin D1 expression in cardiomyocytes (Fig. 7C). Additionally, siRNA-mediated knockdown of JNK2 confirmed its involvement in mediating T3-dependent Wip1 expression (T3 + Scrambled siRNA: 1 ± 0.18 versus T3 + JNK2 siRNA: 0.037 ± 0.004, *P* = 0.002). JNK2 depletion also prevented T3-stimulated, and Wip1 mediated, dephosphorylation of p-p53 resulting in a 5-fold increase in p-p53 (T3 + Scrambled siRNA: 1 ± 0.05 versus T3 + JNK2 siRNA: 5.20 ± 0.78, *P* = 0.002).

**Figure 8:**
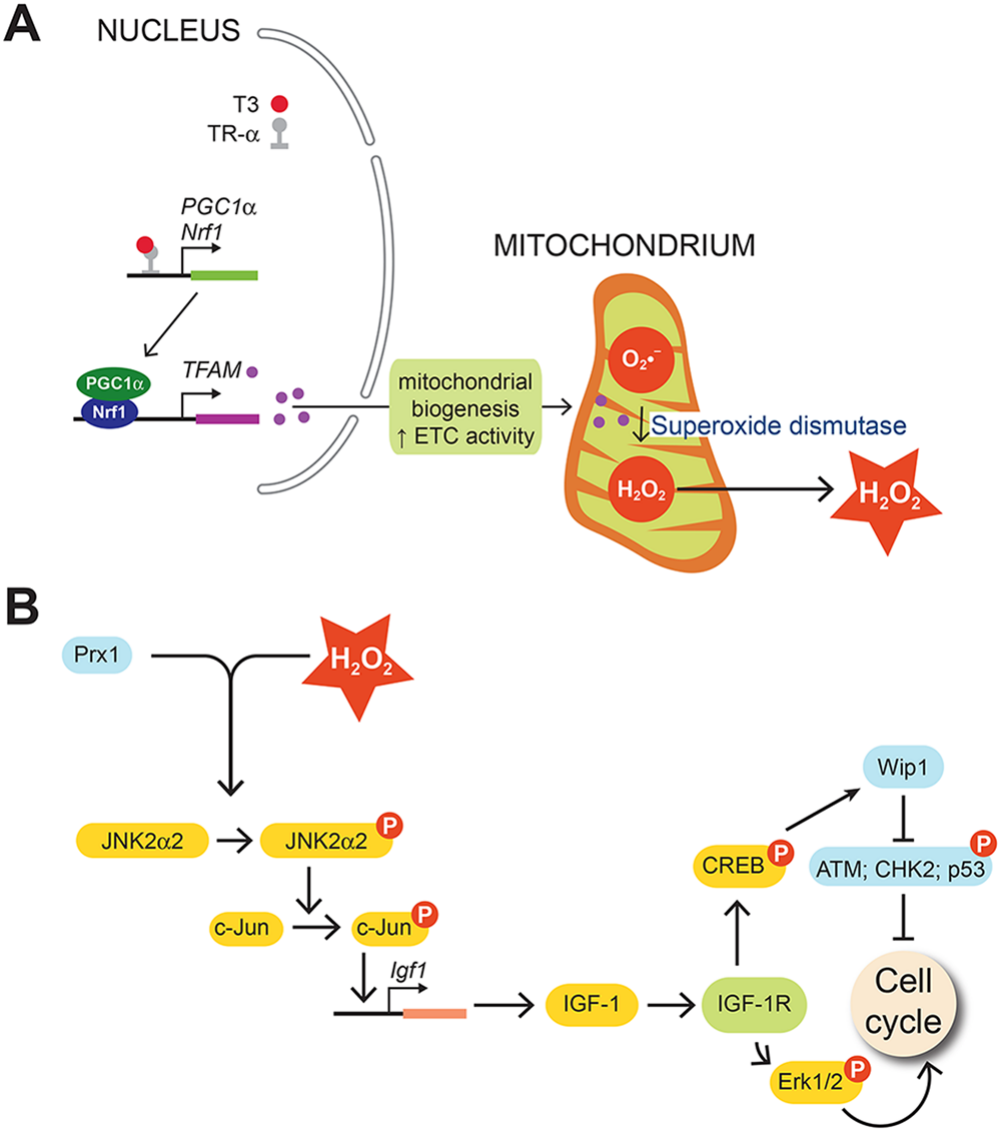
A model for the mechanism underlying T3-stimulated mH_2_O_2_ accumulation and its role in proliferative signaling. (**A**) T3 liganded with nuclear hormone receptor TRα transcribes *PGC1α* and *Nrf1*. Co-activation of PGC1α/Nrf1 generates TFAM that is translocated from the nucleus-to-mitochondria. TFAM increases mitochondrial biogenesis resulting in increased levels of superoxide (O_2_.^−^), that is converted into H_2_O_2_ by superoxide dismutase. (**B**) mH_2_O_2_-dependent IGF-1 formation links T3-actions on mitochondria to pathways that activate proliferation and inactivate cell cycle checkpoints. T3-stimulated mH_2_O_2_ activates JNK2α2 through a redox-sensitive mechanism involving the H_2_O_2_ sensor Prx-1. JNK2α2 activates c-Jun, which increases *Igf1* transcription and IGF-1 formation. IGF-1 signaling activates Erk1/2-dependent proliferative signaling and it inhibits the tumor suppressor p53 through CREB/Wip1 phosphatase signaling. Red circle with a “P” indicates the phosphorylated state of the protein.

Taken together, our data show that T3/mH_2_O_2_-mediated redox activation of JNK2α2 in neonatal cardiomyocytes initiates signaling cascades that not only stimulate cardiomyocyte proliferation, but also inactivate cell cycle checkpoints (Fig. 8A,B).

### mH_2_O_2_ is required for cardiomyocyte number increase during preadolescence

**Figure 9:**
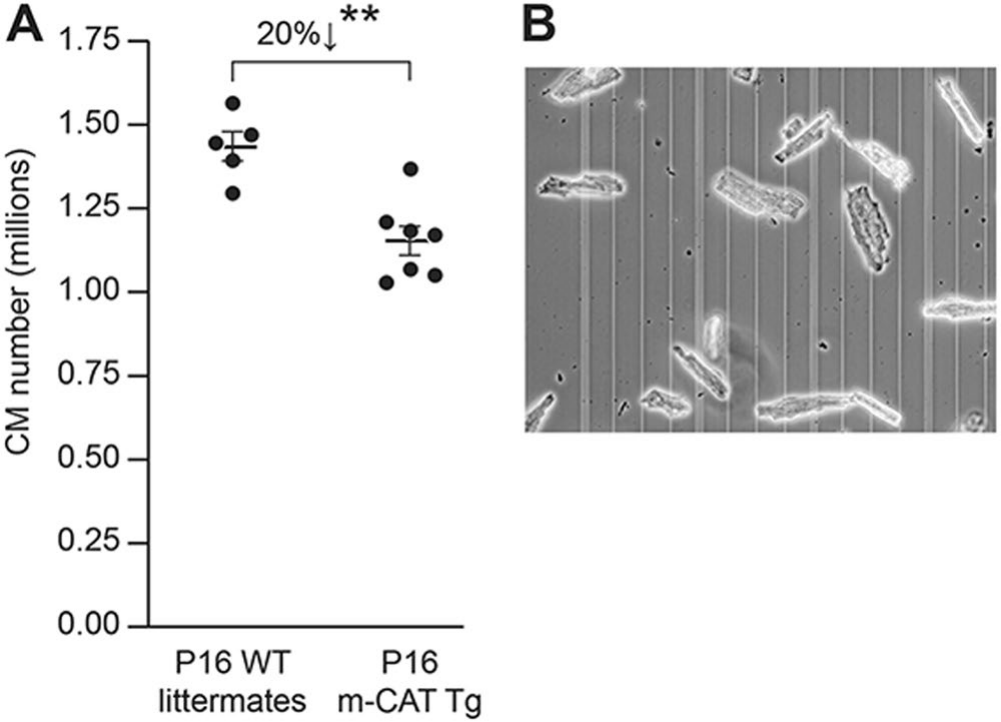
mH_2_O_2_ is required for developmental cardiomyocyte number expansion during preadolescence. (**A**) mH_2_O_2_ scavenging, using transgenic expression of catalase to mitochondria (m-CAT-Tg mice), reduced the number of ventricular cardiomyocytes in preadolescent P16 mice compared to WT littermate controls. **P < 0.01. Error bars indicate SEM. (**B**) A phase-contrast image of cardiomyocytes from a WT P16 disaggregated heart in a hemocytometer field. The image shows that cardiomyocytes can be readily identified because of their size and rod shape.

Circulating T3 levels increase over 6-fold between P10 and P12^2^. This surge increases T3 levels in the circulation to about 3 nmol/L and is linked to developmental cardiomyocyte replication during early preadolescence, well after the neonatal period^2^. We studied if cardiomyocyte replication during preadolescence is dependent on mH_2_O_2_ signaling. We found that cardiomyocyte numbers, although similar between m-CAT-Tg mice and their WT littermates at the end of neonatal period, that is at P7 (Fig. 3), were 20% lower (*P* < 0.01) in early preadolescence, that is at P16, in m-CAT-Tg mice (Fig. 9A). We next addressed if these differences in cardiomyocyte numbers, determined by hemocytometer counting, as above (see example in Fig. 9B), between m-CAT-Tg mice and their WT littermates could be explained by differences in digestion efficiencies. However, we found that at P16, digestion efficiencies were ~97% for both mouse groups (97.2 ± 0.59% and 97.5 ± 1.17% in m-CAT-Tg and WT hearts, respectively; *P* = 0.81). These data indicate that redox biology regulates cardiomyocyte proliferation during preadolescence.

## Discussion

We show here that T3 stimulates mH_2_O_2_ generation in neonatal cardiomyocytes, which activates JNK2α2-mediated IGF-1 formation. Redox activation of JNK2α2 acts as a nexus to connect T3 actions on the mitochondria to IGF-1-dependent proliferative ERK1/2 signaling. In addition, we show that IGF-1 signaling increases levels of Wip1, an oncogenic phosphatase that reverses G_1_/S and G_2_/M cell cycle checkpoints^11^. T3 administration in neonates promotes S phase and mitosis in cardiomyocytes, increases the number of mononuclear cardiomyocytes without increasing ploidy.

Mitochondria-generated ROS were once thought to exclusively cause cellular damage. Puente *et al*.^9^ proposed that increases in mH_2_O_2_ in neonatal hearts, associated with mitochondrial biogenesis, inhibit cardiomyocyte proliferation. Hirose *et al*.^8^ identified T3 signaling as an upstream regulator of this pathway. However, recent evidence suggests that mitochondrial ROS are critical for healthy cell function^26–29^. We found that exogenous T3 administration starting at P2 increased the cardiomyocyte endowment of neonatal hearts. Three lines of evidence underpin the involvement of mH_2_O_2_ in this T3 action. First, we show that T3 increases H_2_O_2_ secretion from neonatal cardiomyocytes *in vitro* by about 0.2 µmol/L; an effect inhibited by mitoTEMPO, a mitochondria-targeted antioxidant that inhibits H_2_O_2_ generation by mitochondria^30^. Although H_2_O_2_ is freely diffusible, the presence of antioxidants creates a peroxide gradient across the cell. By building a compartmental model that estimates this gradient using experimentally determined kinetics of H_2_O_2_ adsorption rates across the membrane, Antunes *et al*.^31^ predicted a gradient of ~10-fold. This means that the ~0.2 µmol/L increase in H_2_O_2_ secretion from cardiomyocytes, caused by a 1-hour T3-treatment, represents an increase in levels of cytosolic H_2_O_2_ that is about 10-fold higher. Second, in support of the involvement of mitochondria in the T3 proliferative response, we also show that T3-stimulated proliferative signaling in neonatal cardiomyocytes *in vitro* can be inhibited by siRNA-induced knockdown of NRF1; PGC1α and NRF1 together transcriptionally increase TFAM, which regulates mitochondrial biogenesis and oxidative phosphorylation^13^. Third, we show that, *in vivo*, T3 stimulation of cardiomyocyte proliferation in neonates is blocked by mitochondria-targeted scavenging of H_2_O_2_ using catalase overexpression. These findings indicate that exogenous T3 administration stimulates H_2_O_2_ generation primarily in mitochondria, and that generation of this ROS is required for T3-stimulated cardiomyocyte proliferation.

Nevertheless, these findings raise the question: how do low levels of mH_2_O_2_ find their target substrate given its high reactivity for cellular antioxidants^21^ and low reactivity for the cysteines in most proteins? The mechanism we describe is akin to that reported for STAT3 inhibition by a redox relay in which the thiol peroxidase, Prx2, is oxidized by exogenous H_2_O_2_ and passes its oxidation onto STAT3^23^. However, our findings differ in important ways from that of STAT3. We show that the source of the H_2_O_2_ is mitochondria, which is secondary to T3 stimulating mitochondrial biogenesis and increasing H_2_O_2_ formation from this subcellular organelle. We also show that Prx1 acts as a H_2_O_2_ signal receptor and transmitter in MAPK redox regulation. Prx1 forms a covalent multimer with JNK2α2 within 60 minutes of exposure of neonatal cardiomyocytes to T3, in a process that consumes cellular Prx1 and JNK2α monomers and requires mH_2_O_2_. In the absence of *Jnk2*, T3 is unable to mount a proliferative response in neonatal cardiomyocytes. Species and development stage-specific differences in the transcriptional regulation of *Jnk2*, and alternative splicing of its precursor mRNA that gives rise to JNK2 isoforms α1, α2, β1, β2, could provide a cellular context to the unusual T3 proliferative response in neonatal murine cardiomyocytes; a response that is not seen in cardiomyocytes derived from human embryonic stem cells^32^, late-term fetal sheep^33^ or zebrafish^8^.

While exogenous T3 stimulates mH_2_O_2_/JNK2α2-mediated cardiomyocyte proliferation in neonatal hearts, the proliferation of cardiomyocytes observed developmentally in neonates is independent of the early, small initial increase in endogenous T3 that occurs at P4. We base this conclusion on the finding that neither *Jnk2* deletion (Fig. 5D) nor targeted overexpression of catalase in mitochondria (Fig. 3) influence the developmental increase in cardiomyocyte numbers during the neonatal period before P7.

Hirose *et al*.^8^ showed that deleting TRα or inhibiting endogenous T3 biosynthesis with PTU from before birth onwards, increased cardiomyocyte endowment at P14. However, immediately after the neonatal period, starting at around P10, circulating T3 increases rapidly to levels seen in adults^2^. This hormonal increase is associated with an increase in cardiomyocyte numbers, and short-term PTU treatment inhibits *in vivo* cardiomyocyte proliferation in post-neonatal hearts^2^. The reasons for these differing findings may lie in the nature of experiments used. For example, TRα deletion can activate numerous pathways that are normally repressed by the TRα-aporeceptor^34–36^ and long-term PTU treatment has profound effects on organ growth^37,38^. In this regard, our studies with m-CAT-Tg mice may better address the issue, because in our experiments we selectively interfere with mH_2_O_2_ signaling in cardiomyocytes without impacting core genomic effects of the TRα-aporeceptor, or of the T3-liganded TRα. We show here that the increase in cardiomyocyte numbers during early preadolescence is also inhibited by *in vivo* mH_2_O_2_ scavenging, consistent with the proposed mechanism of T3-stimulated cardiomyocyte proliferation.

A reason why the 4-fold rise in endogenous T3 at P4 does not affect cardiomyocyte proliferation might be that, despite this T3 increase, circulatory T3 concentrations in neonates remain below ∼20 pmol/L^8^; at these concentrations, which are ∼100-fold lower than in adults^2^, TR occupancy by T3 is expected to be exceedingly low^39^. By contrast, soon after the neonatal period, at ~P11/P12, circulating T3 levels increase to ∼3 nmol/L^2^; a surge in T3 levels that precedes an increase in cardiomyocyte numbers^2^. As discussed above, we show that the developmental increase in cardiomyocyte numbers during preadolescence is inhibited by mH_2_O_2_ scavenging, which supports a role for redox biology in regulating developmental stage-specific T3-mediated cardiomyocyte proliferation.

In previous studies into the mechanisms of cardiomyocyte cell cycle block and its reversal by mitogenic stimulation, cell proliferation was established by showing increases in ventricular cardiomyocyte numbers. In these studies, cardiomyocyte numbers were either determined by dividing the myocyte fraction of ventricular volume by the average myocyte volume^40^ or by direct counting of cardiomyocytes after enzymatic disaggregation of hearts^2,8^. The cardiomyocyte-to-ventricular volume ratio method is an indirect measure and is influenced by cardiomyocyte shrinkage resulting from cell/tissue fixation. The direct counting method has been used more widely. The Sadek^9,41,42^, Tzahor^43^, and Huang^8^ laboratories, for example, have all used direct counting to demonstrate cardiomyocyte replication. However, a key criticism of the cell-counting method has been the impact of digestion/disaggregation efficiencies between laboratories, and between animal treatment groups. Thus, we optimized our digestion protocols to rapidly obtain single cell suspensions from the cardiac ventricles with digestion efficiencies of ~97% in hearts of mice of all ages from P1 to P16. Upon microscopic examination, the residual tissue was almost entirely undigested cardiac valves and blood vessels; consistent with this, over-digestion with collagenase did not increase cardiomyocyte yields.

The integration of mitochondrial retrograde signaling, from mitochondria to the nucleus, into hormonal activation of cellular proliferation—through *Igf1* transcription—is distinct from previous examples of retrograde signaling, which are considered adaptive because they convey information about alterations in mitochondrial metabolic and respiratory states, or mitochondrial genetic instability^44^. The absolute reliance on a relatively rare JNK isoform as a mediator of proliferation implies that this type of mitochondrial signaling is critical for, but restricted to, the regulation of discrete physiological responses.

Here we have defined an entirely novel biochemical pathway whereby T3 acts as a potent mitogen for expanding cardiomyocyte endowment during preadolescence. A prospective randomized clinical study suggests a therapeutic benefit of T3 in the postoperative period in newborn patients undergoing operations for complex congenital heart disease^45^. Continued evaluations of T3 as a pro-regenerative therapy that allows structural and functional repair of the injured juvenile heart is warranted.

## Materials and Methods

A detailed description of the experimental procedures related to immunoblotting, immunoprecipitation, evaluation of multimeric protein complexes, RT-qPCR, H_2_O_2_ measurement and immunofluorescence is provided in the Supplementary Information section.

### Animal husbandry and mouse models

All animal studies were approved by the Institutional Animal Care and Use Committee (IACUC) of Emory University. We confirm that all experiments were performed in accordance with IACUC guidelines and regulations. all methods were performed in accordance with the relevant guidelines and regulations. Adult C57BL/6 mice were used for breeding. The mothers were between 3–8 months old. All mice were allowed free access to food and water. An animal husbandry protocol was developed to minimize variation between litters and between studies. Factors considered during the critical period of post-neonatal development studied, were as follows: (1) age of the mother—dams <36 weeks were used; (2) animal chow—50/50 mix of Purina Lab Diets Rodent diet 5001 (standard diet) and 5015 (breeder diet); 3) minimization of stress on the dams, especially between the first 2 days after delivery and weaning; (4) standardization of litter sizes—only pups from litter sizes of 6–8 (counted at P2) were used; (5) for studies on preadolescent mice, litter sizes from these births were further adjusted to 4 pups per dam at P2 to minimize inter-experiment variation in growth rates; (6) to minimize the effect of intra-litter variation, drugs were usually given to about half of the pups in a litter and vehicle to the rest; and 7) separating plugged/pregnant dams into individual cages. Drugs or vehicle controls were administered randomly to pups within each litter by intraperitoneal injection. C57BL/6 wild type (Jackson Laboratory, 000664), m-CAT-Tg (Jackson Laboratory, 016197) and *Jnk2*^−/−^ (Jackson Laboratory, 004321) mice were used in these studies. Both, *Jnk2*^−/−^ and m-CAT-Tg mice are on C57BL/6 background. Only progeny born from breeding heterozygous m-CAT-Tg sires with wild type C57BL/6 dams were used to generate heterozygous m-CAT-Tg transgenic mice and WT littermate controls. For *in vivo* studies, T3 (Sigma-Aldrich, T6397-100MG) or vehicle (phosphate buffered saline, PBS) were administered intraperitoneally to each mouse as indicated in each Figure.

### Primary cardiomyocyte culture

Medium, HEPES buffer and PBS was pre-warmed to 37 °C. After euthanasia of P1–P3 pups by decapitation (without the use of CO_2_ or any other chemicals that could affect cardiomyocyte responses to T3), hearts were harvested and immediately placed into fresh ice-cold calcium-and magnesium-free PBS in a 10 cm dish. Extra cardiac tissue and atria were carefully removed, and 50 intact pooled ventricles were transferred to the C-tube of the gentleMACs Dissociator (Miltenyi Biotec). Digestion buffer (7.5 ml, 0.4 mg/ml Collagenase type 2; volumes were scaled up when working with a greater number of hearts) was added, and the hearts incubated at 37 °C for 15 min and then the C-tube was inserted into the gentleMACS Dissociator for dissociation according to program m_neoheart_0.1.01. The supernatant fraction was then aspirated and gently transferred into a fresh 50 ml Falcon tube. Digestion was stopped by adding 5 ml of Stop Buffer containing 10% fetal bovine serum (FBS; Atlanta Biologicals, S11110H, triple 0.1 µm filtered, heat-inactivated). If required, the digestion was repeated up to three times until most of the tissue was digested. To repeat the digestion, 2.5 ml digestion buffer was added, and the mixture incubated for 10 min for each repeat. Pooled cells were transferred into a fresh 15-ml tube, which was filled with Stop Buffer and then centrifuged at 340 × g for 5 min. The supernatant fraction was aspirated and discarded, and the pellet washed with 10 ml of culture medium (DMEM/F12, Invitrogen, 11330-032) supplemented with 10% FBS containing penicillin-streptomycin (1 in 50 dilution) and glutamine (1 in 50 dilution). The pellet was re-suspended in 10 ml of culture medium and transferred to a sterile 100-µm pluriStrainer (pluriSelect, 43-50100-03) to remove aggregates and connective tissue. The strainer was washed once with 5 ml of culture medium. The pass-through fraction not retained by the 100 µm-filter was transferred to a sterile 20 µm-pluriStrainer (pluriSelect, 43-50020-03) and the resulting retentate saved; the pass through fraction mainly contained non-myocytes. The fraction retained by the 20 µm-filter was enriched in cardiomyocytes, which were harvested by inverting the pluriStrainer, and then transferred them to a fresh 50 ml tube. The strainer was washed twice with 10 ml of culture medium or until most of the cells on the strainer are washed off into the tube, and the cells pelleted at 300 × g for 5 min. After removal of the supernatant fraction, the cell pellet was re-suspended in 10 ml of culture medium. To further purify the cardiomyocytes, we removed leftover non-myocytes by plating these cells for 2 h at 37 °C on a non-collagen coated tissue culture plate. This time period allows cardiac fibroblasts to attach to the plate, whereas cardiomyocytes remain suspended. After incubation, the non-adherent cells in the solution (predominantly cardiomyocytes) were transferred into a 15 ml-tube, and pelleted at 300 × g for 5 min. The supernatant fraction was discarded, 5 ml Dulbecco’s modified Eagle’s medium (DMEM/F12, Invitrogen, 11330-032) containing 10% FBS was added to the pellet and the cardiomyocytes re-suspended by multiple gentle passes through a wide nozzle transfer pipette. To evaluate the purity of isolated cardiomyocytes, expression of αMHC, a cardiomyocyte specific marker, and vimentin, a non-myocyte marker, were determined by immunoblotting. These studies confirmed that our isolated cardiomyocytes were virtually devoid of non-myocytes (Supplementary Fig. S4). Additionally, the quality of these cardiomyocytes was also established by showing that they mediated canonical responses to T3 signaling, both at the mRNA and protein level (Supplementary Tables S1, S2 and S4).

Collagen type I coating reagent (Sigma, C3867) was added to 12-well cell culture plates for 2 h at 37 °C. After this time, remaining reagent was aspirated, and the wells washed twice with 0.5 ml PBS. After aspirating the PBS, the plates were air dried. 1 ml of cell suspension (containing about 1.5 × 10^6^ cardiomyocytes) was then added to each pre-coated well, and the cardiomyocytes incubated for 12 h at 37 °C in a 5% CO_2_ humidified incubator (Hera cell 240, Thermo Scientific). The culture medium was aspirated, pre-warmed serum-free medium (1 ml of fresh Dulbecco’s modified Eagle’s medium (DMEM/F12, Invitrogen, 11330-032) supplemented with penicillin-streptomycin (1 in 50 dilution) and glutamine (1 in 50 dilution) added, and the cardiomyocytes incubated at 37 °C for 1 h. After washing twice with pre-warmed serum-free medium, the cardiomyocytes were incubated at 37 °C for 8 h in serum-free modified DMEM/F12 (as above) with vehicle or T3 (10 nmol/L, unless indicated otherwise), either alone or in combination with ERK1/2 inhibitor (20 µmol/L, Sigma, PD98059), IGF-1 antibody solution (1 µg ml–1, Abcam, ab9572) or PEG-catalase (200 U/ml, Sigma, C4963-2MG). In some experiments, H_2_O_2_ (0.15, 1.5 or 15 µmol/L, Sigma, 88597-100ML-F) was added to the culture medium instead of T3. In some experiments, culture medium was then collected for determination of H_2_O_2_ using Amplex Red assays (Life Technologies, A22188). Cardiomyocytes were washed twice with 500 µl of ice-cold PBS. For RNA isolation, the cardiomyocytes were then suspended in 240 µl of RNAlater stabilization solution (22 °C) (Ambion, AM7020), and then snap-frozen in liquid nitrogen. For protein determination, cardiomyocytes were suspended in 250 µl of RIPA buffer (Cell Signaling, 9806S) supplemented with phosphatase inhibitor cocktail 2 and 3 (Sigma-Aldrich, P5726-1ML and P0044-1ML), 0.1 mmol/L phenylmethylsulfonyl fluoride (PMSF, Sigma-Aldrich, 93482-50ML-F) and protease inhibitor cocktail (Roche, 11697498001); cardiomyocytes were then lysed by sonication and centrifuged at 21,000 × g for 30 min. The resulting supernatant fractions were aliquoted into fresh Eppendorf tube and then snap-frozen in liquid nitrogen.

For siRNA experiments, after 12 h of incubation in DMEM/F12 with 10% FBS (as above), cardiomyocytes were washed once with 2 ml of siRNA transfection medium (Santa Cruz, sc-36868) and then 1 ml of target gene siRNA transfection reagent mixture was added to cardiomyocytes. siRNA transfection reagent mixture was prepared according to the manufacturers guidelines (Santa Cruz). Cardiomyocytes were incubated with the siRNA containing transfection mixture for 2.5 h at 37 °C in a 5% CO_2_ incubator to ensure siRNA uptake by the cardiomyocytes. The transfection mixture was then replaced with 1 ml of pre-warmed serum-free medium Dulbecco’s modified Eagle’s medium containing T3 (10 nmol/L). We then incubated the cardiomyocytes for an additional 8 h before collection with RIPA buffer for protein analysis. Scrambled siRNA was used as a control.

### Determination of cardiomyocyte numbers, nucleation and ploidy

Heparin (50–100 µl, 1000 USP units/ml) was injected intraperitoneally 8 minutes prior to harvesting. Hearts were removed with their atria and aorta intact, washed with PBS and then the aorta cannulated for retrograde perfusion through the coronary circulation. Hearts were immediately perfused with cytofix (BD Biosciences, 554655) for 1 min. Subsequently, hearts were perfused with perfusion buffer (120 mM NaCl, 15 mM KCl, 0.5 mM KH_2_PO_4_, 5 mM NaHCO_3_, 10 mM HEPES, and 5 mM glucose, at pH 7.0) for 2 min and then with perfusion buffer containing collagenase type 2 (Worthington, LS004176) for 8–15 min at 37 °C. Perfusion and digestion buffers were freshly prepared, warmed to 37 °C and aerated with 5% CO_2_. Collagenase concentration was 1 mg/ml for P2 to P7 hearts and 2 mg/ml for P16 or older hearts. After 8–15 min of digestion, the atria were excised, and the cardiac ventricles were placed in a 6 cm dish containing 1 ml of digestion buffer; we then added ~3 ml of STOP buffer (perfusion buffer plus 10% bovine calf serum and 12.5 mM CaCl_2_). The ventricles were teased apart into smaller sized pieces using forceps followed by trituration through a small-aperture pipette. The digested cardiomyocytes from each heart were collected in a 15 ml falcon tube and more STOP buffer was added to a volume of 10 ml. The final cell suspension was used to count cardiomyocytes using a hemocytometer. To avoid losses, cardiomyocytes were not purified, but could be readily identified by phase contrast microscopy based on their cytoplasmic size and rod shape. Multiple aliquots were counted per heart and the mean value was used to determine the total number of ventricular cardiomyocytes/heart.

For accurate cardiomyocyte number determination, a critical step is optimal digestion efficiency. This variable is chiefly dependent on collagenase concentration in the perfusion medium, exposure time and temperature. To optimize digestion efficiencies, these variables were adjusted depending on the age and weight of the mouse. Digestion efficiency was calculated [ventricular weight %, determined by (original weight – residual)/original weight] after each change in condition. We found that maximal digestion efficiencies were between ∼ 97% and 99%. At P7, digestion efficiencies were ~97% (97.0 ± 1.1% and 97.1 ± 1.4% in vehicle- and T3-treated hearts, respectively; *P* = 0.94; *n* = 4). At P16, digestion efficiencies were also ~97% (97.2 ± 0.59% and 97.5 ± 1.17% in m-CAT-Tg and WT hearts, respectively; *P* = 0.81; *n* = 4). Upon microscopic examination, the residual tissue was almost entirely undigested cardiac valves and blood vessels. Over-digestion neither improved digestion efficiencies, nor did it increase cardiomyocyte yield. We did not estimate cardiomyocyte numbers from under-digested hearts in which disaggregation of myocardial tissue was incomplete. Suboptimal cannulation of the aorta was the cause of under-digestion, but it was infrequent.

For cardiomyocyte nucleation analysis, after purification, cardiomyocytes were stained with 6-diamidino-2-phenylindole (DAPI) for nucleation counts, which were plotted as percentage of counted cardiomyocytes^2^. Ploidy analysis on purified cardiomyocytes was performed using DAPI and cardiac troponin T as described by Hirose *et al*.^8^.

### Statistical analysis

Statistical significance was assessed by data analyses using Grahpad Prism 8. Shapiro-Wilk test was used to determine if the data were normally distributed, in which case we used one-way ANOVA followed by Tukey for multiple comparisons, and unpaired two-tailed Student’s t-test for comparison involving 2 groups. The F-test was used for estimation of variance when comparing two groups and the Brown-Forsythe test was used for estimation of variance when multiple groups were compared using one-way ANOVA. Where the data were not normally distributed, we used non-parametric tests: the Kruskal-Wallis test followed by Dunn’s multiple comparison test, or the Mann-Whitney test for comparison of 2 groups. Differences at *P* < 0.05 were considered significant.

## Supplementary information

Supplementary info

## Data Availability

The data and resources generated for this manuscript are available upon reasonable request from the corresponding authors.
