# Supplementary material for: Redox activation of JNK2α2 mediates thyroid hormone-stimulated proliferation of neonatal murine cardiomyocytes
Source: Sci Rep. 2019 Nov 27;9:17731. doi: 10.1038/s41598-019-53705-1 (PMC6881338; doi:10.1038/s41598-019-53705-1)
Supplement: Supplementary file 1 — Supplementary info [file 41598_2019_53705_MOESM1_ESM.pdf]

Redox activation of JNK2α2 mediates thyroid hormone-stimulated proliferation of neonatal murine cardiomyocytes

Lin Tan[1,†], Nikolay Bogush[1,†], Hussain Naib[1], Jennifer Perry[2], John W. Calvert[3], David I.K. Martin[4], Robert M. Graham[5], Nawazish Naqvi[1,*], Ahsan Husain[1,*]

[1]Department of Medicine (Cardiology), Emory University School of Medicine, Atlanta, Georgia, USA; [2]Department of Animal Resources, Emory University School of Medicine, Atlanta, Georgia, USA; [3]Department of Surgery, Carlyle Fraser Heart Center, Emory University School of Medicine, Atlanta, Georgia, USA; [4]Children's Hospital Oakland Research Institute, Oakland, California, USA; [5]Victor Chang Cardiac Research Institute, Darlinghurst, New South Wales, Australia.

[†]These authors contributed equally to this work.

[*]Corresponding Authors: Ahsan Husain, PhD, Department of Medicine (Cardiology), Emory University, 3311 WMRB, 101 Woodruff Circle, Atlanta Georgia 30322. Tel: 404-727-8125; E-mail ahusai2@emory.edu or Nawazish Naqvi, PhD, Department of Medicine (Cardiology), Emory University, 323 WMRB, 101 Woodruff Circle, Atlanta Georgia 30322. E-mail nnaqvi@emory.edu

Short title: T3-stimulated cardiomyocyte proliferation is mediated by mitochondria-generated H_2O_2.

Key words: Thyroid hormone, cardiomyocyte proliferation, cell cycle, mitochondrial H_2O_2, JNK2α2, and IGF-1.

Word Count: Abstract 184; Main Text 3869; Methods 2045

Supplementary Section includes Supplementary Methods, five Supplementary Tables and four Supplementary Figures and a separate pdf file with all the uncut immunoblotting gels.

Supplementary Methods

Immunoblotting

Whole cell cardiomyocyte lysates were generated, aliquoted and stored as detailed in the cell culture protocol above. Aliquots were removed from –80 °C freezer and allowed to thaw on ice. Initially, 5 to 10 µl of each sample (~20 µg protein) was mixed with an equal volume of 2x Laemmli sample buffer (Bio-Rad, 1610737) and heated for 5 min at 95–99 °C and then immediately cooled on ice. For protein complexes to be analyzed under native/non-denaturing conditions we used Native sample buffer (Bio-Rad, 1610738) instead of the Laemmli buffer. After cooling on ice for 5 min we centrifuged tubes briefly and then we loaded the samples onto a SDS–polyacrylamide gel (12–18%) for electrophoresis (SDS-PAGE) at 200 volts for 5 min, then at 150 volts for 30 min to 2 h, and then transferred to a PVDF membrane by electroblotting (Turbo Transfer; Bio-Rad). Depending upon the molecular weight of the proteins or protein complexes, the transfer time on Turbo Transfer was varied for high (11 min), average (7 min) and low (5 min) molecular weight proteins. After transfer, all blots were pre-blocked from 30–60 min in Superblock (Thermo Scientific, 37536). Initially, the samples were probed with GAPDH antibody. Based on GAPDH, the loading for each sample was adjusted to run gels containing all samples with an equal amount of GAPDH. Membranes were probed with the target protein specific primary antibody. For quantitative analysis, the membrane was then stripped and re-probed with GAPDH to normalize loading for each sample. For stripping, the membrane was washed twice with 1x Tris-buffered saline (TBS, Thermo Scientific, BP2471-1) 5 min each and then incubated with Restore™ Western Blot Stripping Buffer (Thermo Scientific, 21059) for 5–15 min and then washed again twice with 1x TBS and pre-blocked with Superblock (Thermo Scientific, 37536) for 1 h before incubating with GAPDH antibody. Primary antibodies (see

below) were also diluted in Superblock and incubated for 2 h at 22 °C, or overnight at 4 °C, followed by horseradish peroxidase (HRP)-labeled secondary antibody (1:10,000) for 45 min at 22 °C. The signals were detected using Super Signal West Dura Detection Reagent (Thermo Scientific, 34075) and images captured on a Bio-Rad GelDoc system equipped with CCD camera and ImageLab program (Bio-Rad). Quantification was performed by densitometry using the ImageLab program.

The antibodies used for immunoblots, immunoprecipitation, or both were: c-Jun (Cell Signaling, 9165P); cyclin D1 (Abcam, ab134175); cyclin A2 (Abcam, ab181591); cyclin B1 (Abcam, ab32053); ERK1/2 (Cell Signaling, 4695); γH2AX (GeneTex, GTX80694); IGF-1 (Abcam, ab9572); JNK(1/2/3) (Abcam, ab179461); JNK2 (Cell Signaling, 9258); JNK2α (Abcam, ab134567); JNK2β (Abcam, ab133158); Nrf1 (Abcam, ab175932); phospho-ATM (S1981) (Abcam, ab36810); phospho-c-Jun (S73) (Cell Signaling, 3270); phospho-CHK2 (T68) (Abcam, ab183895); phospho-CREB (S133) (Cell Signaling, 9198); phospho-ERK1/2 (T202/Y204) (Cell Signaling, 4370); phospho-JNK(1/2/3) (T183/Y185) (Cell Signaling, 9255); phospho-MKK4 (S257) (Cell Signaling, 4514); phospho-MKK7 (S271/T275) (Cell Signaling, 4171); phospho-p53 (S15) (Cell Signaling, 12571); Prx1 (Cell Signaling, 8499); Prx2 (Abcam, ab109367); TFAM (Sigma, SAB1401383); TRα (Thermo Scientific, PA1-211A); TRβ (Thermo Scientific, MA1-216); and Wip1 (Cell Signaling, 11901S). Most of these antibodies are profiled in 1DegreeBio and were also validated using siRNA.

Immunoprecipitation

Cardiomyocytes were collected post-T3 treatment and lysates were generated as detailed in the cell culture protocol (above). To immune-precipitate phosphorylated forms of JNK antigens from the cardiomyocyte lysates, we first incubated primary pan-phosphorylation-specific JNK

antibody (1-10 µg) with 50 µl (1.5 mg) immunoglobulin (antibody) binding Protein A Dynabeads (Life technologies, 10001D) for 20 min at room temperature for immobilizing the antibody to the solid Dynabead support. We then placed the tube containing the magnetic Dynabeads on a magnet. The magnetic Dynabeads attached to the inner wall of the tube touching the magnet. This allowed removal of supernatant without disturbing the beads containing the antibody. The supernatant was removed and 200 µl PBS containing 0.02% Tween-20 (PBST) added. The tube was detached from the magnet and the PBST solution was gently mixed with the beads. The tube was again placed on magnet and the supernatant containing unbound primary antibody liberated from the Dynabeads by mixing with PBST, was removed. The tube now contained antibody coupled magnetic beads. We then added whole cell protein lysate prepared earlier from T3 treated cardiomyocytes (typically 0.1–1 ml) for 1 to 2 h at room temperature to allow formation of antibody-antigen complexes. At the end of incubation, we washed 3x with 200 µl PBST using the magnet as described earlier. These washings helped purification of only the antibody-antigen bound complex from any unbound non-specific antigens. We then added 20 µl of 2x Laemmli sample buffer (Biorad, 1610737), and after mixing the beads, the sample was heated for 5 min at 95–99 °C and immediately cooled on ice. The tube was then placed on the magnet and the supernatant containing the immunoprecipitated antigen was aspirated and loaded on to an SDS-PAGE gel. Gel running conditions, transfer to PVDF membrane, probing with antibodies and detection were performed as described above.

Non-reducing/reducing SDS-PAGE for detection of multimeric protein complexes

Cardiomyocytes were treated with T3 (10 nmol/L) and collected at 0.5, 1, 2, 3, 4 and 8 h post-T3 treatment. To ensure that the endogenous oxidation and disulfide state of proteins was maintained, cardiomyocytes were immediately washed twice in 500 µl ice-cold PBS

supplemented with 10 mg/ml catalase (Sigma-Aldrich, C1345-1G) and 100 mmol/L N-ethylmaleimide (NEM, Sigma-Aldrich, E3876-5G). The solution was then aspirated, and the cardiomyocytes were harvested in 250 µl of RIPA buffer supplemented with 10 mg/ml catalase for 20 min on ice. Whole cell cardiomyocyte lysates were generated as detailed in the cell culture protocol above.

For detection of multimeric complexes, we ran the lysates under non-reducing conditions. We mixed equal volumes of each sample (~10 µl) with equal volume of Native Sample Buffer (Bio-Rad, 161-0738), vortexed briefly and centrifuged at 16,000 x g for 5 min. We collected the supernatant and loaded onto 4-15% Criterion™ TGX™ gel (Bio-Rad, 5671084). The gel was run in pre-chilled 1x running buffer (Tris-Glycine Buffer without SDS; Bio-Rad, 161-0734) and placed the gel tank in iced water. Electrophoresis was performed at 200 volts for 5 min, and then at 150 volts until the loading dye ran through the entire gel. Proteins were then transferred to PVDF membrane, probed with antibody and signal was detected as detailed above in the immuno-blotting protocol. To determine if JNK2/Prx1 multimeric complex are stabilized by disulfide bonds, we added DL-Dithiothreitol (DTT, Sigma-Aldrich, D0632-1G) at a final concentration of 350 mmol/L in the samples diluted in 2x Laemmli sample buffer (Bio-Rad, 161-0737); to reduce disulfide bonds. We heated the samples at 95–99 °C for 5 min and immediately cooled on ice. The samples were then vortexed briefly and centrifuged at 16,000 x g for 5 min. We collected the supernatant and loaded it onto 4-15% Criterion™ TGX™ gel (Bio-Rad, 5671084). We ran these DTT containing samples on 12% Criterion™ TGX™ gel (Bio-Rad, 5671044) with 1x denaturing conditions running buffer (Tris/Glycine/SDS Buffer–Bio-Rad, 161-0732). After electrophoresis, proteins were transferred to PVDF membrane, incubated with primary antibody and proteins of interest detected as described above.

RT-qPCR

Cardiomyocyte sample tubes containing RNAlater stabilization solution were removed from –80 °C freezer and thawed on ice. The samples were then centrifuged at 21,000 x g for 10 min. RNAlater supernatant was removed and replaced with 240 µl of lysis binding buffer from the mirVana miRNA Isolation Kit (ThermoFisher, AM1560). RNA was purified according to the manufacturer's guidelines. Purified RNA was reverse transcribed using Transcriptor Reverse Transcriptase (Roche, 03531295001) and random primer (Primer, random p(dN)6, Roche, 11034731001). Quantitative PCR was performed with SYBR Green Supermix (Bio-Rad, 1708882) on a iQ5 Thermal Cycler (Bio-Rad). Primers were synthetized by IDT Technologies; their sequences are presented in Supplementary Table S5.

Cardiomyocyte purification for immunoblotting and immunocytochemistry

For Western blotting and immunocytochemistry, hearts were enzymatically digested, as described above. Before making single cell suspensions, atria were excised, and cardiac cells were disaggregated into single cell suspension. Cardiomyocytes were purified with 3 low speed centrifugations (18 xg for 4 min at room temperature), which caused cardiomyocytes to settle as a pellet. Supernatant fractions, enriched in non-myocytes, were discarded. These cardiomyocyte preparations were > 95% pure (2). Cardiomyocytes were snap frozen in liquid nitrogen and stored at –80 °C for Western blotting. Additionally, cardiomyocytes were fixed with Cytofix (BD Biosciences, 554655) for 5 min and spread on glass slides for immunocytochemistry.

H2O2 measurement: After treating cardiomyocytes with T3 alone or in combination with other reagents tested, the culture medium was collected for H_2O_2 quantitation using the Amplex® Red Hydrogen Peroxide/Peroxidase Assay Kit (Thermo Scientific, A22188). Assays were performed immediately after media collection, according to manufacturer's guidelines.

Immunofluorescence

Cardiomyocytes were isolated as described above and fixed in Cytofix (BD Biosciences, 554655) for 5 min. After pre-blocking, cardiomyocytes were stained with anti-cardiac troponin T- (Miltenyi Biotec, 130-119-674), or anti-phospho-histone H3-AlexaFluor 594 conjugate (Cell signaling, 8481S) in 10% v/v goat serum. EdU positive cardiomyocyte nuclei were detected using Click-iT EdU Alexa Fluor 594 Imaging Kit (Invitrogen, C10339). DAPI was used to stain cardiomyocyte nuclei. Images were acquired on a confocal microscope (Leica SP5).

Supplementary Table S1. Effect of T3 on genes that regulate cardiomyocyte mitochondrial Function, DNA damage and antioxidant response

Gene	Vehicle	T3 (10 nmol/L)	*P*-value
**Mitochondrial biogenesis**			
Nrf1	1±0.066	1.60±0.22	0.04
PGC1α	1±0.13	1.75±0.17	0.01
Tfam	1±0.11	1.76±0.15	0.007
**Oxidative phosphorylation**			
Accα	1±0.17	2.29±0.37	0.02
Cox4	1±0.074	1.65±0.21	0.03
Cox5b	1±0.073	1.44±0.16	0.05
Cytc	1±0.17	2.93±0.46	0.007
Sdha	1±0.16	1.47±0.080	0.04
**Fatty acid synthesis**			
Atp5a1	1±0.095	1.01±0.059	0.92
Elvol6	1±0.085	1.76±0.24	0.02
Fasn	1±0.13	1.82±0.19	0.01
**DNA damage response**			
Atm	1±0.28	1.14±0.20	0.69
Brca1	1±0.17	0.84±0.12	0.47
Brca2	1±0.11	0.77±0.049	0.10
Dclre1a	1±0.25	0.80±0.20	0.56
Fancc	1±0.18	1.02±0.078	0.92
Mlh1	1±0.16	1.11±0.13	0.60
Mlh3	1±0.31	1.40±0.22	0.34
Mre11a	1±0.10	0.92±0.17	0.69
Parp1	1±0.45	0.79±0.075	0.67
Parp2	1±0.24	1.54±0.33	0.24
Ppm1d	1±0.14	4.91±1.06	0.01
Rad50	1±0.37	1.37±0.47	0.56
**Antioxidant response**			
Keap1	1±0.12	0.60±0.086	0.033
Nrf2	1±0.14	1.06±0.17	0.79
Pitx2	1±0.14	2.69±0.30	0.002

Values are mean ± SEM. *n* = 4. Data analyzed by Student's *t*-test.

Supplemental Table S2. Effect of T3 on genes that are associated with cardiomyocyte differentiation

Gene	Vehicle	T3 (10 nmol/L)	P-value
**Cardiomyocyte differentiation/dedifferentiation**			
Acta1	1±0.15	1.09±0.043	0.59
Acta2	1±0.35	6.13±0.89	0.001
Gata4	1±0.083	3.20±0.42	0.002
Meis1	1±0.14	1.30±0.051	0.09
Myh6	1±0.12	1.95±0.30	0.02
Myh6/Myh7	1±0.09	1.71±0.21	0.02
Myh7	1±0.12	1.16±0.19	0.51
Nppa	1±0.14	1.70±0.18	0.02
Tnni1	1±0.085	1.29±0.061	0.03
Tnni3	1±0.10	1.03±0.045	0.76
**Stem/progenitor cell**			
Kit	1±0.18	1.05±0.12	0.82
Runx1	1±0.15	2.39±0.51	0.04

Values are mean ± SEM. $n = 4$. Data analyzed by Student's *t*-test.

Supplemental Table S3. Effect of T3 on cardiomyocyte genes that regulate the cell cycle

Gene	Vehicle	T3 (10 nmol/L)	*P*-value
**Growth factor**			
Erbb2	1±0.10	1.30±0.28	0.36
Erbb4	1±0.092	1.57±0.33	0.15
Igf1	1±0.12	2.25±0.23	0.003
Igf2	1±0.05	1.25±0.2	0.27
Igf1r	1±0.26	7.77±2.51	0.04
**Cell cycle positive regulators**			
Anln	1±0.16	3.37±0.80	0.02
AurkA	1±0.20	3.19±0.48	0.006
AurkB	1±0.12	11.5±3.31	0.02
Bub1b	1±0.10	3.11±0.80	0.04
Ccna2	1±0.43	3.10±0.59	0.03
Ccnb1	1±0.31	2.20±0.34	0.04
Ccnd1	1±0.30	7.16±1.22	0.003
Cdc2	1±0.15	2.12±0.10	0.0008
Cdc20	1±0.064	5.15±1.67	0.04
Cdc25c	1±0.37	44.0±15.2	0.02
Cenpe	1±0.20	1.85±0.27	0.04
Ckap2	1±0.31	2.20±0.34	0.04
Clspn	1±0.27	3.48±0.96	0.04
E2f1	1±0.093	1.87±0.23	0.01
Ect2	1±0.27	1.83±0.07	0.03
Foxm1	1±0.26	3.40±0.82	0.03
Hist1h2af	1±0.04	7.93±2.23	0.02
Incenp	1±0.17	1.77±0.16	0.02
Plk1	1±0.23	9.05±1.36	0.001
Prc1	1±0.081	6.96±1.78	0.02
Rrm2	1±0.36	13.8±4.9	0.04
Tk1	1±0.14	16.8±6.24	0.04
Top2a	1±0.33	3.22±0.76	0.03
**Cell cycle negative regulators**			
P21	1±0.11	0.86±0.078	0.36
P27	1±0.028	0.51±0.026	0.0002

Values are mean ± SEM. $n = 4$. Data analyzed by Student's t-test.

Supplementary Table S4. Effect of T3 on proteins that regulate cardiomyocyte mitochondrial function, cardiomyocyte differentiation, cell cycle, and DNA damage

Protein	Vehicle	T3 (10 nmol/L)	*P*-value
NRF-1	1±0.24	4.86±1.10	0.01
PGC1α	1±0.23	2.25±0.23	0.02
TFAM	1±0.14	7.48±0.55	0.00003
COX4	1±0.12	8.33±0.89	0.0002
SDHA	1±0.07	2.22±0.15	0.0003
WIP-1	1±0.05	7.16±0.34	0.0000002
αMHC	1±0.13	3.39±0.16	0.00002
RUNX1	1±0.21	4.13±0.87	0.03
IGF-1	1±0.51	66.19±20.7	0.02
IGF-1R	1±0.04	35.48±0.95	0.00000003
Cyclin A2	1±0.03	13.66±0.09	0.0000001
Cyclin B1	1±0.52	8.5±2.05	0.01
Cyclin D1	1±0.03	82.46±2.17	0.000003
PLK1	1±0.34	8.05±1.12	0.003
P27	1±0.053	0.47±0.014	0.0001

Values are mean ± SEM. *n* = 4. Data analyzed by Student's *t*-test.

Supplemental Table S5. PCR primers used in this study

Gene	5' primer	3' primer
Accα	GGAGGAGGAGGGAAAGGGAT	GAAGCTTCCATCCTGGCTGT
Acta1	GACATCAAAGAGAAGCTGTG	ACTCCATACCGATAAAGGAAG
Acta2	Biorad syber green assay primer pair: qMmuCIP0032840	
Anln	TAGAGTCCTCATATTAACATTAGC	CAGAGTTGTAGAAAGTGTCATA*G*
Atm	AGCAGTCGGCAGAGCTTGTG	TGCCTTCCTCCACGCCTTTC
Atp5a1	GCGGGACTGGTCTCCAAAAA	AGGTCAACAGACGTGTCAGC
AurkA	ACGCTCTGTCTTACTGTC	GCCTTCAATCATCTCTGG
AurkB	TTTCATCGTGGCACTCAAG	ATCCTCTGCTGGTCGTAG
Brca1	GCGCCTGGACAGAAGACAGC	TGTCCAACACCCAGTCCCAC
Brca2	GCCTCCACTTGCTGTGCTTC	TGCCATCTGGGCTGAGTGAG
Bub1b	ACAACCTCTTGACAATCG	GGAGAAGAACAGTTAGCC
Ccna2	CTGGTTGAGGTGGGAGAAGA	ATGACTCAGGCCAGCTCTGT
Ccnb1	CTAAAGTCGGAGAGGTTG	GTCTTCACTGTAGGATAGG
Ccnd1	AGGAGCAGAAGTGCGAAGAGGA	AAAGTGCGTTGTGCGGTAGC
Cdc2	TACTTACACCAAATCCTCCAG	TACCACAGCGTCACTACC
Cdc20	CCAGAAGGCTACCAGAAC	GGATGTCACCAGAACCAG
Cdc25c	CGGTGCCAGGGAAACACCCG	GCTTCGCCAGCTCAGCAGCT
Cenpe	AAGTGACTCGGACAGATTC	GGCTCCACATTCTCTACG
Ckap2	AGGACTGATTCGTCTAATAC	GGTGACTACTGAATGAGG
Clspn	TGAGTGAAAGAGATTCTGATG	TAAGTTCTCCTGAGTGTCC
Cox4	GTCTTGGTCTTCCGGTTGCG	GTTCATCTCGGCGAAGCTCT
Cox5b	GCGTTGTTAGACTCCCACCA	TGCTGATGGACGGGACGGGAC*TAGA*
Cytc	ACTACAGCCACGCTTTACCC	AGCCTTTTCACCCCCACAAA
Dclre1a	GCGTTCCGACCCACAGGATG	ACCGAAAGCTGCCGACATTG
E2f1	AAGGGAGAGGGAGACAGAC	AGGACAGCAGAGGAGCAC
Ect2	AGGACCTTCCATTCGAACCT	GACTCGGGTGTGTTGGAGAT
Elvol6	TCTGATGAACAAGCGAGAGCCA	CCTAGTTCGGGTGCTTTGCT
Erbb2	CGCTGCCCCAGTGGTGTGAAG	GCAGCCTCGTTCGTCCAGGT
Erbb4	GCCCCAAAGCCAACGTGGAGT	GCGGCATCAGCTGCGTAACC
Fancc	CGGTGCTCCATGTCTTGCTG	GCGGAGACCCACGAGTACAG
Fasn	AGAAGAGCCATGGAGGAGGT	GAAGCGTCTCGGGATCTCTG
Foxm1	CTATTGGATTCGGGTGTAG	AGTAGTGTTGTTCTGTAGC
Gata4	TAAATCTAAGACGCCAGCAGGT	CCCAGTTTTTCTTCAGGAGTTG
Hist1h2af	AGTACCTGACGGCCGAGAT	CTTTCCCTTGGGCTTATGGT
Igf1	ATAAAGATACACATCATGTC	TTGTAGGCTTCAGTGGGGCAC
Igf1r	CGCCTGGAAAACTGCACG	AGCTGCCCAGGCACTCCG
Igf2	CGCTTCAGTTTGTCTGTTCGG	TGGGTGGTAACACGATCAGG
Incenp	GAGAGGGTGGAACAGATG	TCCTTCTTCCGTTGTAGC
Keap1	CATCCACCCTAAGGTCATGGA	GACAGGTTGAAGAACTCCTCC
Kit	GTAACAACAAAGAGCAAATC	TCTCCTCGACAACCTTCC
Meis1	GTTGTCCAAGCCATCACCTT	ATCCACTCGTTCAGGAGGAA
Mlh1	TCTCAGGCCAGCAGAGTGAC	CGGAGGTAGGAGGTGTGAGC
Mlh3	AGAAGGTGTTGGCCTCCCAG	TCCAGGTCAGCTAAGGGCAG
Mre11a	AGGGCGAAGAGGAGCCAGAG	ATCTCCAGCCCAGTGTCTCG
Myh6	AGTACTTTGCCAGCATTGCAG	CGGACACCTCTCCCTGAGAGA
Myh7	AATATTTTGCTGTTATTGCCGC	CAGTCGTCTCTCCTTGGGAGAT
Nppa	CCTGTGTACAGTGCGGTGTC	ACACACCACAAGGGCTTAGG
Nrf1	GCACCTTTGGAGAATGTGGT	CTGAGCCTGGGTCATTTTGT
Nrf2	TGCCCCTGGAAGTGTCAAACA	CAACAGGGAGGTTAATGATTT
P21	TATCCAGACATTCAGAGCCACA	CAGGGCAGAGGAAGTACTGG
P27	CAAACTCTGAGGACCGGCAT	TTCTTAATTCGGAGCTGTTTACG
Parp1	TCAACGACACCTGCCTGCTG	GGTGCAGAGGCACTAGGGAG
Parp2	GGGCAAGCATAGCACCAAGG	TGCTGGTCCTAAGGGCACTG
PGC1α	AAACTTGCTAGCGGTCCTCA	ACGTCTTTGTGGCTTTTGCT
Pitx2	AGGGAGGGAGGCAAGAAAAG	CTTGAAAGAGCCAGGGAACG
Plk1	CATTGAGTGCCACCTTAG	GCCATACTTGTCCGAATAG
Ppm1d	CTGACTGATAGCCCTACTTACAACA	GAGAAGGCATTACTGCGAACA
Prc1	GCTTGTCTGACCTGTTGAG	GAAGAGTAGTGATGGGTTTGG
Rad50	GCCTTGGATGAGCCGACAAC	AGTTGGAAGTTGCGCTGCTG
Rrm2	AGCGAGTAGGCGAGTATC	GGTGTAGCCAGTTGGTTG
Runx1	Biorad syber green assay primer pair: qMmuCEP0041879	
Sdha	CCAACCGGCTTGGAGCAAAT	ATCATACTCATCGACCCGCA
Tfam	AGGAGGCAAAGGATGATTCGG	GTCTCCGGATCGTTTCACAC

Supplemental Table S5. PCR Primers Used in this Study (Continued)

Gene	5' primer	3' primer
Tk1	TTTCCACCCACGGACTCTC	ATACTTGATGACCAGGCACTTG
Tnni1	CCACGAGGACTAAACTAGGCAC	GAGGACGCTTGAACTTCCCA
Tnni3	GATGCGGCTGGGGAACC	GCTGTCGGCATAAGTCCTGA
Top2a	CATTGCCGTTTAAGCCTGTC	TTCATCCTCATCCTTCTCATCC

Supplementary Figures with Figure Legends

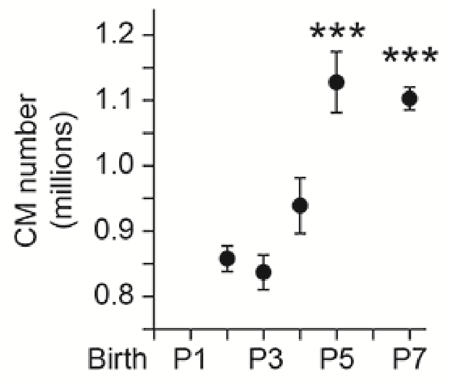

Supplementary Figure S1. Changes in cardiomyocyte (CM) number during the first week of life. Assessments of ventricular cardiomyocyte numbers between postnatal day-2 (P2) and P7. n's at P2, P3, P4, P5 and P7 were 12, 7, 8, 5 and 11, respectively. ****P* < 0.001 compared to P2 using ANOVA.

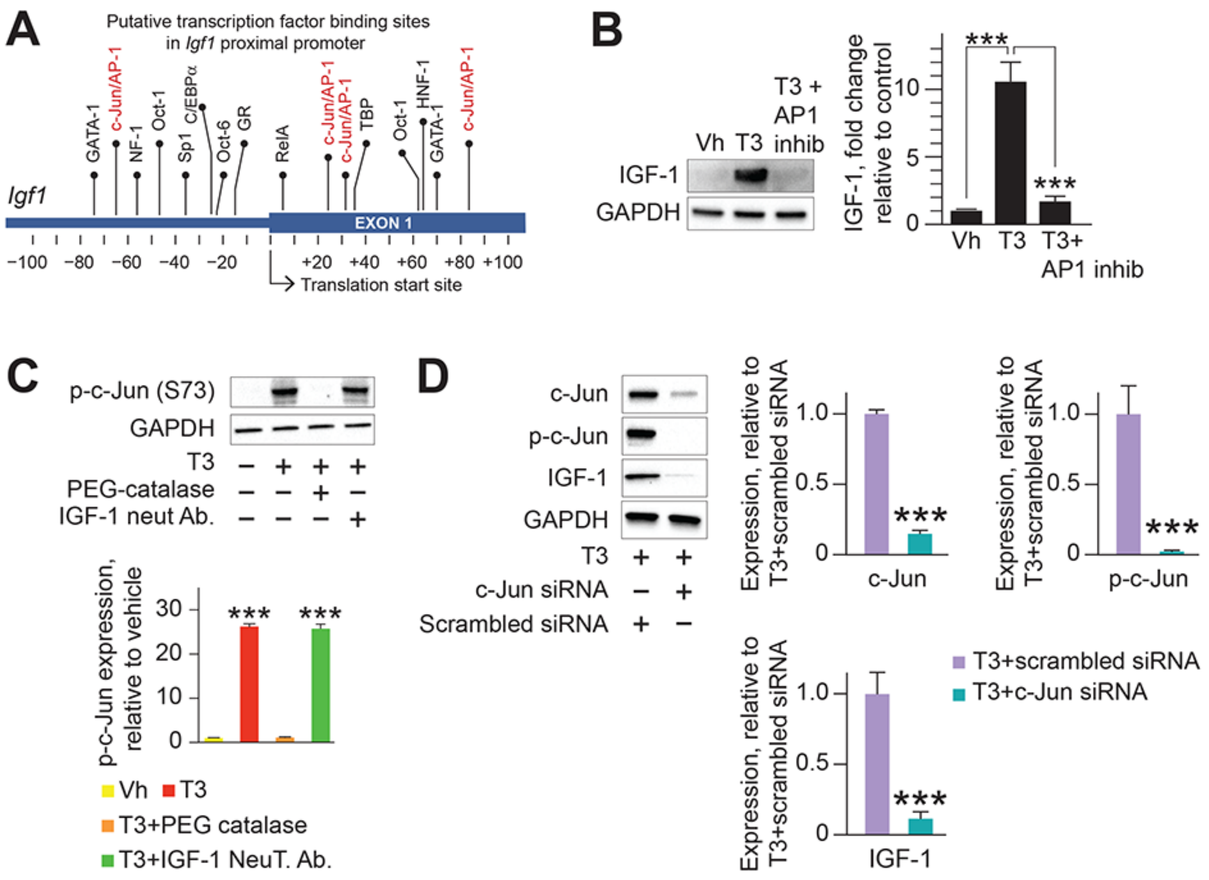

Supplementary Figure S2. T3 stimulates IGF-1 formation in neonatal cardiomyocytes by activating (phosphorylation) c-Jun, a transcription factor that is a component of the AP1 complex. (**A**) Schematic showing the location of putative transcription factor binding sites on the proximal *Igf1* promoter by *in silico* analysis using AliBaba2. Multiple c-Jun/AP1 binding sites are present on *Igf1* promoter. (**B**) Representative immunoblot of lysates from cultured neonatal cardiomyocytes showing that AP1 inhibitor blocks T3-dependent IGF-1 formation. Quantitative analyses of these data are also shown. (**C**) Representative immunoblot of neonatal cardiomyocytes lysate showing that PEG-catalase, but not an IGF-1 neutralizing antibody, inhibits c-Jun phosphorylation by T3. (**D**) Representative immunoblot showing that c-Jun knockdown by siRNA inhibits T3-dependent c-Jun phosphorylation and IGF-1 expression. Error bars indicate SEM. n = 4/group. ***$P < 0.001$.

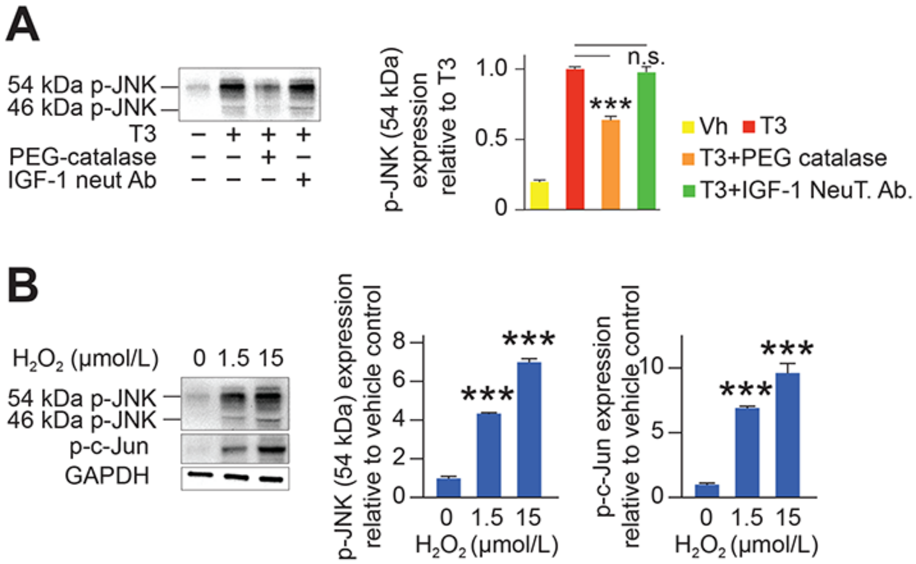

Supplementary Figure S3. Evidence that H_2O_2 mediates T3-stimulated phosphorylation of 54 kDa JNK in neonatal cardiomyocytes. (**A**) Representative immunoblot showing that PEG-catalase, but not an IGF-1 neutralizing antibody, attenuates JNK phosphorylation by T3. (**B**) Representative immunoblot and quantitative analyses of neonatal cardiomyocytes lysate showing dose dependent effect of H_2O_2 on JNK and c-Jun phosphorylation. Immunoblot shows that H_2O_2 treatment to cardiomyocytes mainly phosphorylates high molecular weight 54 kDa band of JNK2. Error bars indicate SEM. n = 4/group. ****P* < 0.001, compared to T3 treatment; n.s., nonsignificant.

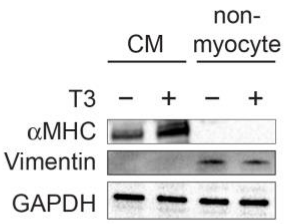

Supplementary Figure S4. Purity and quality of isolated cardiomyocytes for T3 studies. Representative immunoblots show the purity of isolated cardiomyocytes (CM) by showing robust expression of αMHC, a cardiomyocyte specific marker, as compared to vimentin, a non-myocyte marker. This figure also shows that cardiomyocytes are responsive to canonical T3 signaling as evident, for example, by increased αMHC expression. Non-myocytes are devoid of αMHC but positive for vimentin. Representative of 4 biological replicates.

Figure 1B

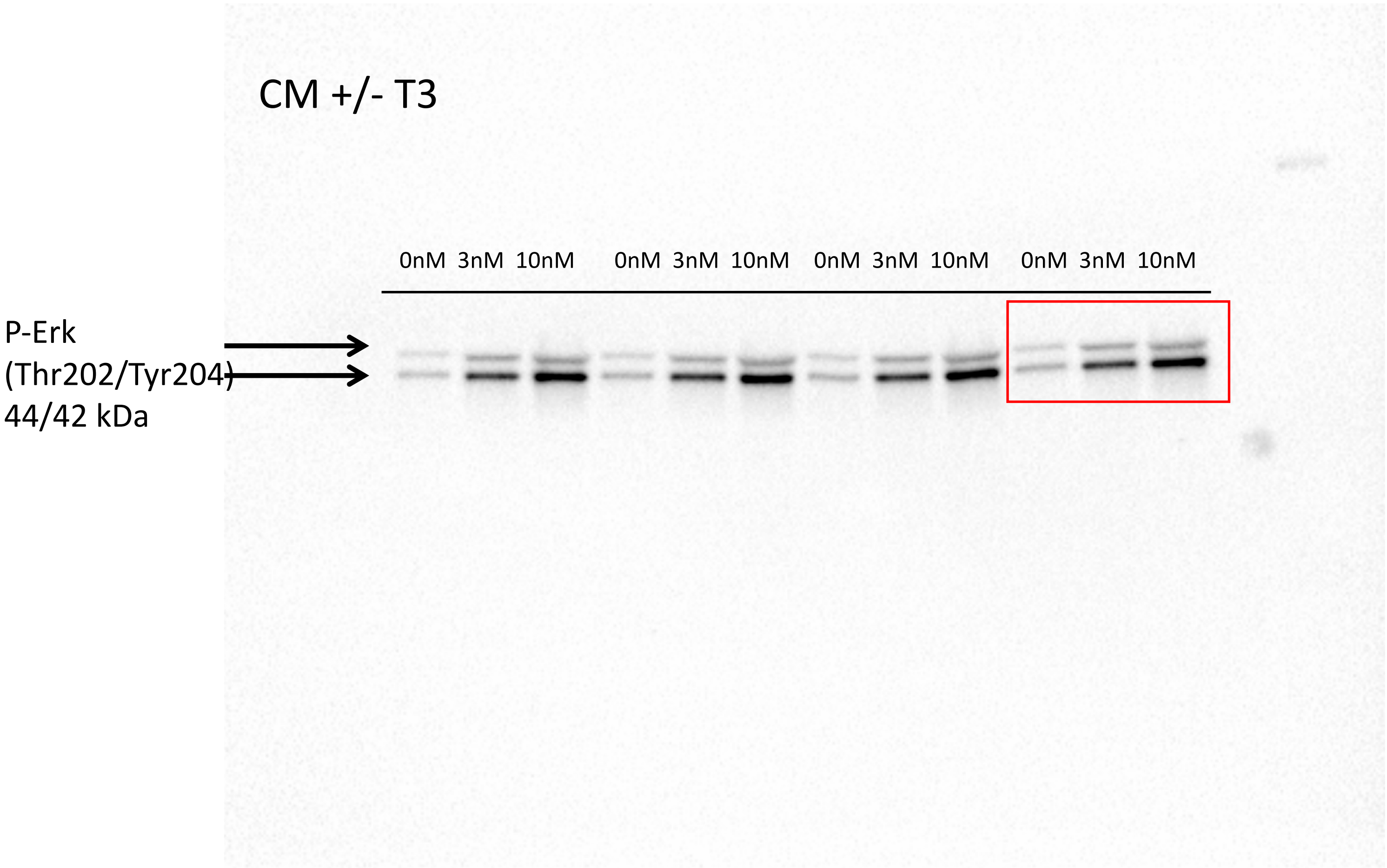

CM +/- T3
0nM 3nM 10nM 0nM 3nM 10nM 0nM 3nM 10nM 0nM 3nM 10nM
P-Erk
(Thr202/Tyr204)
44/42 kDa

Figure 1B

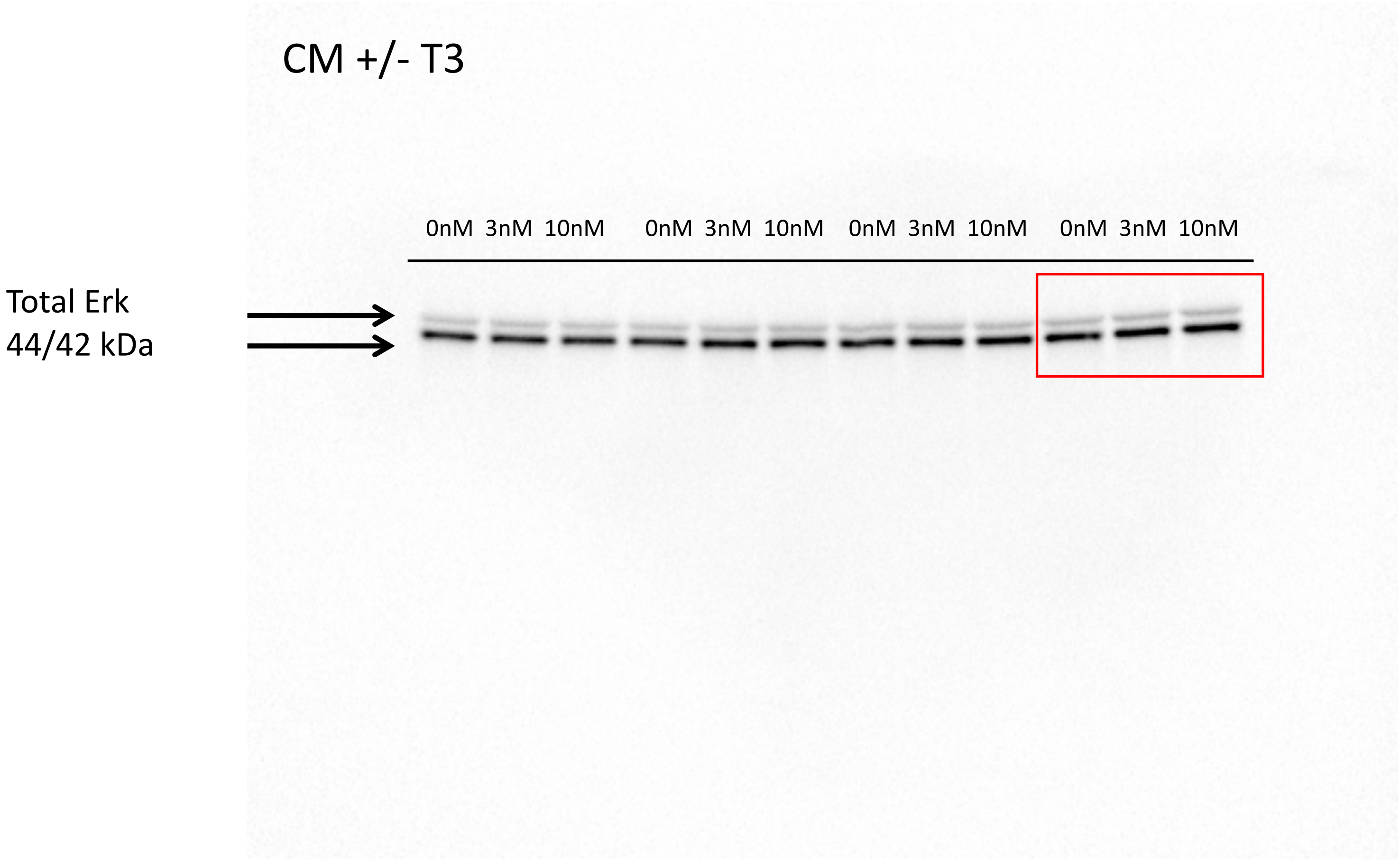
CM +/- T3
0nM 3nM 10nM 0nM 3nM 10nM 0nM 3nM 10nM 0nM 3nM 10nM
Total Erk
44/42 kDa

Figure 1B

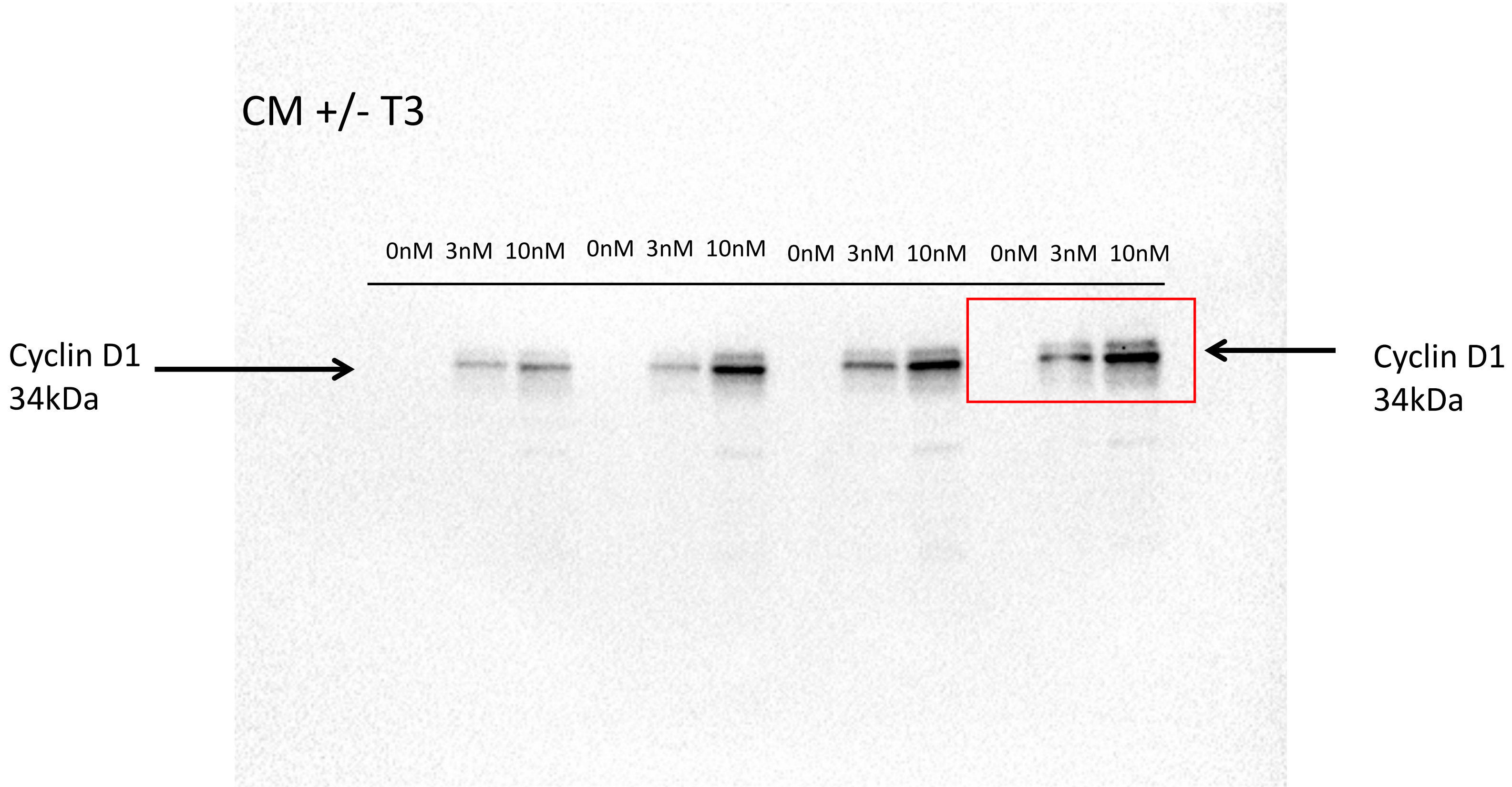

CM +/- T3
0nM 3nM 10nM 0nM 3nM 10nM 0nM 3nM 10nM 0nM 3nM 10nM
Cyclin D1
34kDa
Cyclin D1
34kDa

Figure 1B

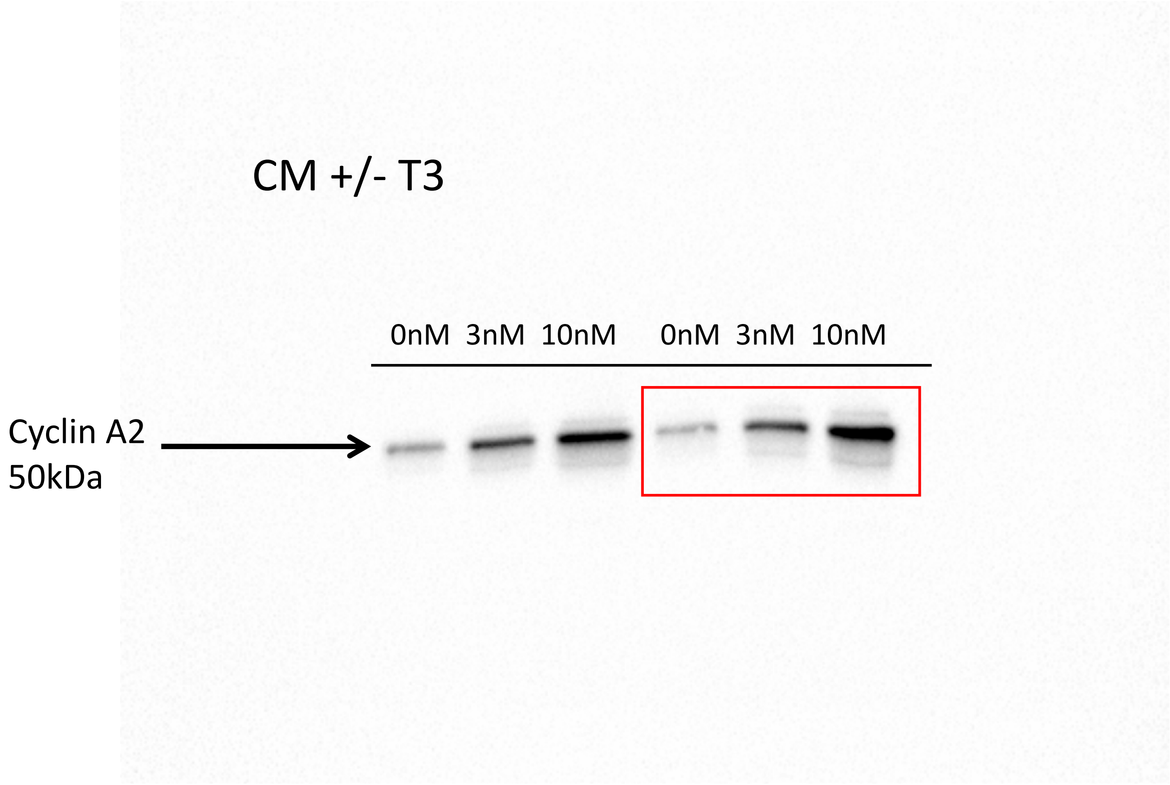

Figure 1B

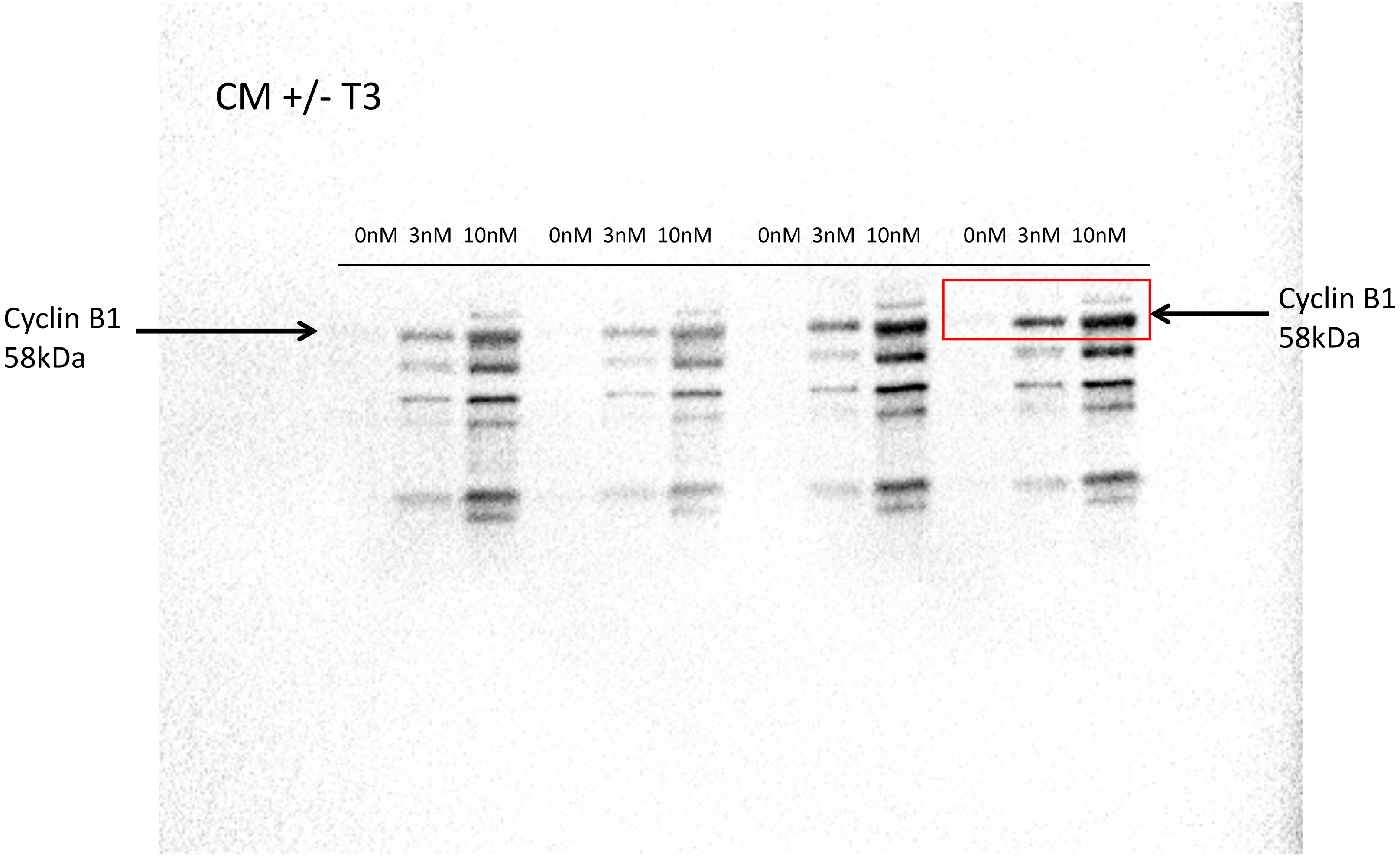

CM +/- T3
0nM 3nM 10nM 0nM 3nM 10nM 0nM 3nM 10nM 0nM 3nM 10nM
Cyclin B1
58kDa
Cyclin B1
58kDa

Figure 1B

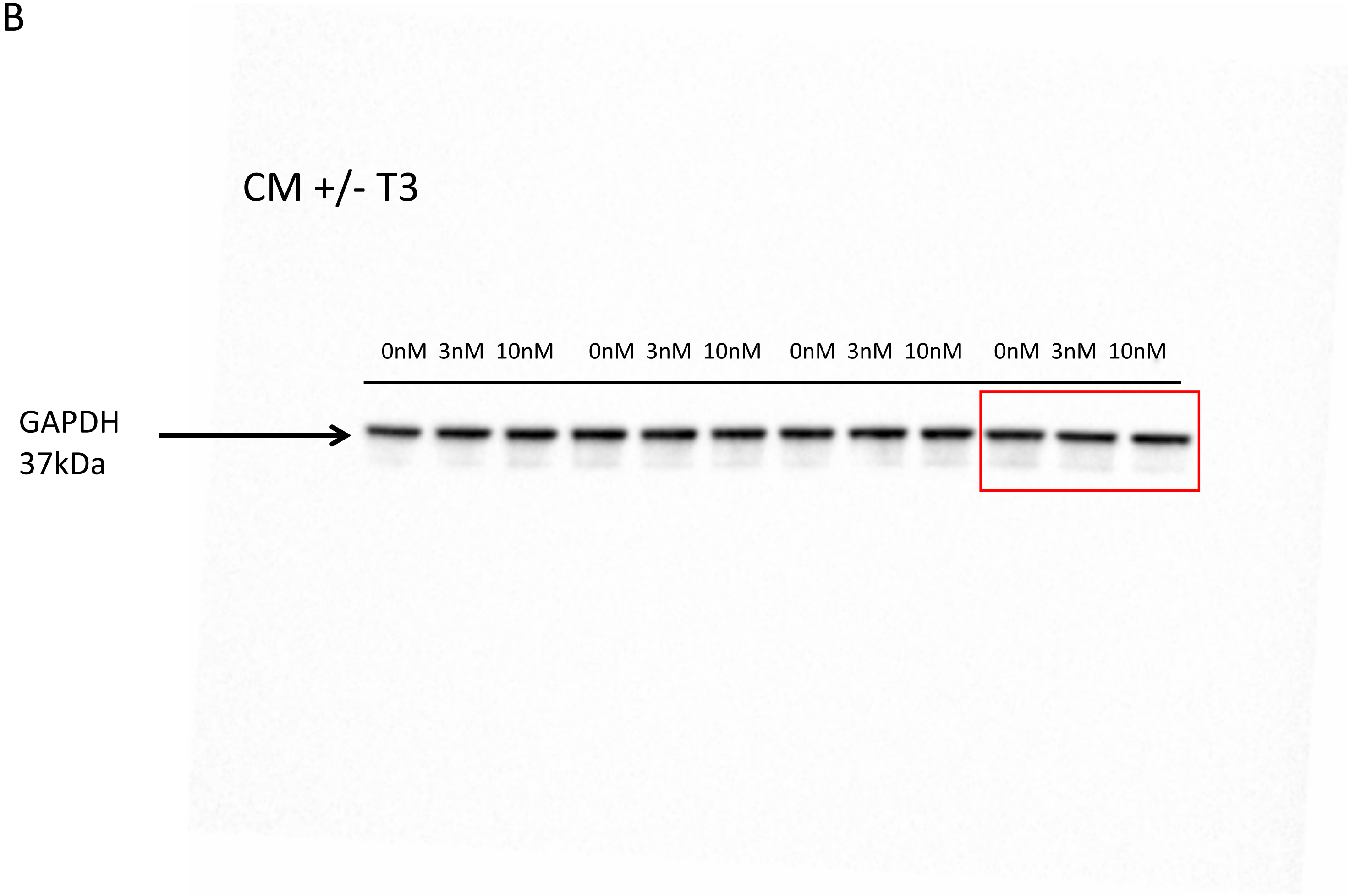
CM +/- T3
0nM 3nM 10nM 0nM 3nM 10nM 0nM 3nM 10nM 0nM 3nM 10nM
GAPDH
37kDa

Figure 1C

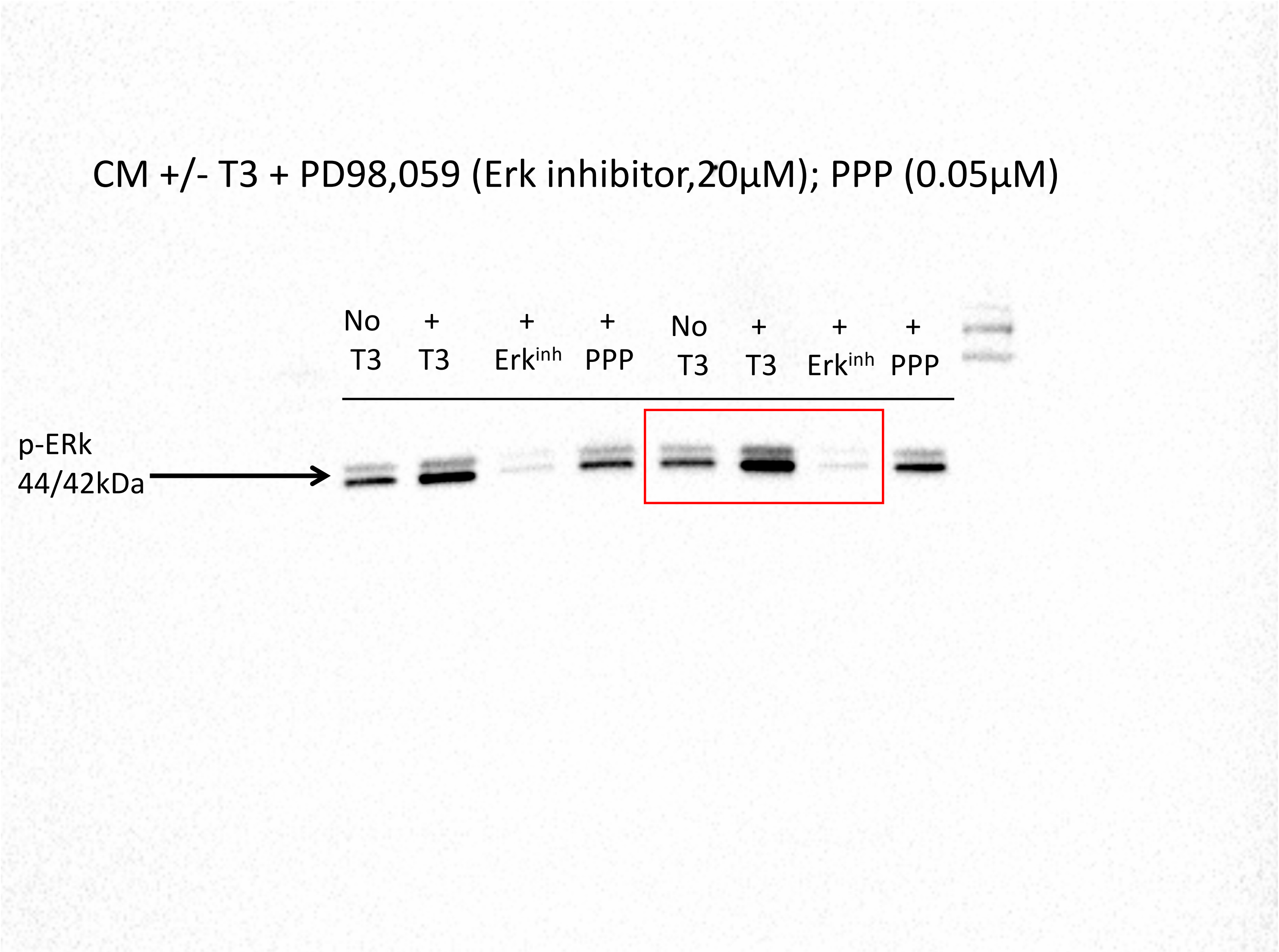

CM +/- T3 + PD98,059 (Erk inhibitor,20µM); PPP (0.05µM)
No T3
+ T3
+ Erkinh
+ PPP
No T3
+ T3
+ Erkinh
+ PPP
p-ERk 44/42kDa

Figure 1C

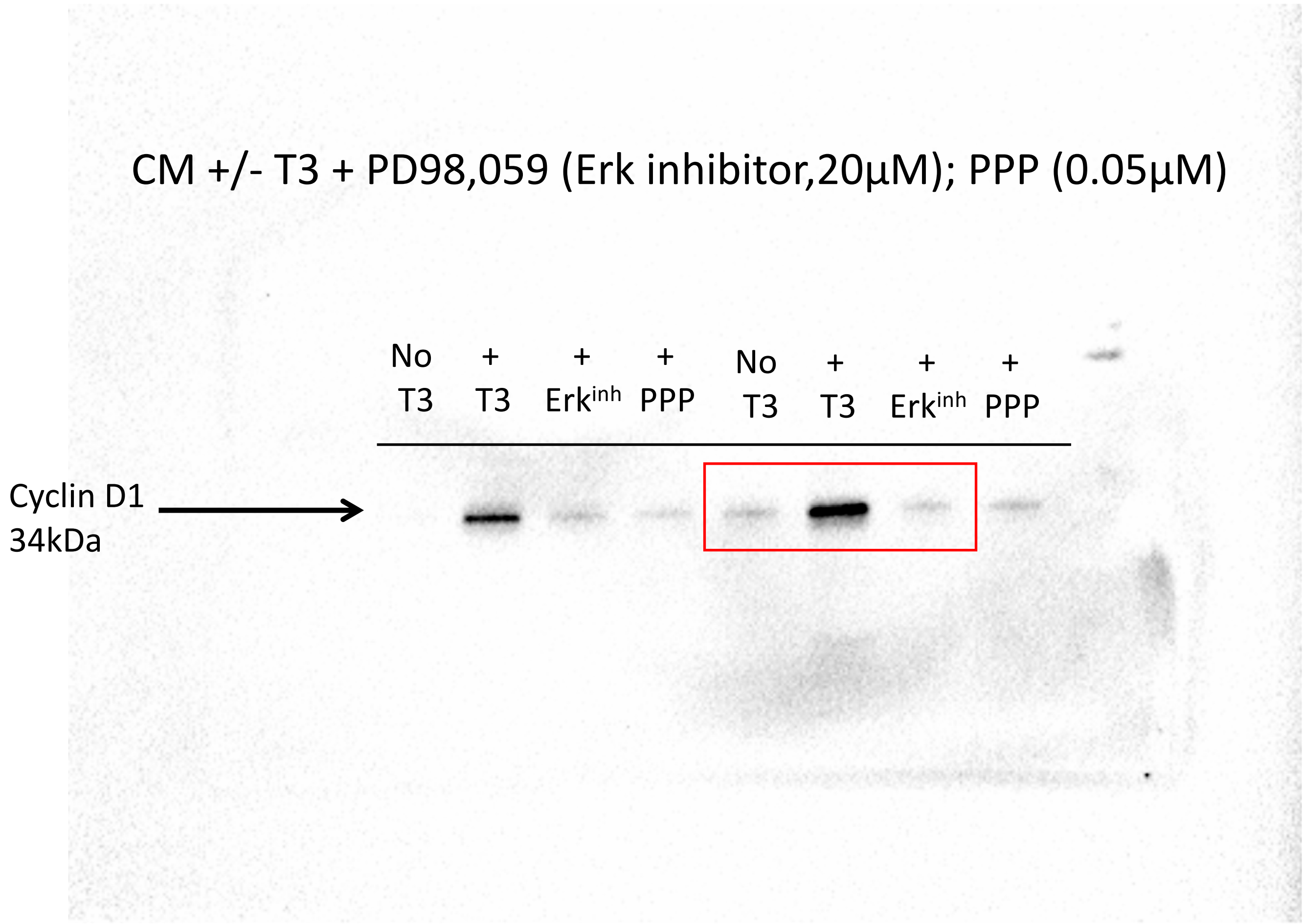
CM +/- T3 + PD98,059 (Erk inhibitor,20μM); PPP (0.05μM)
No T3
+ T3
+ Erkinh
+ PPP
No T3
+ T3
+ Erkinh
+ PPP
Cyclin D1
34kDa

Figure 1C

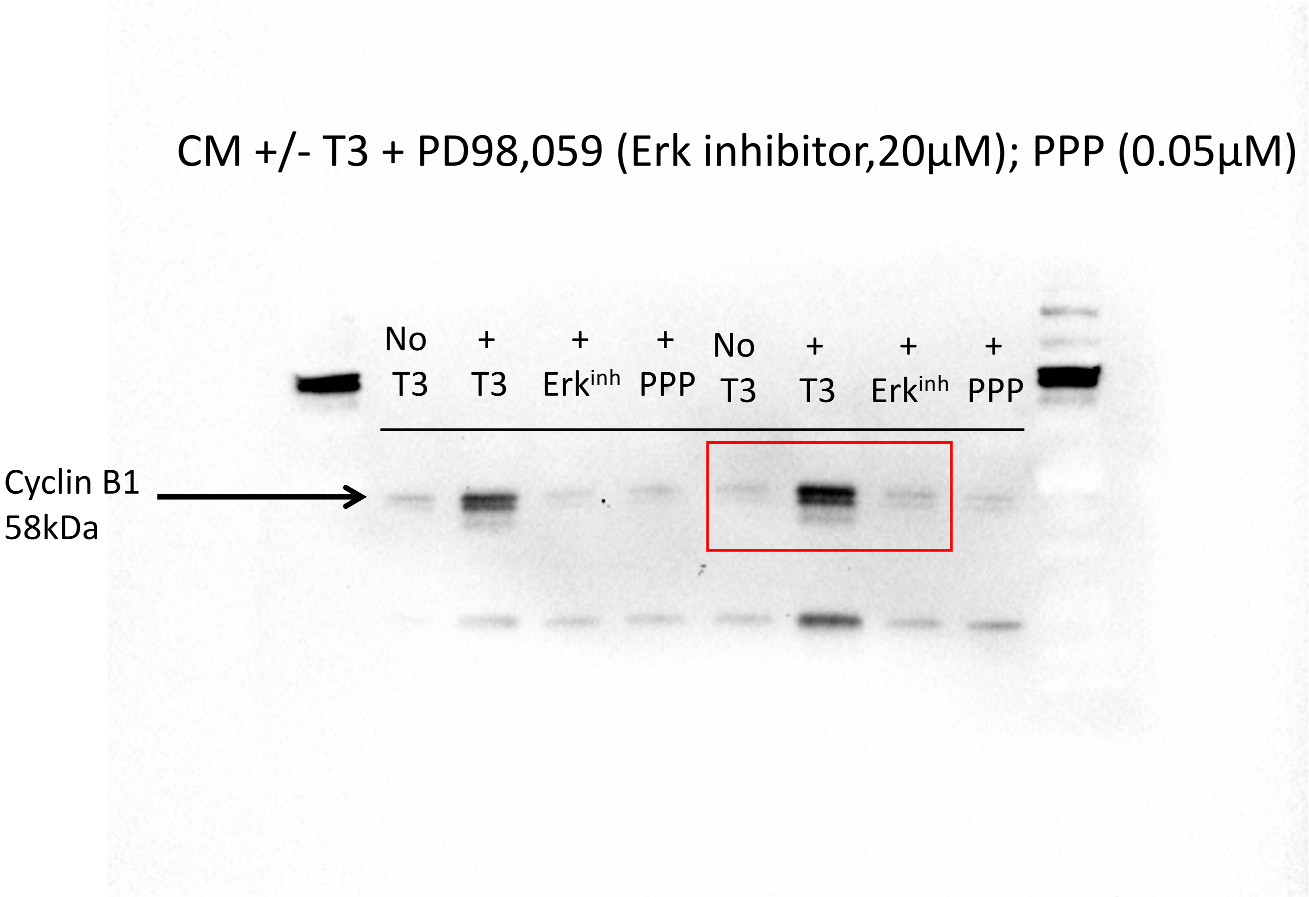

CM +/- T3 + PD98,059 (Erk inhibitor,20µM); PPP (0.05µM)
No T3
+ T3
+ Erkinh
+ PPP
No T3
+ T3
+ Erkinh
+ PPP
Cyclin B1 58kDa

Figure 1C

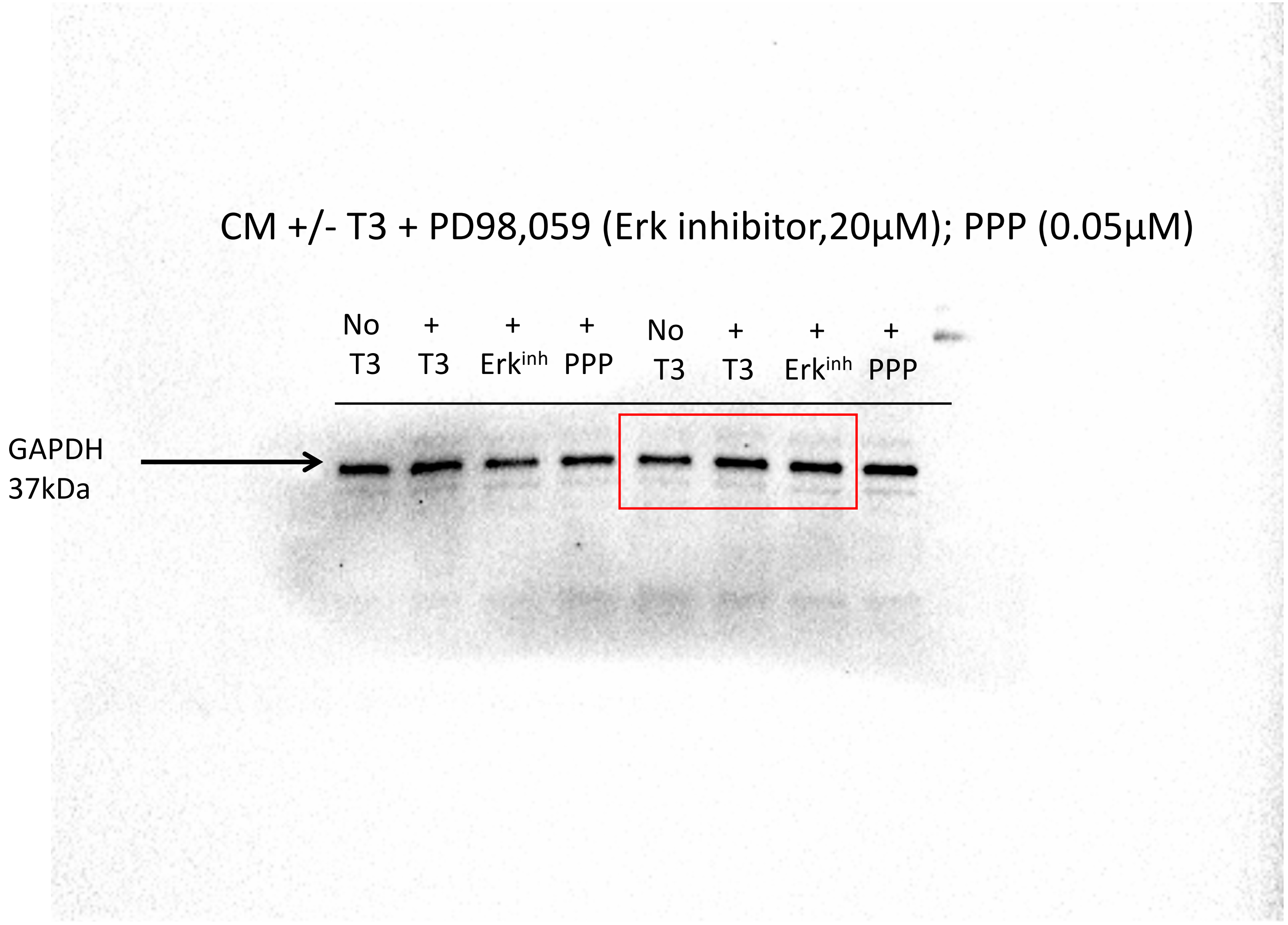
CM +/- T3 + PD98,059 (Erk inhibitor,20µM); PPP (0.05µM)
No T3
+ T3
+ Erkinh
+ PPP
No T3
+ T3
+ Erkinh
+ PPP
GAPDH
37kDa

Figure 1D

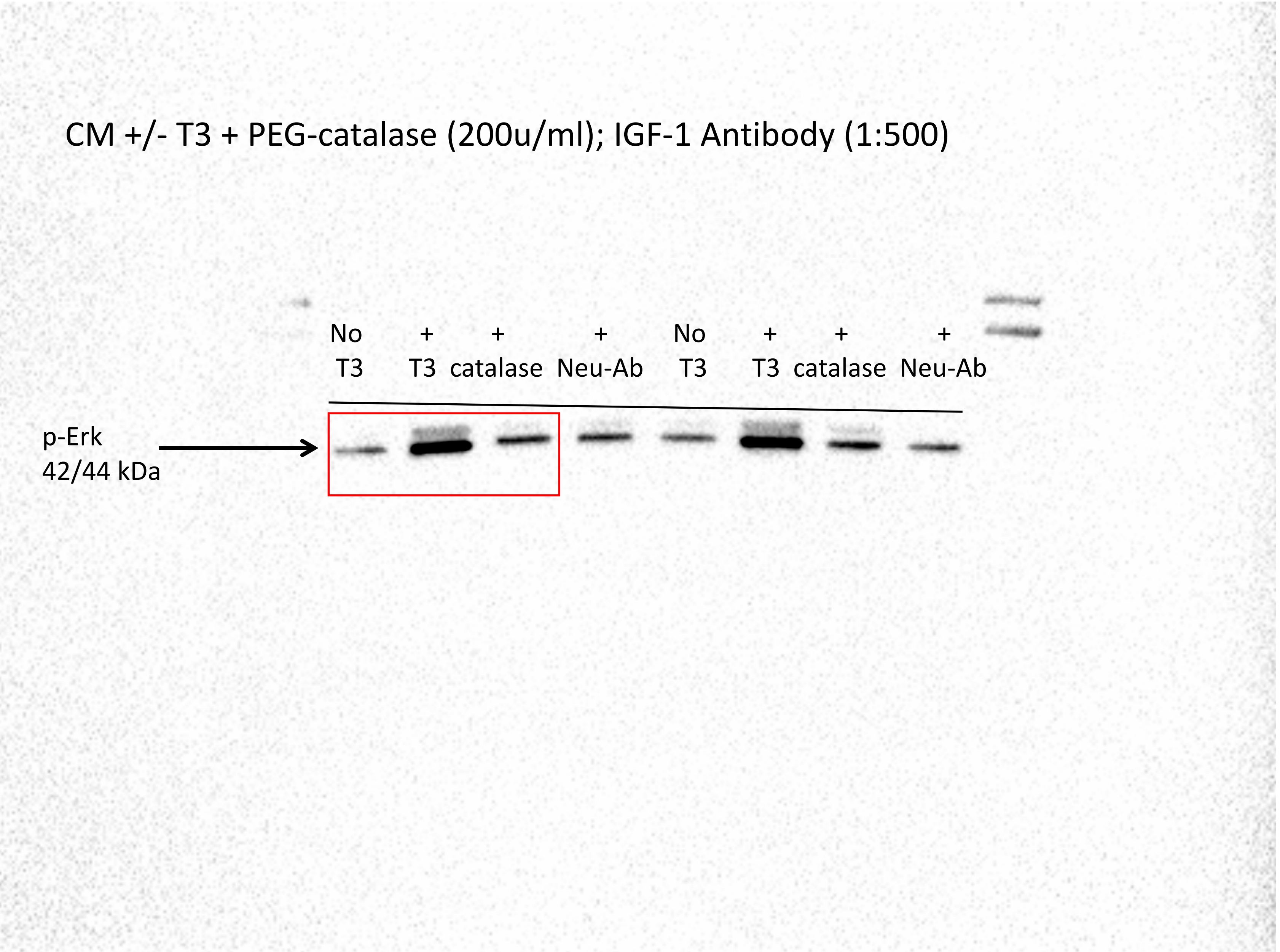
CM +/- T3 + PEG-catalase (200u/ml); IGF-1 Antibody (1:500)
No T3
+ T3
+ catalase
+ Neu-Ab
No T3
+ T3
+ catalase
+ Neu-Ab
p-Erk 42/44 kDa

Figure 1D

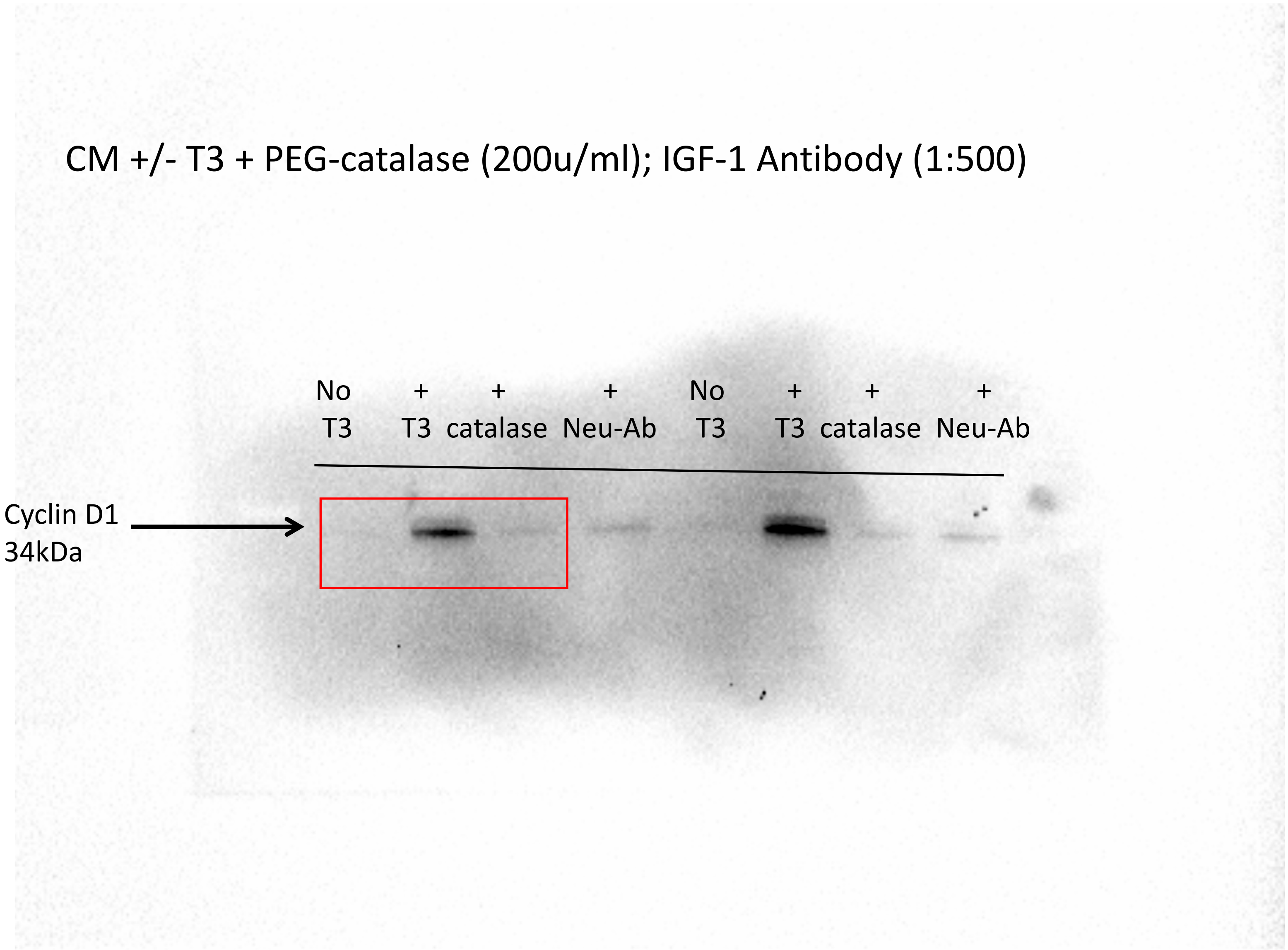

CM +/- T3 + PEG-catalase (200u/ml); IGF-1 Antibody (1:500)
No T3
+ T3
+ catalase
+ Neu-Ab
No T3
+ T3
+ catalase
+ Neu-Ab
Cyclin D1
34kDa

Figure 1D

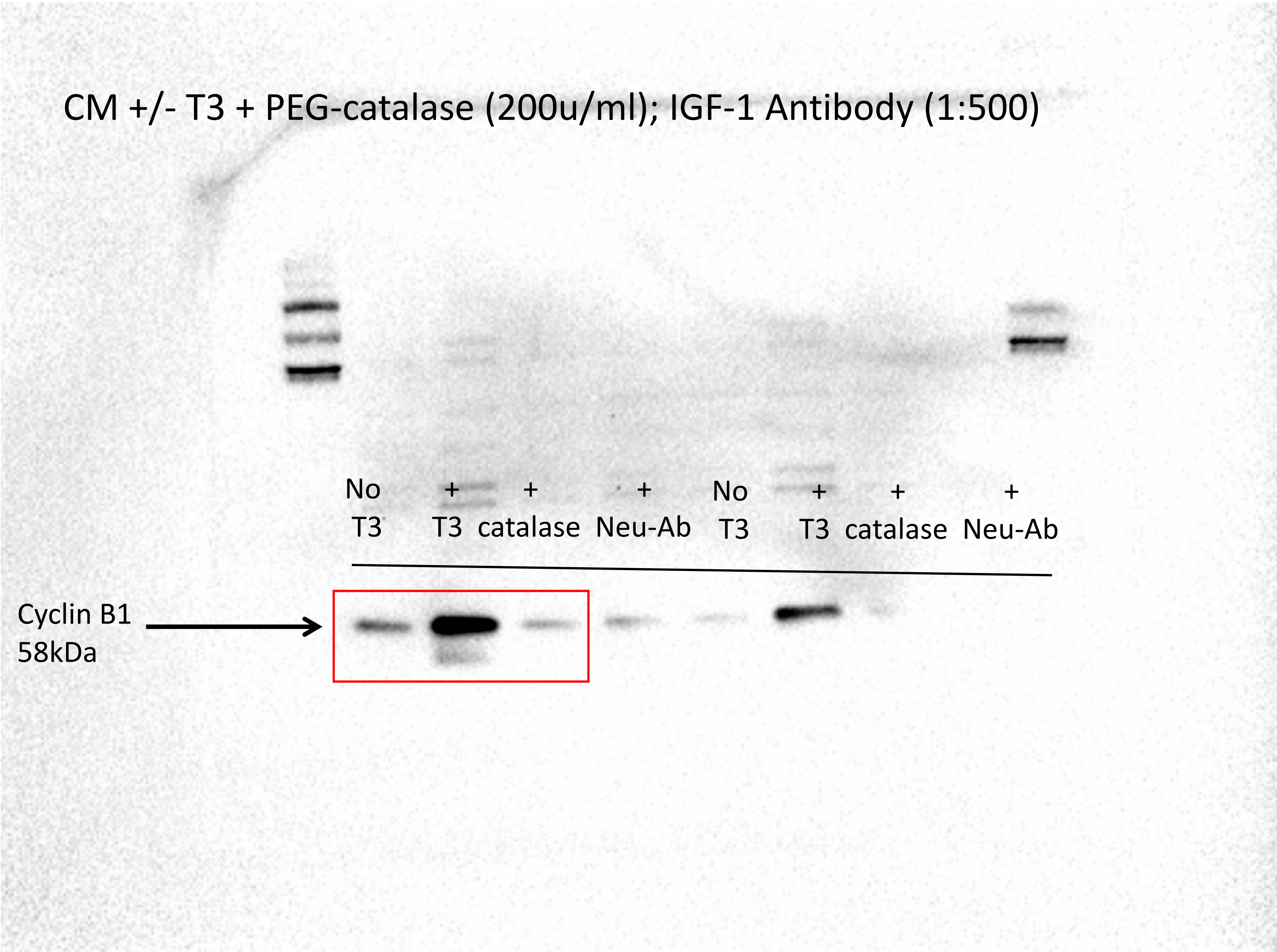
CM +/- T3 + PEG-catalase (200u/ml); IGF-1 Antibody (1:500)
No T3
+ T3
+ catalase
+ Neu-Ab
No T3
+ T3
+ catalase
+ Neu-Ab
Cyclin B1 58kDa

Figure 1D

CM +/- T3 + PEG-catalase (200u/ml); IGF-1 Antibody (1:500)

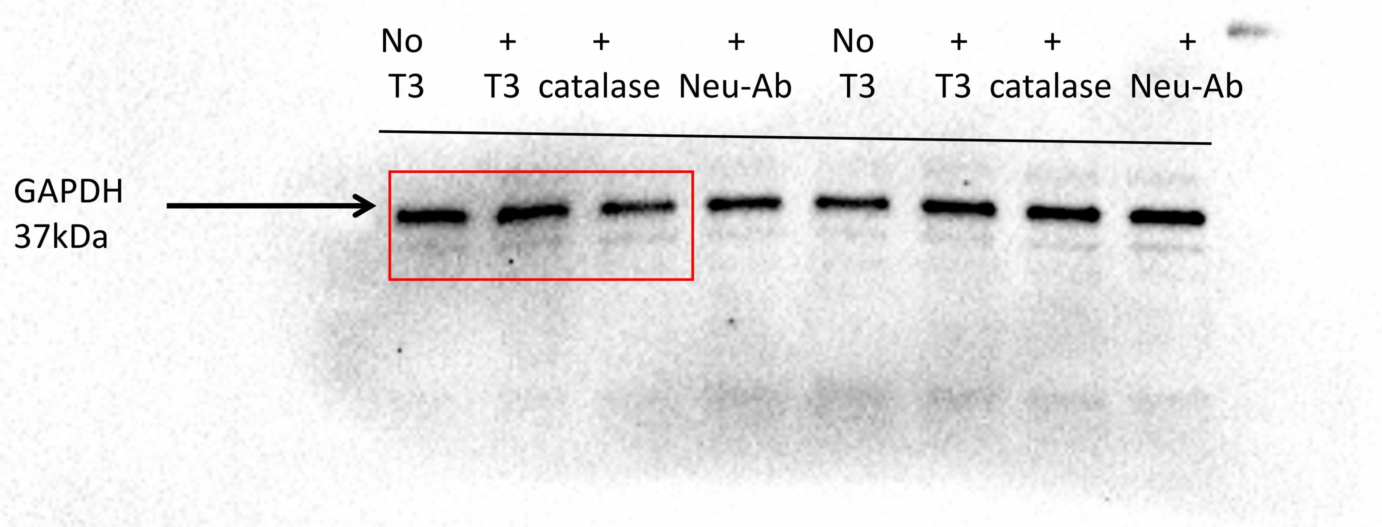

Figure 1E

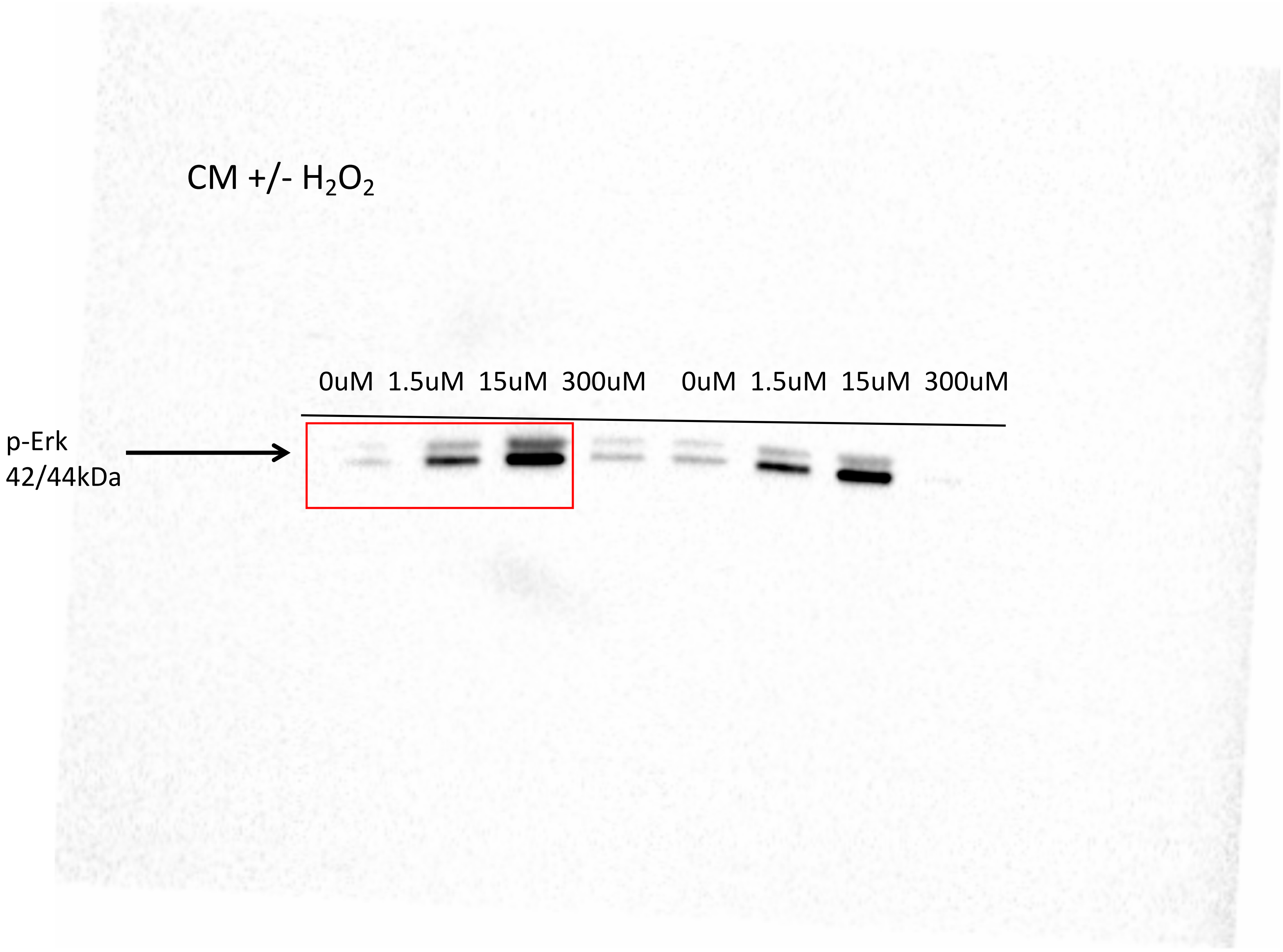

Figure 1E

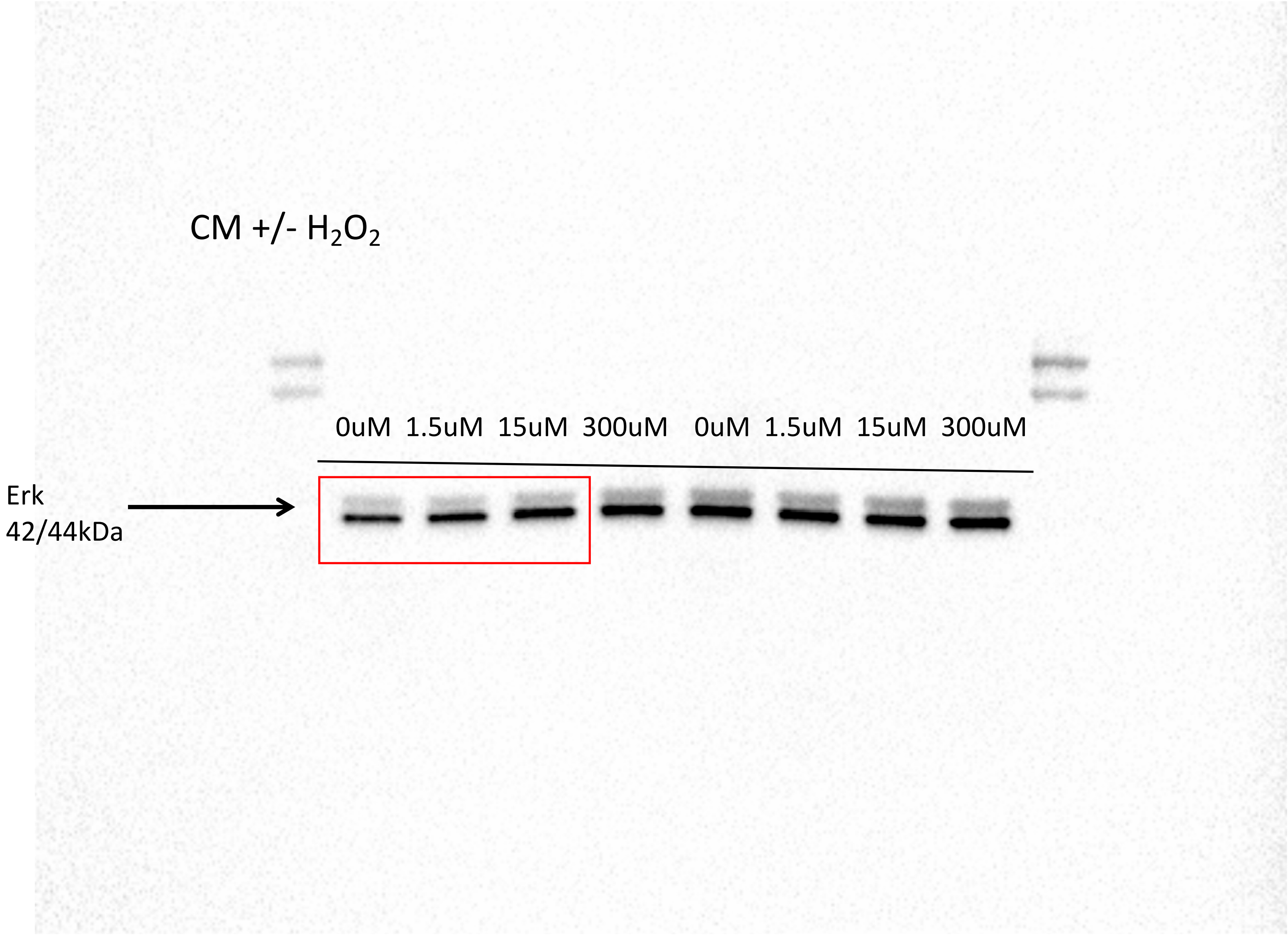

Figure 1E

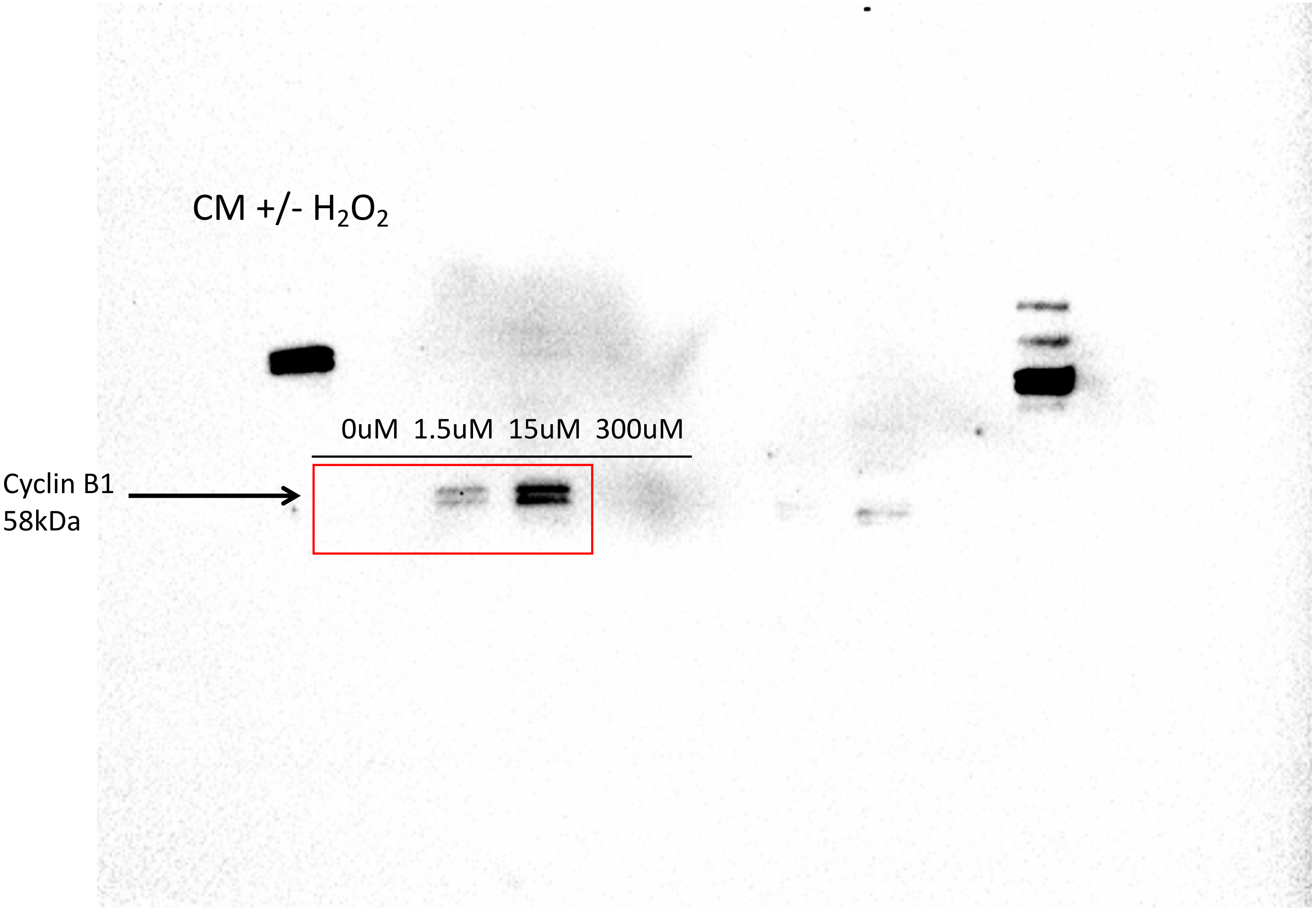

Figure 1E

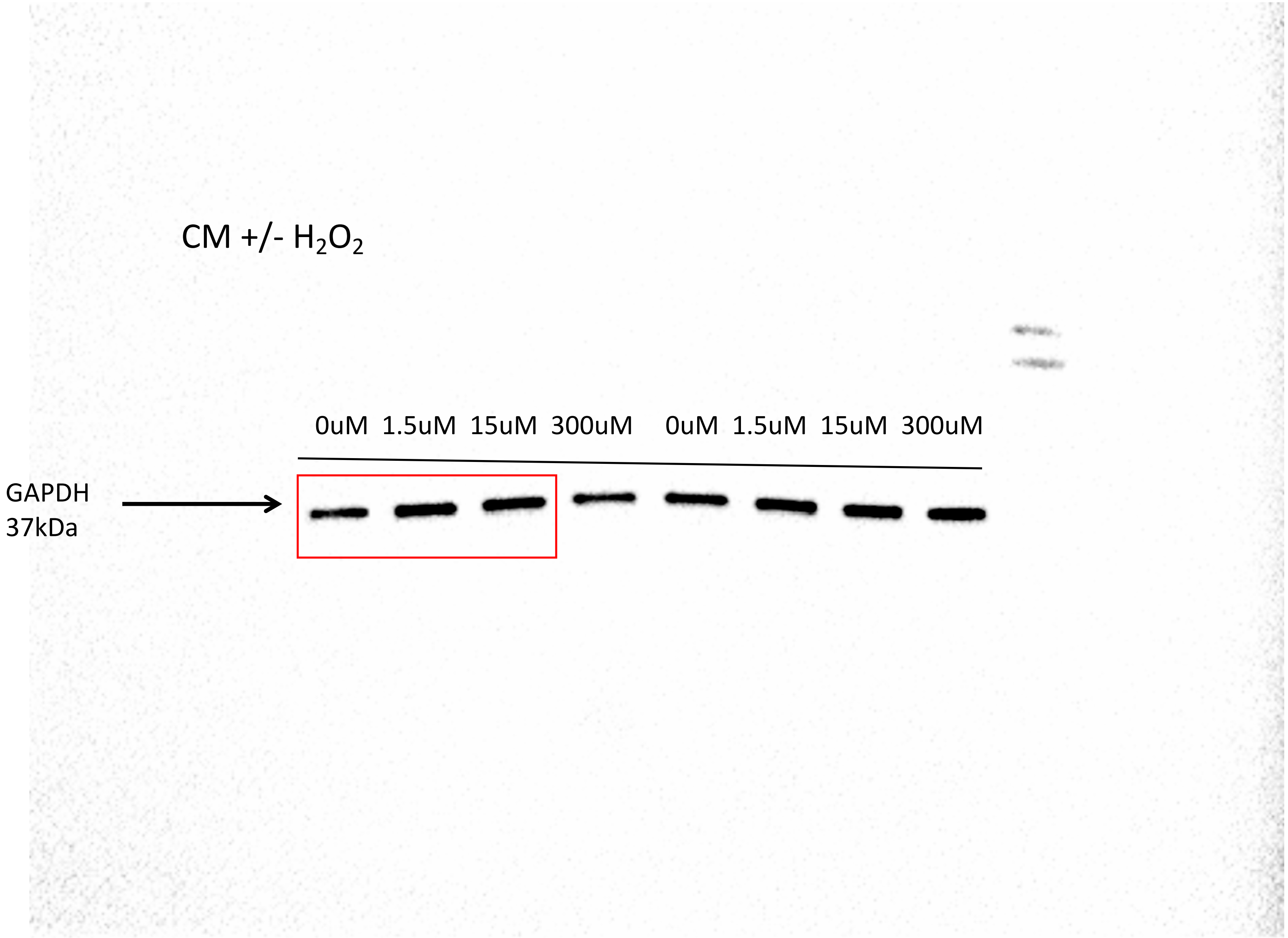

Figure 1F

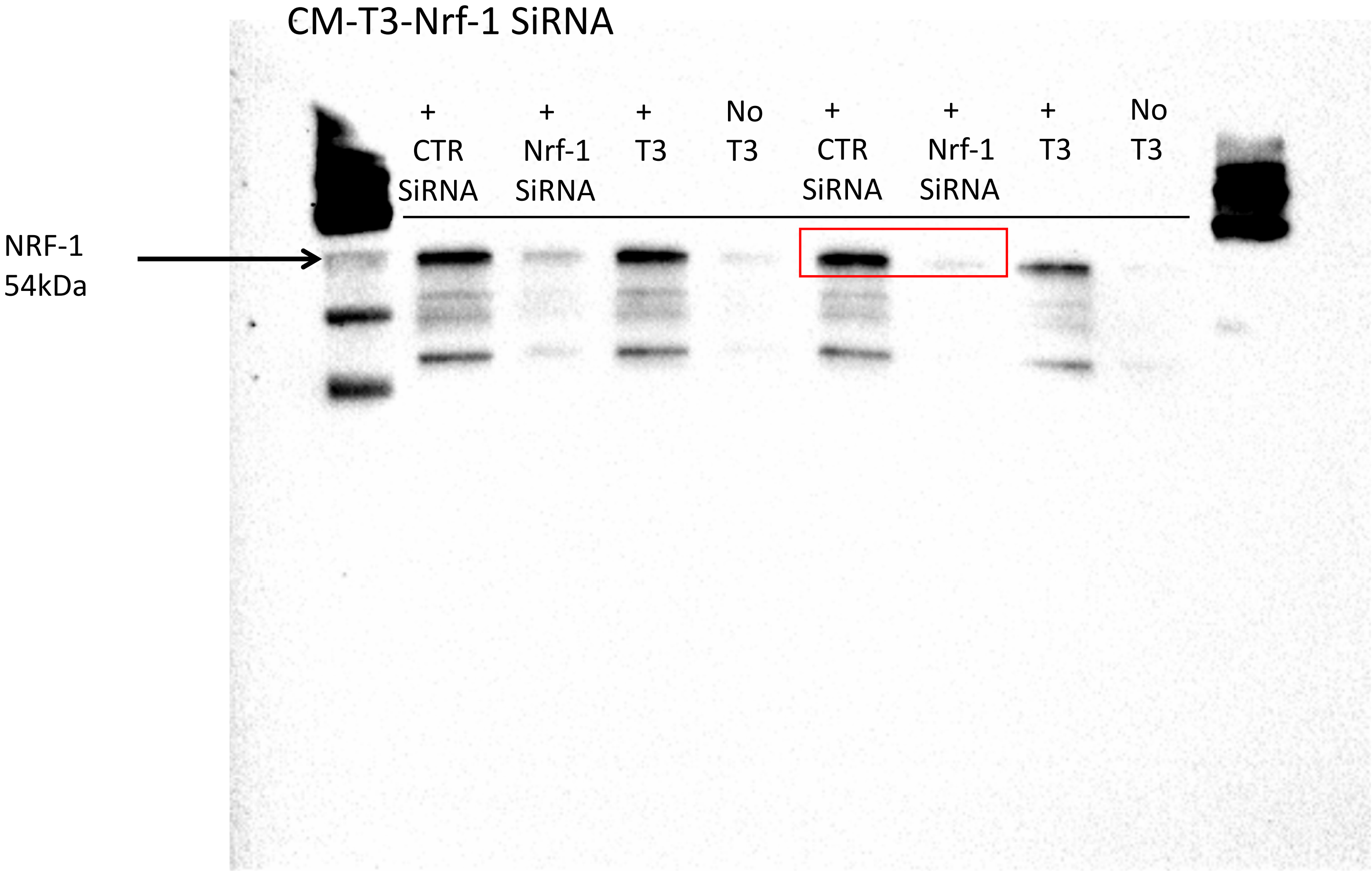
CM-T3-Nrf-1 SiRNA
+ CTR SiRNA
+ Nrf-1 SiRNA
+ T3
No T3
+ CTR SiRNA
+ Nrf-1 SiRNA
+ T3
No T3
NRF-1 54kDa

Figure 1F

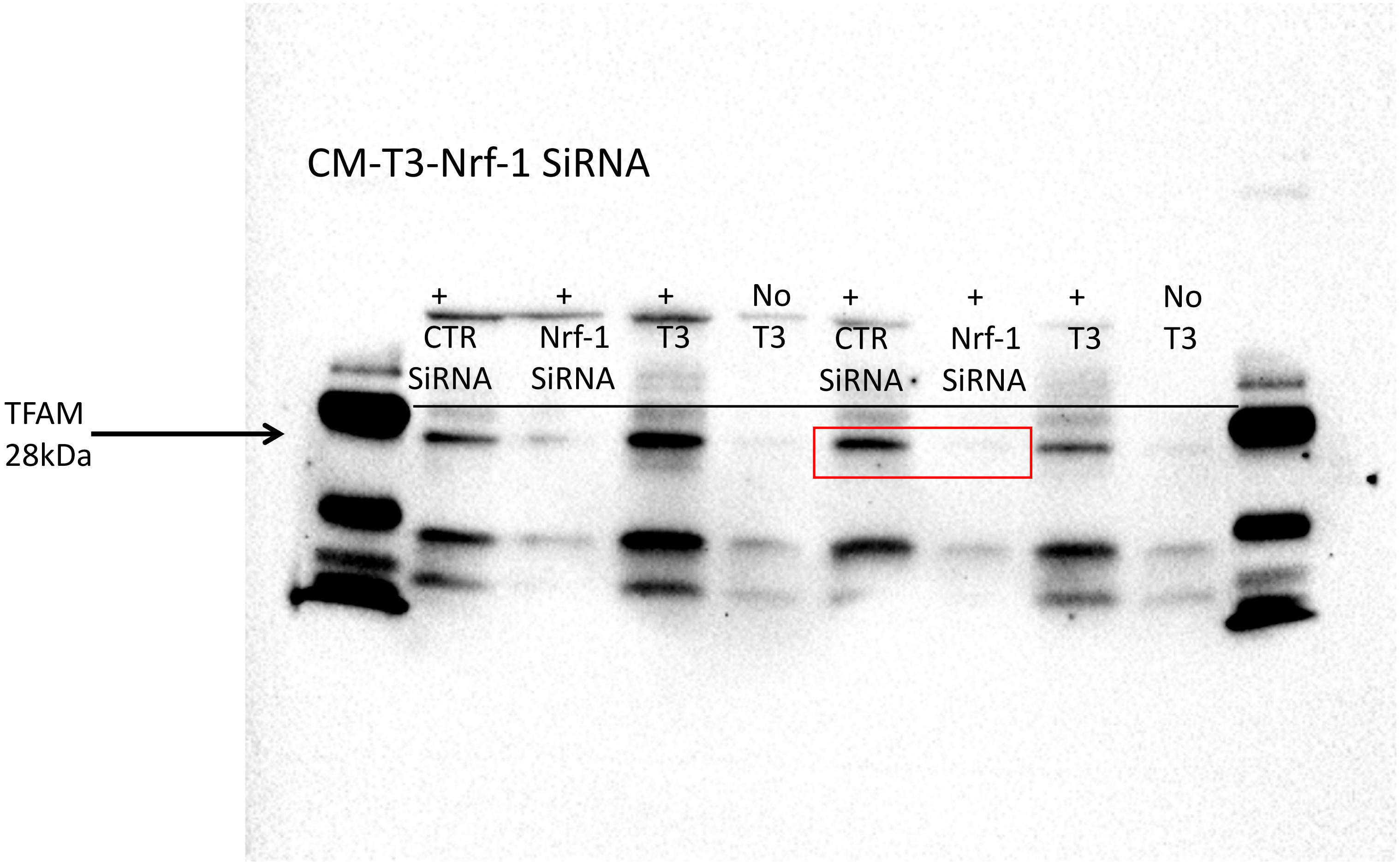
CM-T3-Nrf-1 SiRNA
+
CTR
SiRNA
+
Nrf-1
SiRNA
+
T3
No
T3
+
CTR
SiRNA
+
Nrf-1
SiRNA
+
T3
No
T3
TFAM
28kDa

Figure 1F

CM-T3-Nrf-1 SiRNA

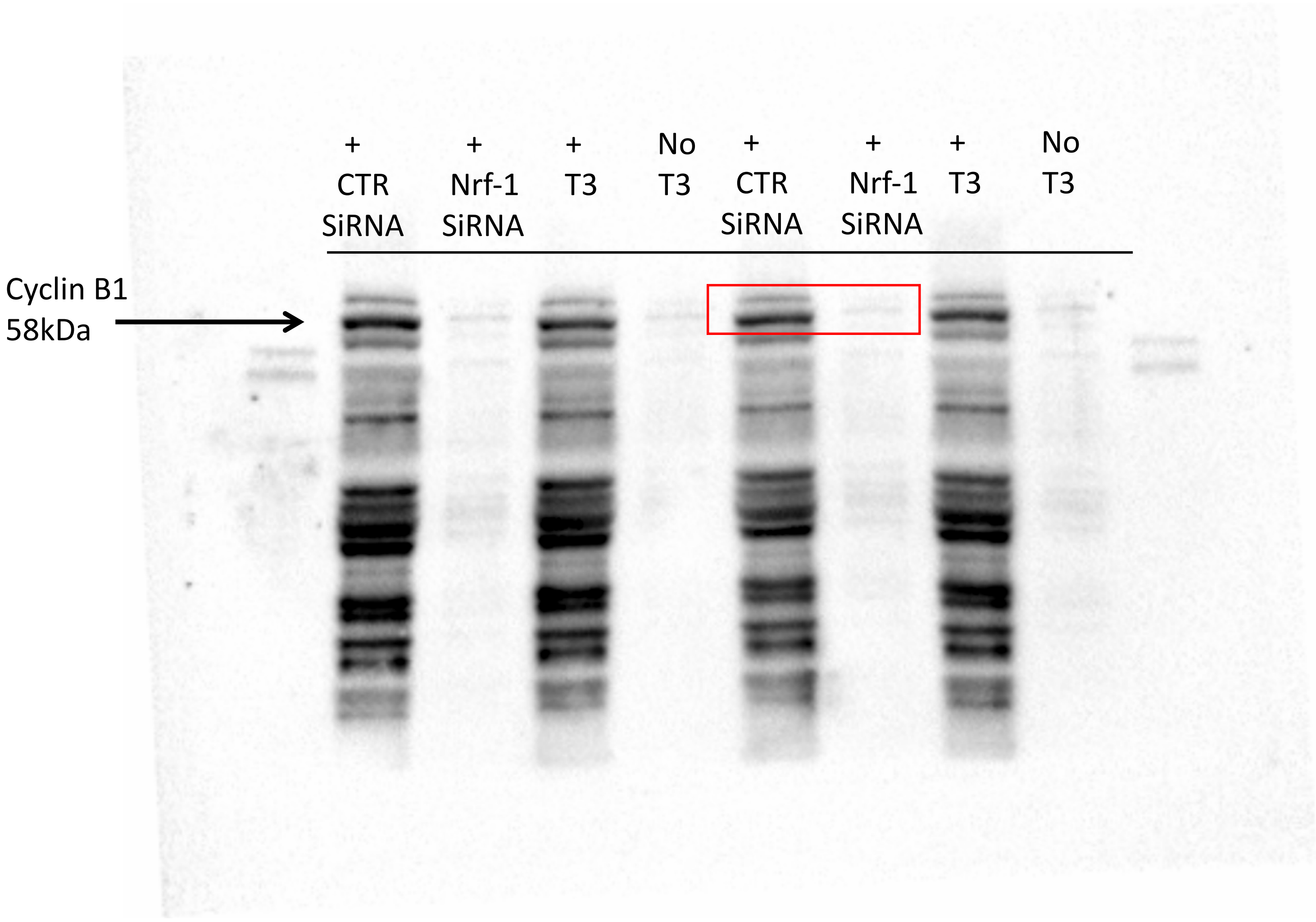

Figure 1F

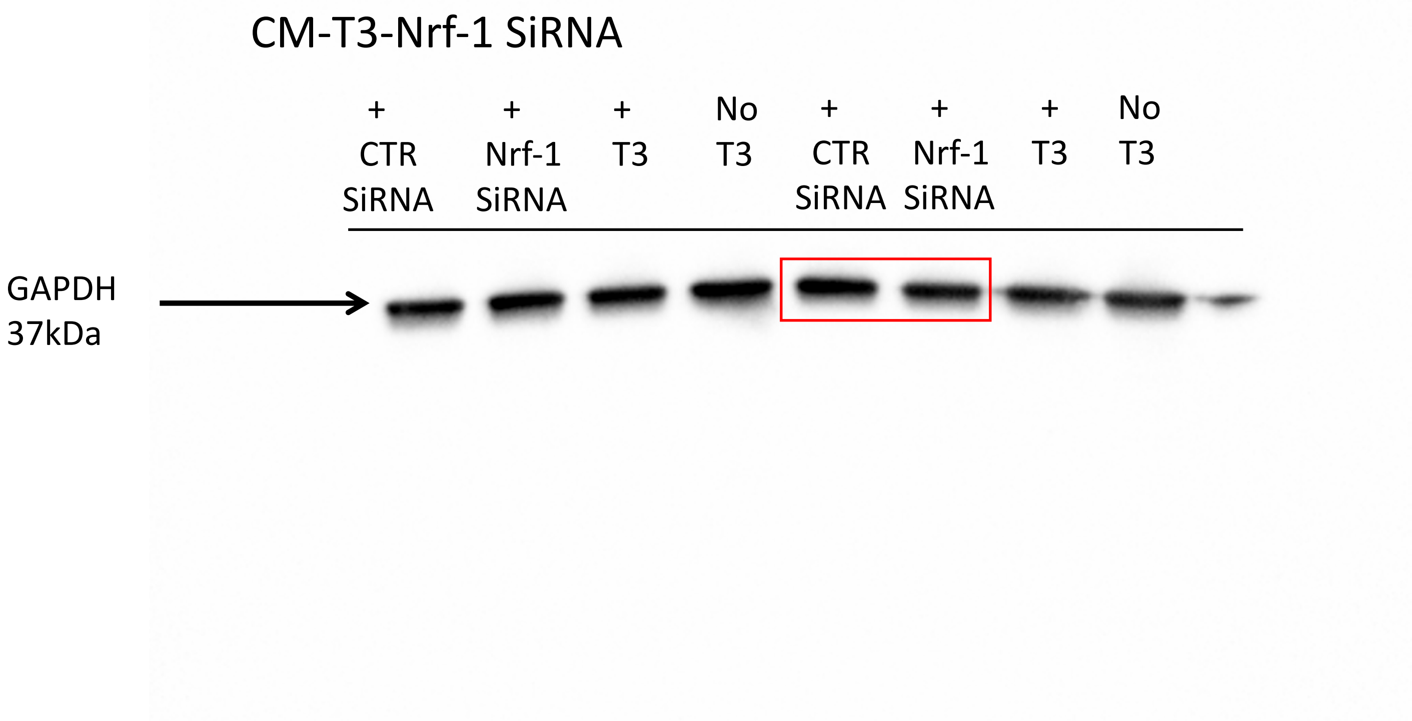
CM-T3-Nrf-1 SiRNA
+ CTR SiRNA
+ Nrf-1 SiRNA
+ T3
No T3
+ CTR SiRNA
+ Nrf-1 SiRNA
+ T3
No T3
GAPDH
37kDa

Figure 4B

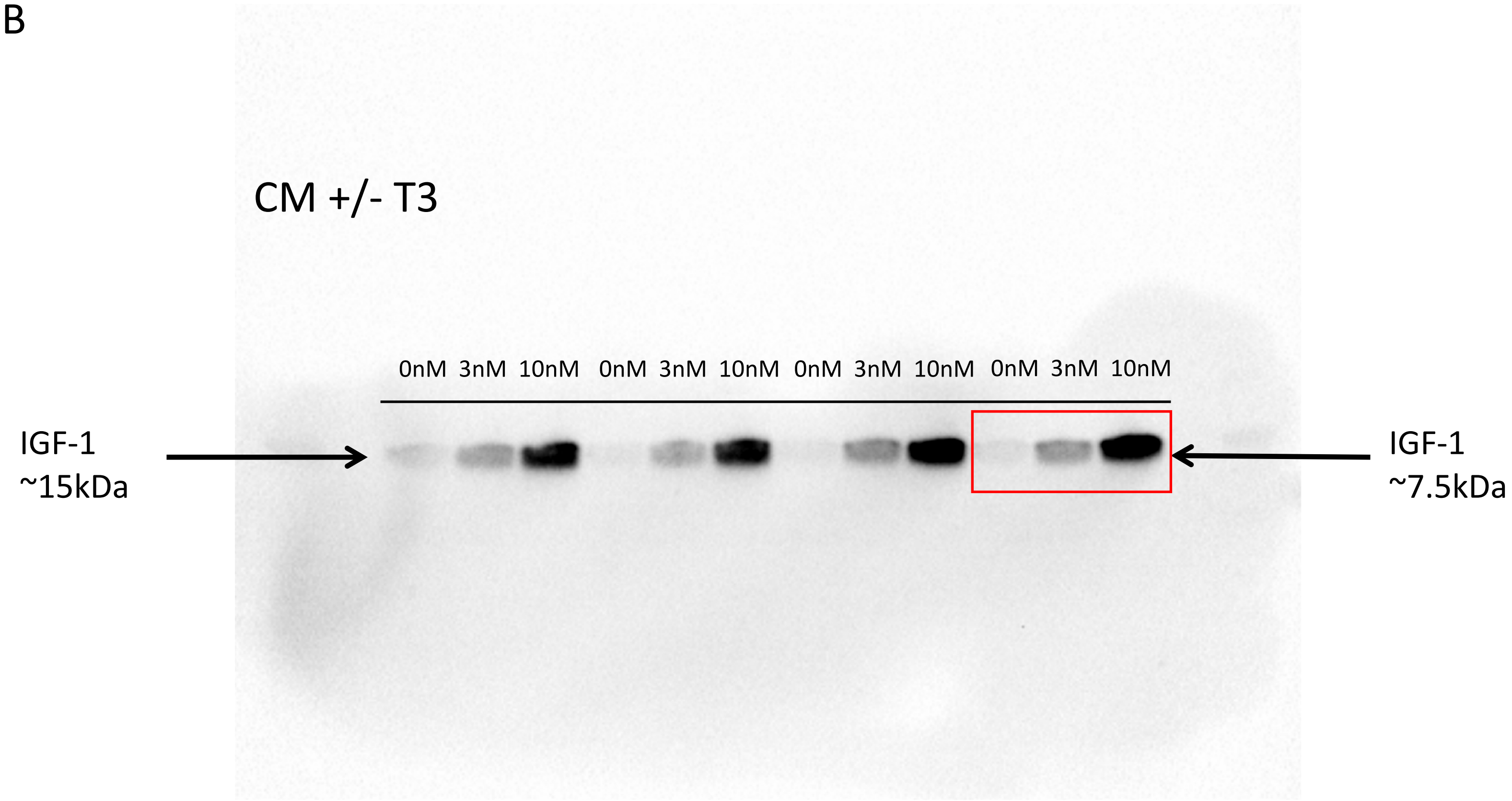
CM +/- T3
0nM 3nM 10nM 0nM 3nM 10nM 0nM 3nM 10nM 0nM 3nM 10nM
IGF-1
~15kDa
IGF-1
~7.5kDa

Figure 4B

CM +/- T3

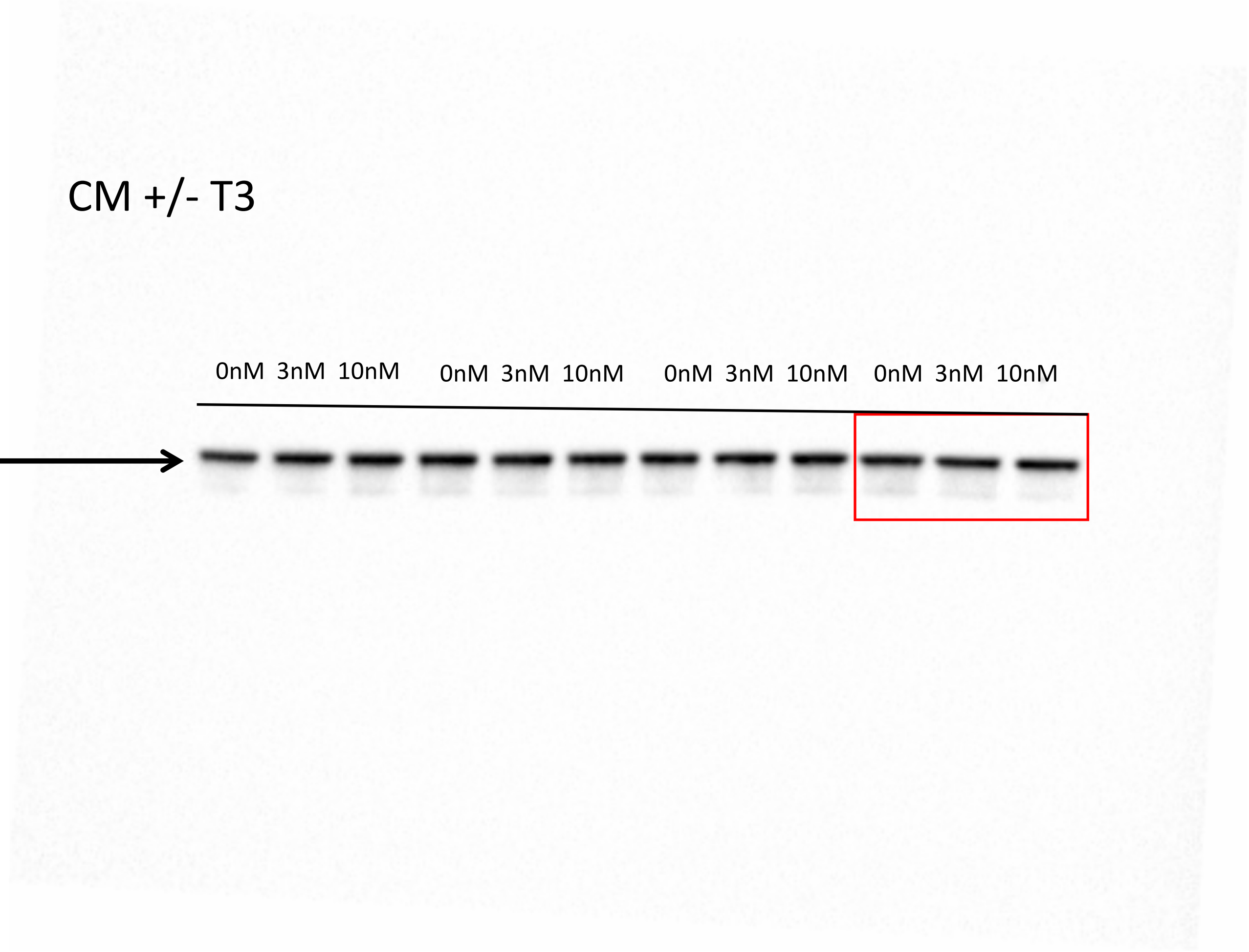

GAPDH
37kDa

Figure 4C

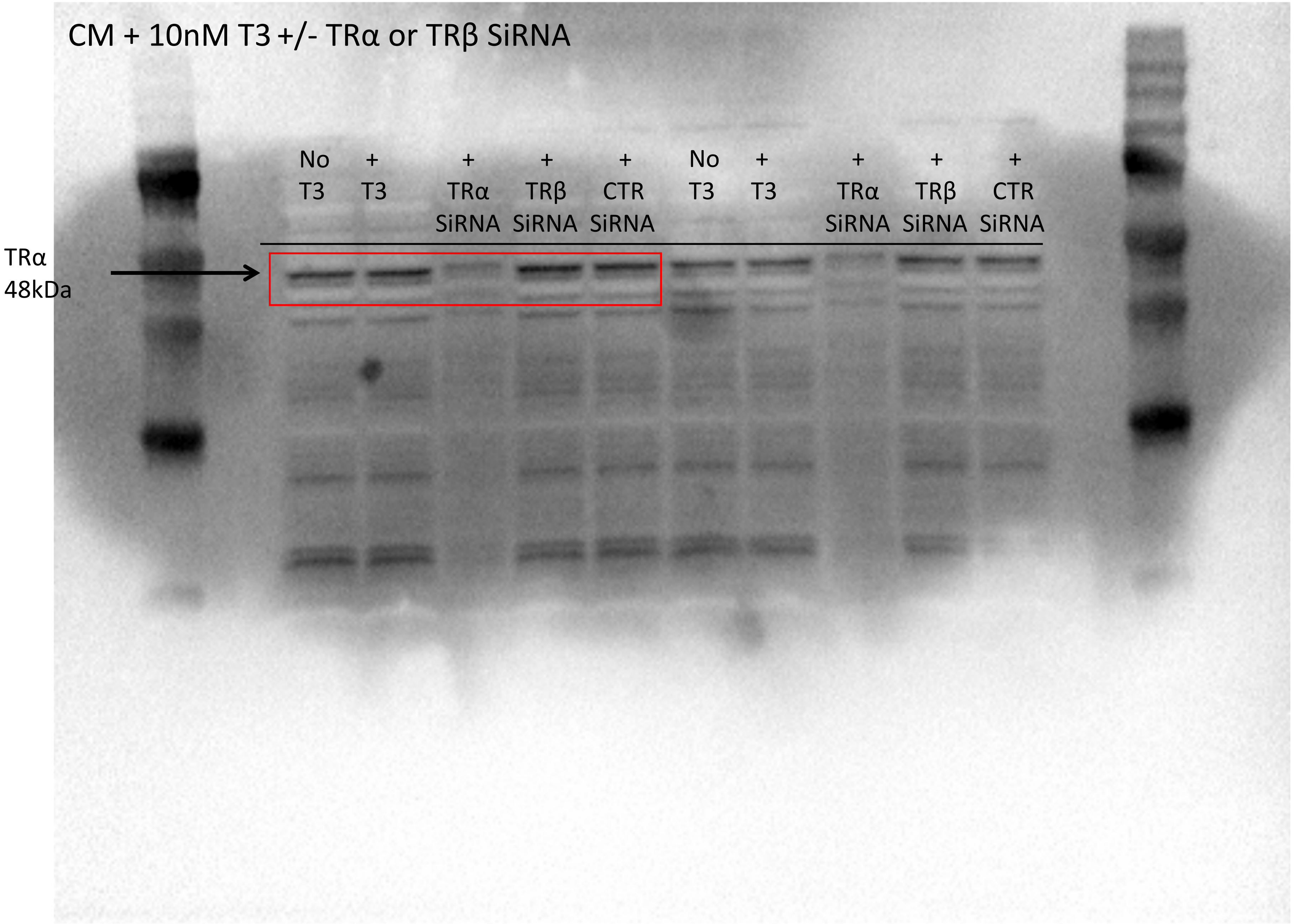

CM + 10nM T3 +/- TRα or TRβ SiRNA
No T3
+ T3
+ TRα SiRNA
+ TRβ SiRNA
+ CTR SiRNA
No T3
+ T3
+ TRα SiRNA
+ TRβ SiRNA
+ CTR SiRNA
TRα 48kDa

Figure 4C

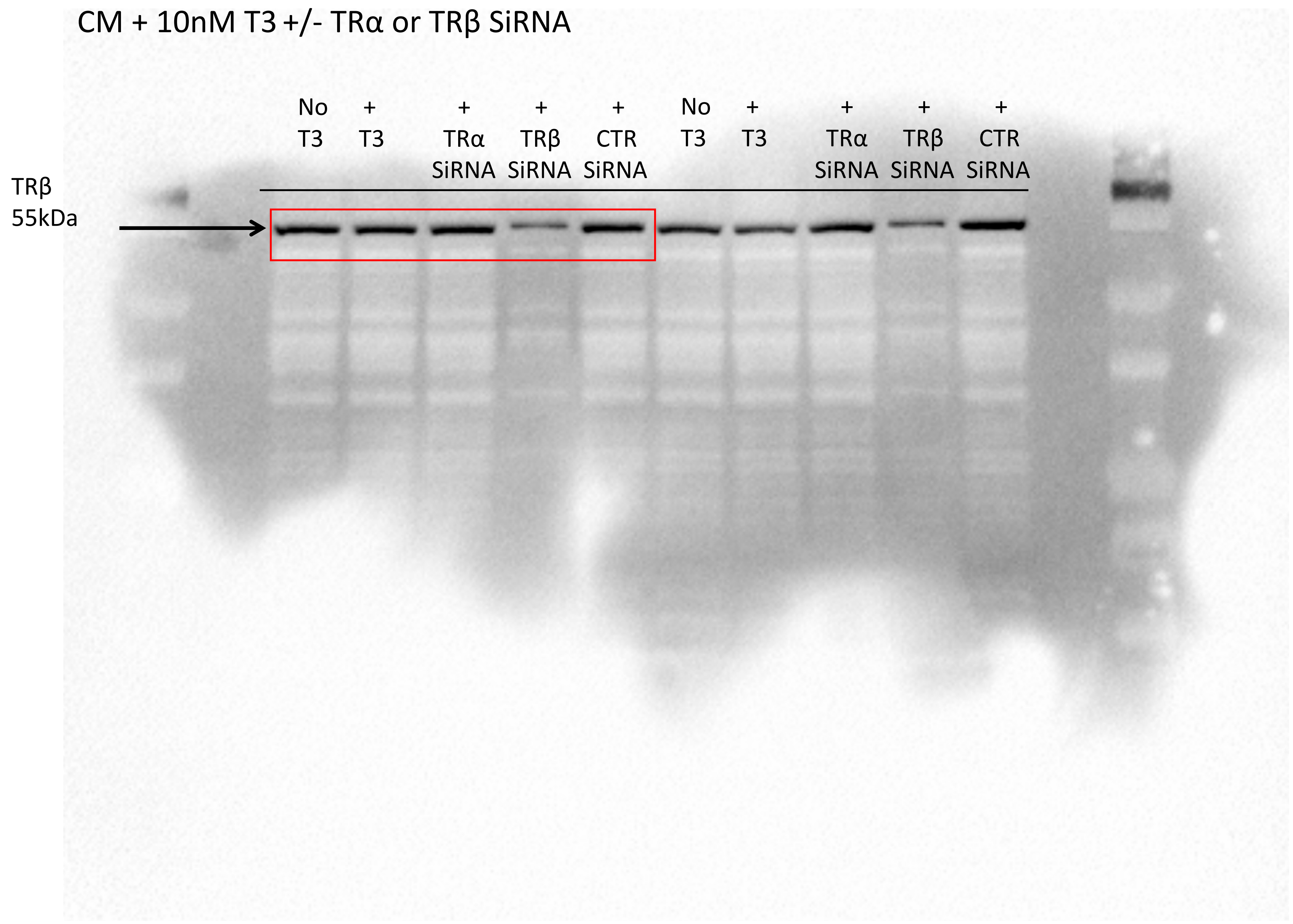
CM + 10nM T3 +/- TRα or TRβ SiRNA
No T3
+ T3
+ TRα SiRNA
+ TRβ SiRNA
+ CTR SiRNA
No T3
+ T3
+ TRα SiRNA
+ TRβ SiRNA
+ CTR SiRNA
TRβ 55kDa

Figure 4C

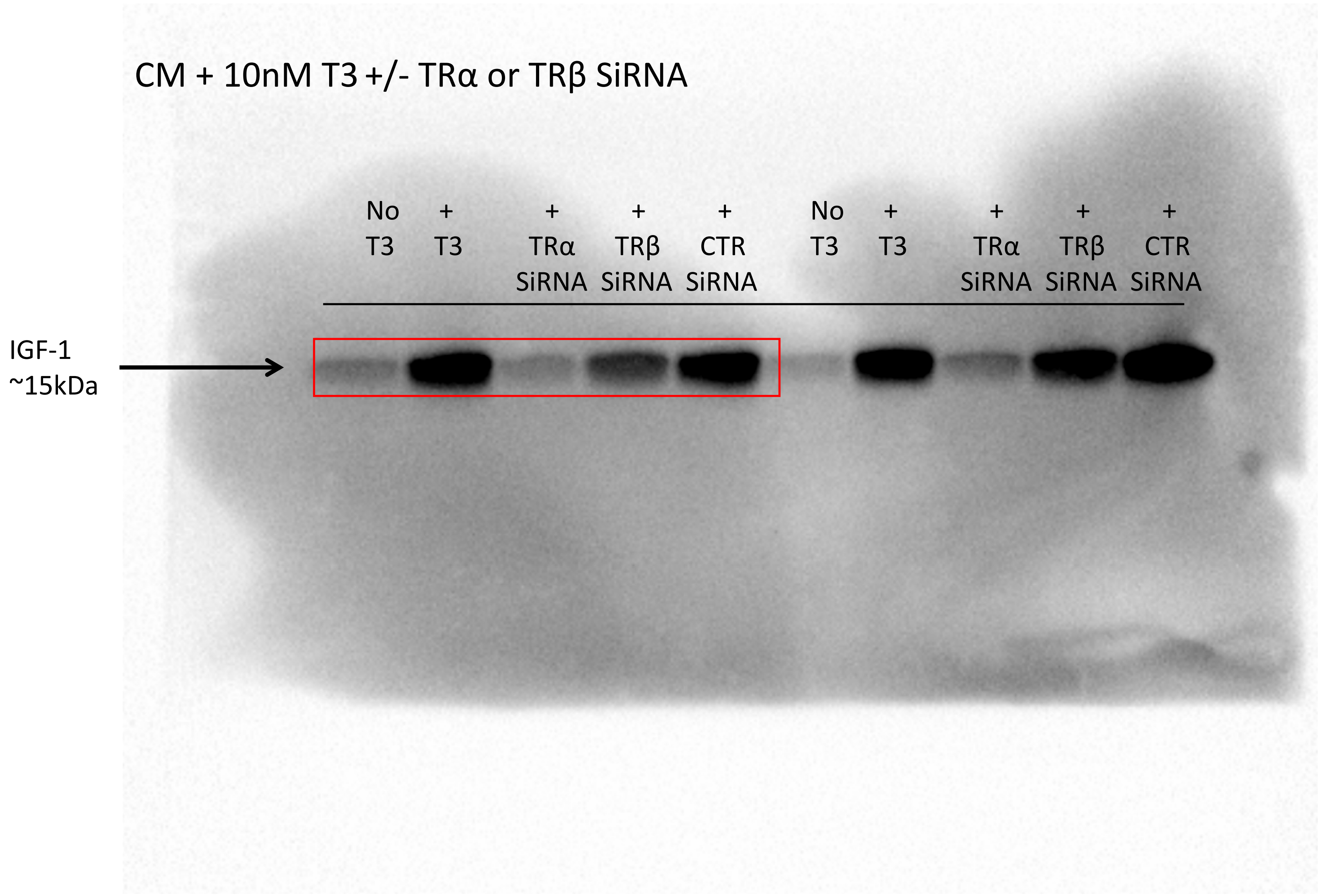
CM + 10nM T3 +/- TRα or TRβ SiRNA
No T3
+ T3
+ TRα SiRNA
+ TRβ SiRNA
+ CTR SiRNA
No T3
+ T3
+ TRα SiRNA
+ TRβ SiRNA
+ CTR SiRNA
IGF-1
~15kDa

Figure 4C

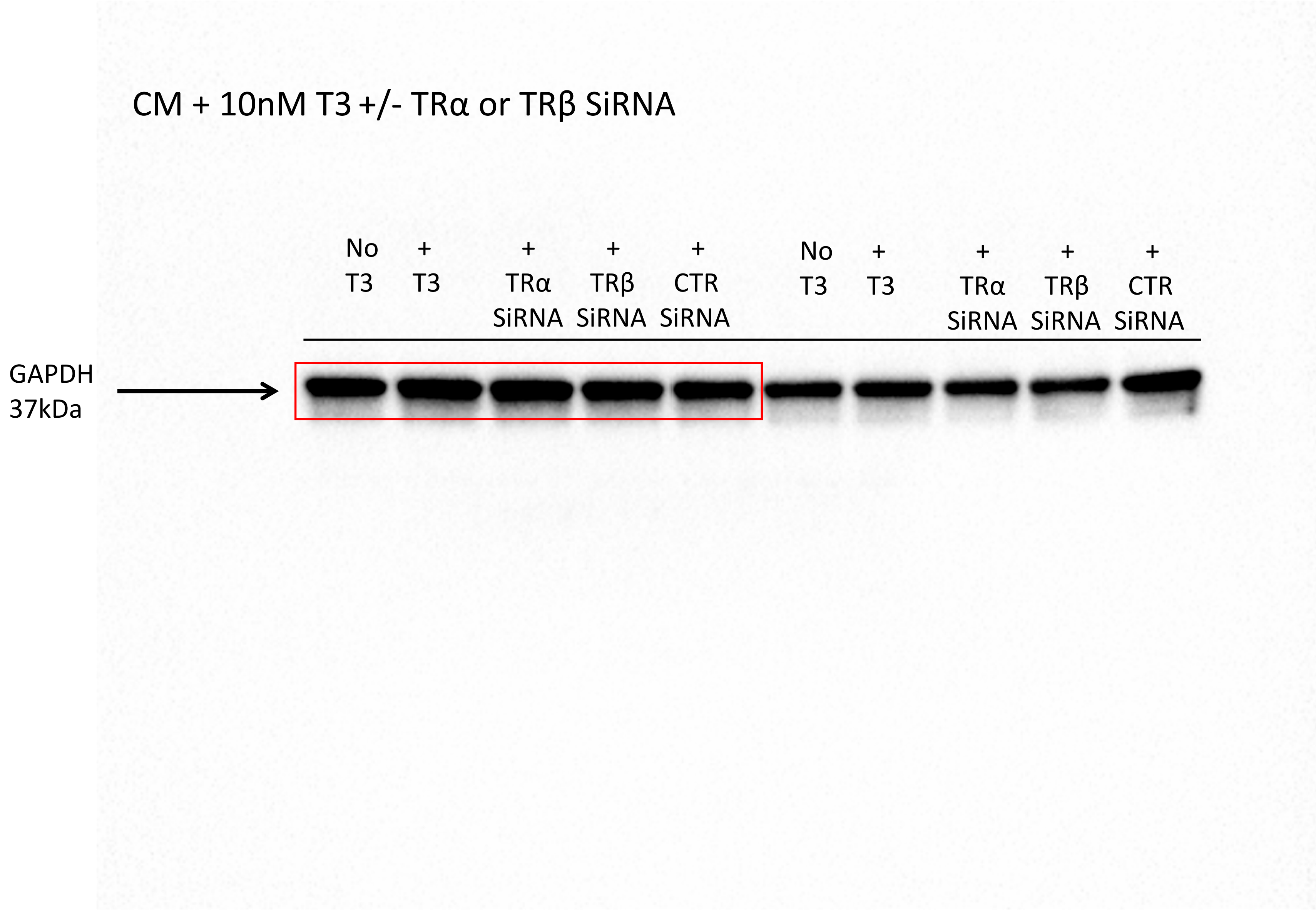
CM + 10nM T3 +/- TRα or TRβ SiRNA
No T3
+ T3
+ TRα SiRNA
+ TRβ SiRNA
+ CTR SiRNA
No T3
+ T3
+ TRα SiRNA
+ TRβ SiRNA
+ CTR SiRNA
GAPDH
37kDa

Figure 4D

CM + 10nM T3 +/- IGF-1 SiRNA

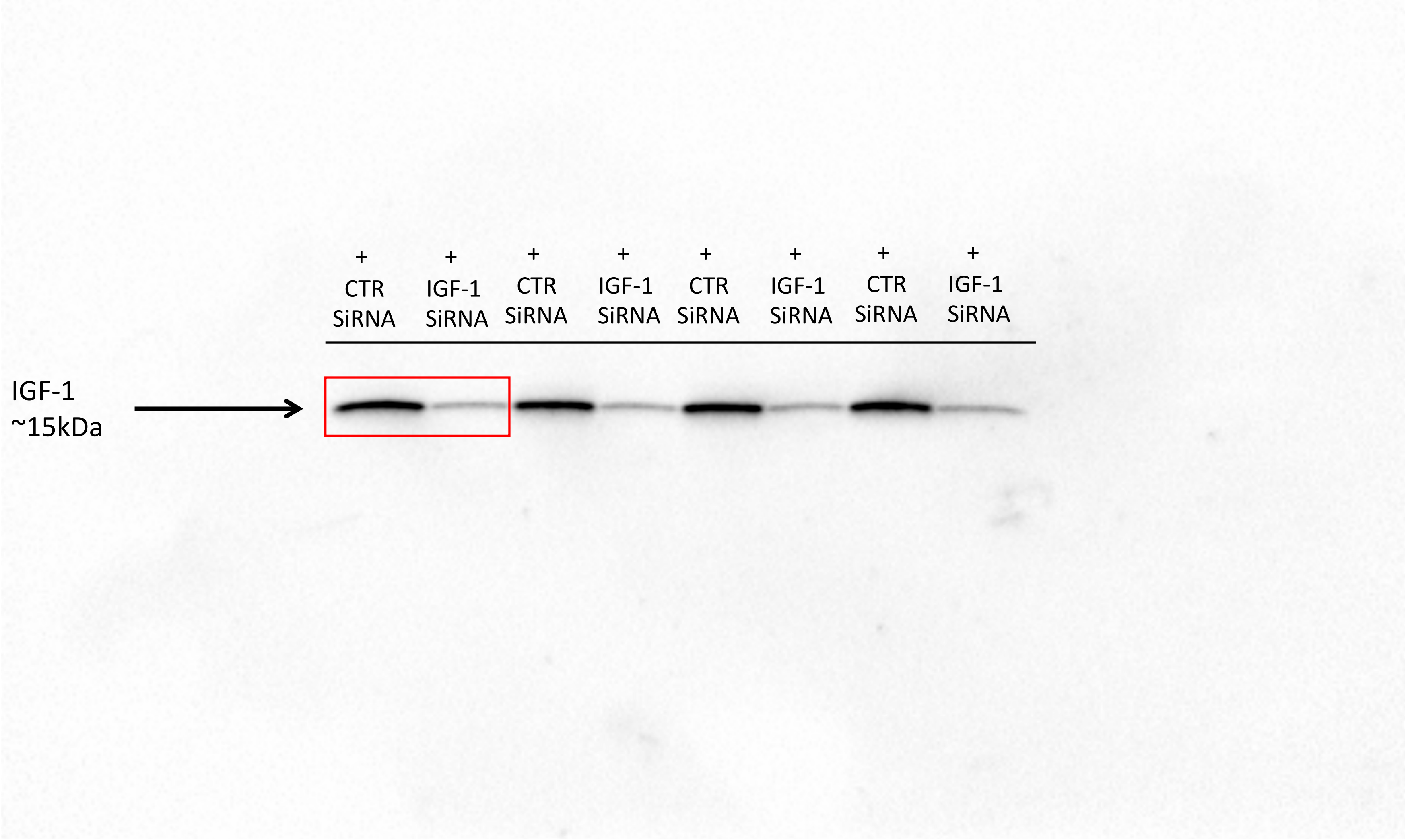

Figure 4D

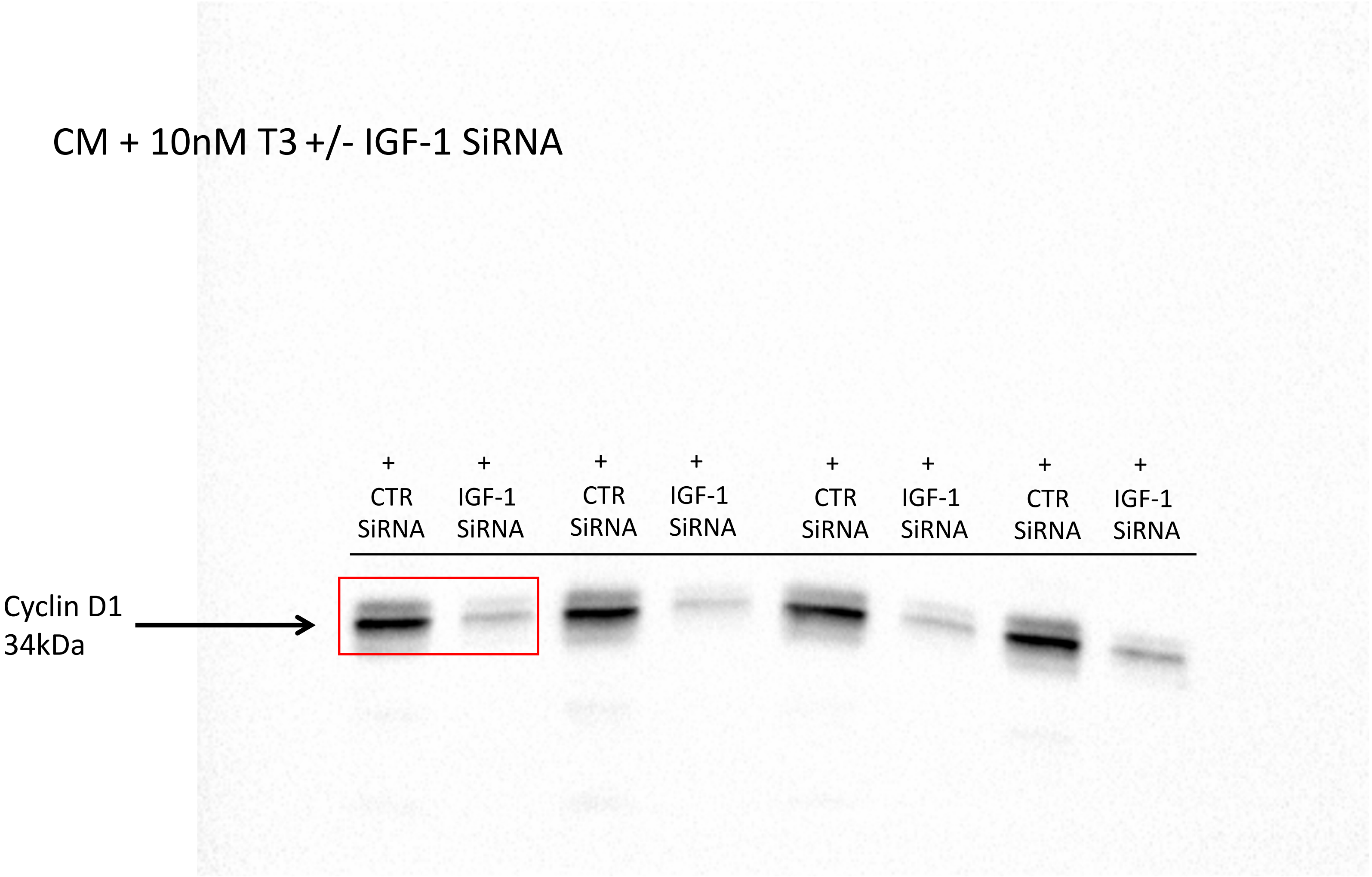
CM + 10nM T3 +/- IGF-1 SiRNA
+ CTR SiRNA
+ IGF-1 SiRNA
+ CTR SiRNA
+ IGF-1 SiRNA
+ CTR SiRNA
+ IGF-1 SiRNA
+ CTR SiRNA
+ IGF-1 SiRNA
Cyclin D1
34kDa

Figure 4D

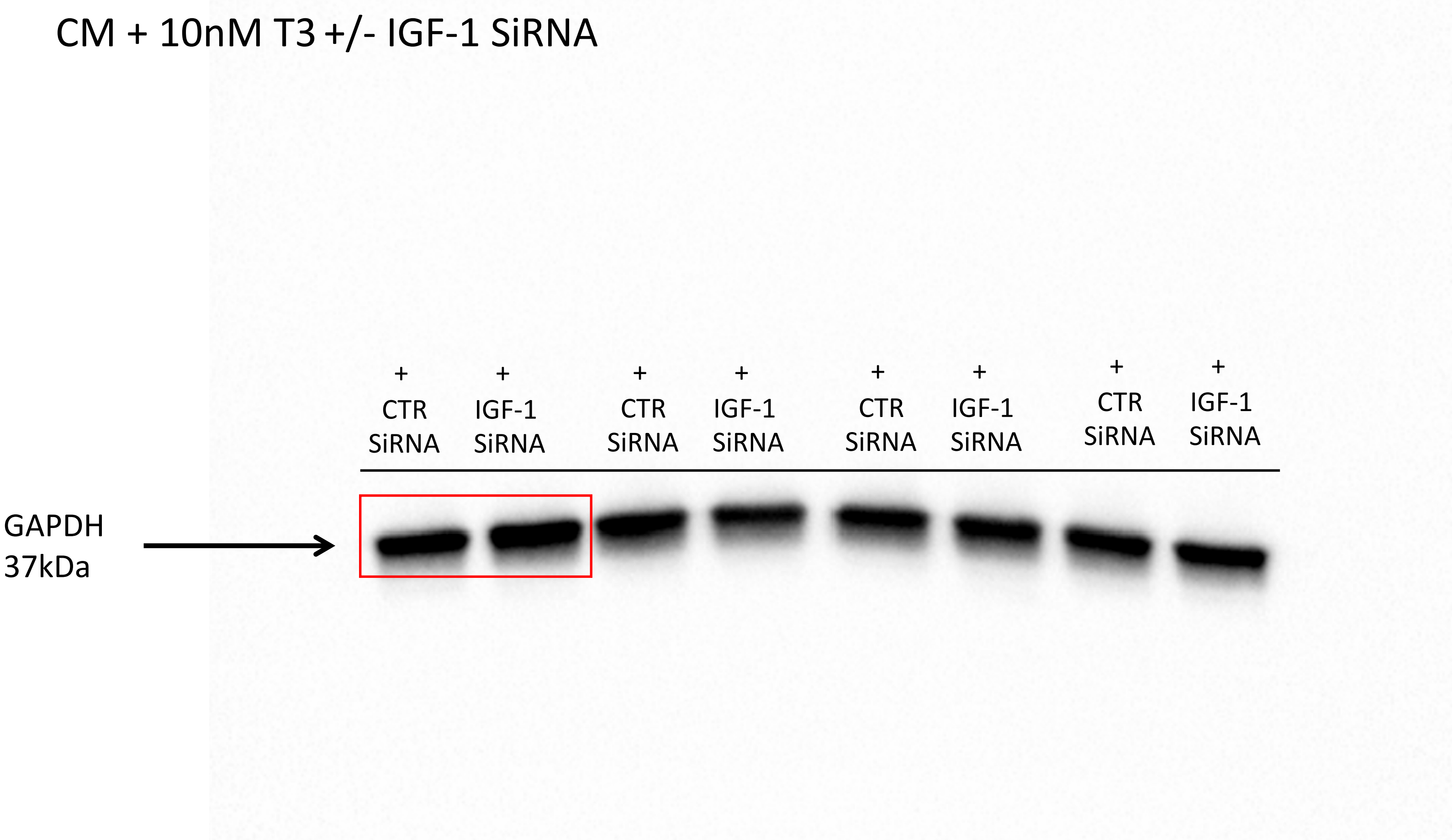

CM + 10nM T3 +/- IGF-1 SiRNA
+ CTR SiRNA
+ IGF-1 SiRNA
+ CTR SiRNA
+ IGF-1 SiRNA
+ CTR SiRNA
+ IGF-1 SiRNA
+ CTR SiRNA
+ IGF-1 SiRNA
GAPDH
37kDa

Figure 5A

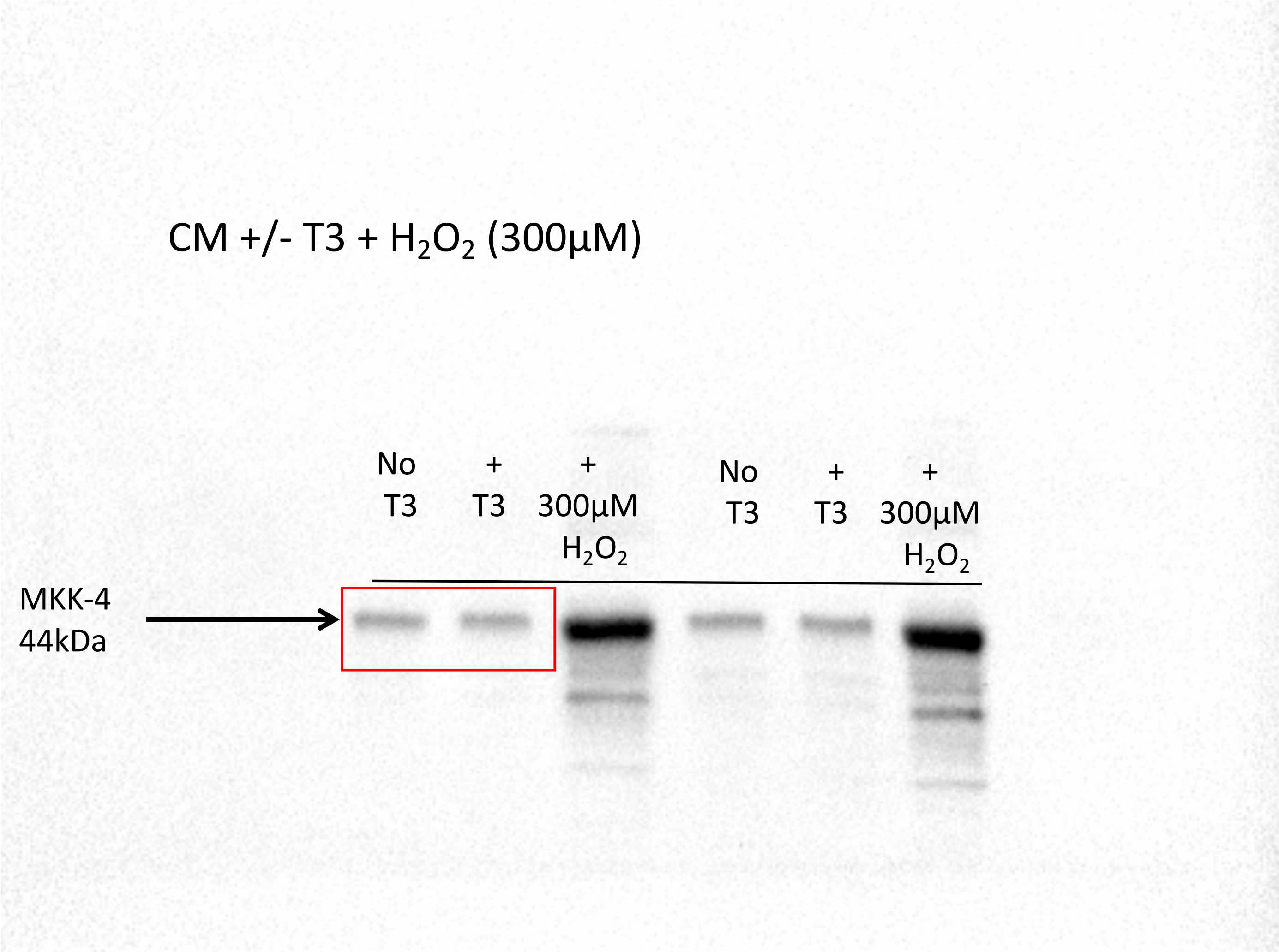

CM +/- T3 + H_2O_2 (300µM)
No T3
+ T3
+ 300µM H_2O_2
No T3
+ T3
+ 300µM H_2O_2
MKK-4
44kDa

Figure 5A

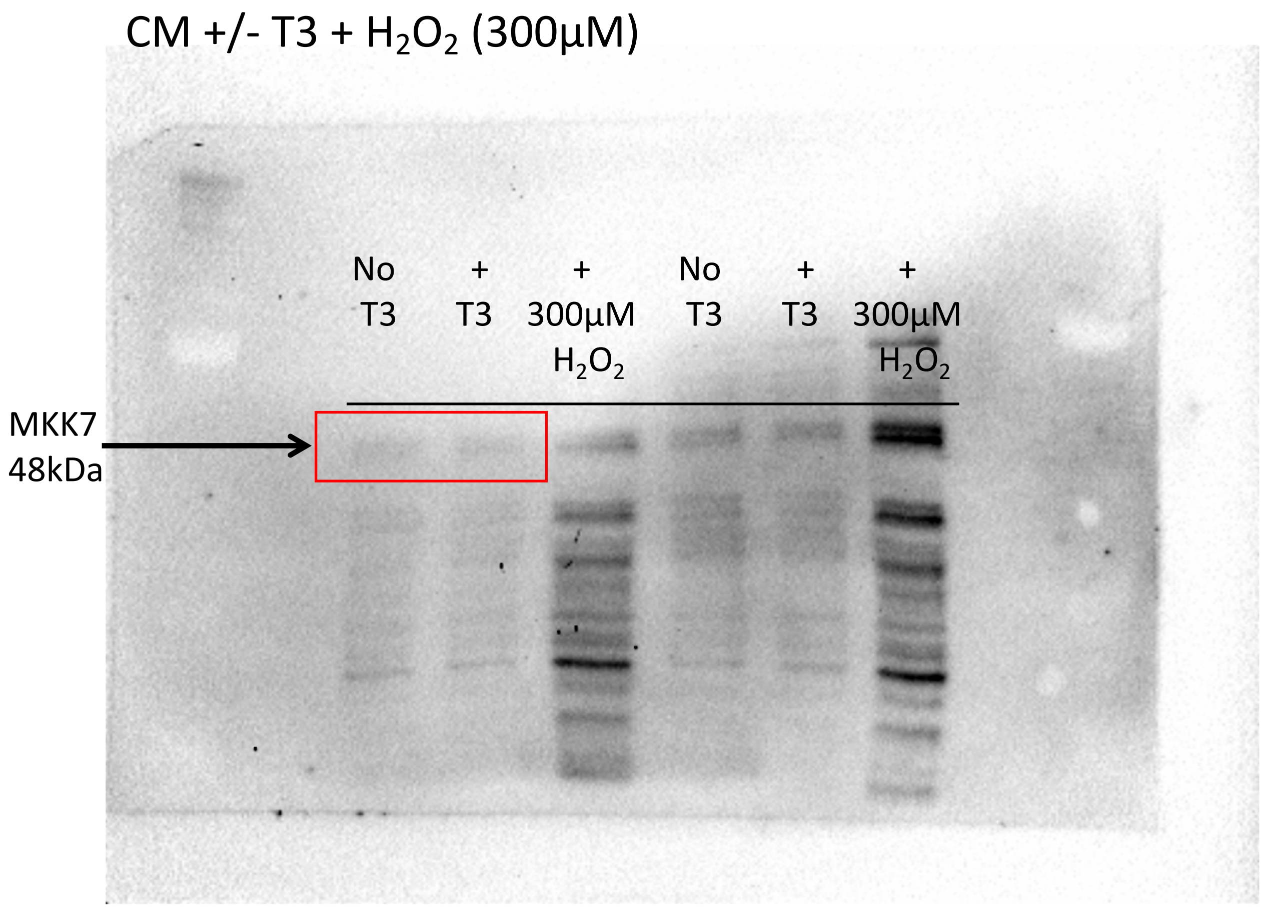

CM +/- T3 + H_2O_2 (300μM)
No T3
+ T3
+ 300μM H_2O_2
No T3
+ T3
+ 300μM H_2O_2
MKK7 48kDa

Figure 5A

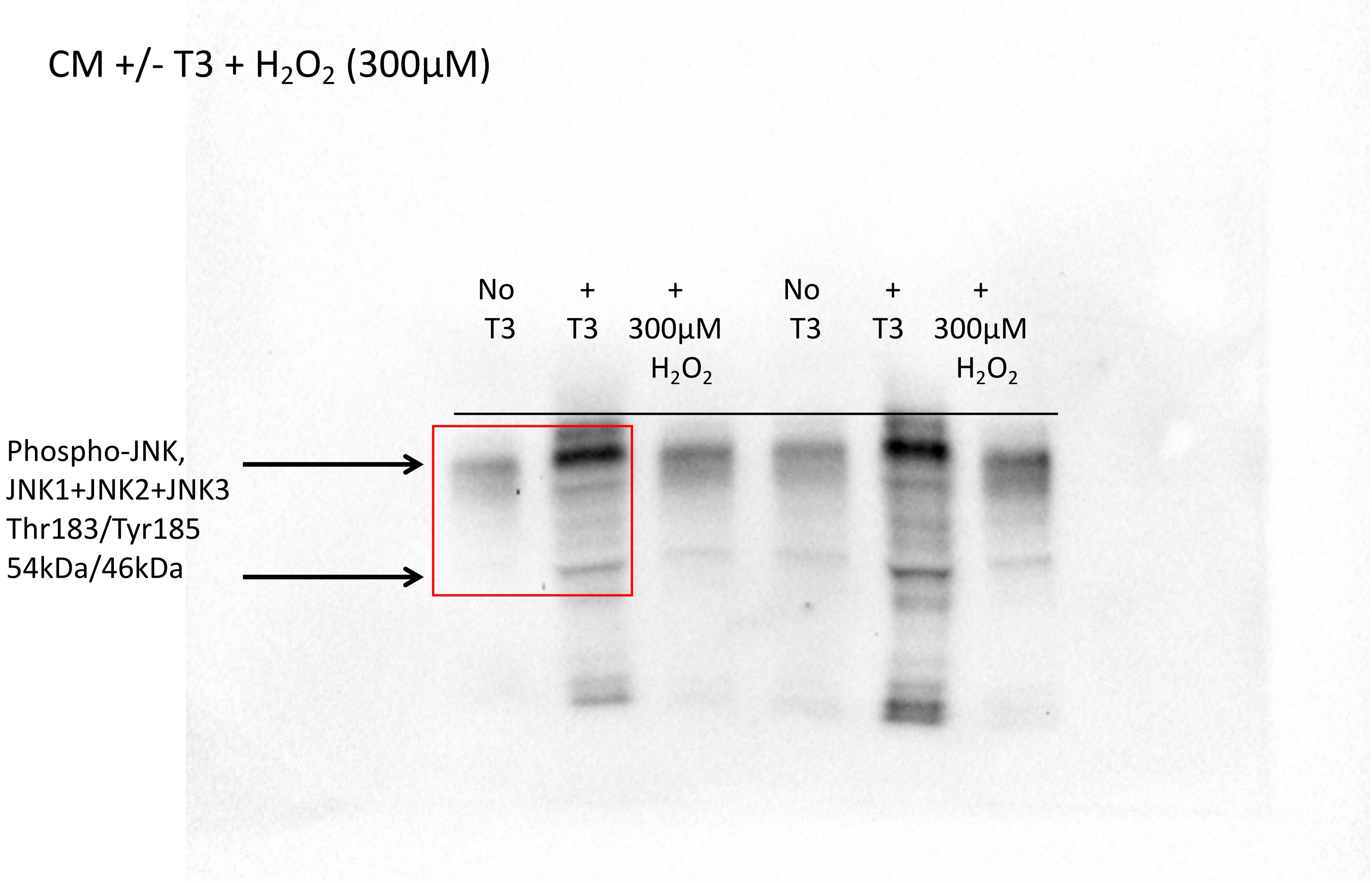
CM +/- T3 + H_2O_2 (300µM)
No T3
+ T3
+ 300µM H_2O_2
No T3
+ T3
+ 300µM H_2O_2
Phospho-JNK,
JNK1+JNK2+JNK3
Thr183/Tyr185
54kDa/46kDa

Figure 5A

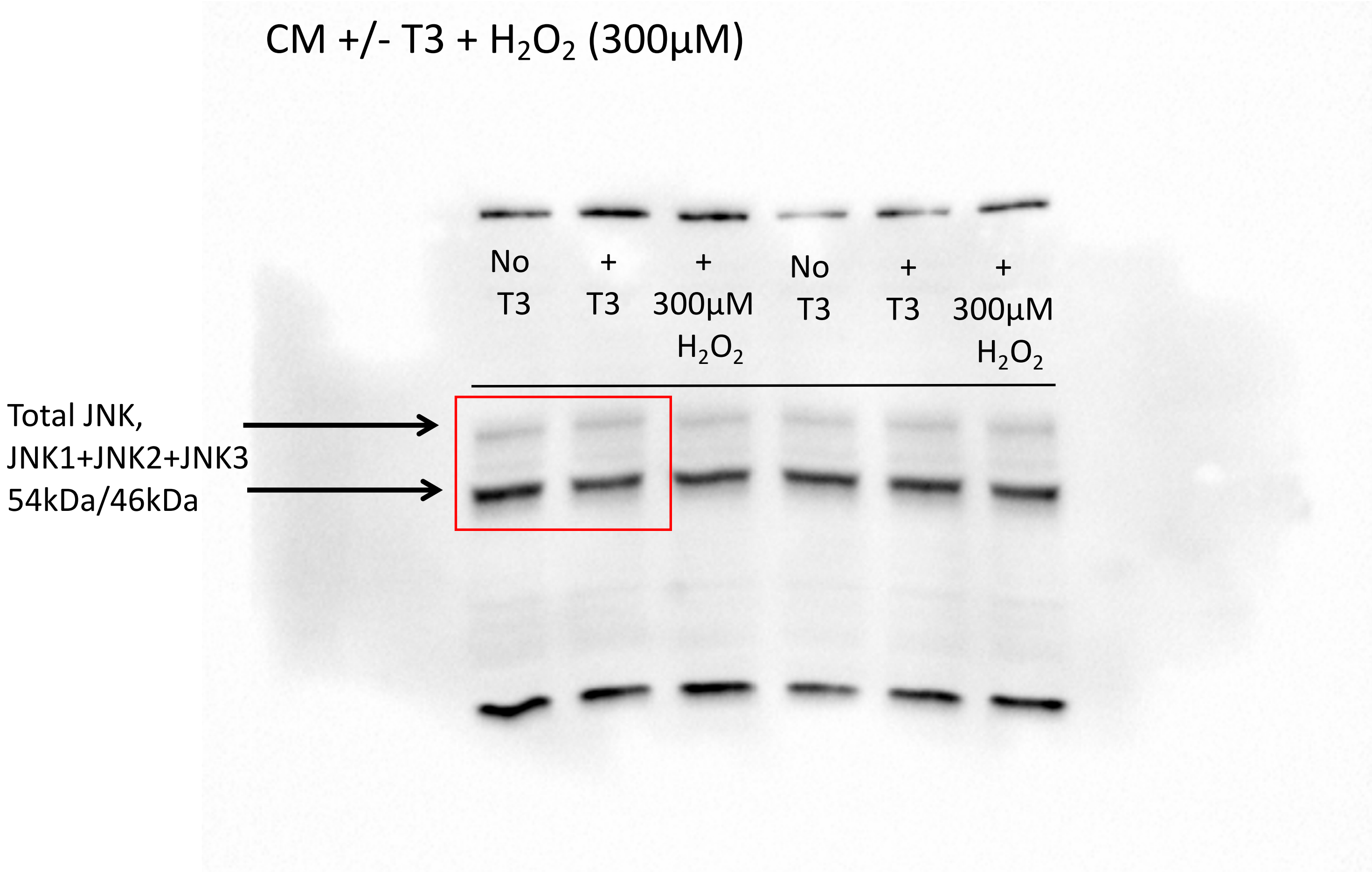

CM +/- T3 + H_2O_2 (300µM)
No T3
+ T3
+ 300µM H_2O_2
No T3
+ T3
+ 300µM H_2O_2
Total JNK,
JNK1+JNK2+JNK3
54kDa/46kDa

Figure 5A

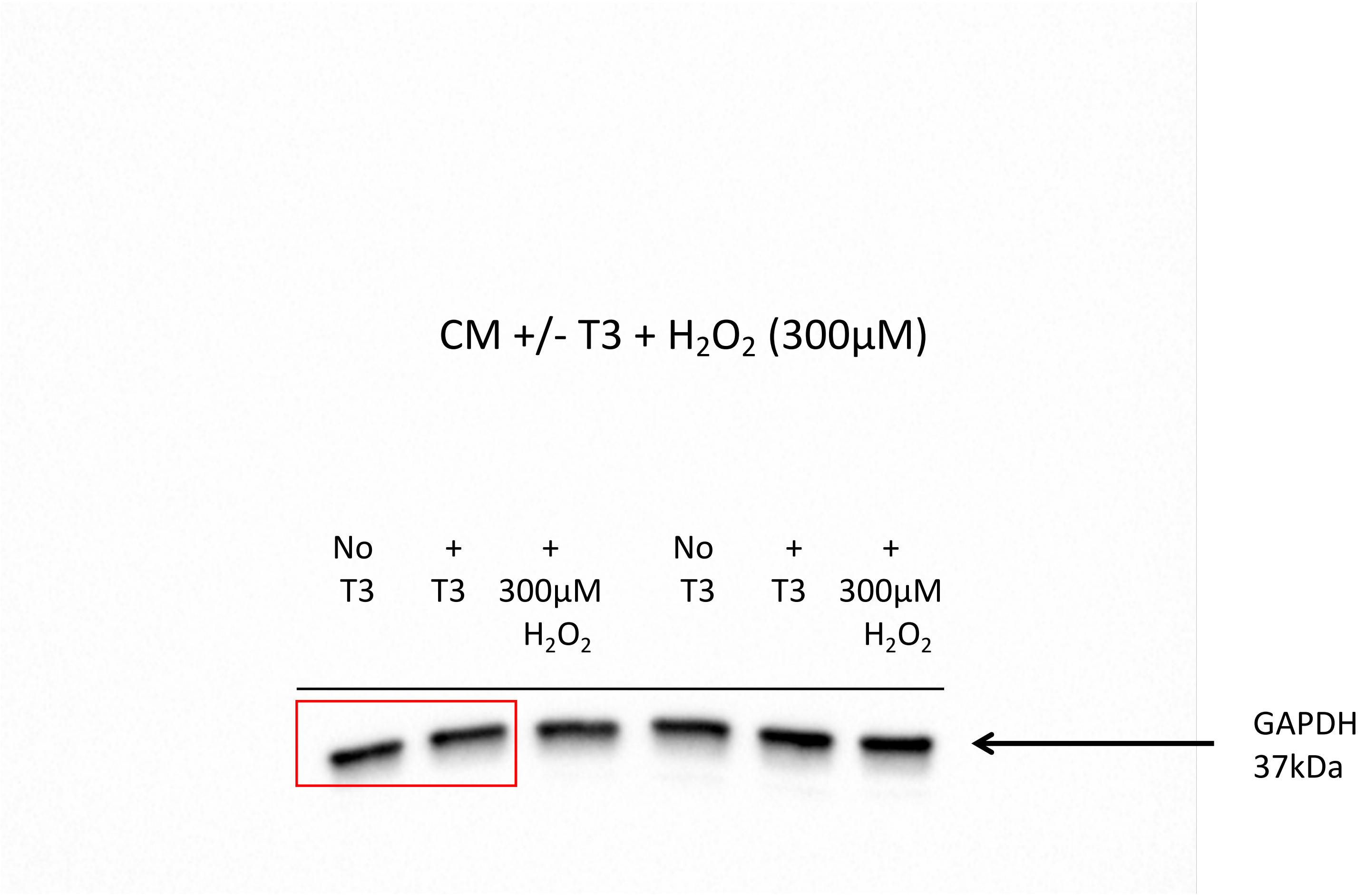

CM +/- T3 + H_2O_2 (300µM)
No T3
+ T3
+ 300µM H_2O_2
No T3
+ T3
+ 300µM H_2O_2
GAPDH 37kDa

Figure 5B

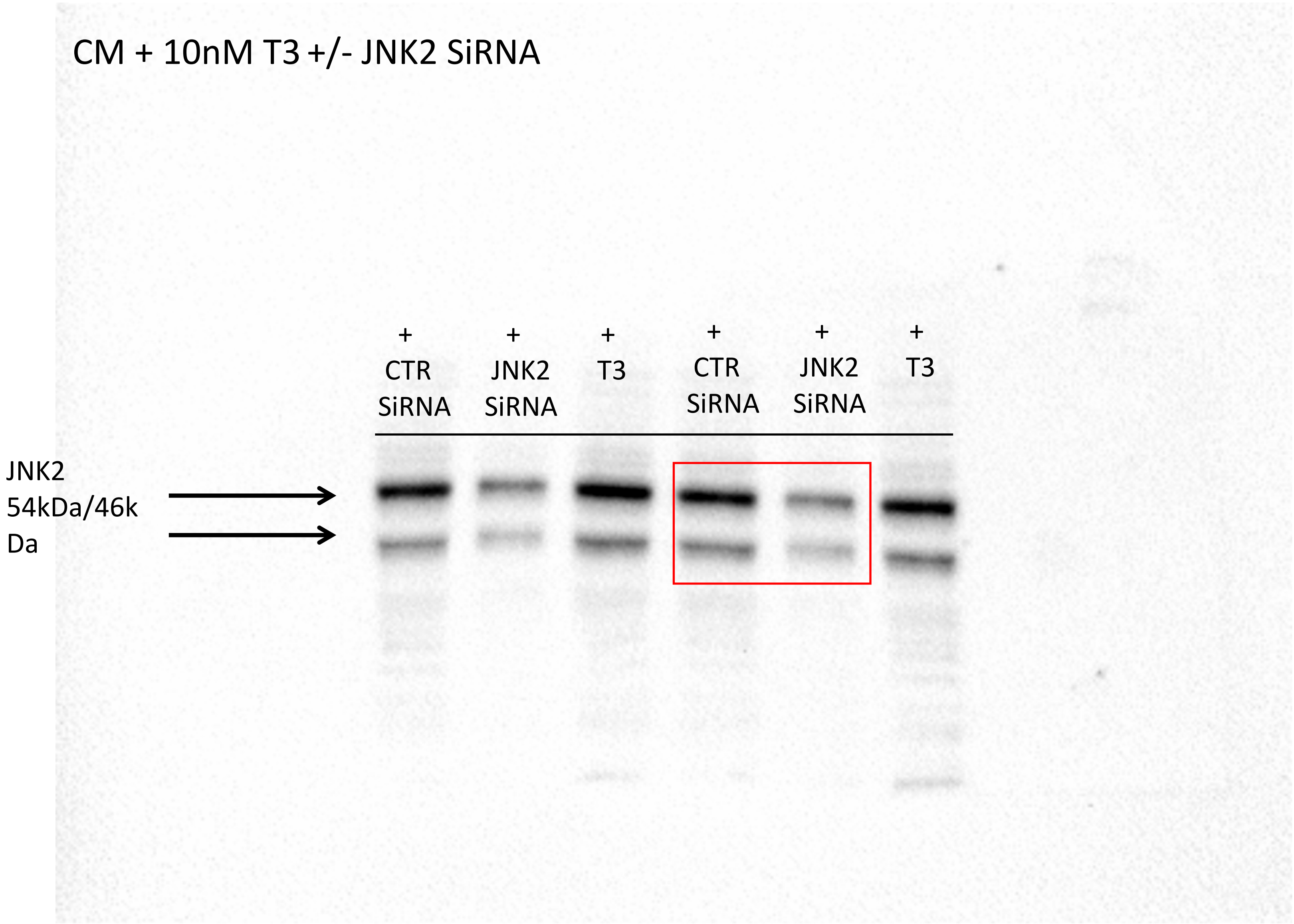
CM + 10nM T3 +/- JNK2 SiRNA
+
CTR
SiRNA
+
JNK2
SiRNA
+
T3
+
CTR
SiRNA
+
JNK2
SiRNA
+
T3
JNK2
54kDa/46k
Da

Figure 5B

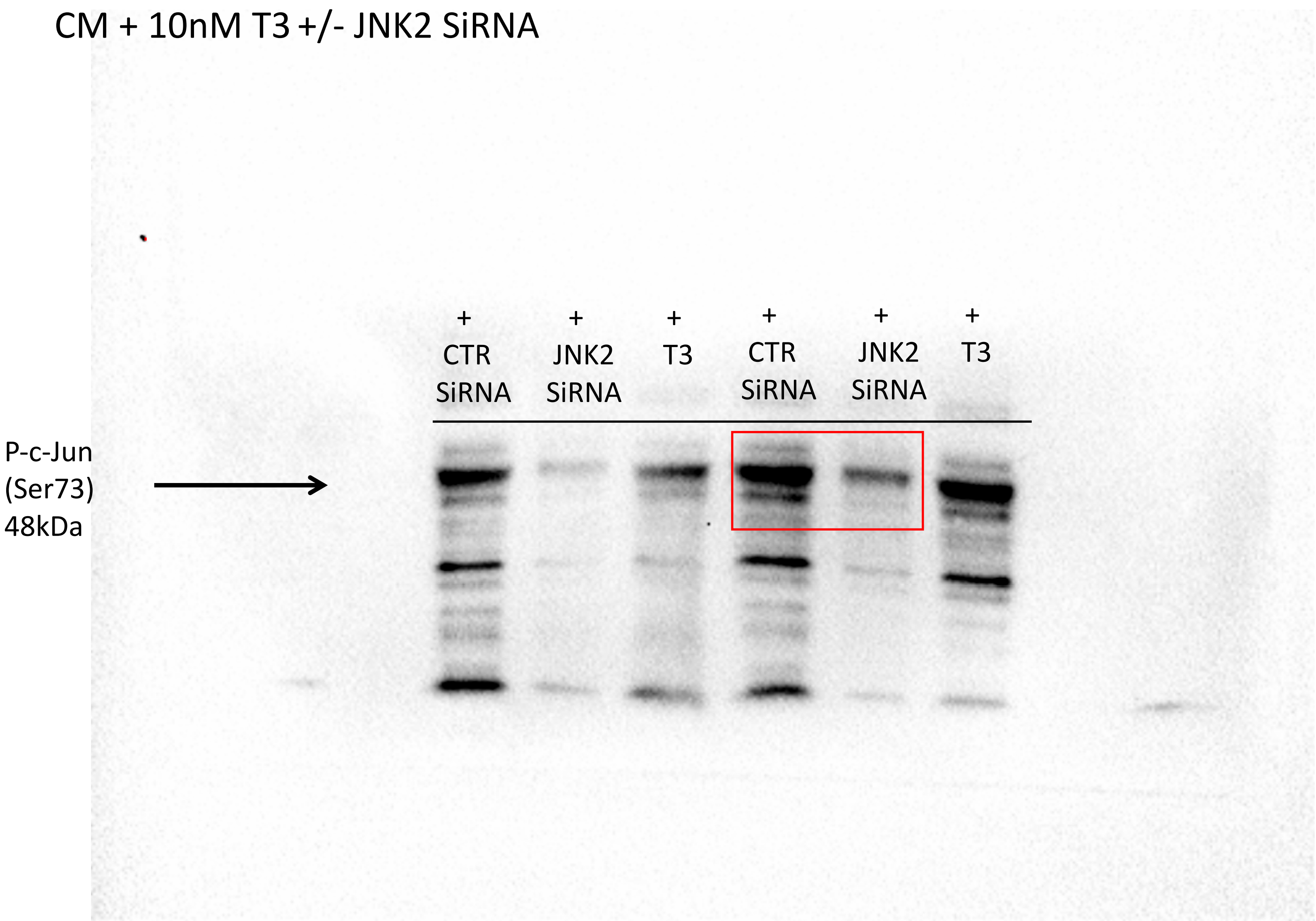

CM + 10nM T3 +/- JNK2 SiRNA
+ CTR SiRNA
+ JNK2 SiRNA
+ T3
+ CTR SiRNA
+ JNK2 SiRNA
+ T3
P-c-Jun
(Ser73)
48kDa

Figure 5B

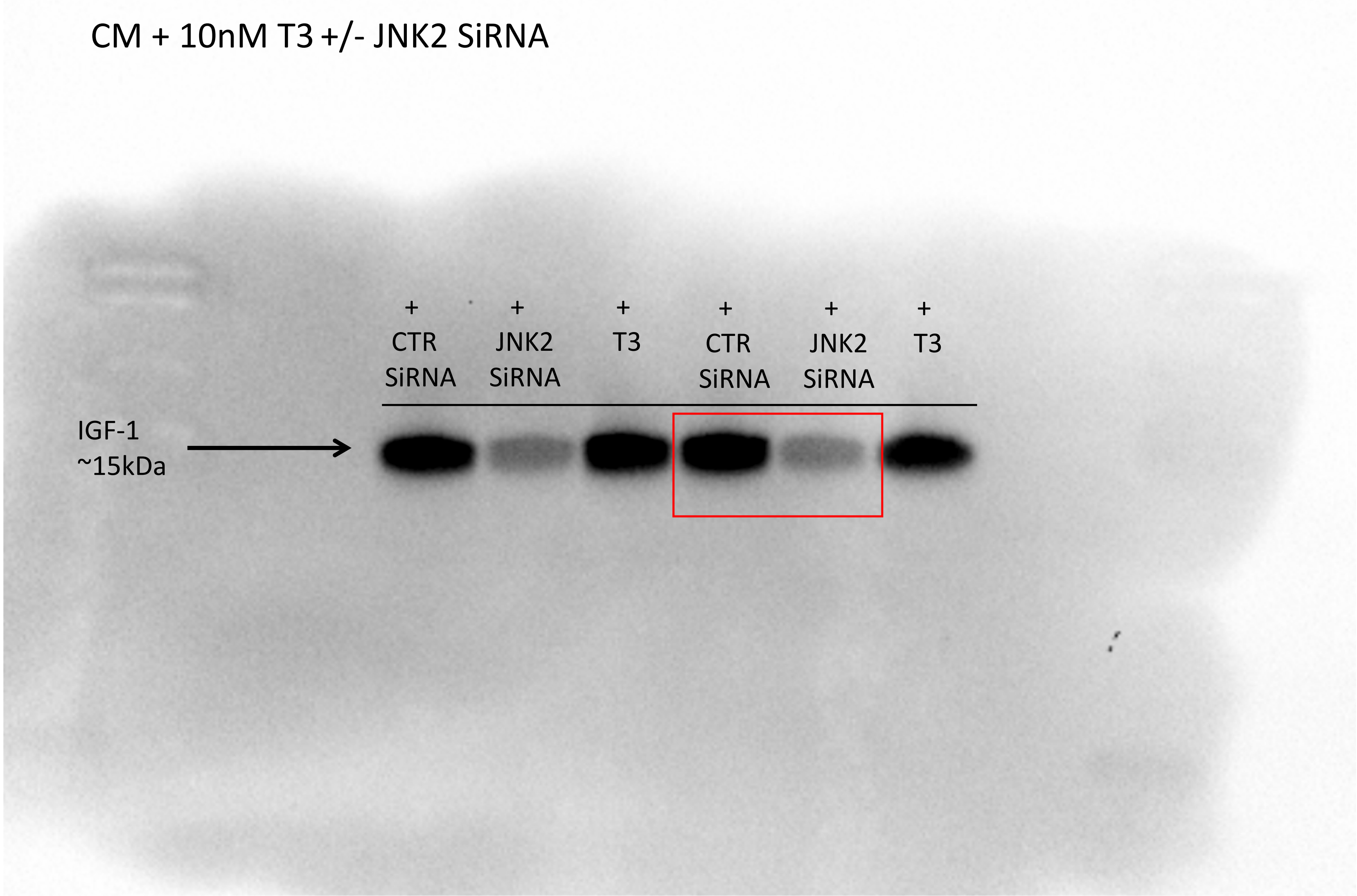
CM + 10nM T3 +/- JNK2 SiRNA
+ CTR SiRNA
+ JNK2 SiRNA
+ T3
+ CTR SiRNA
+ JNK2 SiRNA
+ T3
IGF-1
~15kDa

Figure 5B

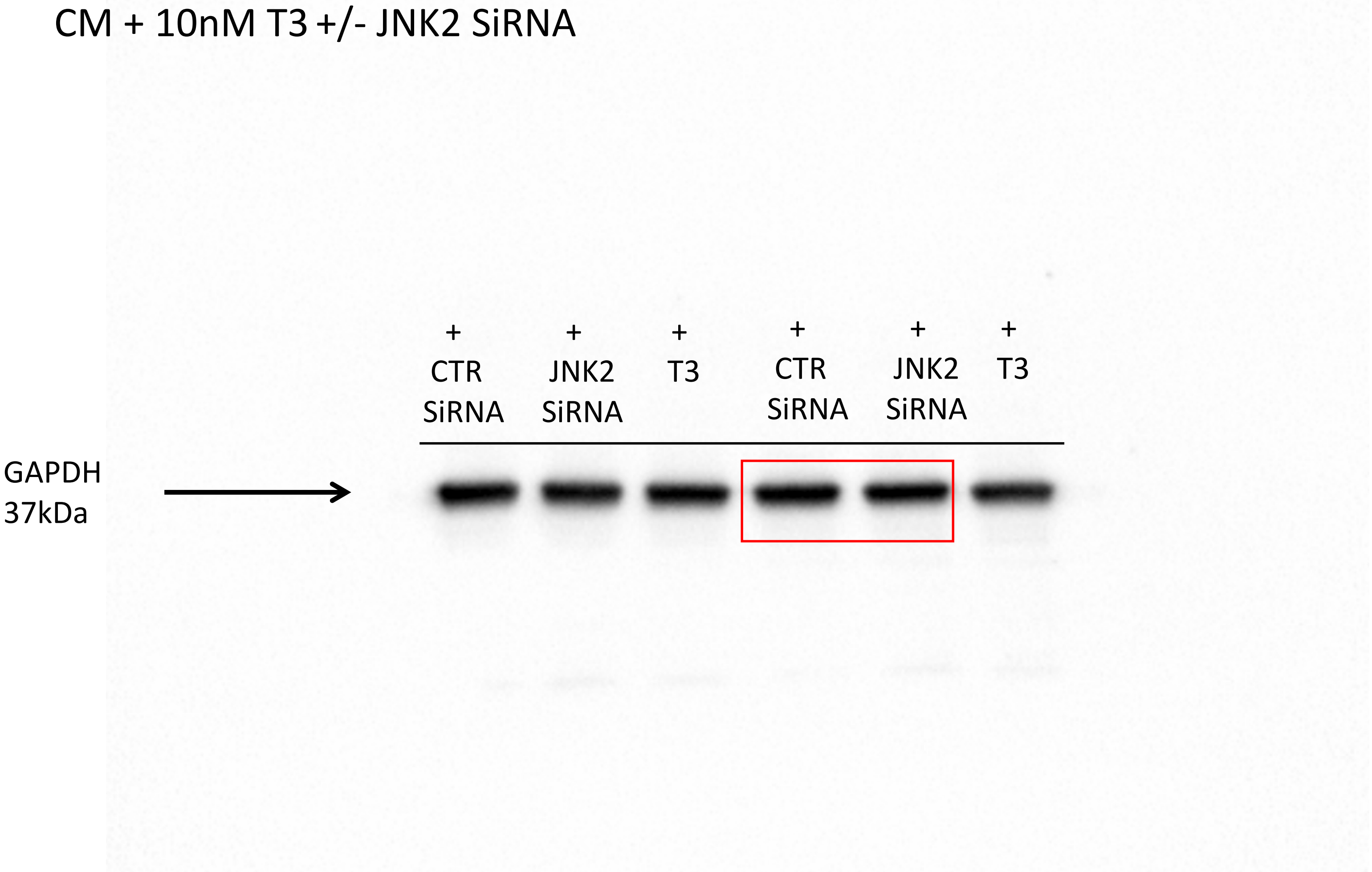

CM + 10nM T3 +/- JNK2 SiRNA
+ CTR SiRNA
+ JNK2 SiRNA
+ T3
+ CTR SiRNA
+ JNK2 SiRNA
+ T3
GAPDH 37kDa

Figure 5C

CM + 10nM T3 IB: JNK-2 α

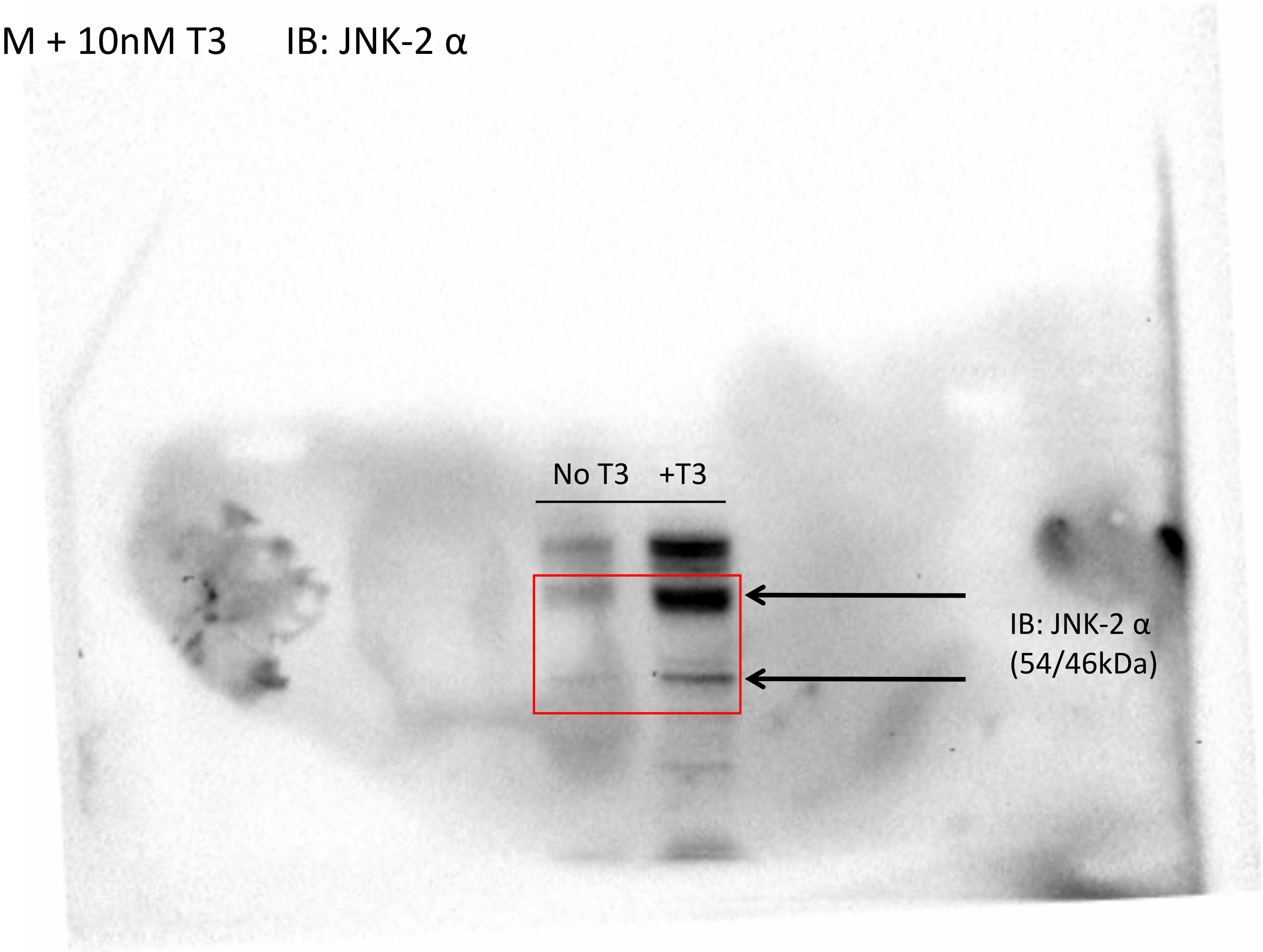

Figure 5C

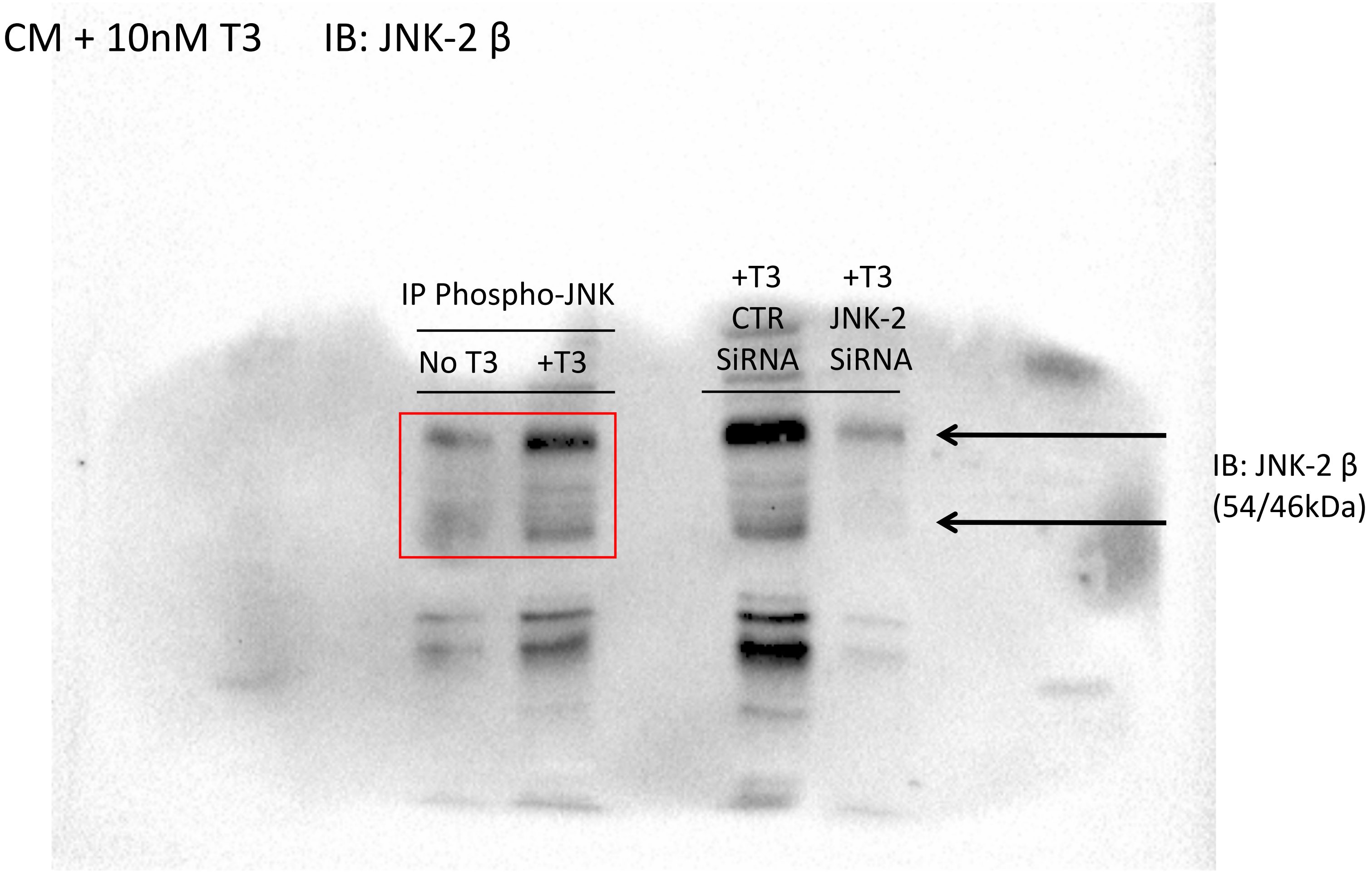

CM + 10nM T3
IB: JNK-2 β
IP Phospho-JNK
No T3
+T3
+T3
CTR
SiRNA
+T3
JNK-2
SiRNA
IB: JNK-2 β
(54/46kDa)

Figure 5D

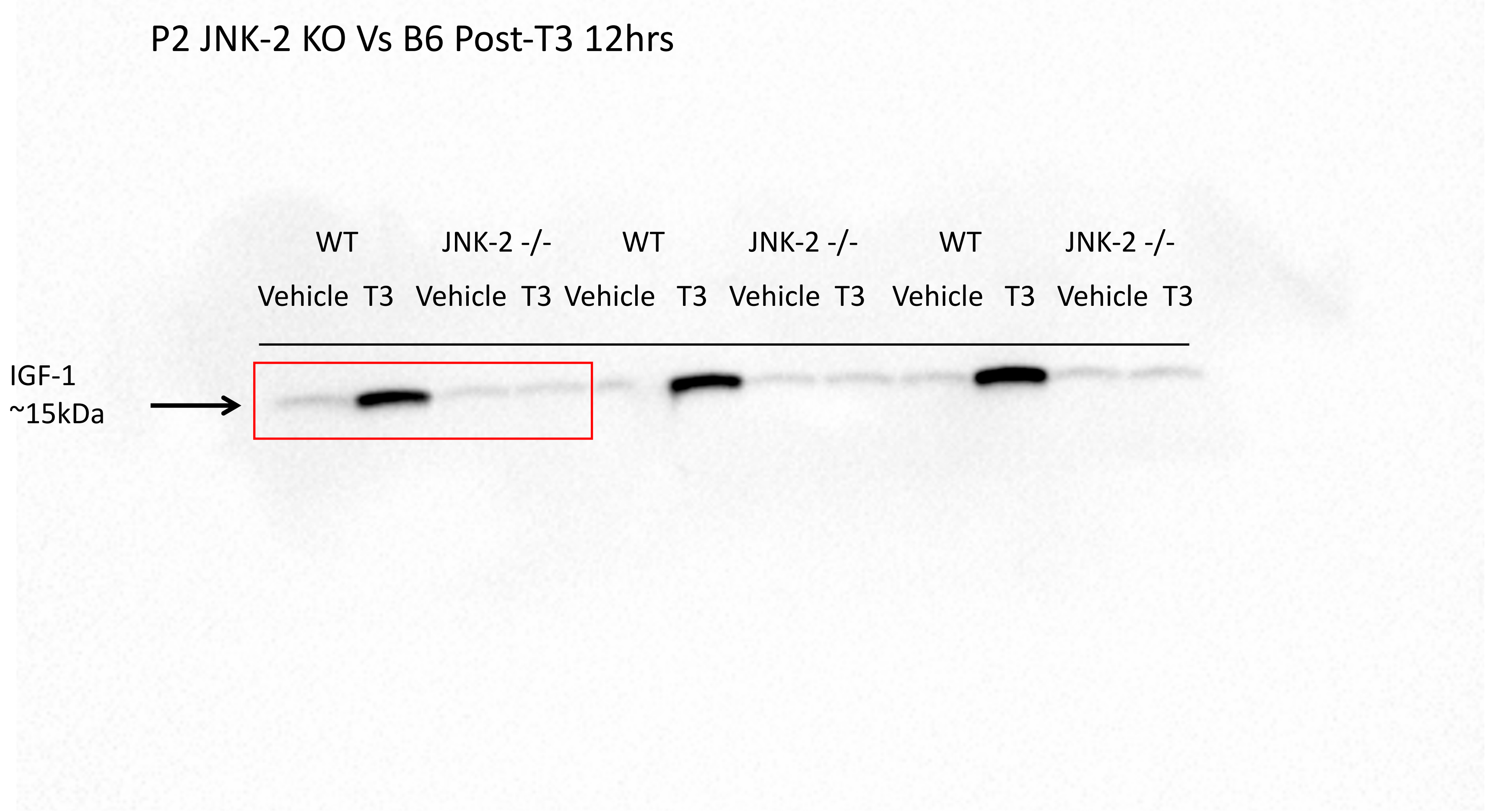
P2 JNK-2 KO Vs B6 Post-T3 12hrs
WT
JNK-2 -/-
WT
JNK-2 -/-
WT
JNK-2 -/-
Vehicle T3 Vehicle T3 Vehicle T3 Vehicle T3 Vehicle T3 Vehicle T3
IGF-1
~15kDa

Figure 5D

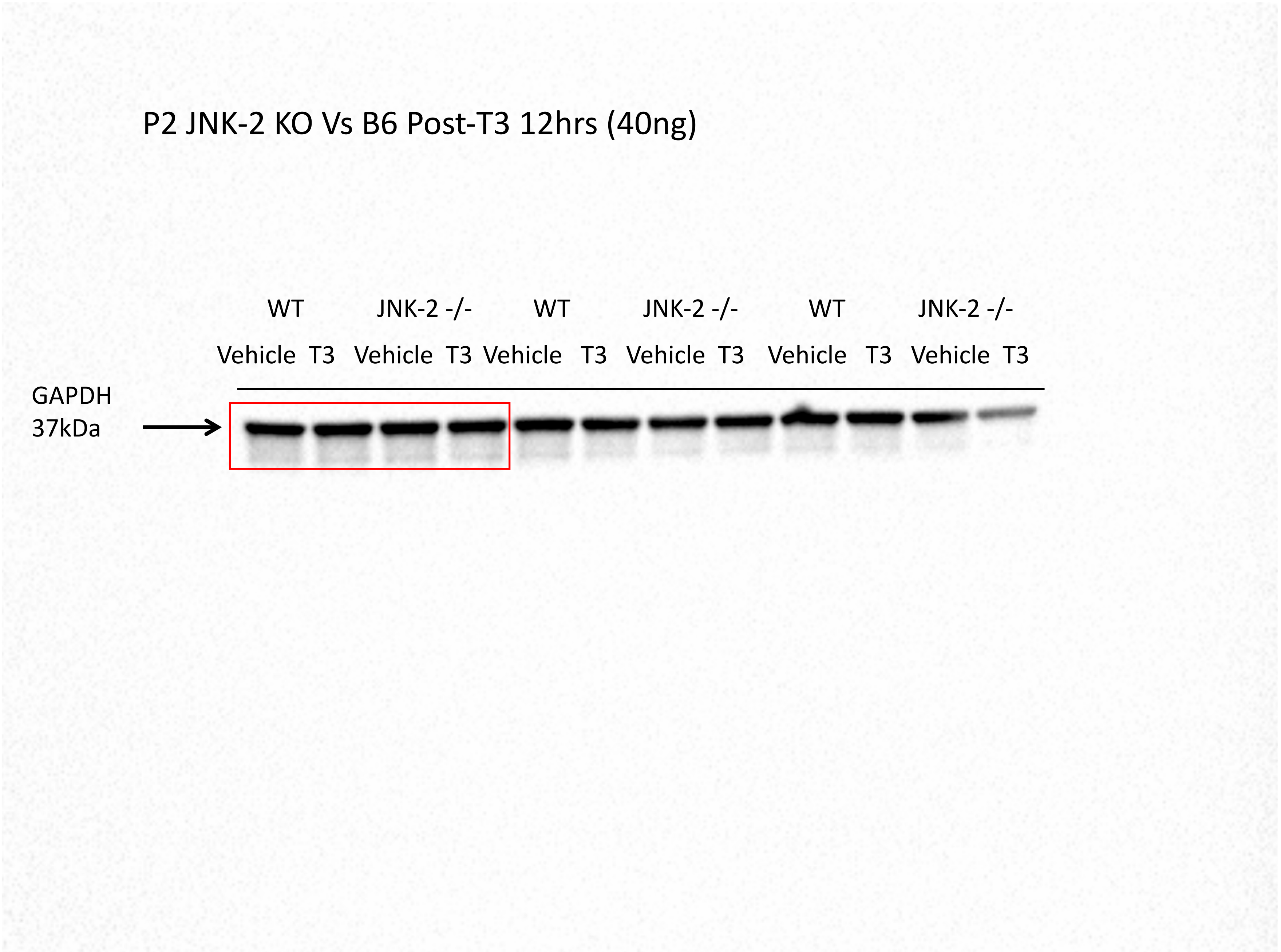

P2 JNK-2 KO Vs B6 Post-T3 12hrs (40ng)
WT
JNK-2 -/-
WT
JNK-2 -/-
WT
JNK-2 -/-
Vehicle T3 Vehicle T3 Vehicle T3 Vehicle T3 Vehicle T3 Vehicle T3
GAPDH
37kDa

Figure 6A

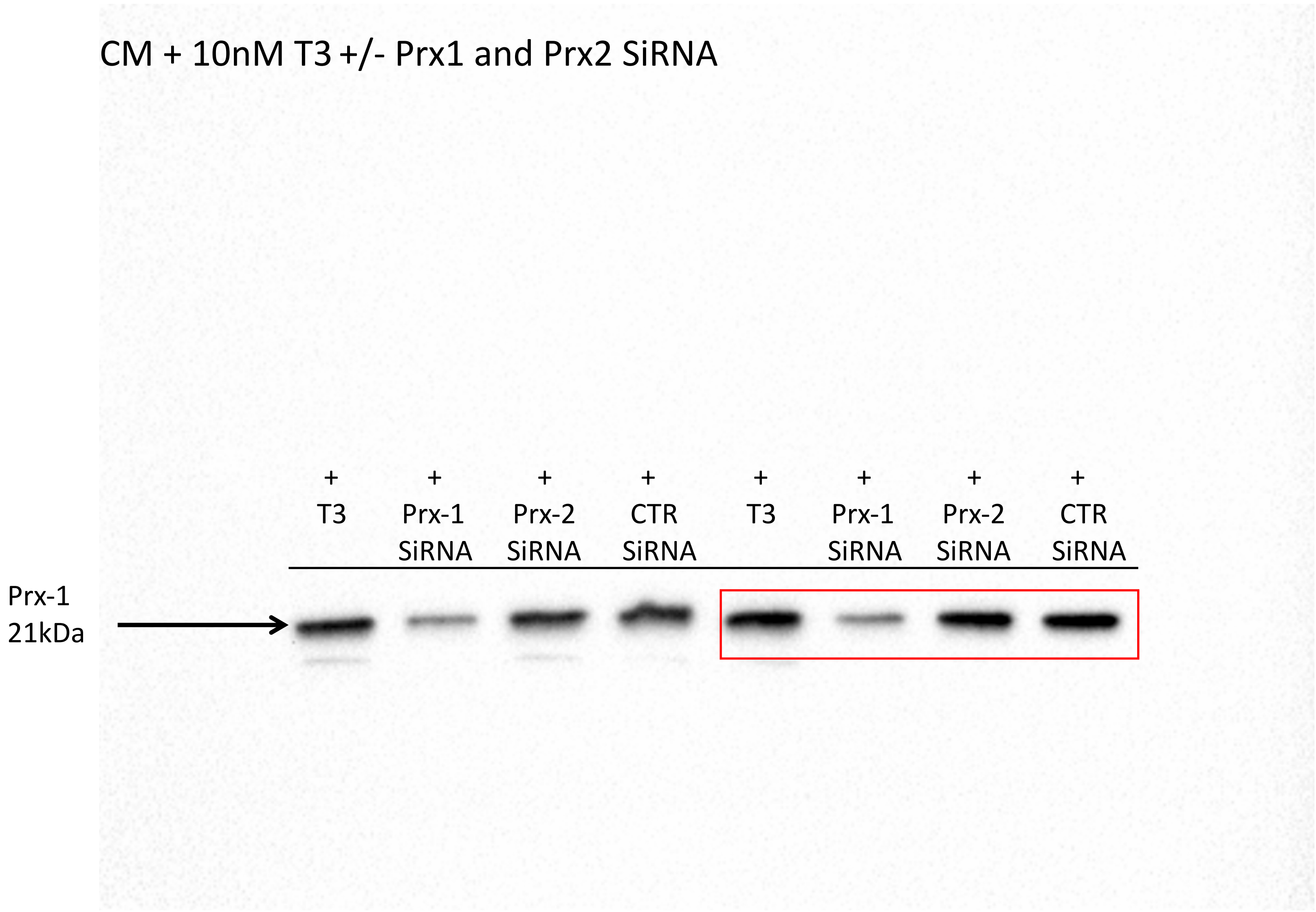
CM + 10nM T3 +/- Prx1 and Prx2 SiRNA
+ T3
+ Prx-1 SiRNA
+ Prx-2 SiRNA
+ CTR SiRNA
+ T3
+ Prx-1 SiRNA
+ Prx-2 SiRNA
+ CTR SiRNA
Prx-1
21kDa

Figure 6A

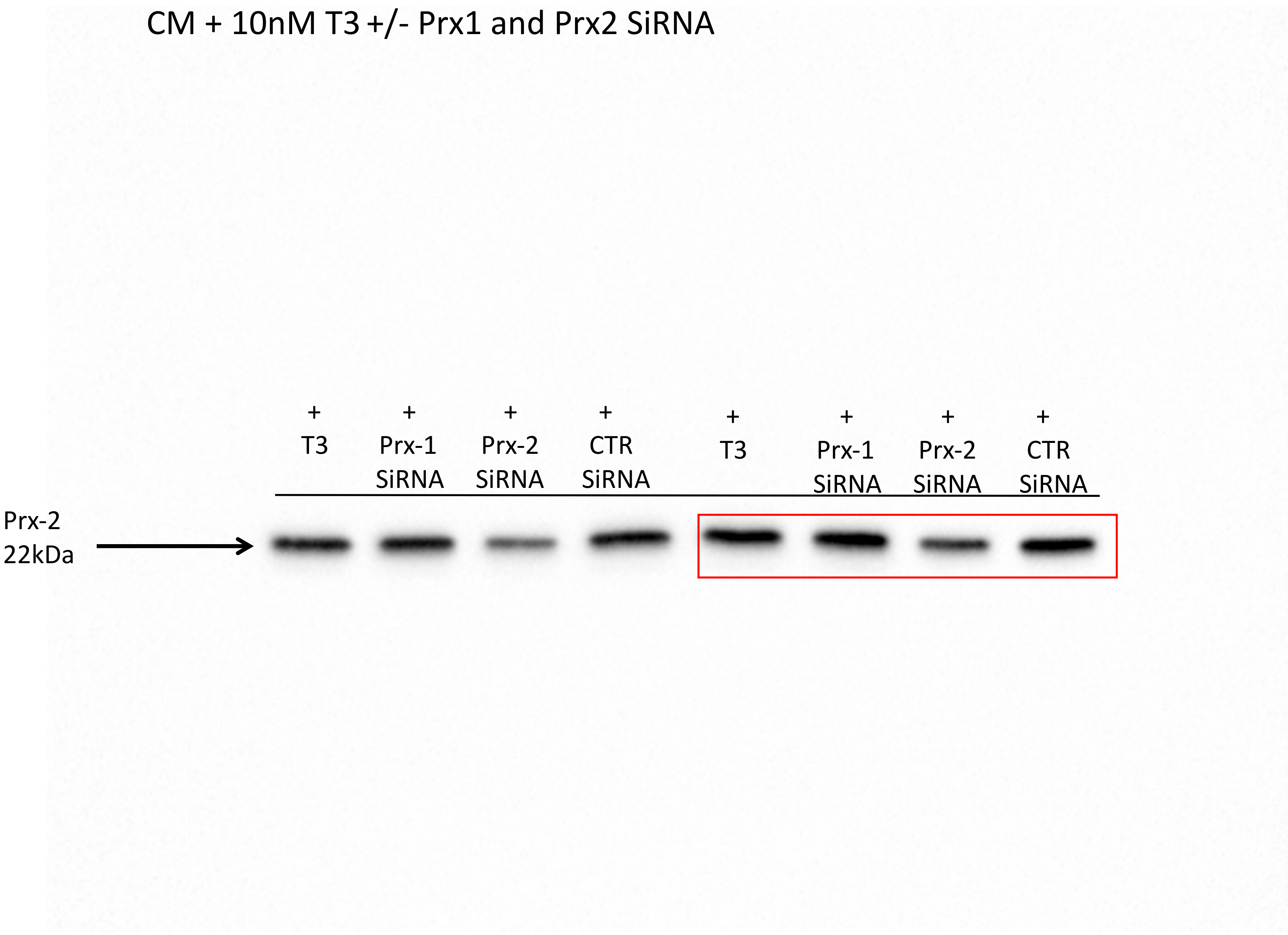
CM + 10nM T3 +/- Prx1 and Prx2 SiRNA
+ T3
+ Prx-1 SiRNA
+ Prx-2 SiRNA
+ CTR SiRNA
+ T3
+ Prx-1 SiRNA
+ Prx-2 SiRNA
+ CTR SiRNA
Prx-2
22kDa

Figure 6A

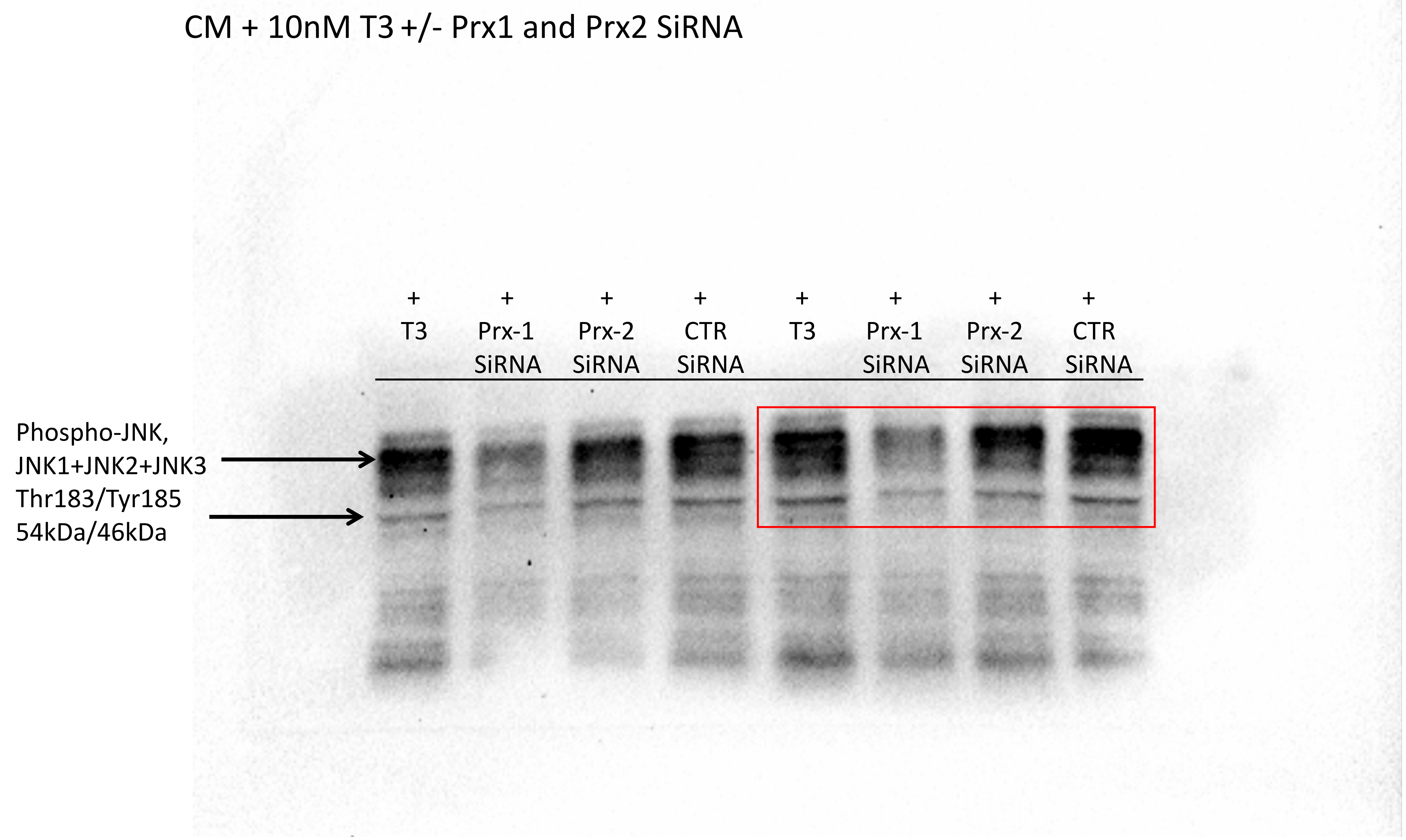

CM + 10nM T3 +/- Prx1 and Prx2 SiRNA
+
T3
+
Prx-1 SiRNA
+
Prx-2 SiRNA
+
CTR SiRNA
+
T3
+
Prx-1 SiRNA
+
Prx-2 SiRNA
+
CTR SiRNA
Phospho-JNK,
JNK1+JNK2+JNK3
Thr183/Tyr185
54kDa/46kDa

Figure 6A

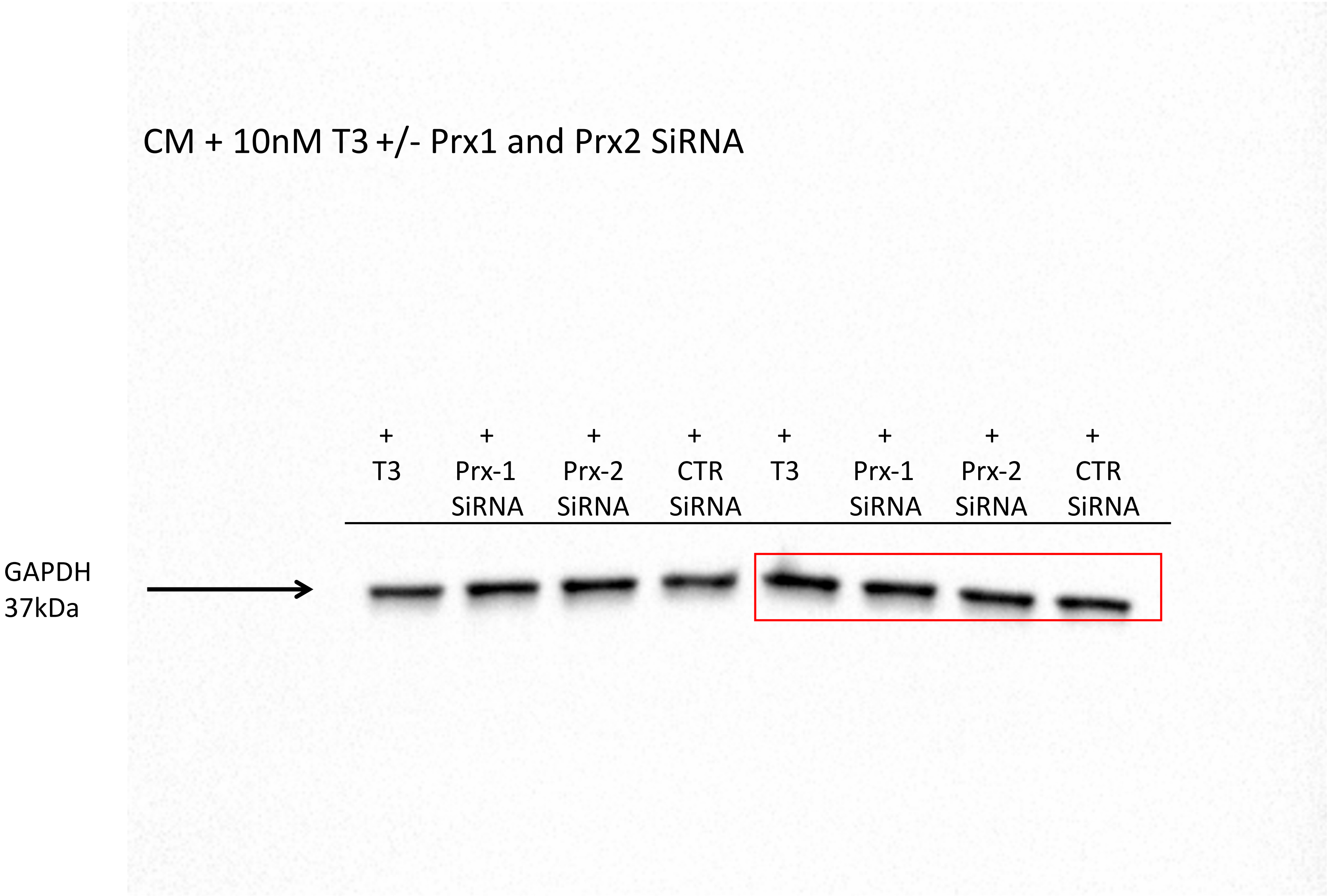
CM + 10nM T3 +/- Prx1 and Prx2 SiRNA
+ T3
+ Prx-1 SiRNA
+ Prx-2 SiRNA
+ CTR SiRNA
+ T3
+ Prx-1 SiRNA
+ Prx-2 SiRNA
+ CTR SiRNA
GAPDH
37kDa

Figure 6B

CM + 10nM T3 Native Gel (Multimers) IB: JNK-2

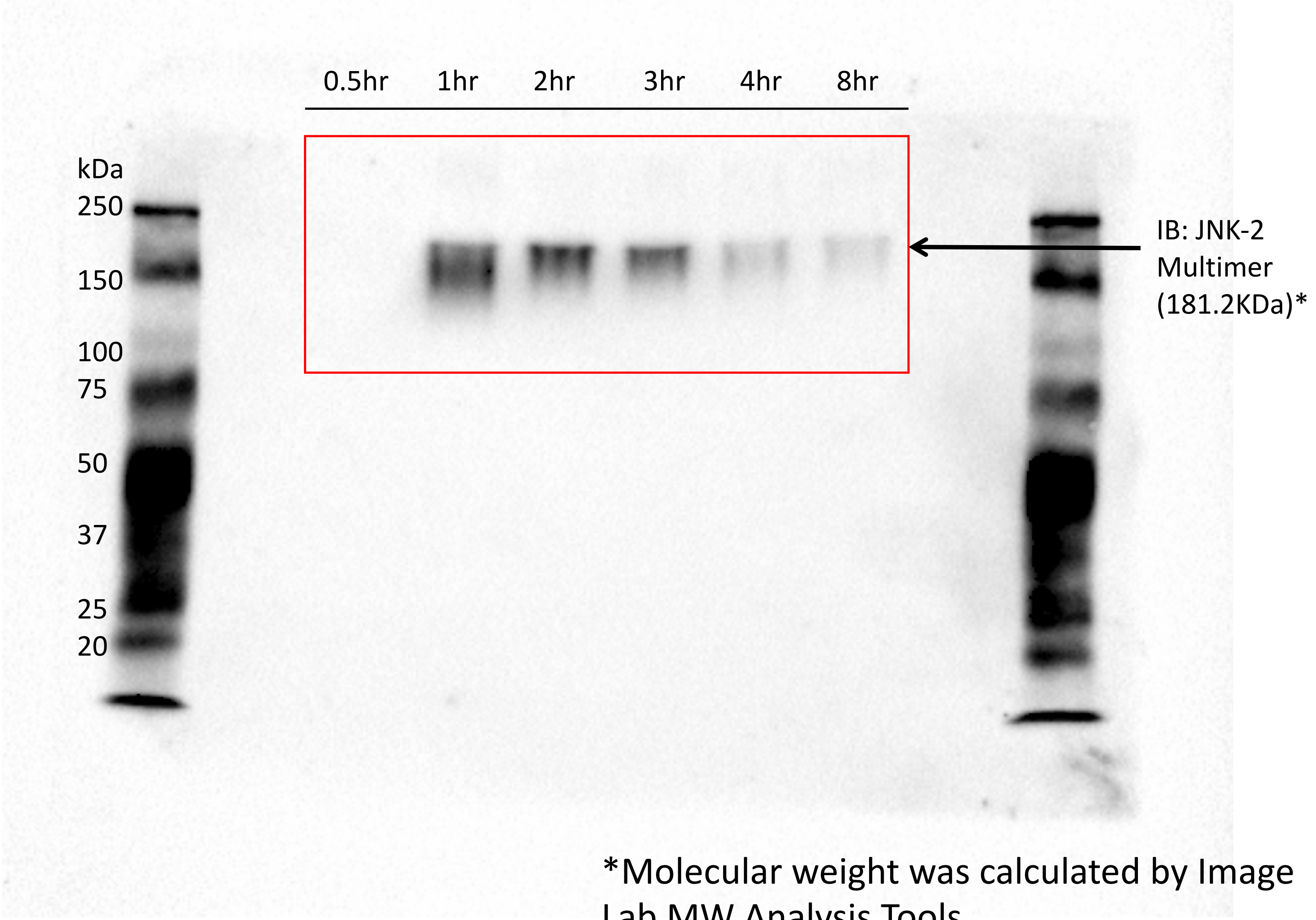

*Molecular weight was calculated by Image Lab MW Analysis Tools

Figure 6B

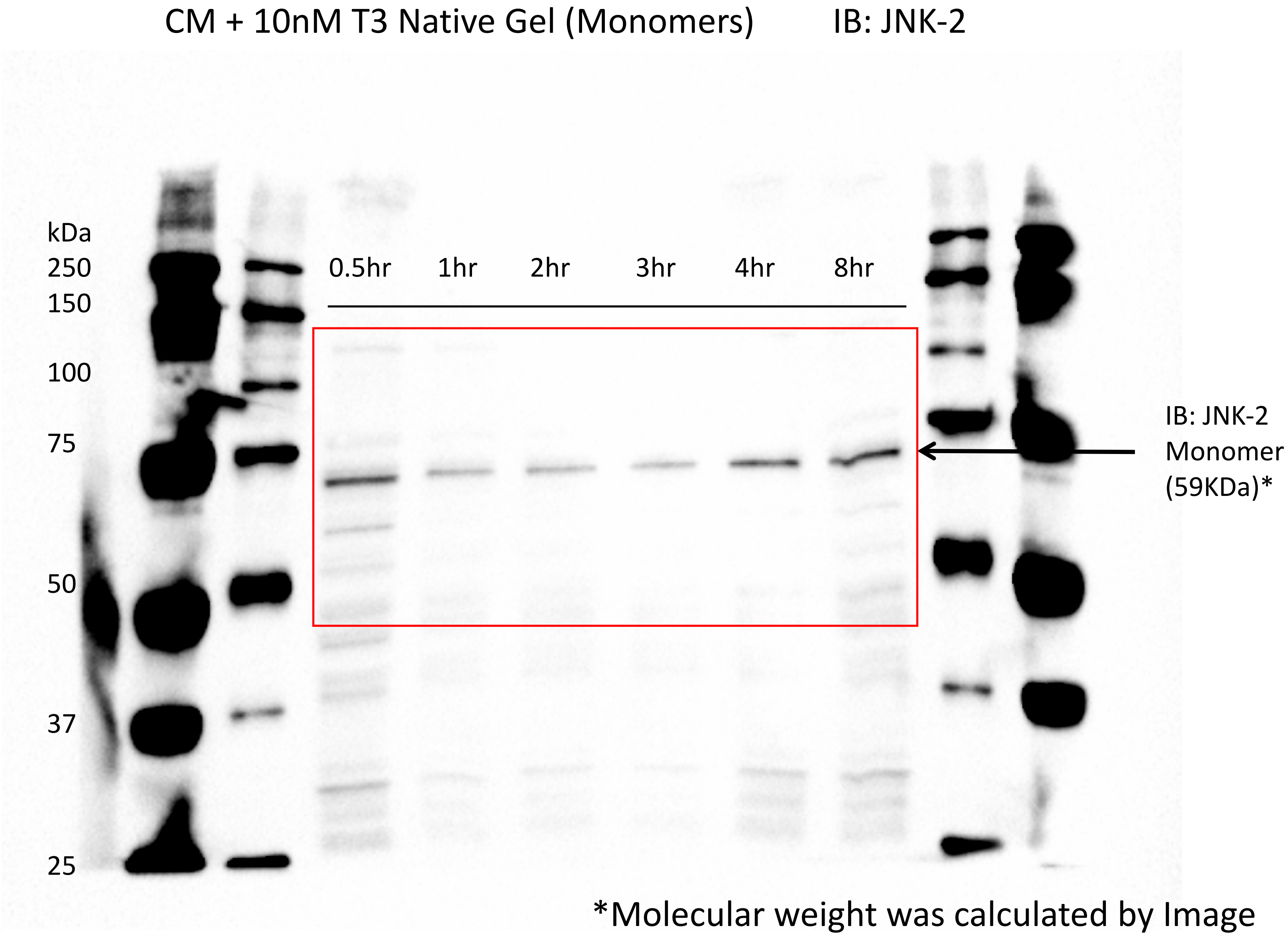

*Molecular weight was calculated by Image Lab MW Analysis Tools

Figure 6C

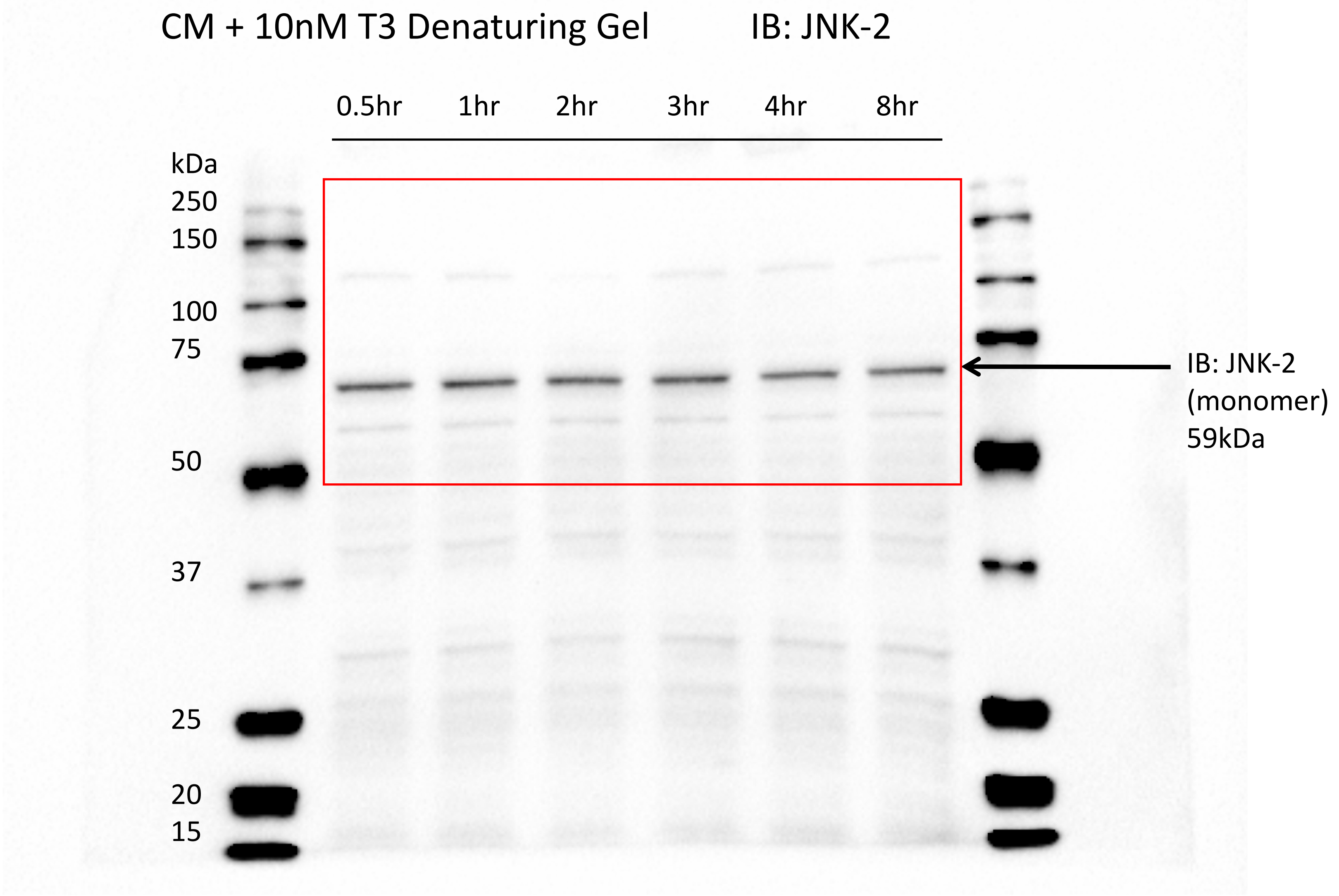

*Molecular weight was calculated by Image Lab MW Analysis Tools

Figure 6D

CM + 10nM T3 Native Gel (Multimers)   IB:Prx-1

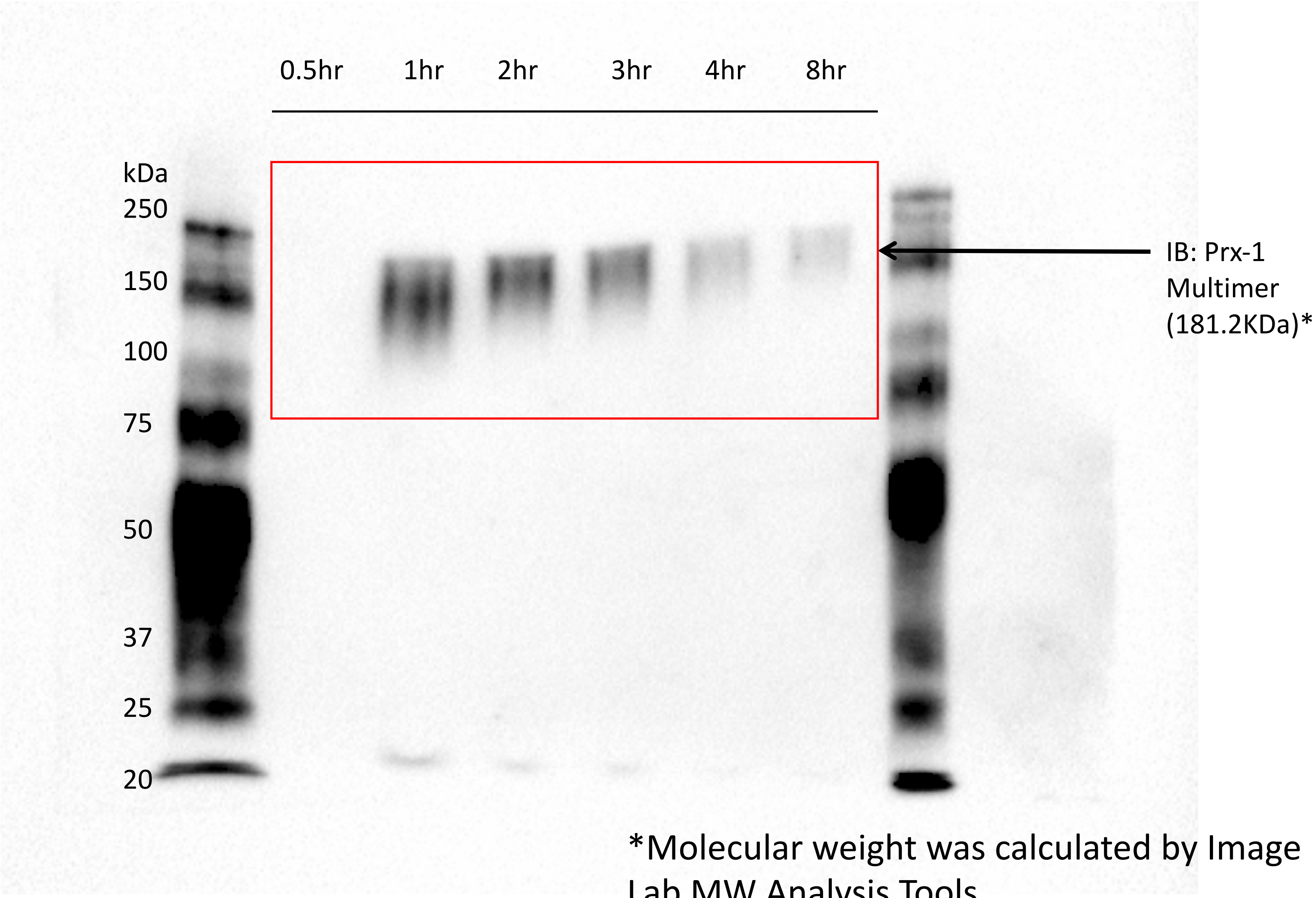

*Molecular weight was calculated by Image Lab MW Analysis Tools

Figure 6D

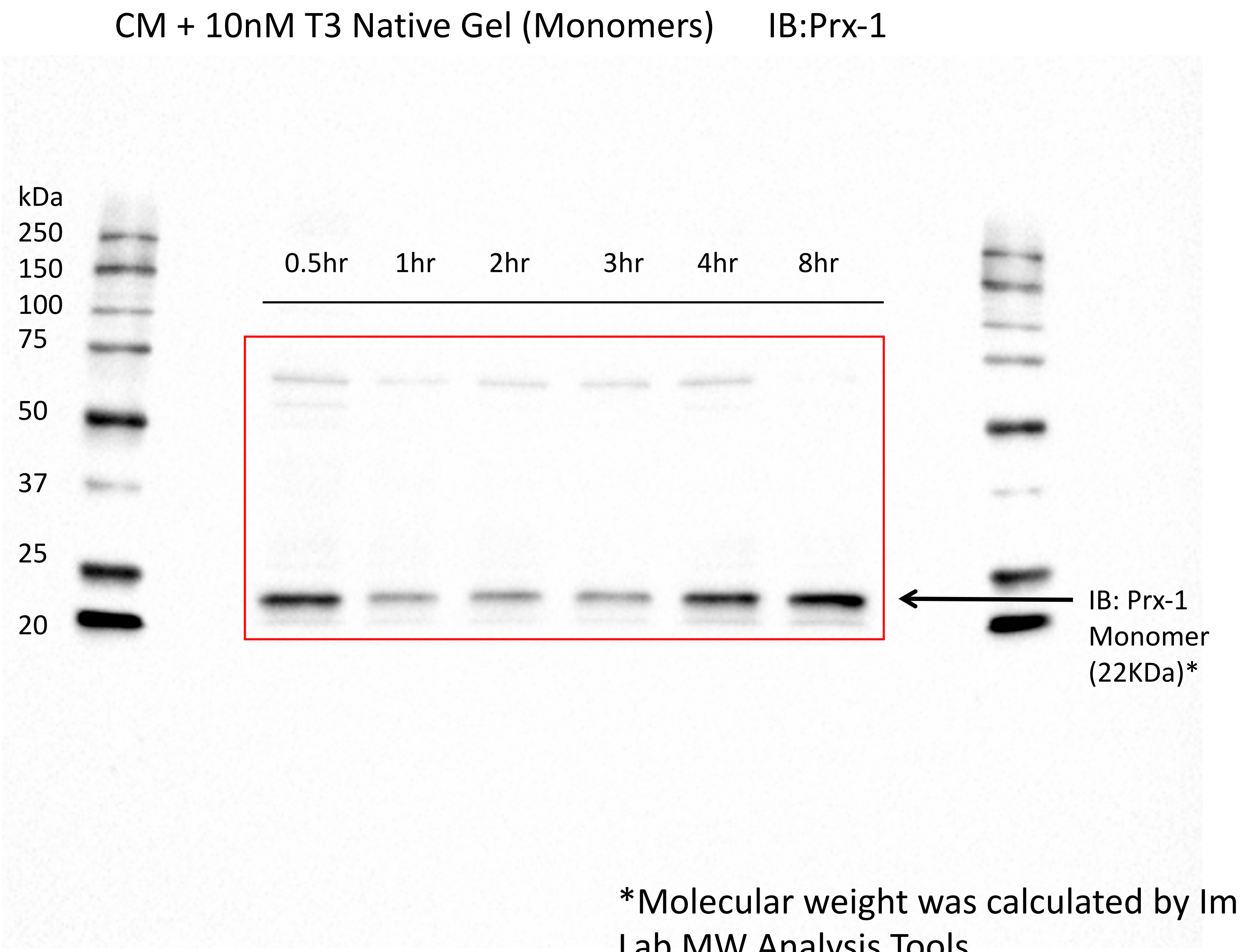

*Molecular weight was calculated by Image Lab MW Analysis Tools

Figure 6E

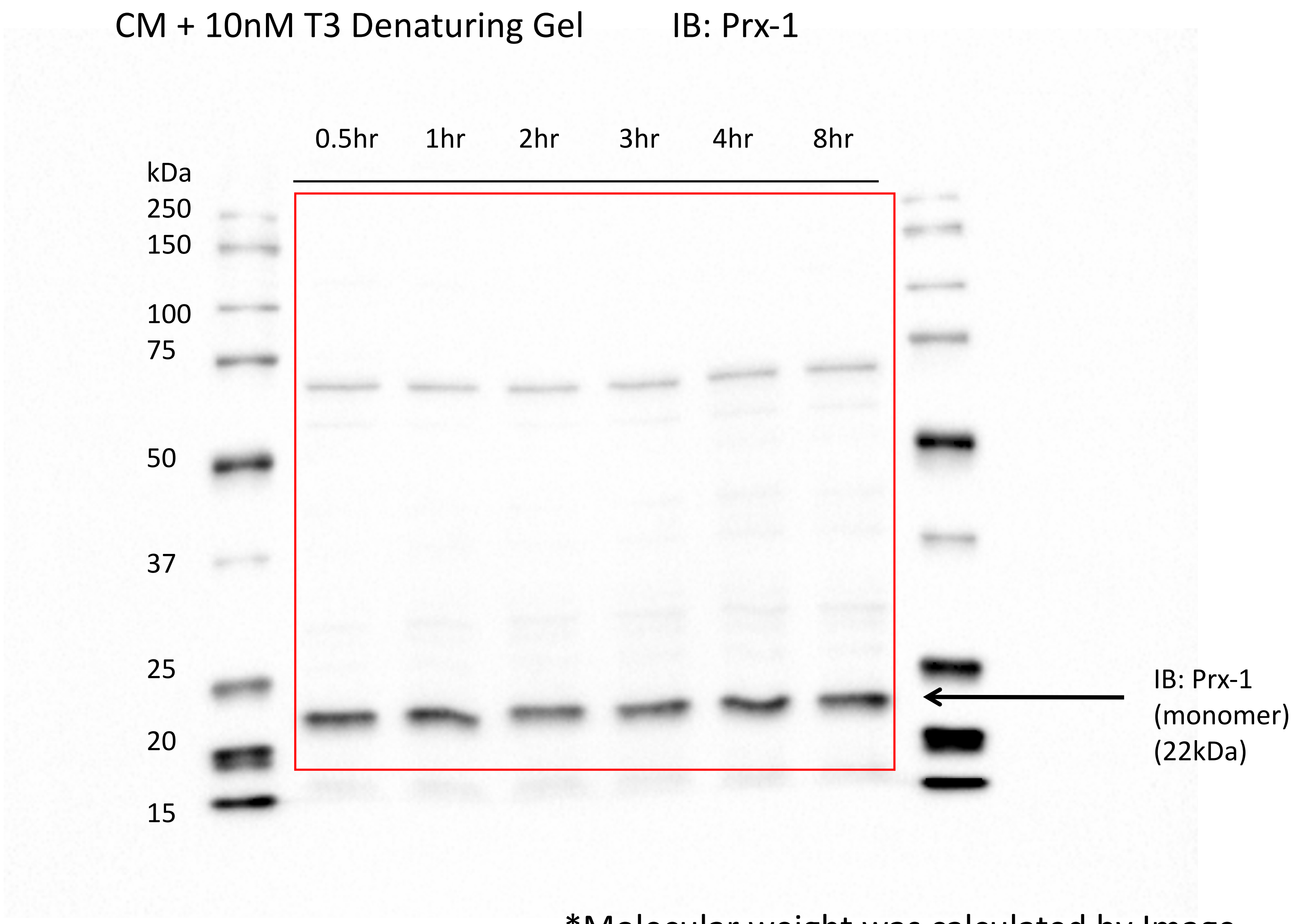

*Molecular weight was calculated by Image Lab MW Analysis Tools

Figure 6F

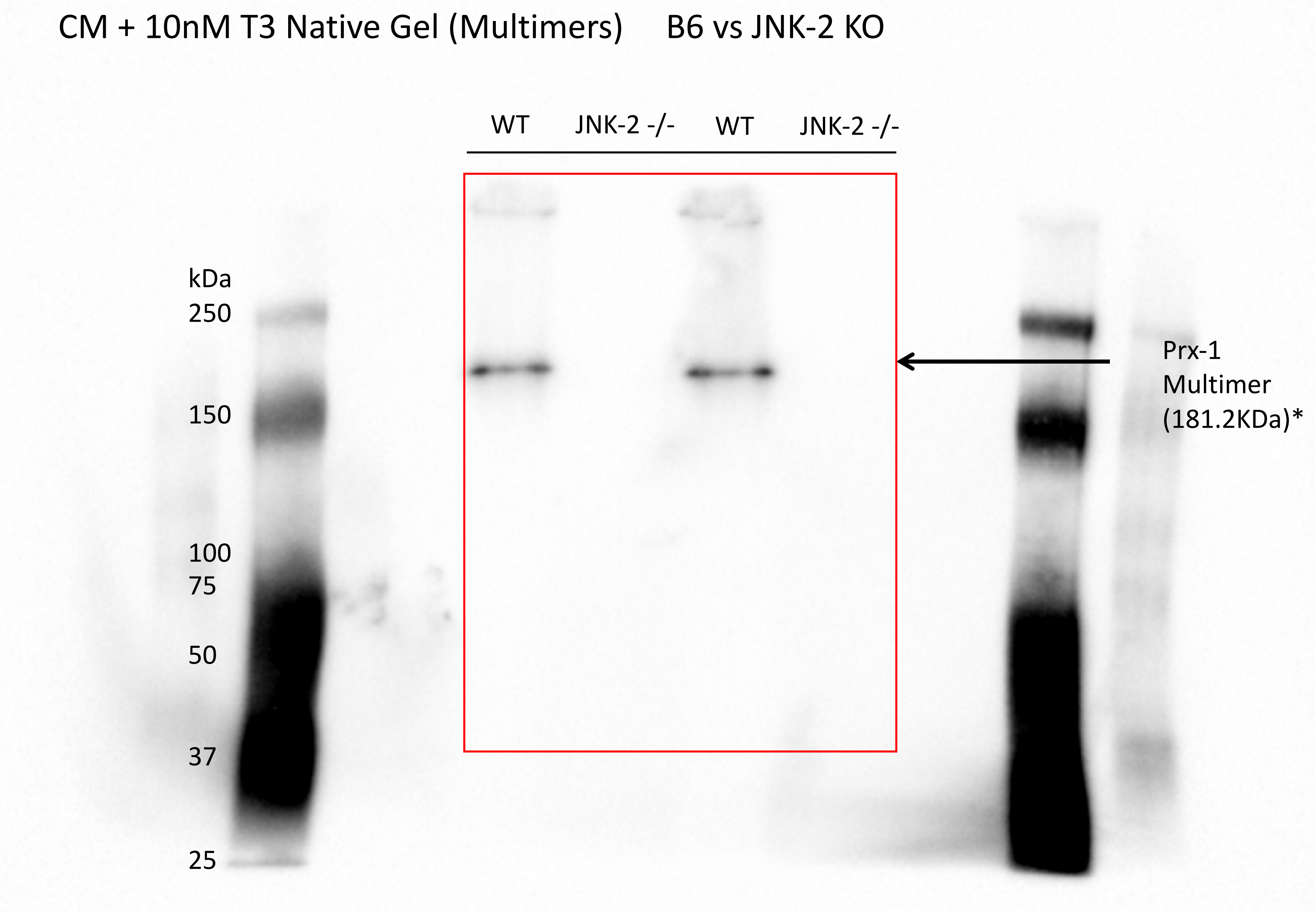

*Molecular weight was calculated by Image Lab MW Analysis Tools

Figure 6F

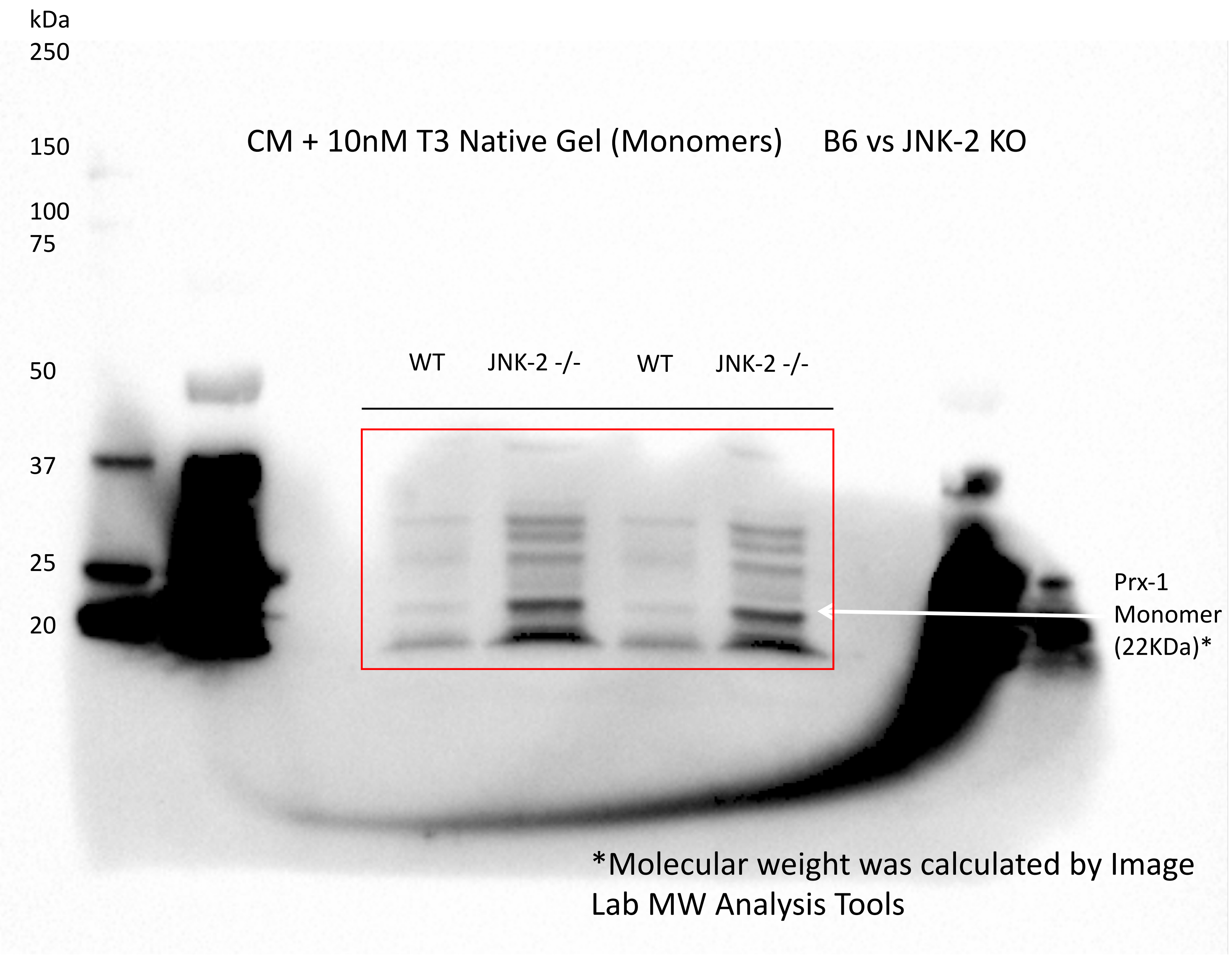
kDa
250
150
100
75
50
37
25
20
CM + 10nM T3 Native Gel (Monomers)
B6 vs JNK-2 KO
WT
JNK-2 -/-
WT
JNK-2 -/-
Prx-1
Monomer
(22KDa)*
*Molecular weight was calculated by Image Lab MW Analysis Tools

Figure 6G

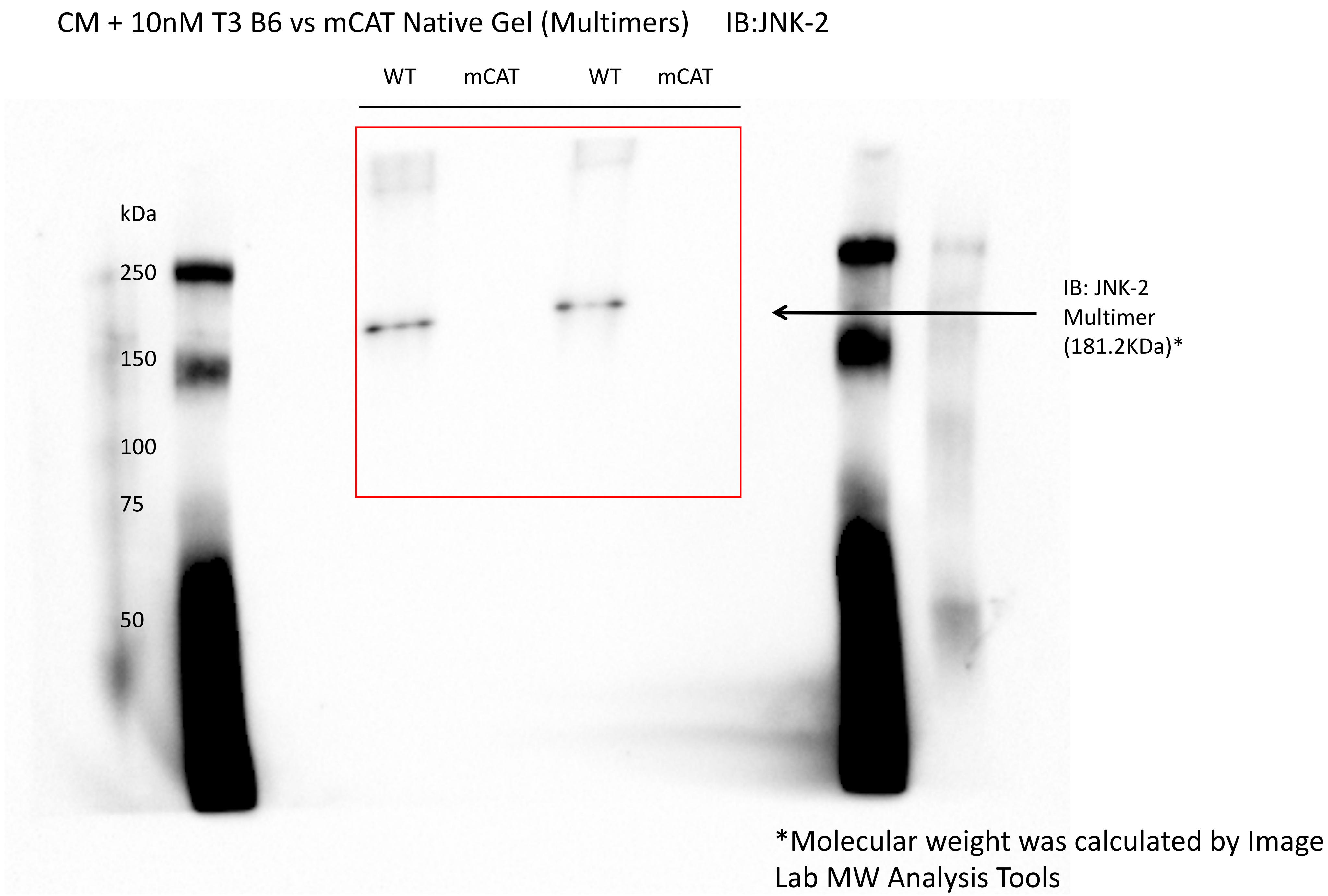
CM + 10nM T3 B6 vs mCAT Native Gel (Multimers) IB:JNK-2
WT
mCAT
WT
mCAT
kDa
250
150
100
75
50
IB: JNK-2
Multimer
(181.2KDa)*
*Molecular weight was calculated by Image Lab MW Analysis Tools

Figure 6G

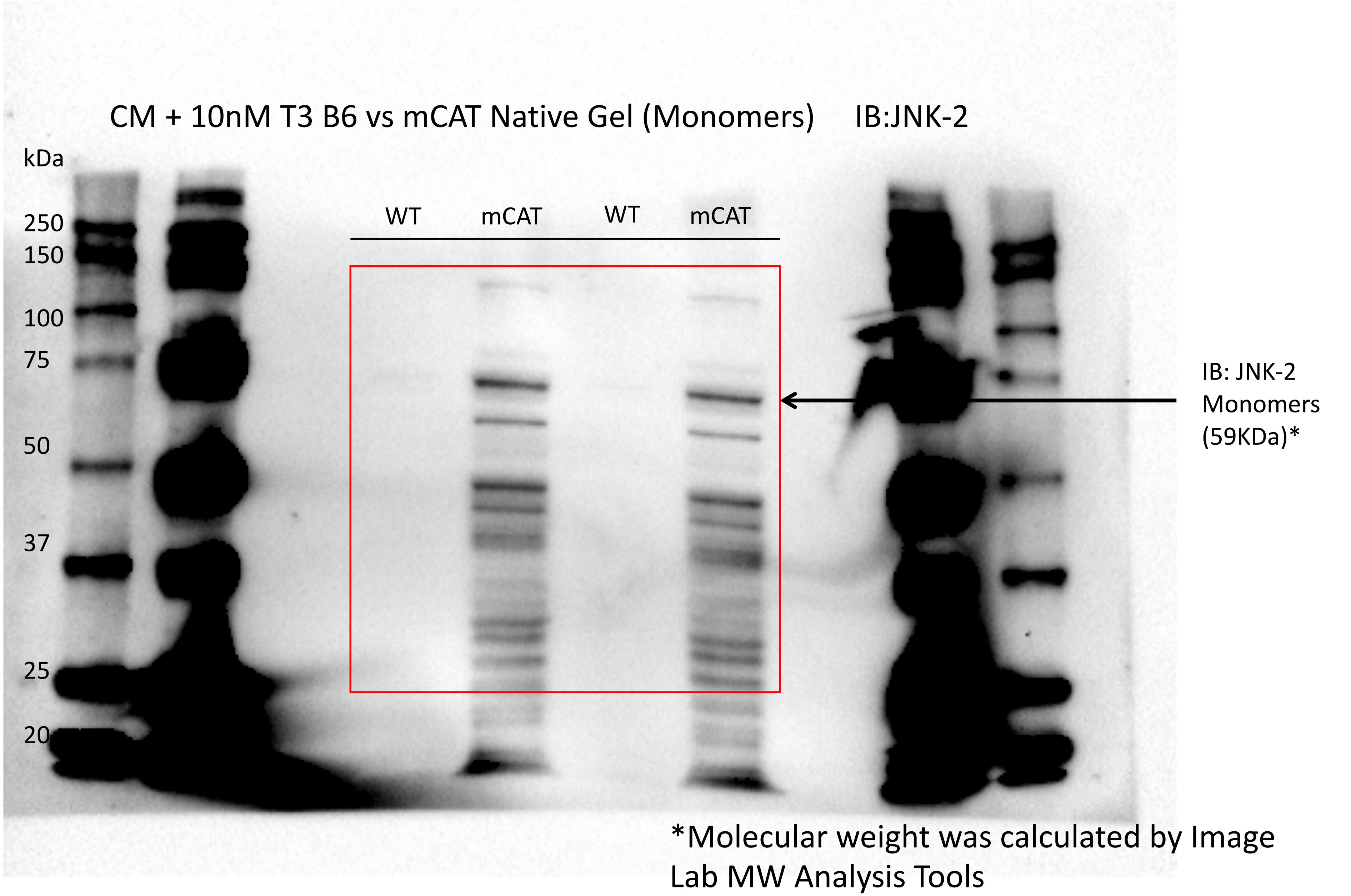
CM + 10nM T3 B6 vs mCAT Native Gel (Monomers)
IB:JNK-2
kDa
250
150
100
75
50
37
25
20
WT
mCAT
WT
mCAT
IB: JNK-2
Monomers
(59KDa)*
*Molecular weight was calculated by Image
Lab MW Analysis Tools

Figure 7A

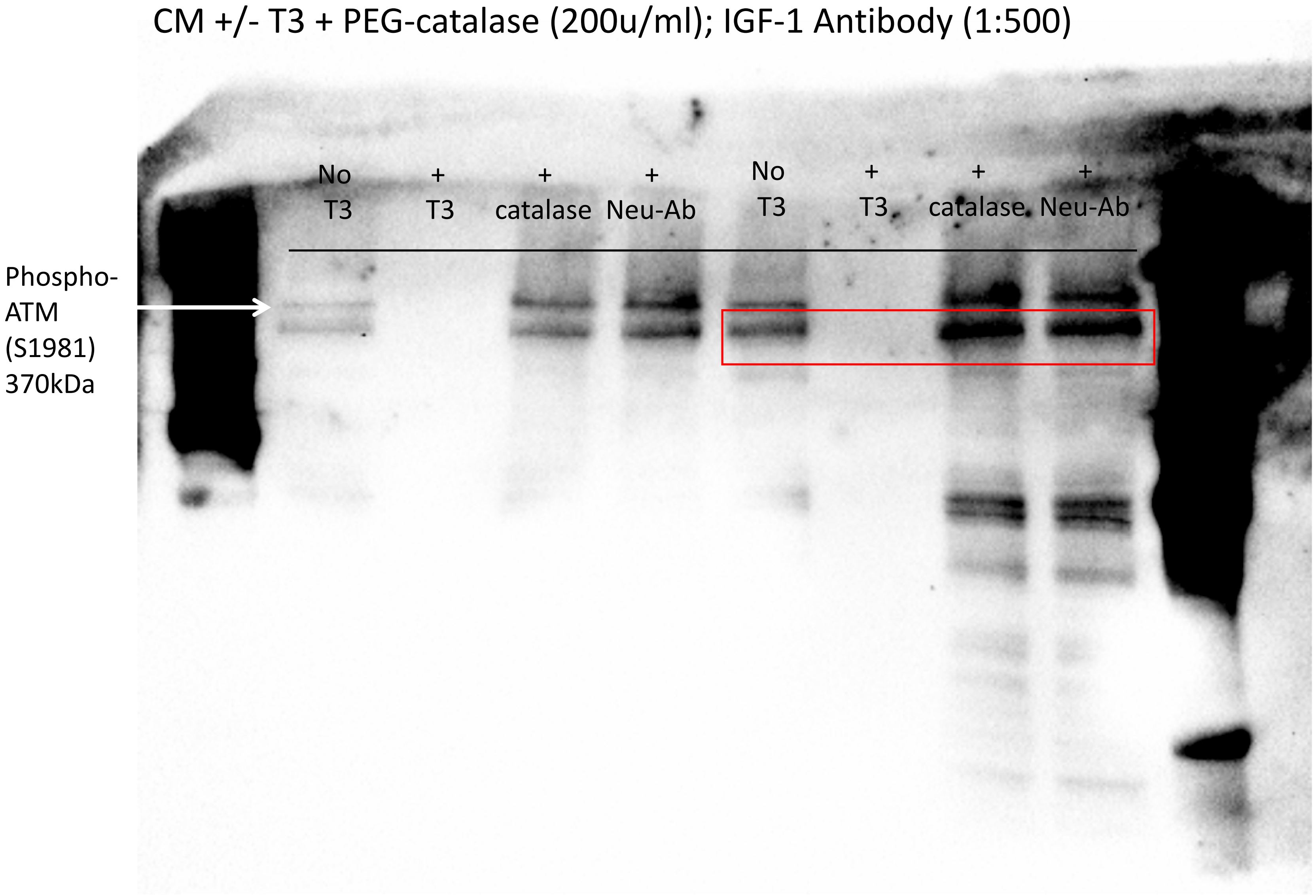
CM +/- T3 + PEG-catalase (200u/ml); IGF-1 Antibody (1:500)
No T3
+ T3
+ catalase
+ Neu-Ab
No T3
+ T3
+ catalase
+ Neu-Ab
Phospho-ATM (S1981) 370kDa

Figure 7A

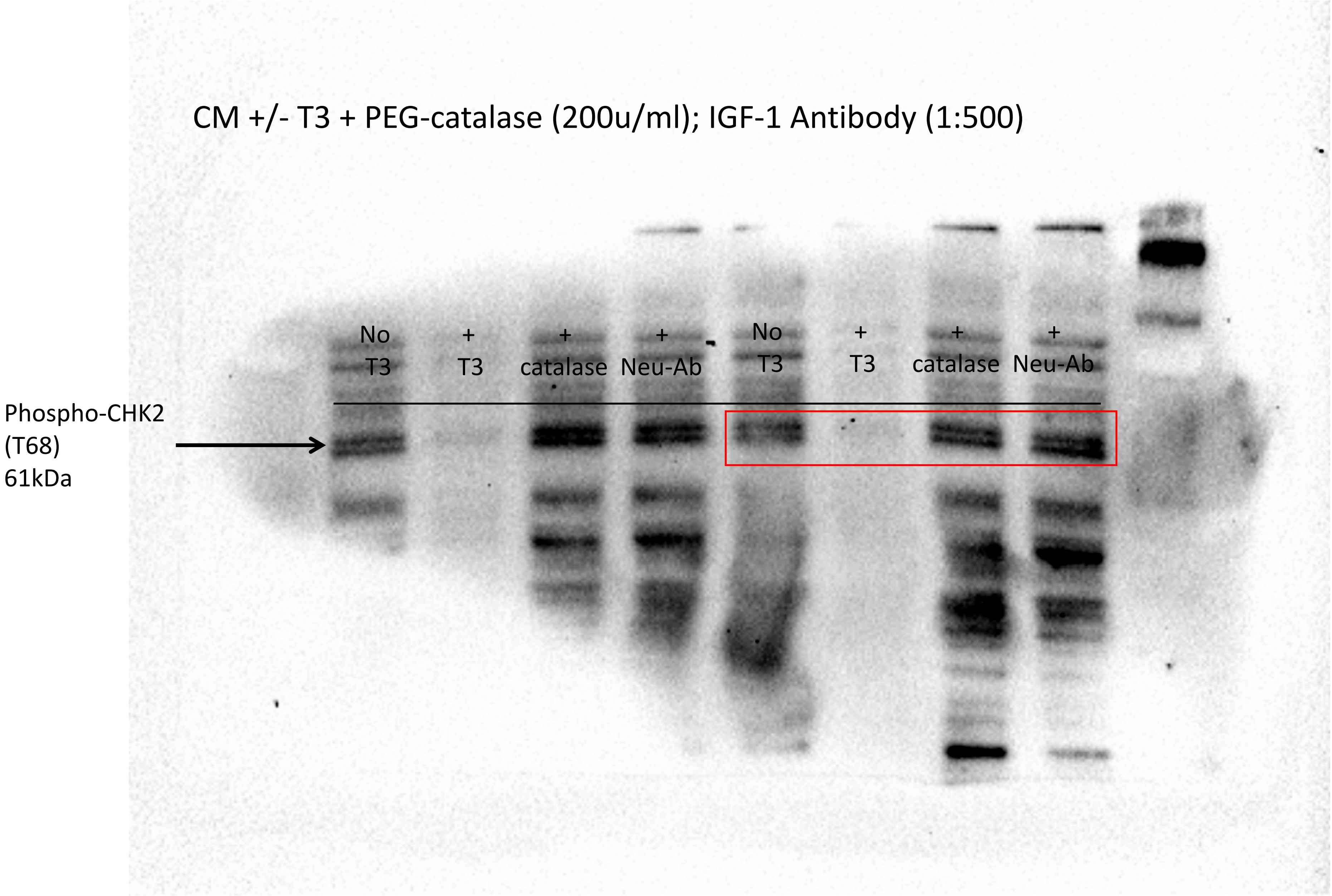
CM +/- T3 + PEG-catalase (200u/ml); IGF-1 Antibody (1:500)
No T3
+ T3
+ catalase
+ Neu-Ab
No T3
+ T3
+ catalase
+ Neu-Ab
Phospho-CHK2 (T68) 61kDa

Figure 7A

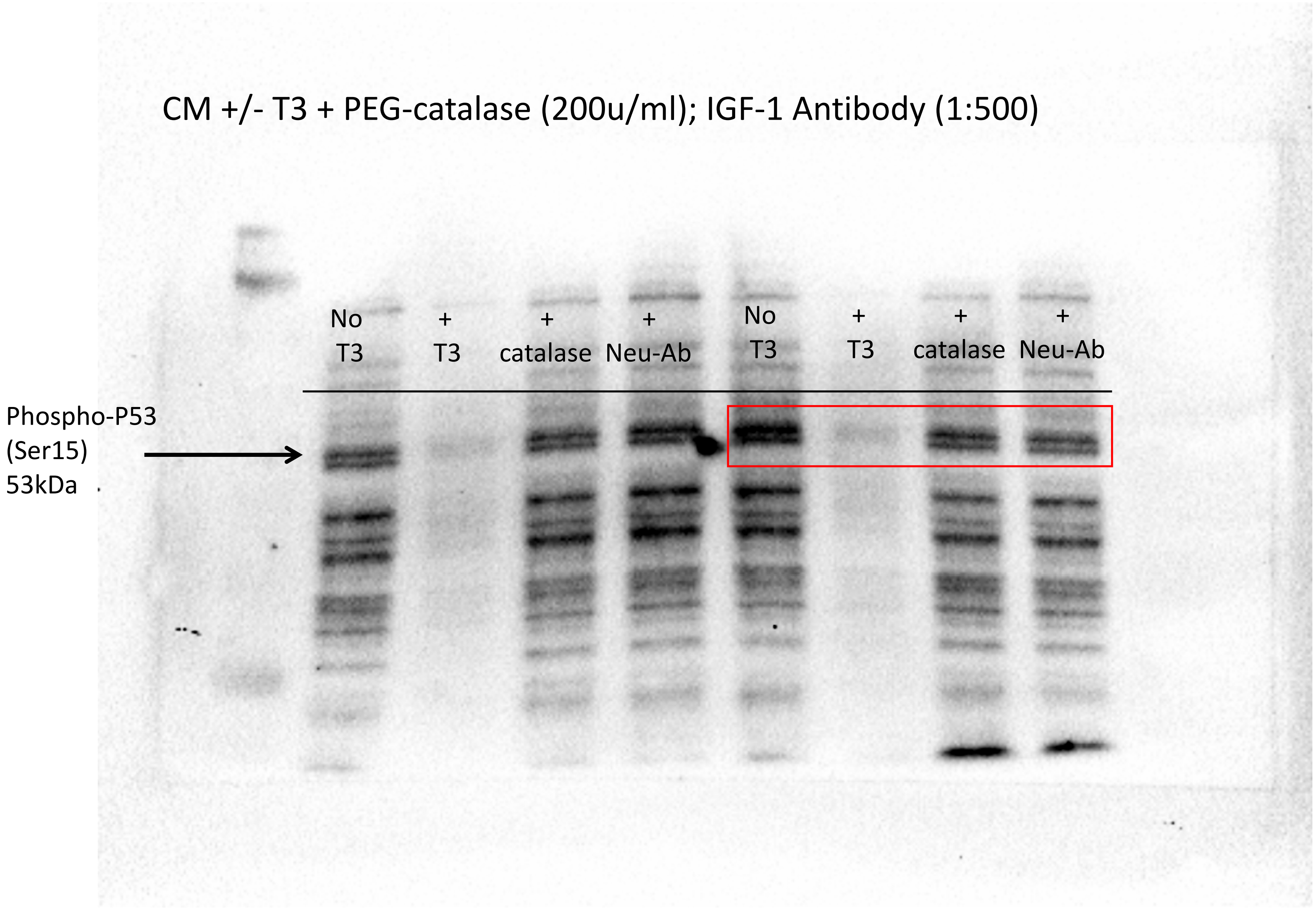
CM +/- T3 + PEG-catalase (200u/ml); IGF-1 Antibody (1:500)
No T3
+ T3
+ catalase
+ Neu-Ab
No T3
+ T3
+ catalase
+ Neu-Ab
Phospho-P53 (Ser15) 53kDa

Figure 7A

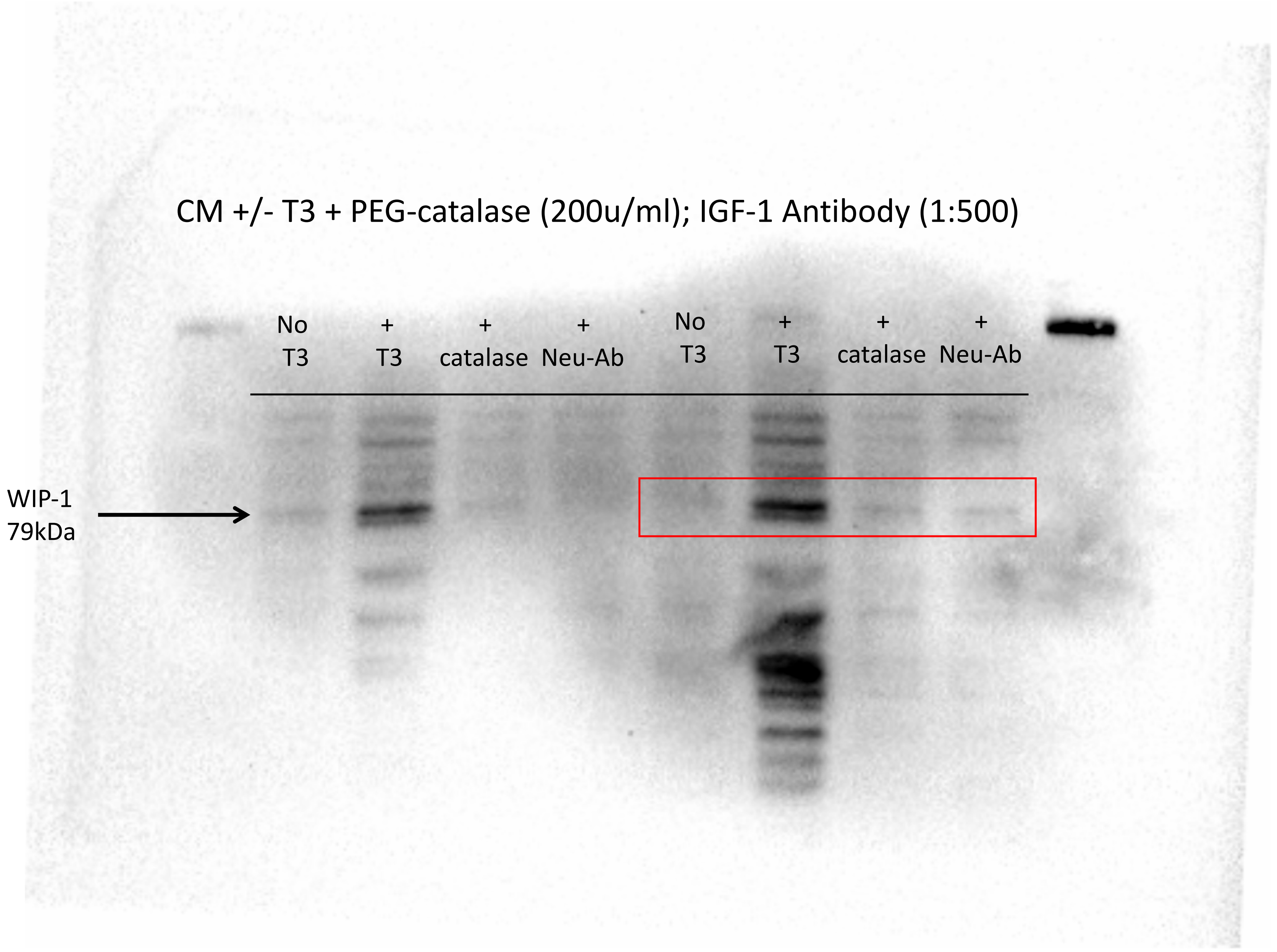
CM +/- T3 + PEG-catalase (200u/ml); IGF-1 Antibody (1:500)
No T3
+ T3
+ catalase
+ Neu-Ab
No T3
+ T3
+ catalase
+ Neu-Ab
WIP-1
79kDa

Figure 7A

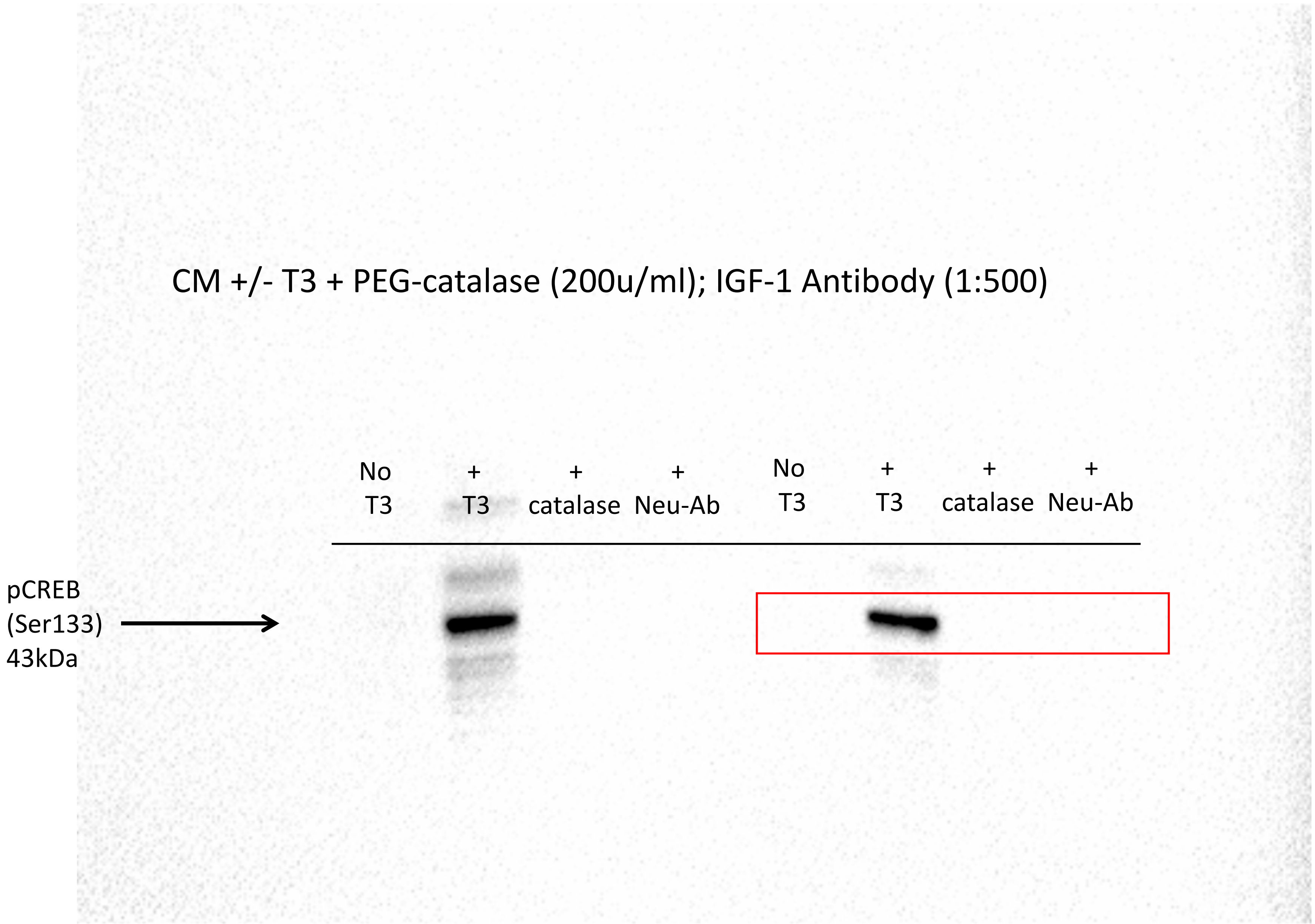
CM +/- T3 + PEG-catalase (200u/ml); IGF-1 Antibody (1:500)
No T3
+ T3
+ catalase
+ Neu-Ab
No T3
+ T3
+ catalase
+ Neu-Ab
pCREB (Ser133) 43kDa

Figure 7A

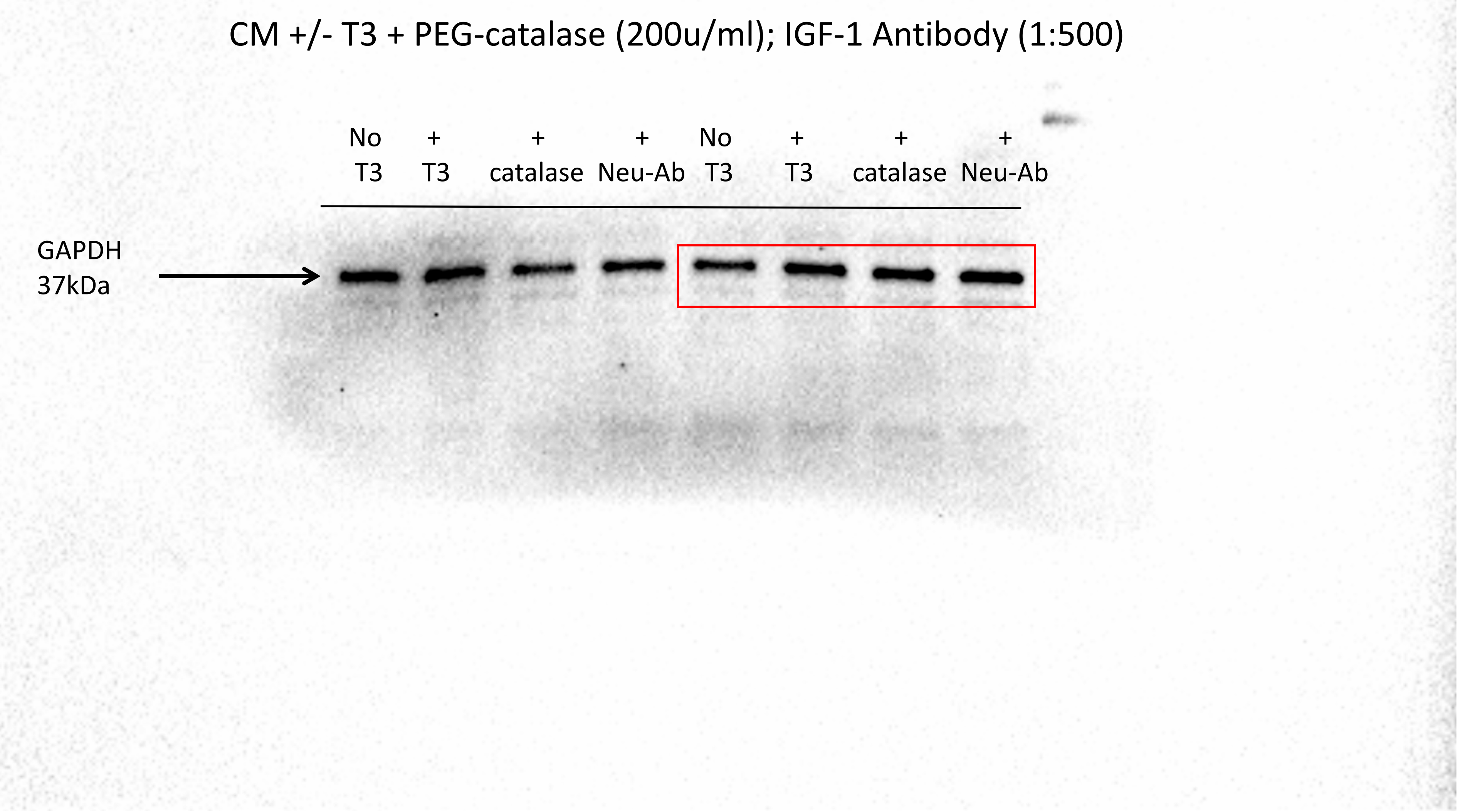

CM +/- T3 + PEG-catalase (200u/ml); IGF-1 Antibody (1:500)
No T3
+ T3
+ catalase
+ Neu-Ab
No T3
+ T3
+ catalase
+ Neu-Ab
GAPDH 37kDa

Figure 7B

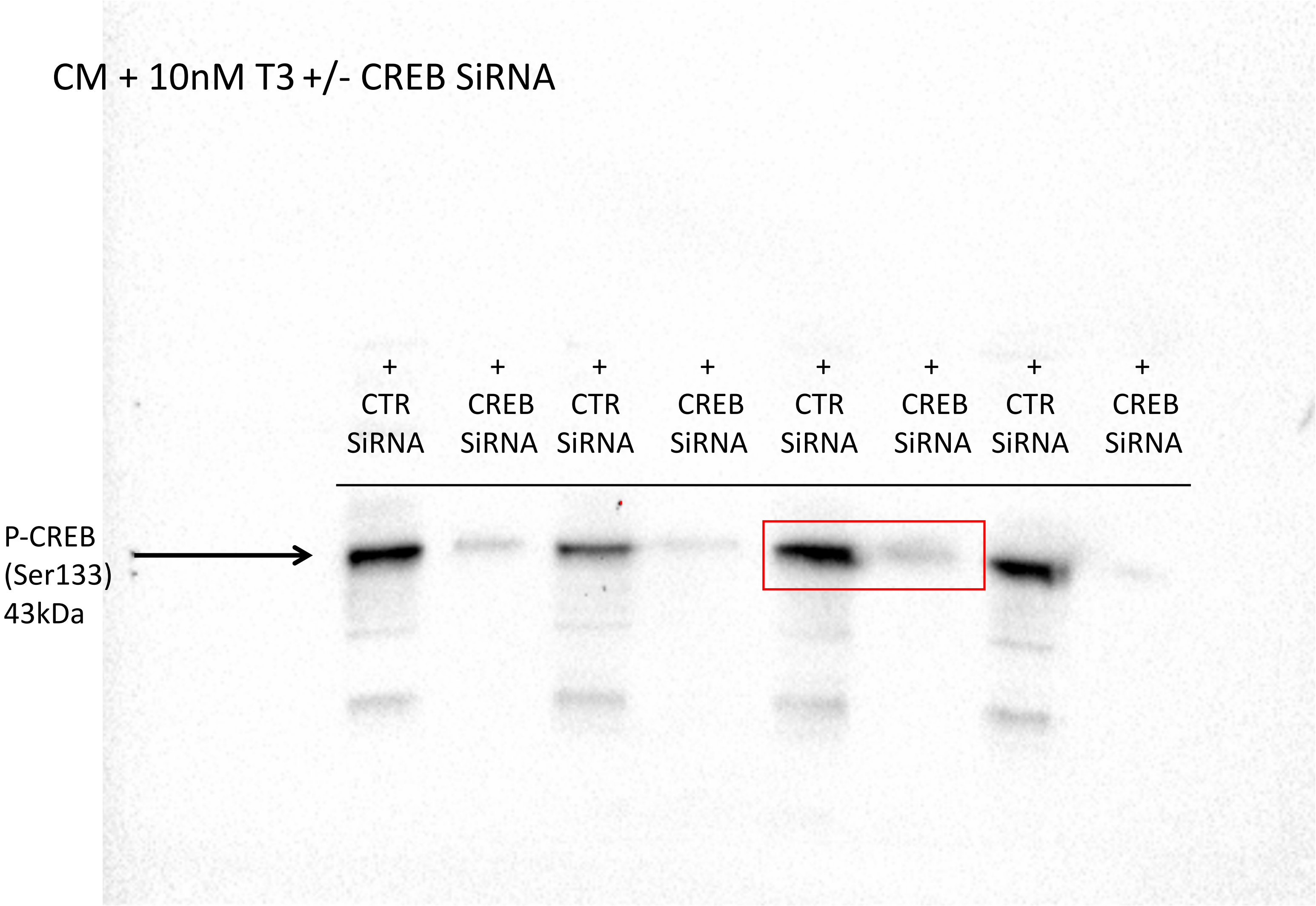
CM + 10nM T3 +/- CREB SiRNA
+ CTR SiRNA
+ CREB SiRNA
+ CTR SiRNA
+ CREB SiRNA
+ CTR SiRNA
+ CREB SiRNA
+ CTR SiRNA
+ CREB SiRNA
P-CREB (Ser133) 43kDa

Figure 7B

CM + 10nM T3 +/- CREB SiRNA

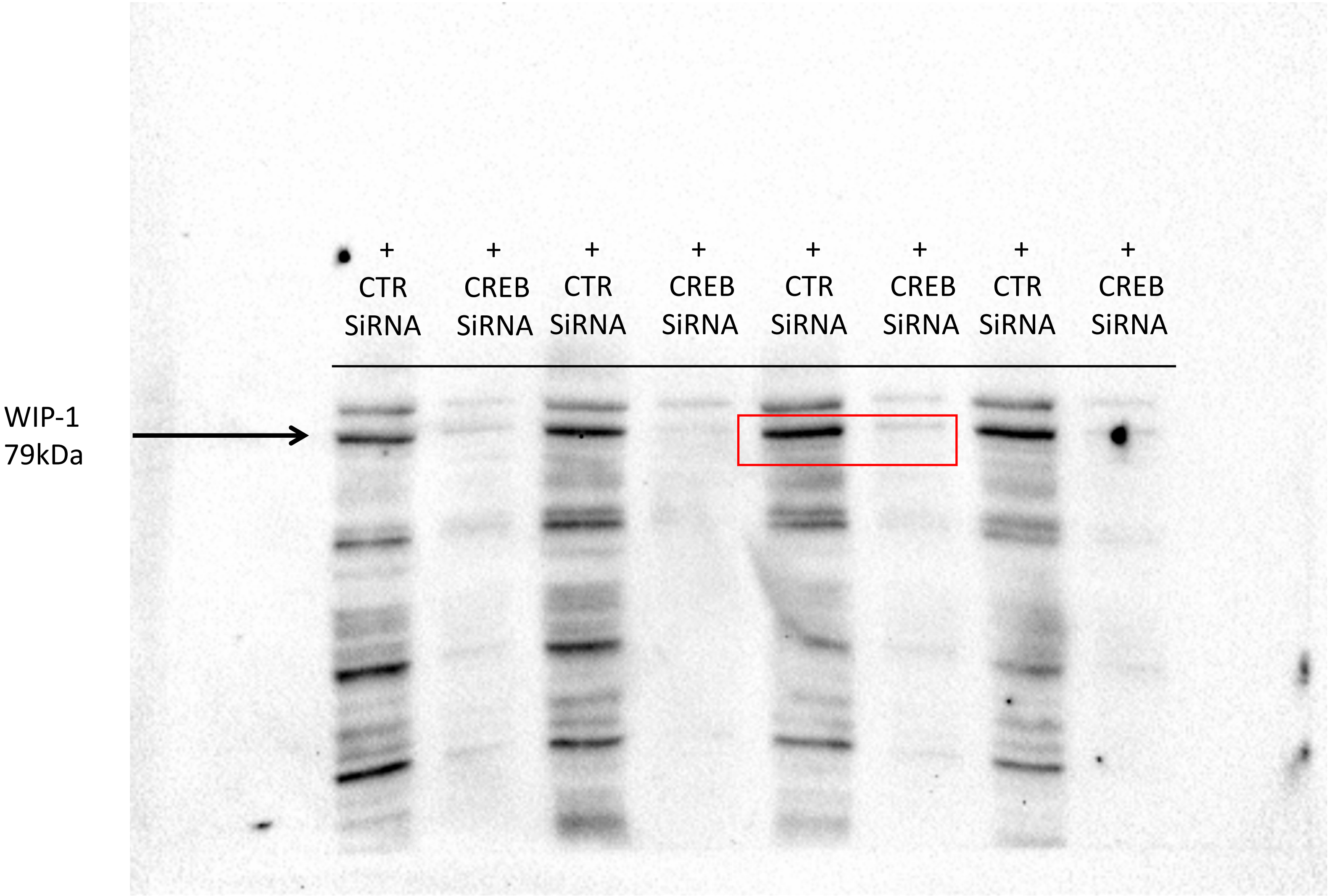

Figure 7B

CM + 10nM T3 +/- CREB SiRNA

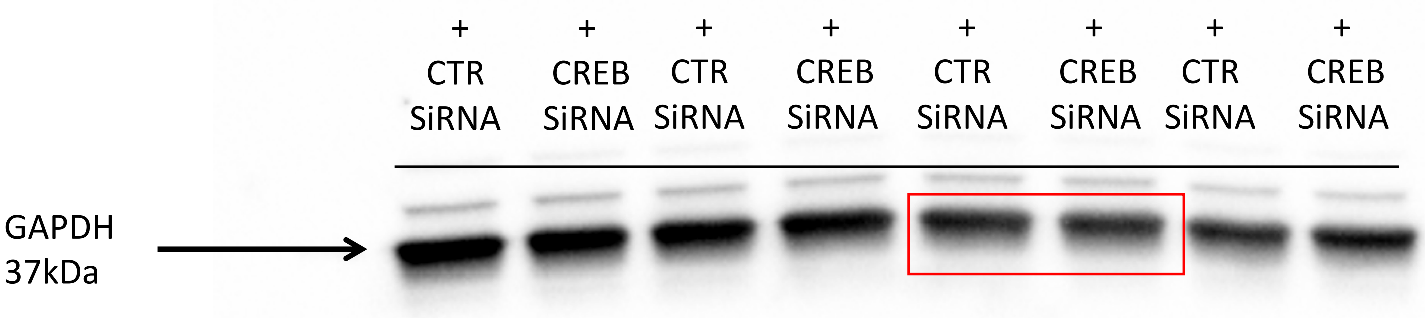

Figure 7C

CM + 10nM T3 +/- Wip-1 SiRNA

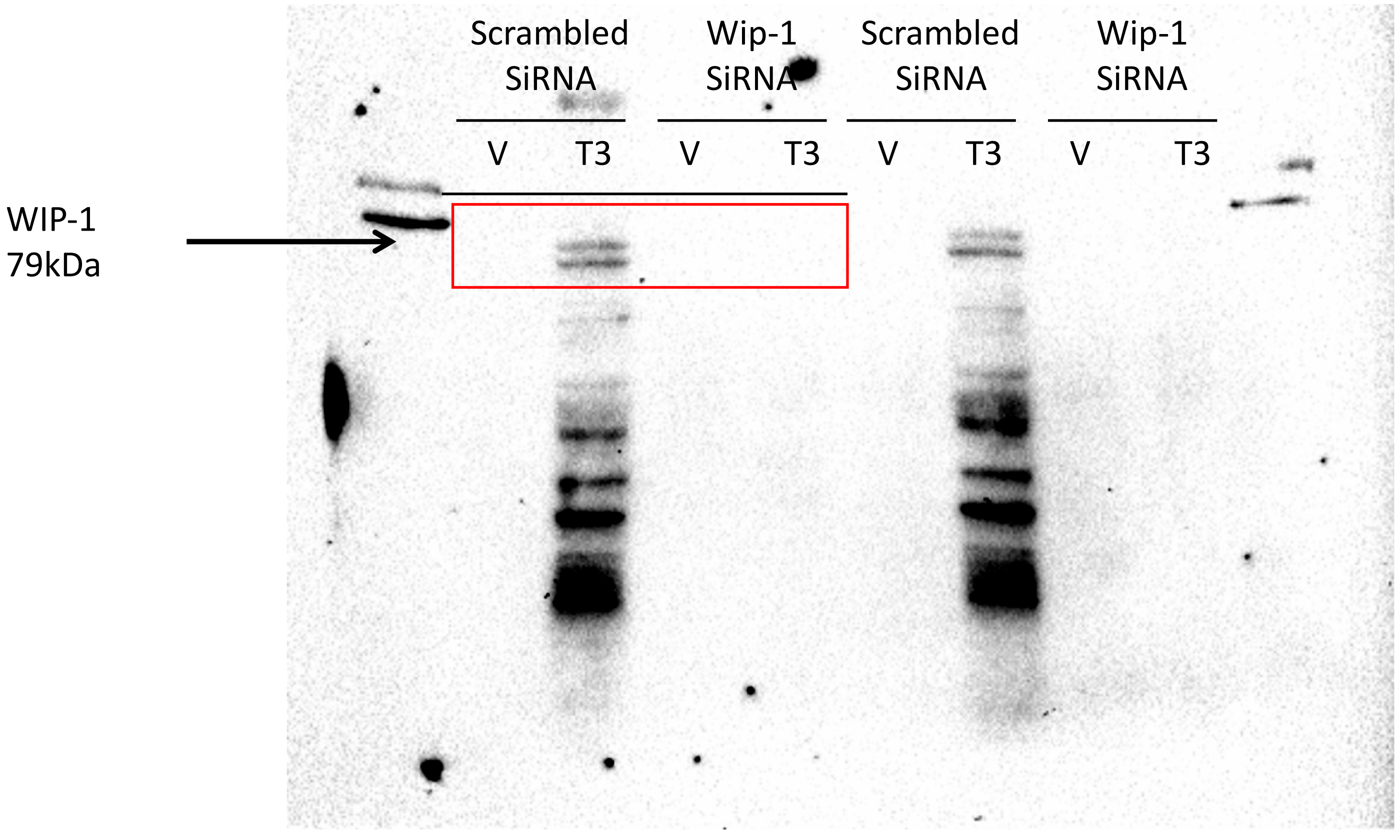

Figure 7C

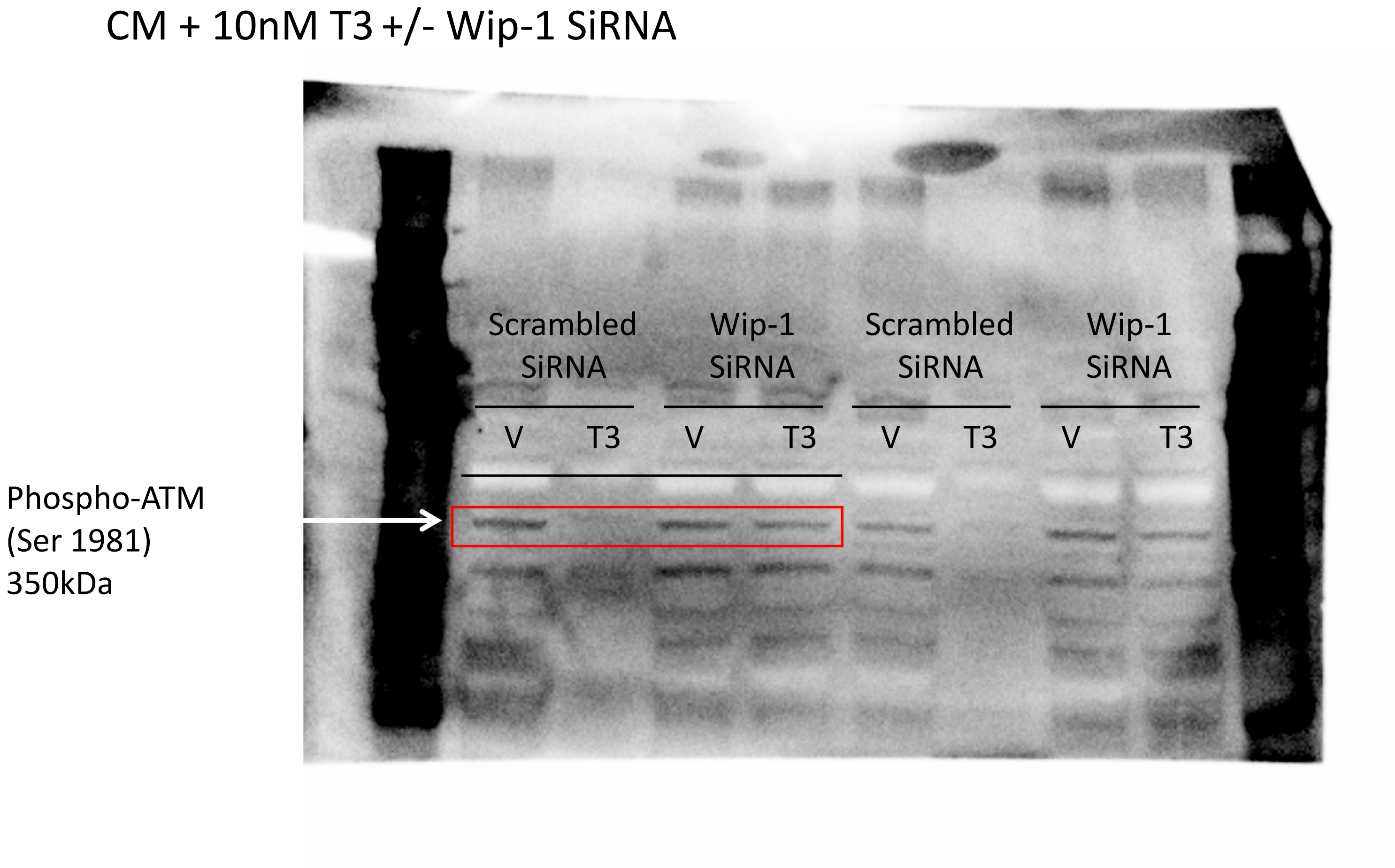

CM + 10nM T3 +/- Wip-1 SiRNA
Scrambled SiRNA
Wip-1 SiRNA
Scrambled SiRNA
Wip-1 SiRNA
V
T3
V
T3
V
T3
V
T3
Phospho-ATM (Ser 1981) 350kDa

Figure 7C

CM + 10nM T3 +/- Wip-1 SiRNA

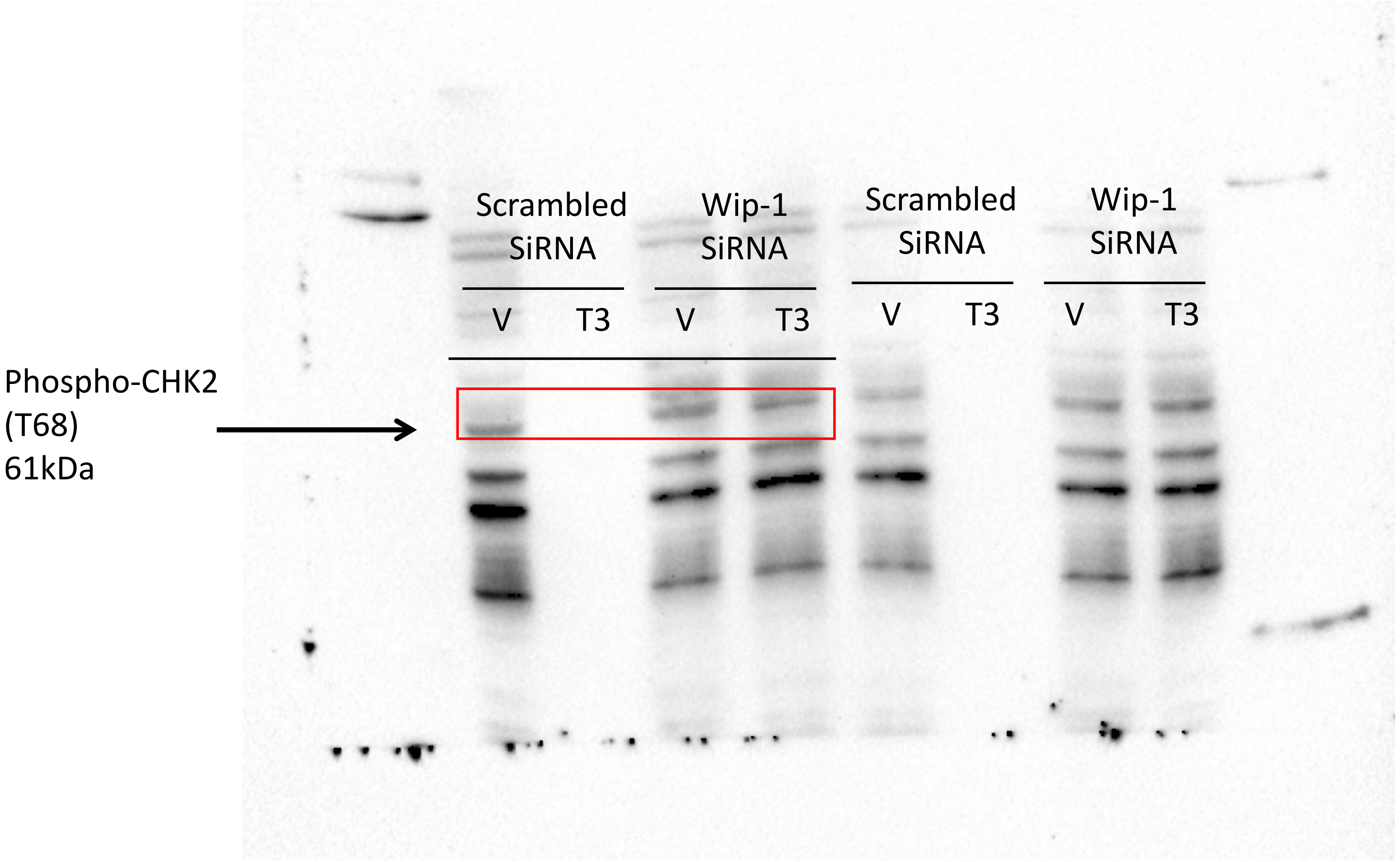

Figure 7C

CM + 10nM T3 +/- Wip-1 SiRNA

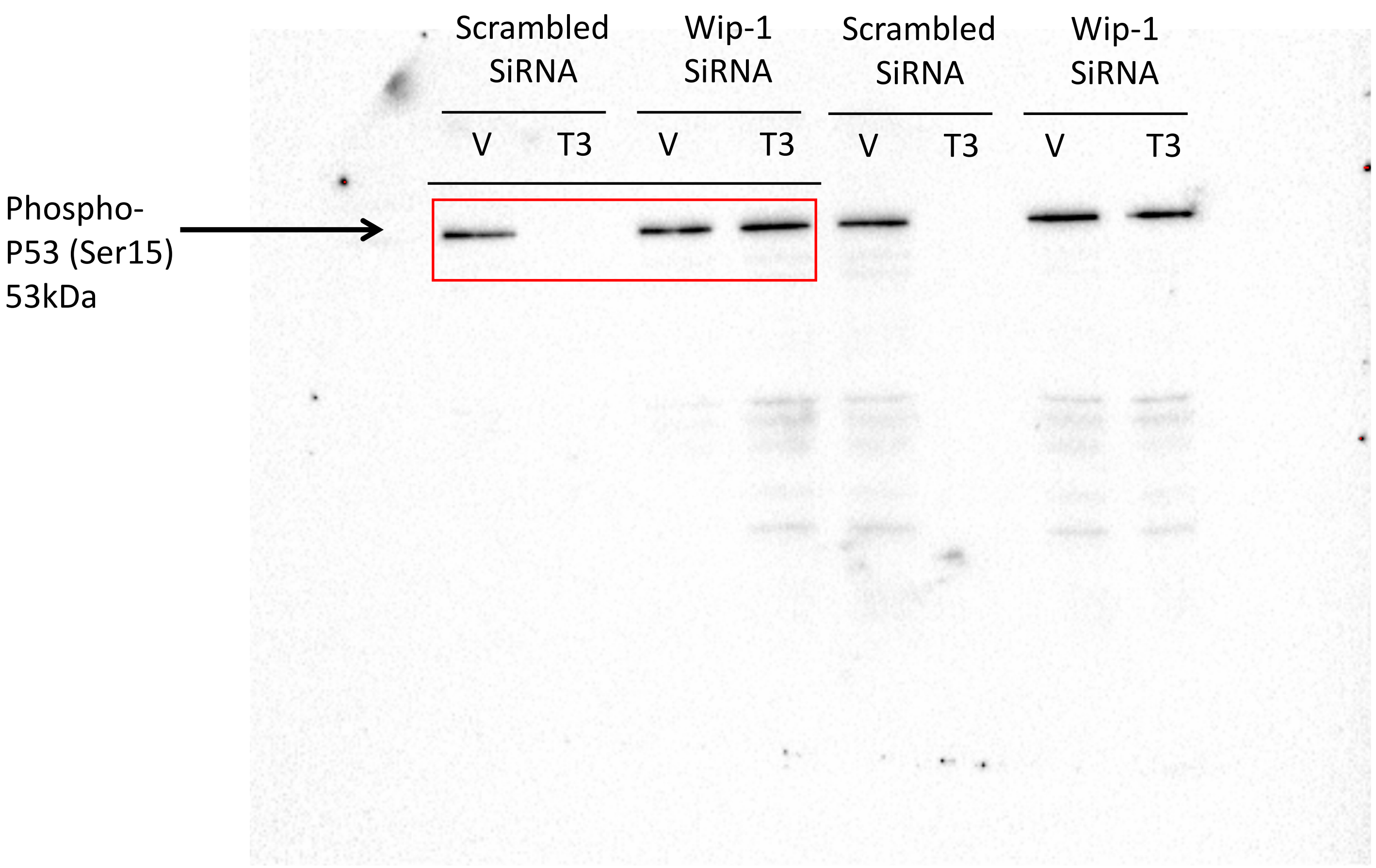

Figure 7C

CM + 10nM T3 +/- Wip-1 SiRNA

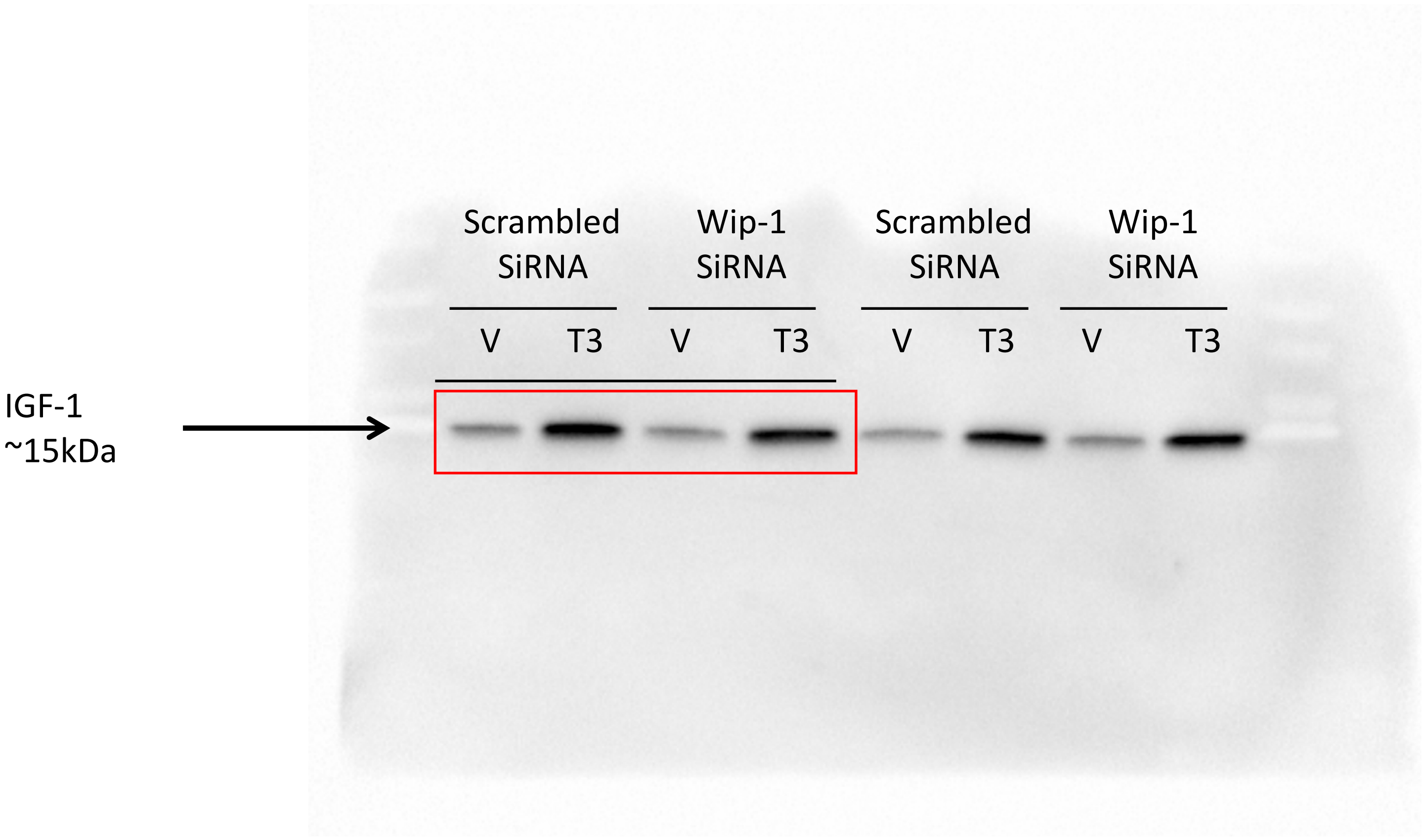

Figure 7C

CM + 10nM T3 +/- Wip-1 SiRNA

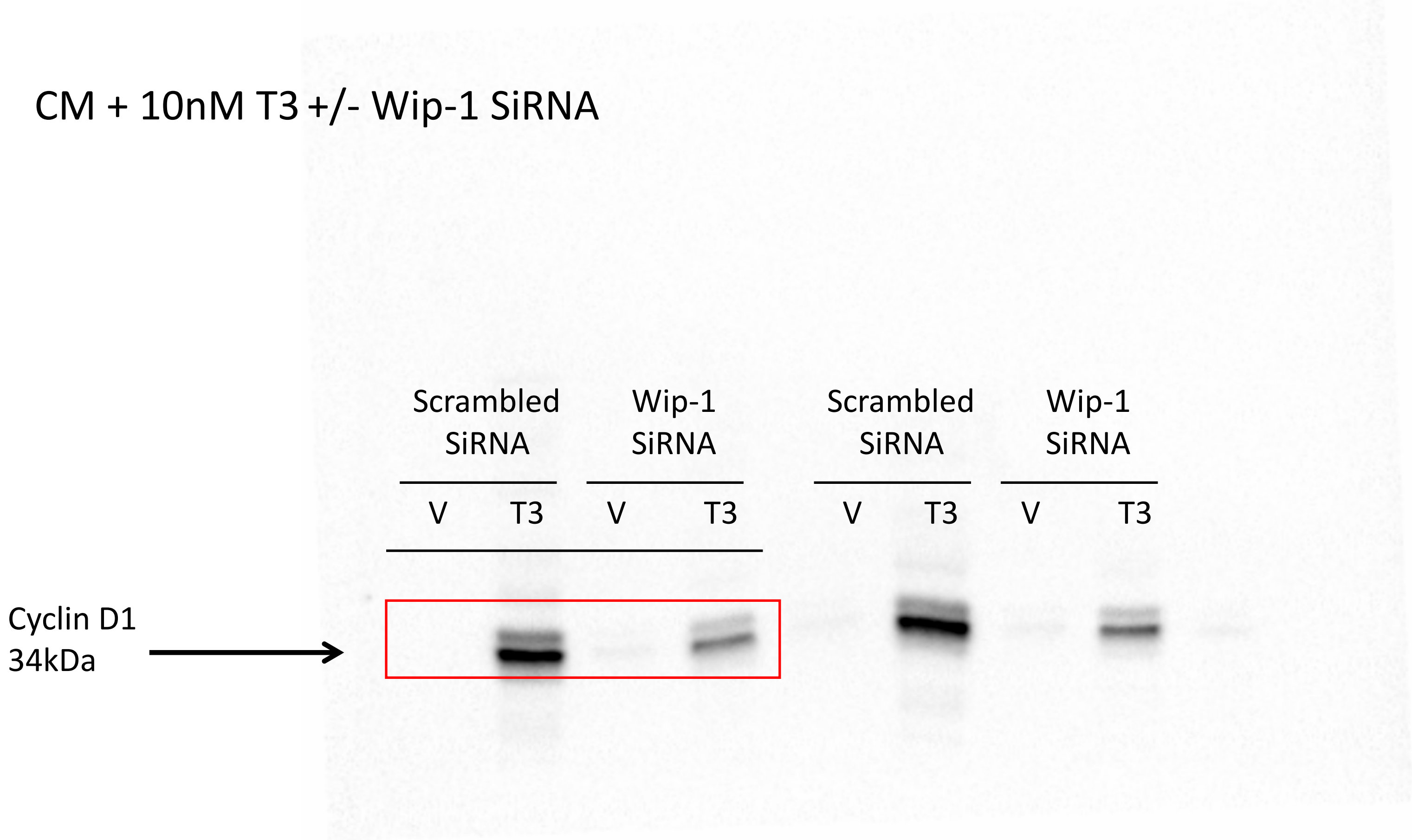

Figure 7C

CM + 10nM T3 +/- Wip-1 SiRNA

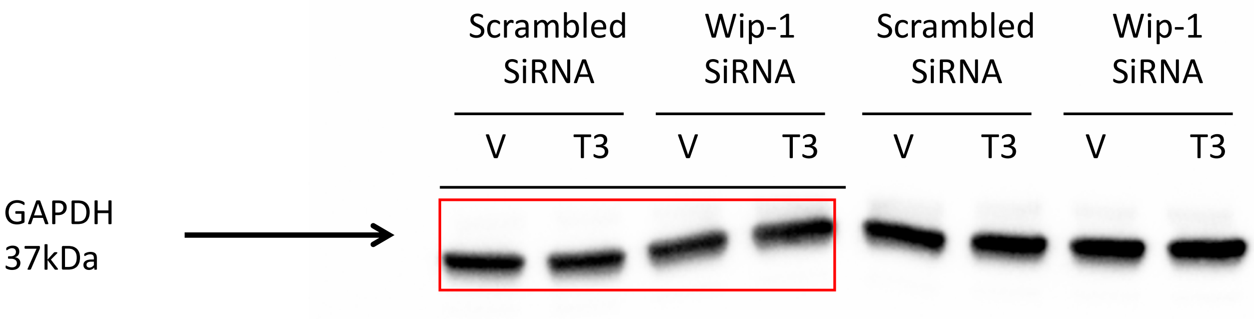

Figure S2B

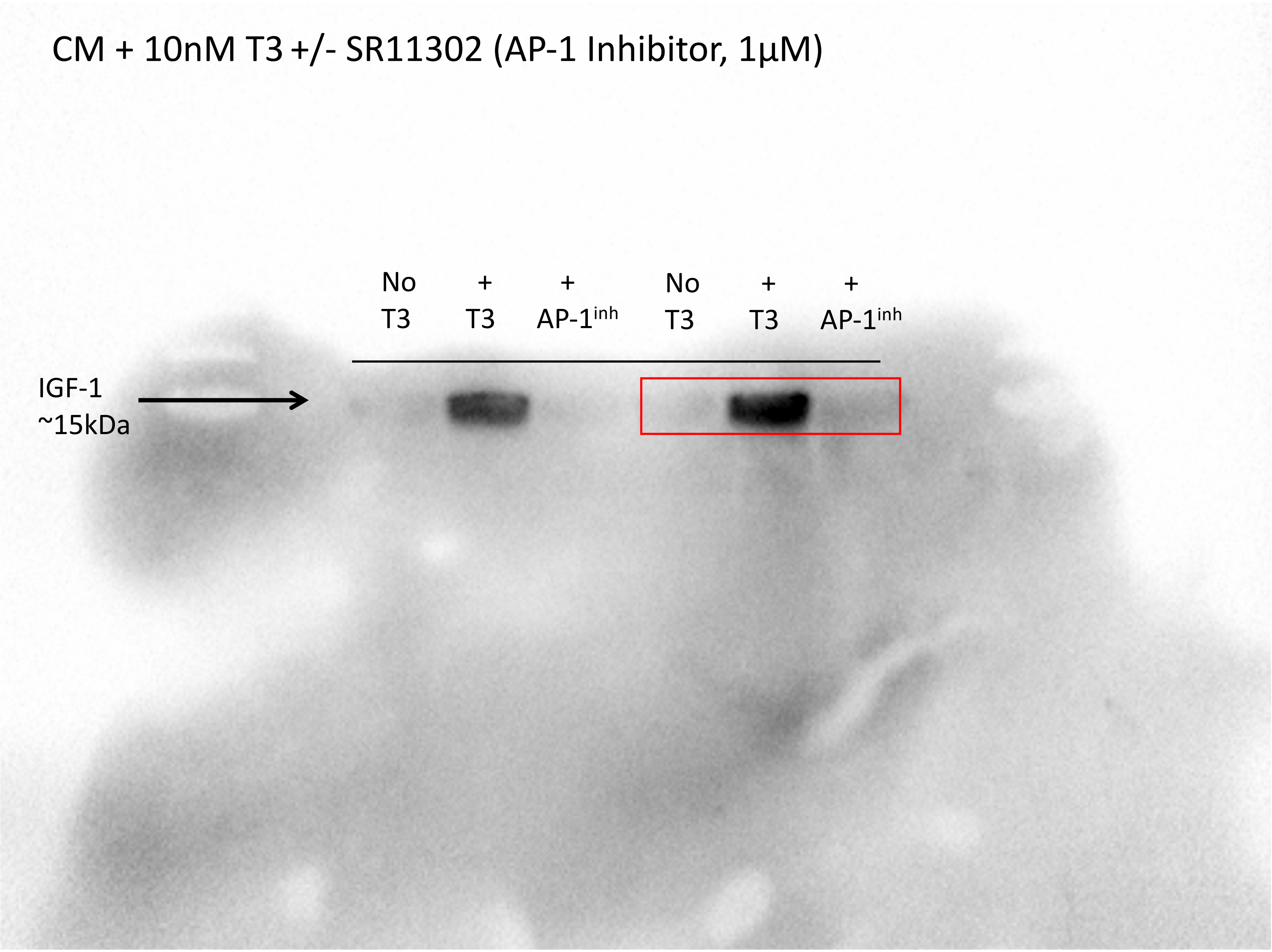
CM + 10nM T3 +/- SR11302 (AP-1 Inhibitor, 1μM)
No T3
+ T3
+ AP-1inh
No T3
+ T3
+ AP-1inh
IGF-1
~15kDa

Figure S2B

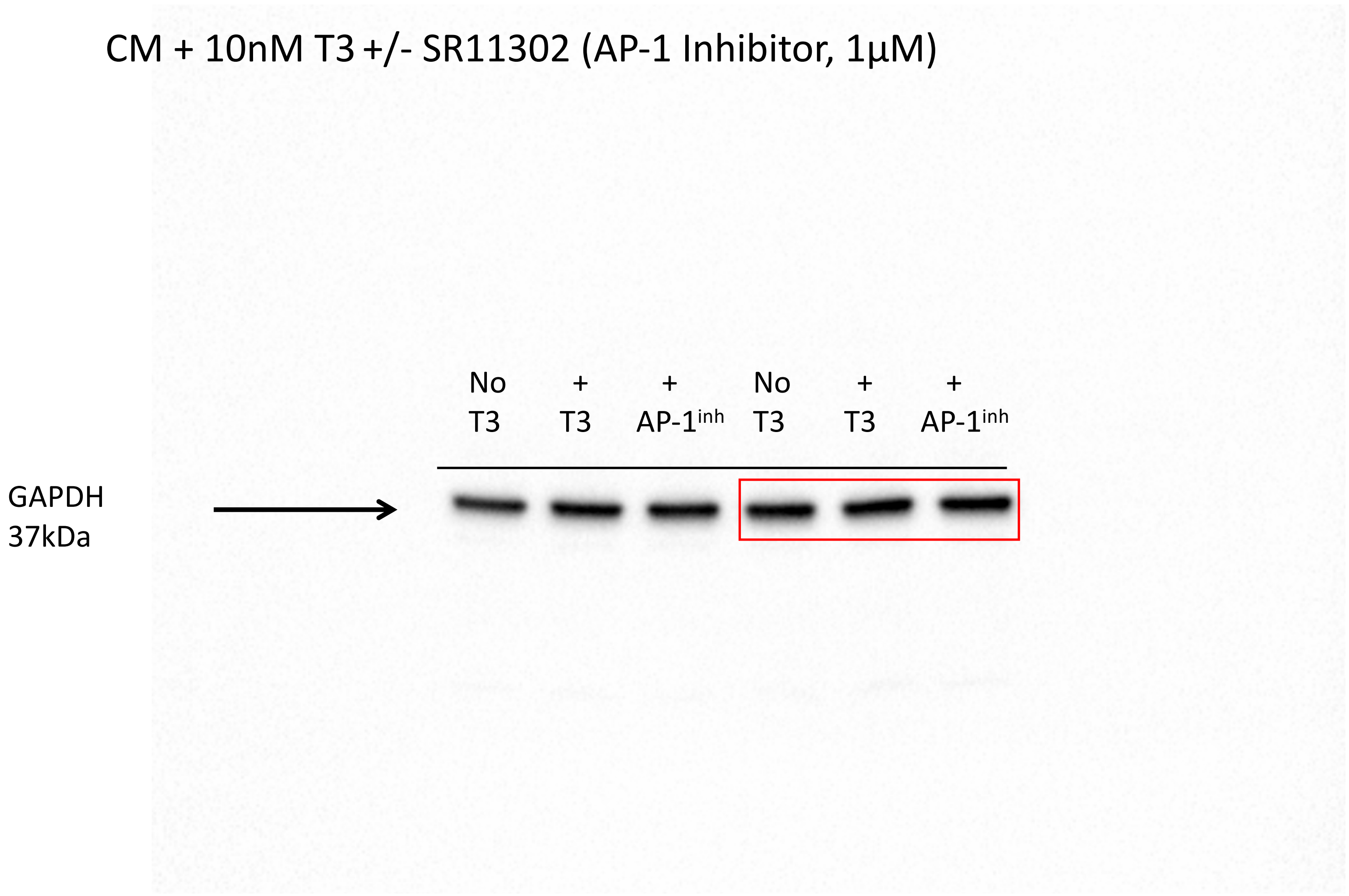

CM + 10nM T3 +/- SR11302 (AP-1 Inhibitor, 1µM)
No T3
+ T3
+ AP-1inh
No T3
+ T3
+ AP-1inh
GAPDH
37kDa

Figure S2C

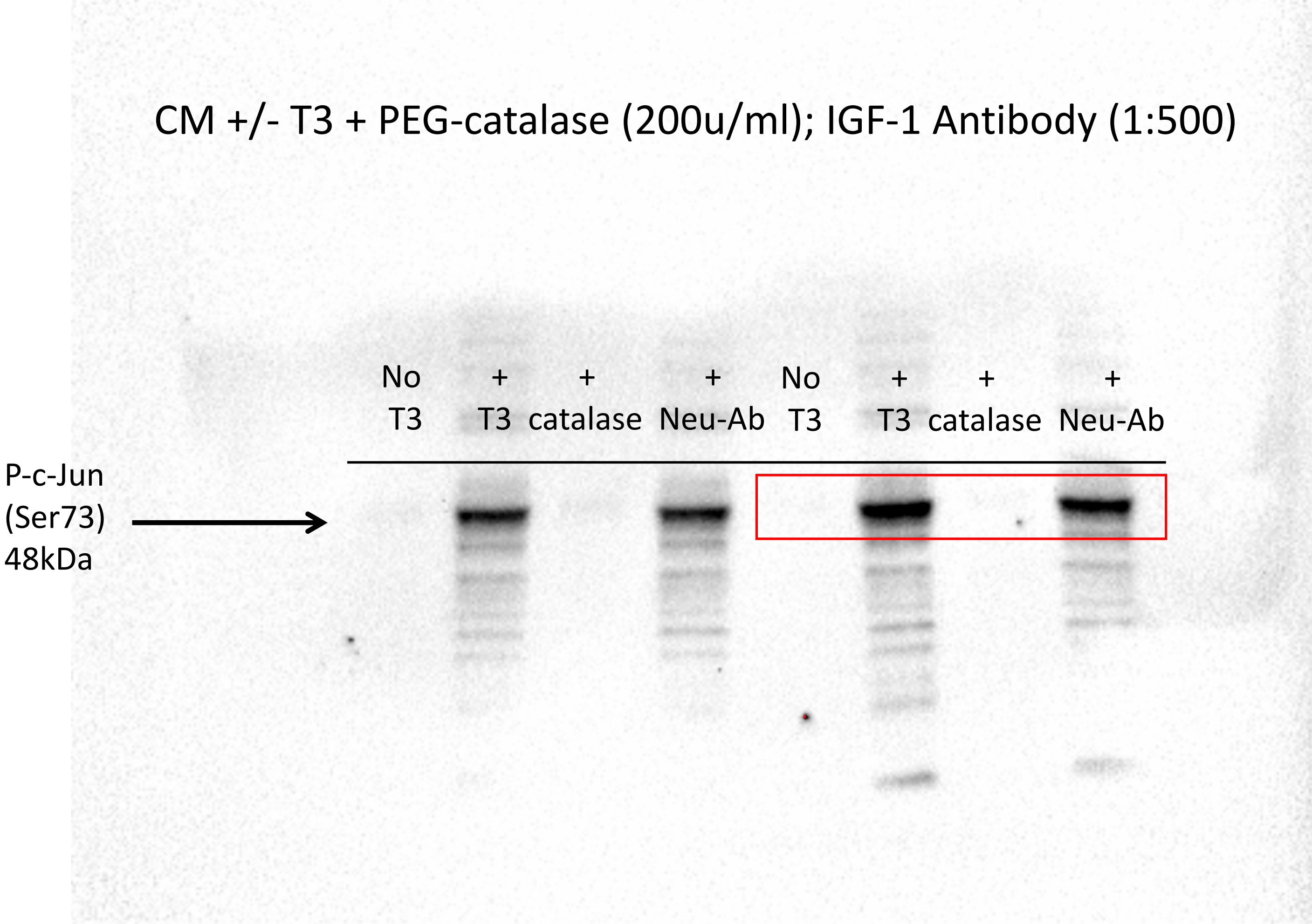
CM +/- T3 + PEG-catalase (200u/ml); IGF-1 Antibody (1:500)
No T3
+ T3
+ catalase
+ Neu-Ab
No T3
+ T3
+ catalase
+ Neu-Ab
P-c-Jun (Ser73) 48kDa

Figure S2C

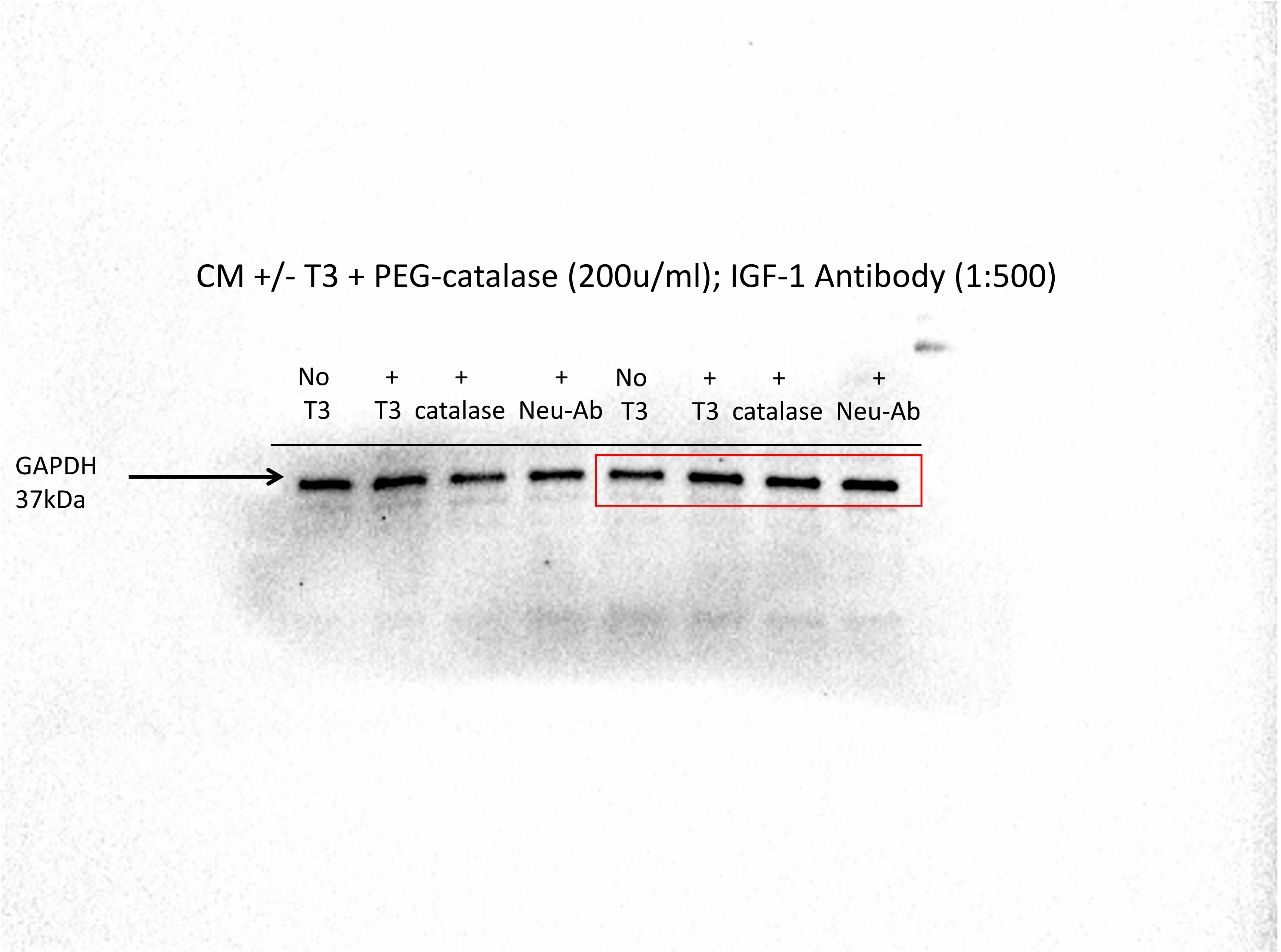
CM +/- T3 + PEG-catalase (200u/ml); IGF-1 Antibody (1:500)
No T3
+ T3
+ catalase
+ Neu-Ab
No T3
+ T3
+ catalase
+ Neu-Ab
GAPDH
37kDa

Figure S2D

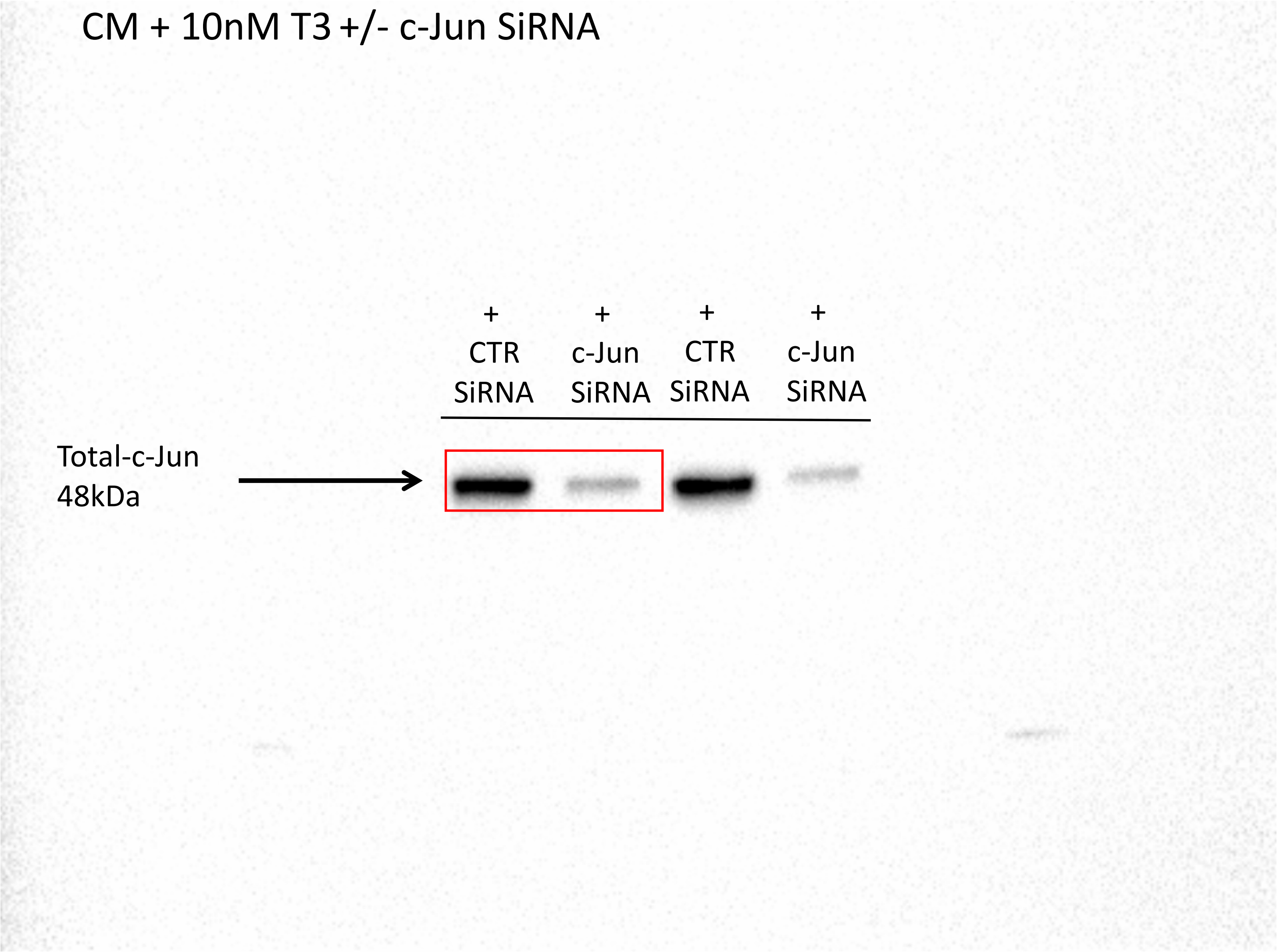

CM + 10nM T3 +/- c-Jun SiRNA
+
CTR
SiRNA
+
c-Jun
SiRNA
+
CTR
SiRNA
+
c-Jun
SiRNA
Total-c-Jun
48kDa

Figure S2D

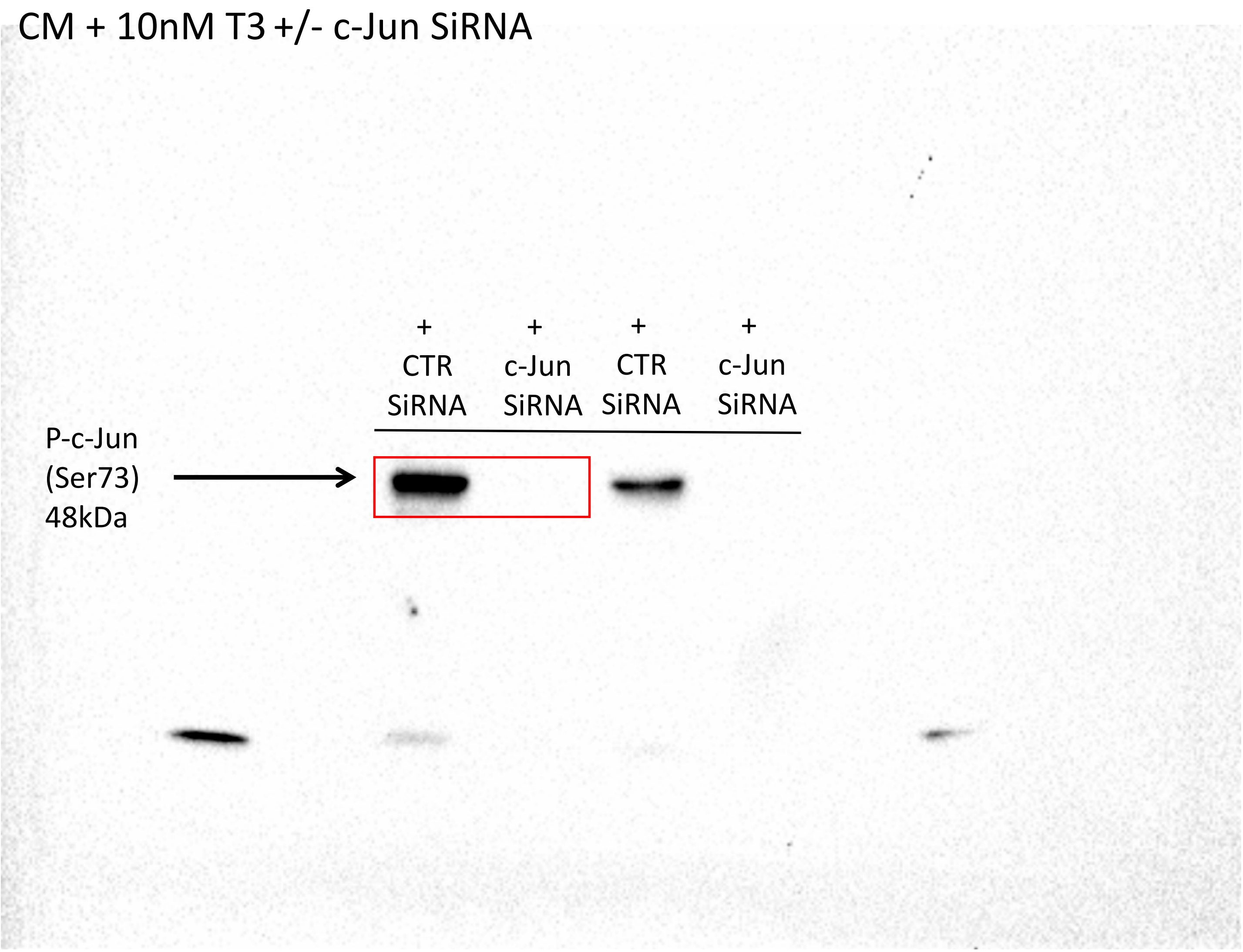
CM + 10nM T3 +/- c-Jun SiRNA
+
CTR SiRNA
+
c-Jun SiRNA
+
CTR SiRNA
+
c-Jun SiRNA
P-c-Jun (Ser73) 48kDa

Figure S2D

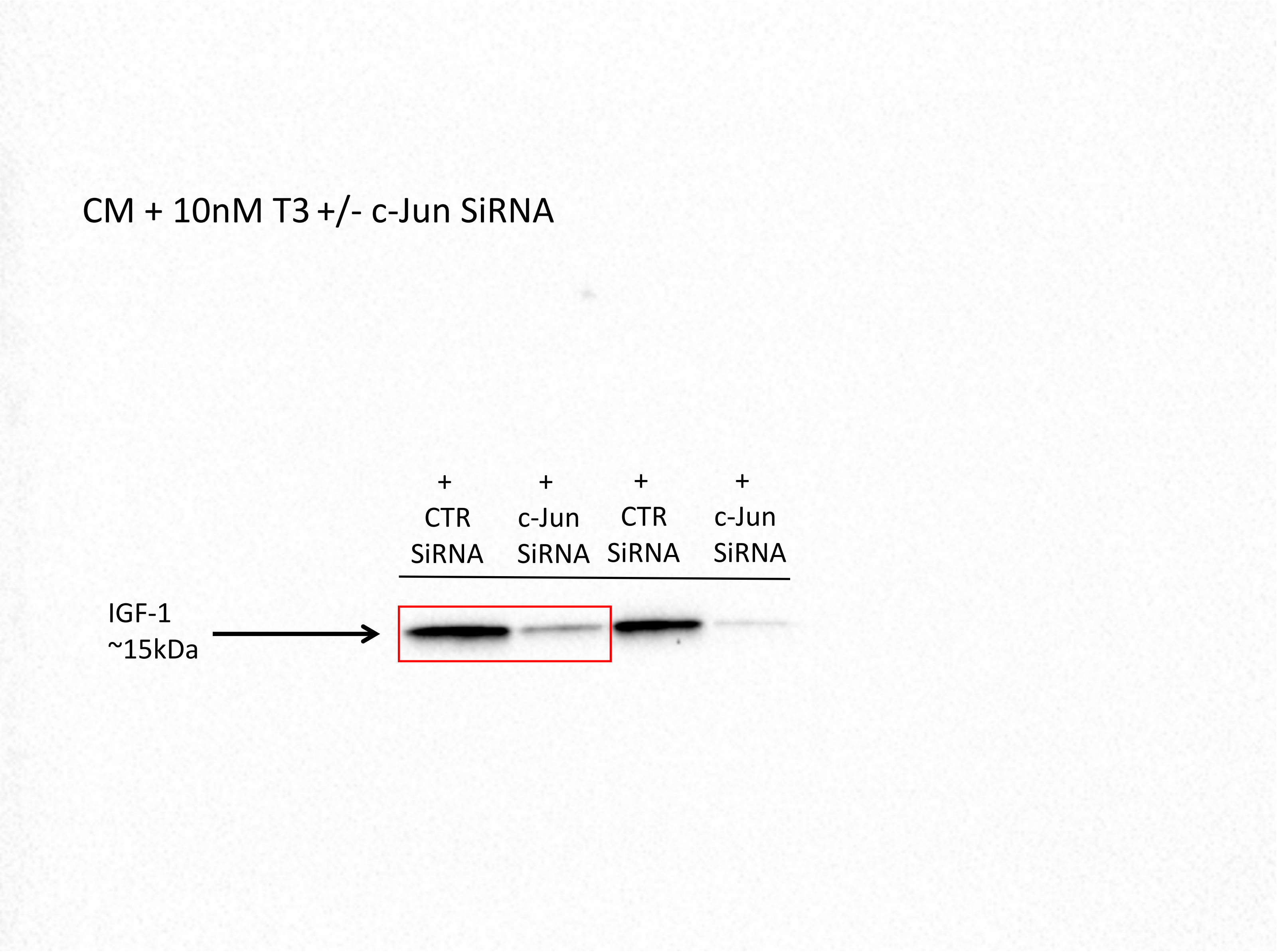
CM + 10nM T3 +/- c-Jun SiRNA
+
CTR SiRNA
+
c-Jun SiRNA
+
CTR SiRNA
+
c-Jun SiRNA
IGF-1
~15kDa

Figure S2D

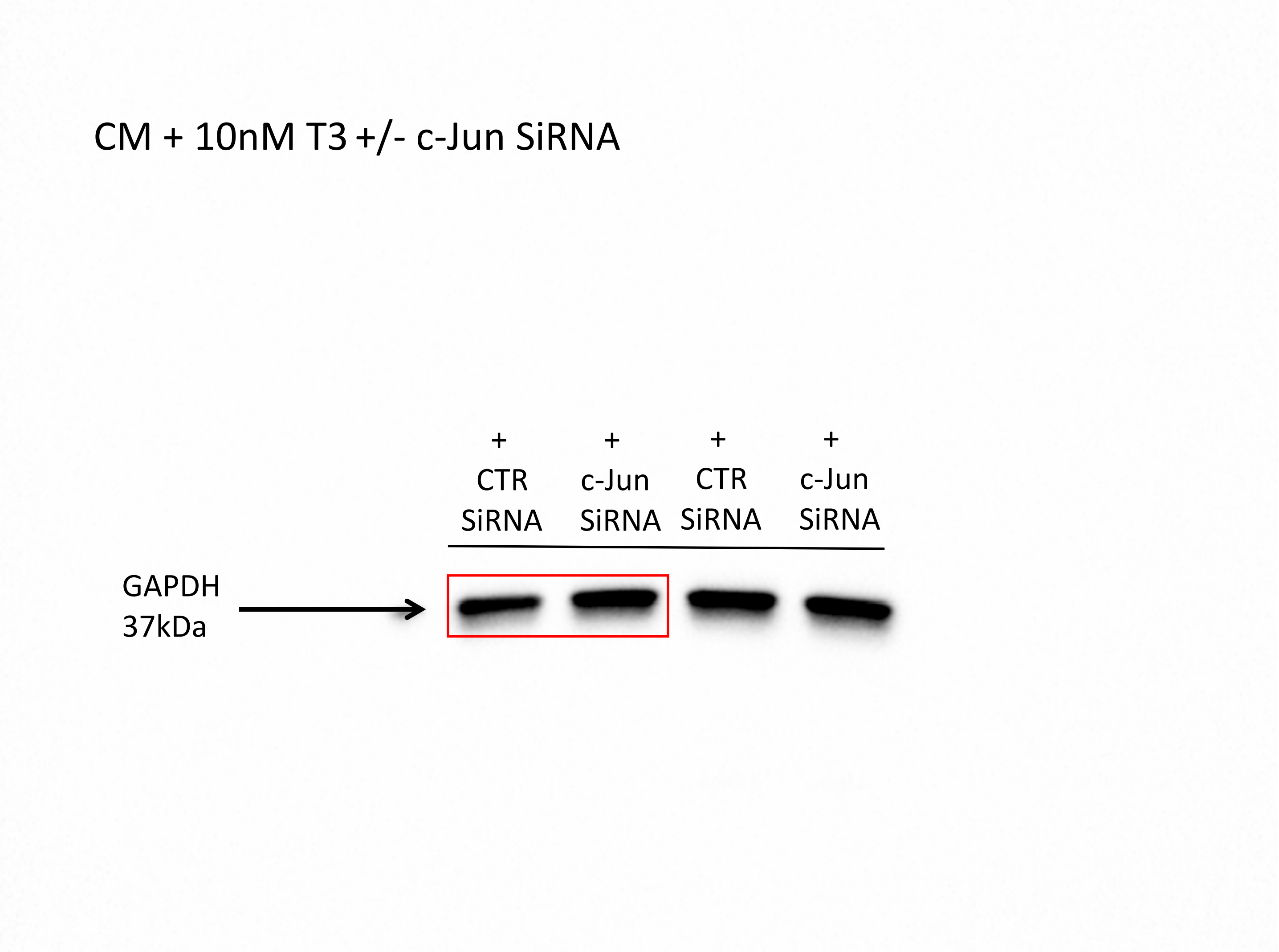
CM + 10nM T3 +/- c-Jun SiRNA
+ CTR SiRNA
+ c-Jun SiRNA
+ CTR SiRNA
+ c-Jun SiRNA
GAPDH 37kDa

Figure S3A

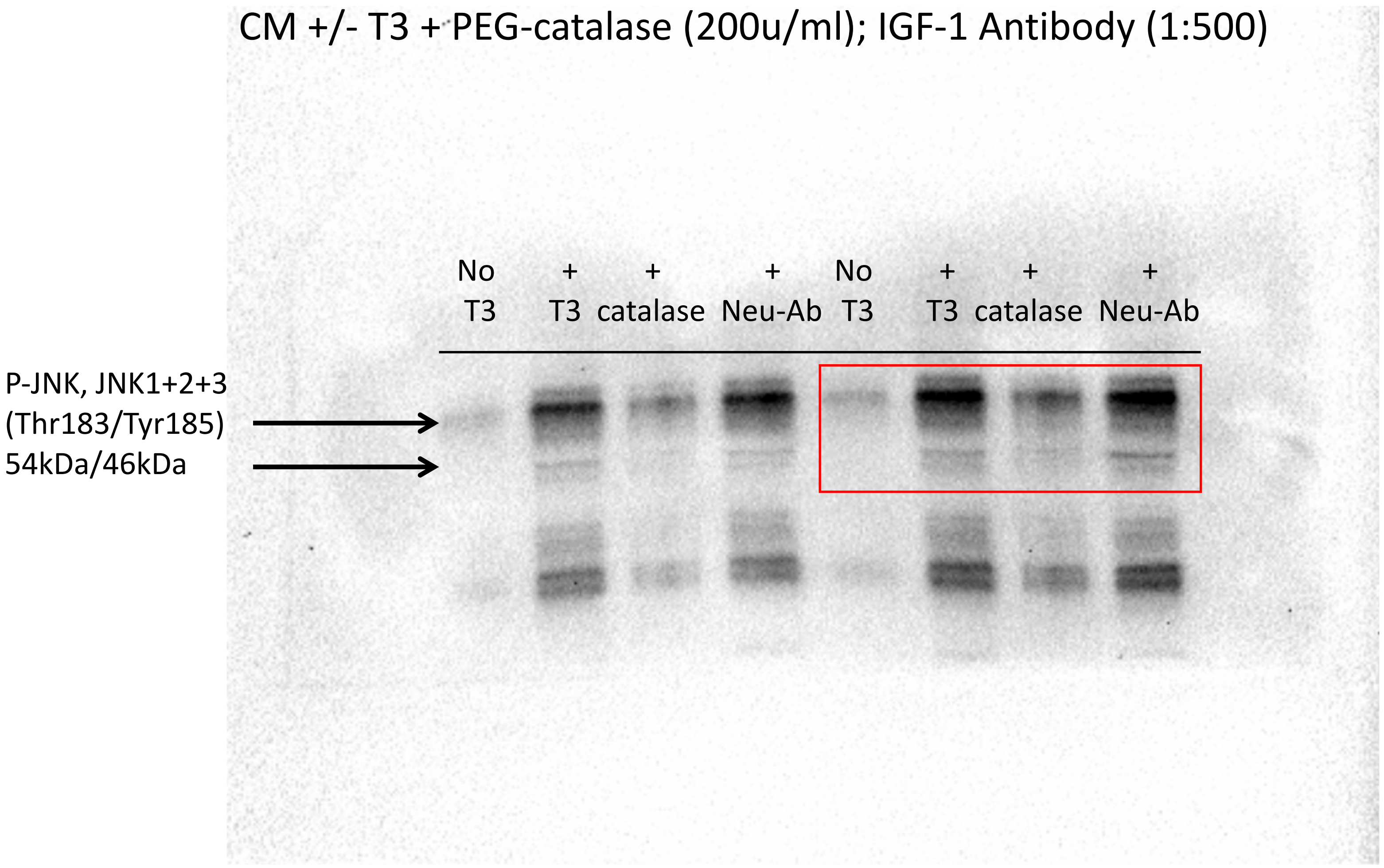
CM +/- T3 + PEG-catalase (200u/ml); IGF-1 Antibody (1:500)
No T3
+ T3
+ catalase
+ Neu-Ab
No T3
+ T3
+ catalase
+ Neu-Ab
P-JNK, JNK1+2+3
(Thr183/Tyr185)
54kDa/46kDa

Figure S3B

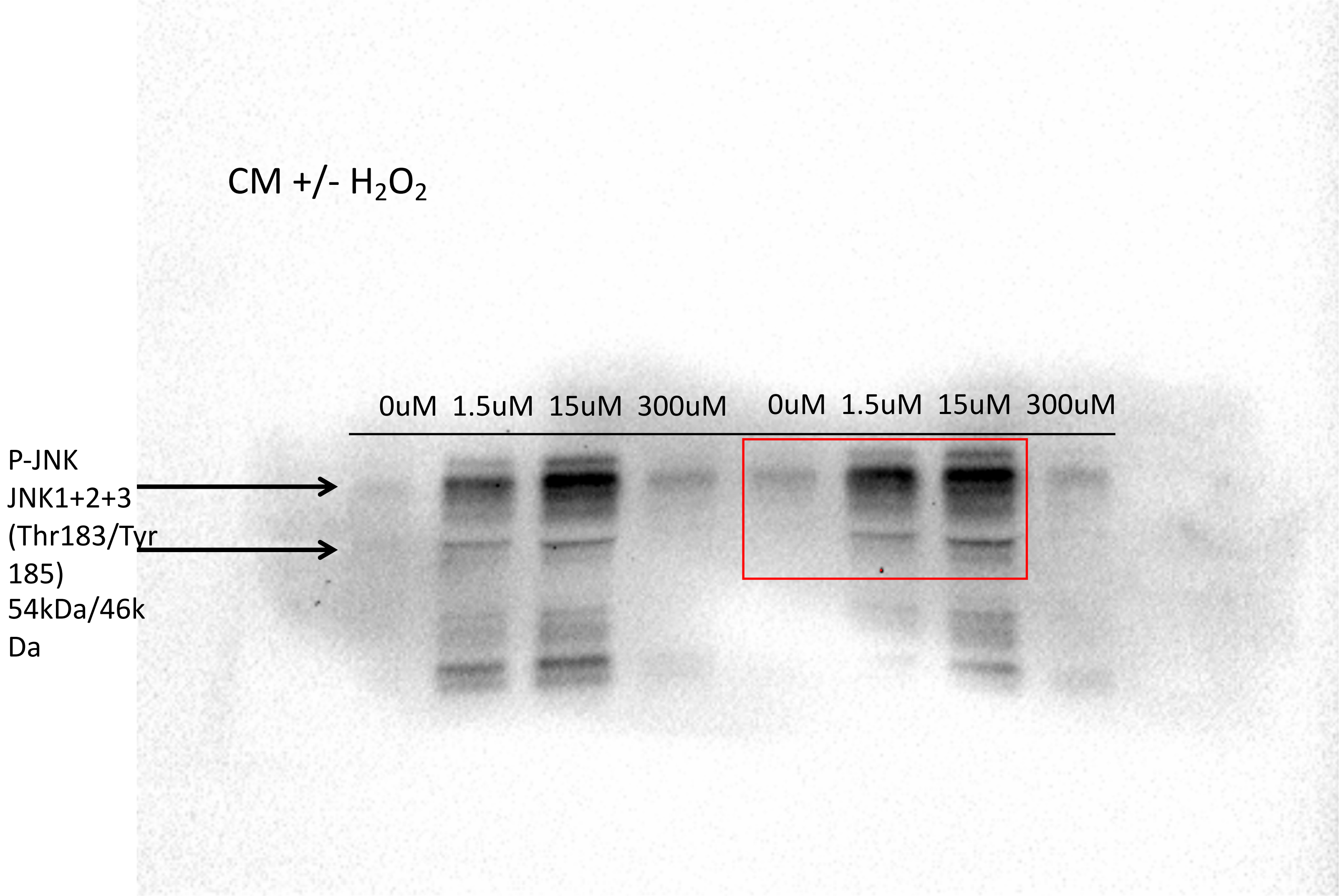
CM +/- H_2O_2
0uM 1.5uM 15uM 300uM 0uM 1.5uM 15uM 300uM
P-JNK JNK1+2+3 (Thr183/Tyr 185) 54kDa/46kDa

Figure S3B

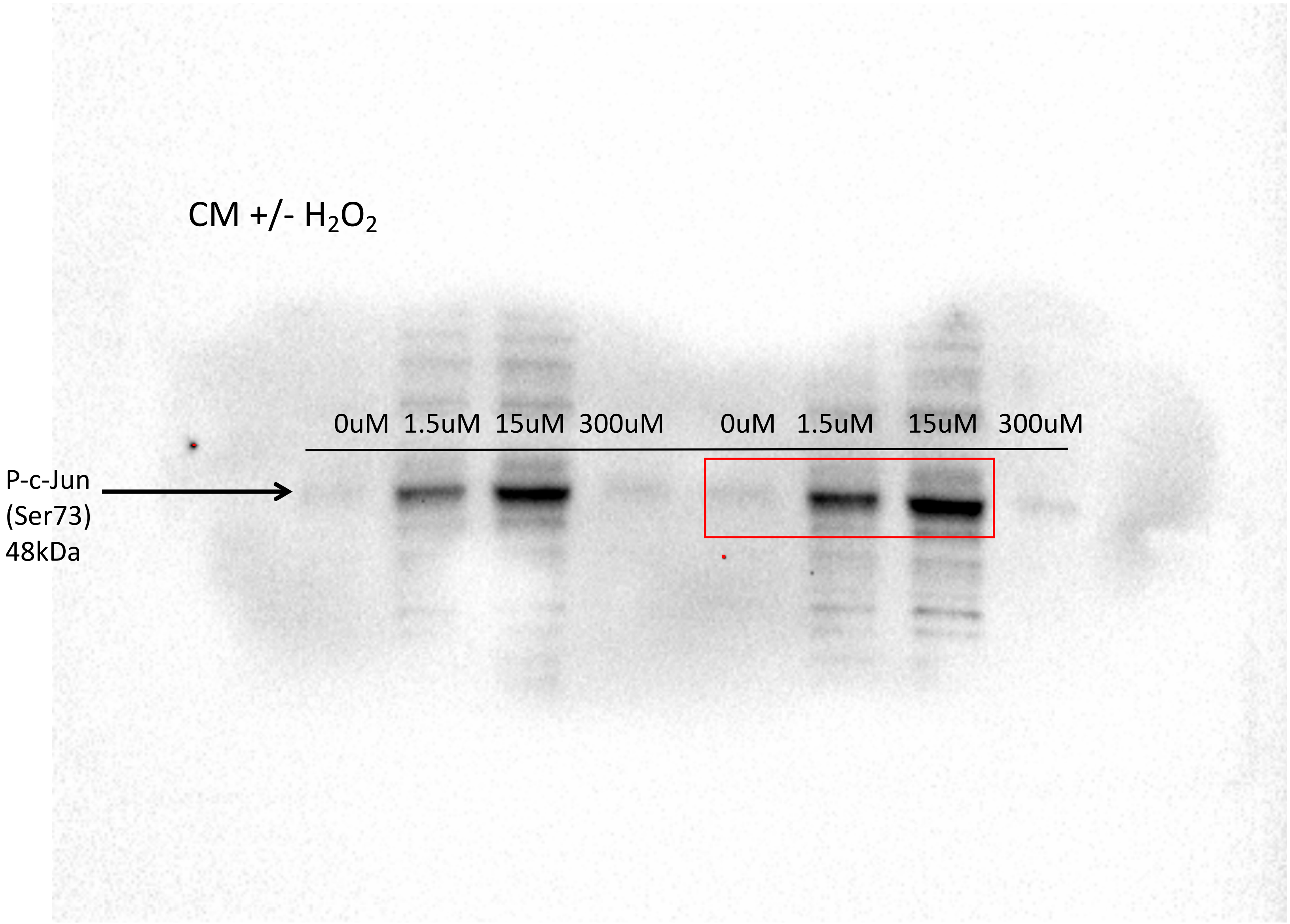
CM +/- H_2O_2
0uM 1.5uM 15uM 300uM 0uM 1.5uM 15uM 300uM
P-c-Jun
(Ser73)
48kDa

Figure S3B

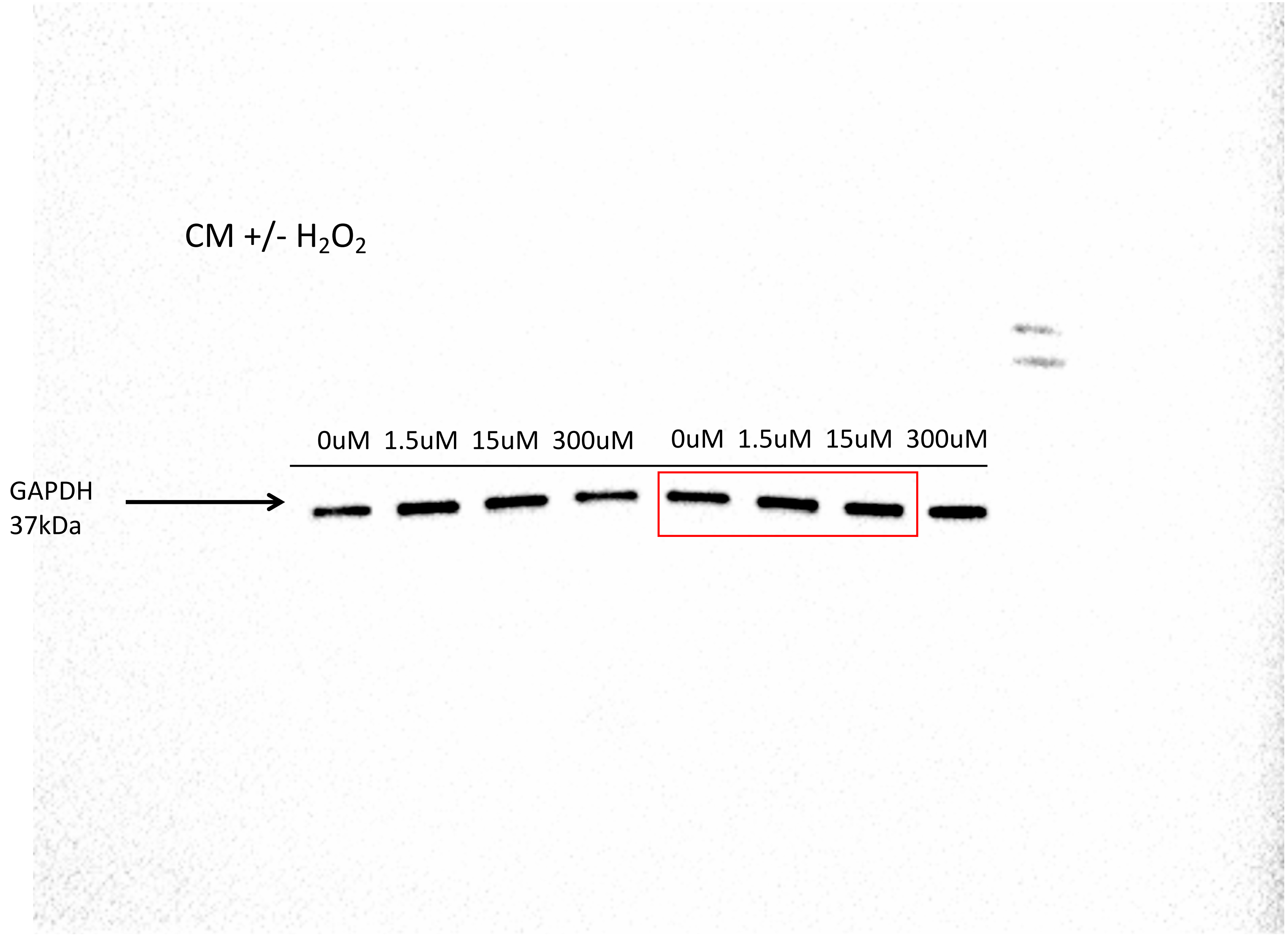

Figure S4

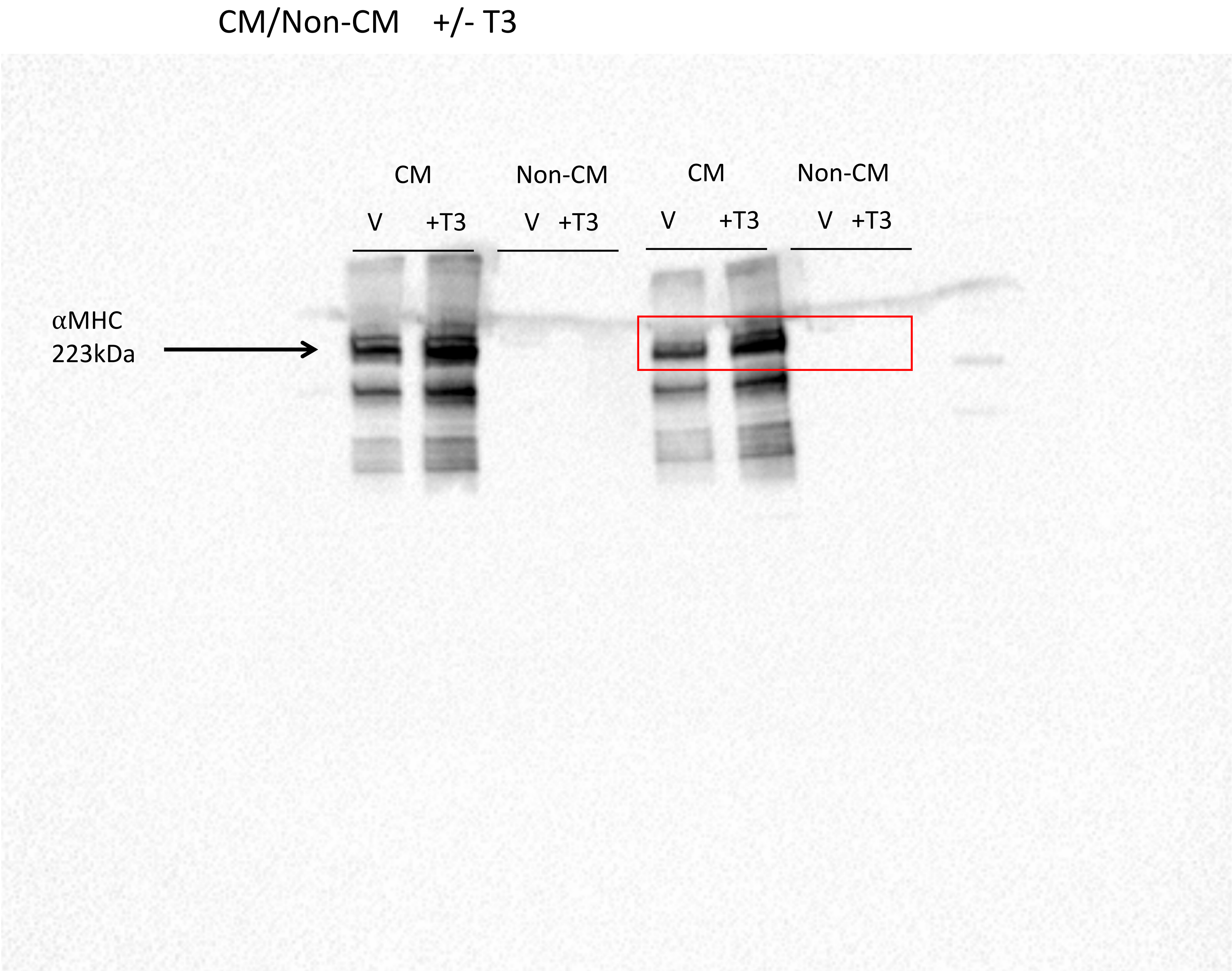

Figure S4

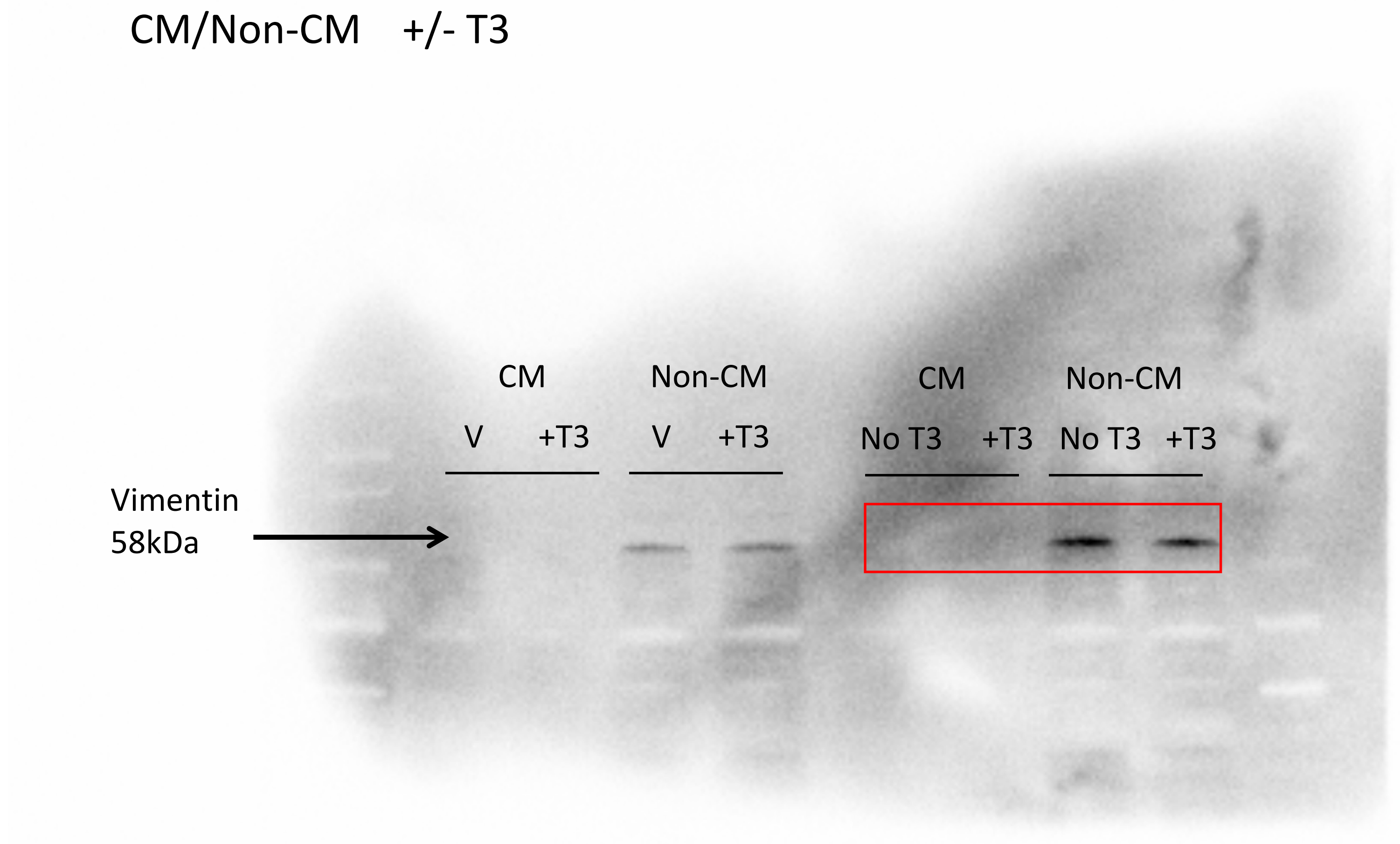

CM/Non-CM +/- T3
CM
Non-CM
V
+T3
V
+T3
CM
Non-CM
No T3
+T3
No T3
+T3
Vimentin
58kDa

Figure S4

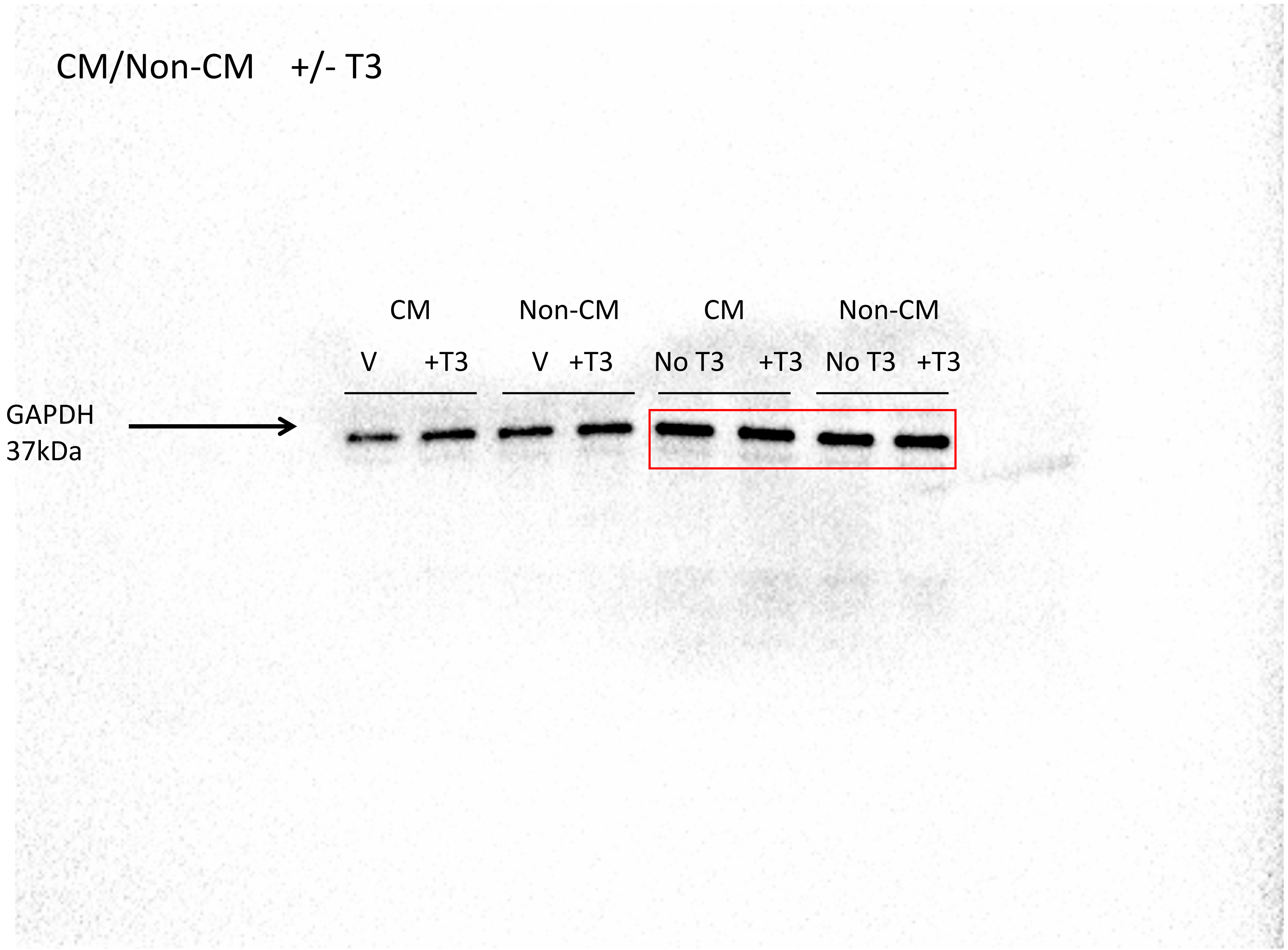

CM/Non-CM +/- T3
CM
Non-CM
CM
Non-CM
V
+T3
V
+T3
No T3
+T3
No T3
+T3
GAPDH
37kDa
